# miRNA as Modifiers of Chromium (Cr) Stress in Mangrove *Avicennia marina*

**DOI:** 10.3390/plants15101451

**Published:** 2026-05-09

**Authors:** Beibei Chen, Quanhu Zhao, Yujian Mo, Qingzhi Liang, Lishan Zhen, Jian Yang, Xiao Xiao

**Affiliations:** 1 College of Coastal Agricultural Sciences, Guangdong Ocean University, Zhanjiang 524088, China; beibeichenk@outlook.com (B.C.); 15296852400@163.com (Q.Z.); moyujian@163.com (Y.M.); qingzhi2002@163.com (Q.L.); ls636923@163.com (L.Z.); yangjian@stu.gdou.edu.cn (J.Y.); 2 School of Chemistry and Environment, Guangdong Ocean University, Zhanjiang 524088, China

**Keywords:** morpho-physiological responses, chromium stress, miRNA, target gene, regulatory model, *Avicennia marina*

## Abstract

Chromium (Cr) is one of the most toxic heavy metals in the environment. The tolerance to metal stress involves sophisticated regulation of gene expression networks, which involve microRNAs (miRNAs). However, the role of miRNAs in Cr stress response in *Avicennia marina* has not been resolved, and was addressed here. The analysis of response characteristics revealed that morpho-physiological traits such as root length, Cr accumulation level and antioxidant enzyme activity all exhibit significant changes under Cr stress. Via sRNA sequencing, a total of 27 known and 149 novel miRNAs were identified, 63 of which showed differential expression after Cr stress (q-value < 0.001). Further, 571 miRNA-target interaction pairs were identified for differentially expressed miRNAs, corresponding to 355 target genes. GO and KEGG analyses indicated that these target genes could participate in stress-related biological processes such as signal transduction, transcription regulation, protein synthesis and the MAPK signaling pathway. 54 miRNA target genes, corresponding to 37 miRNAs such as Ama-miR160, Ama-nmiR25-5p and Ama-nmiR118-5p, were enriched for “plant signal hormone transduction” (ko04075), “phenylpropanoid biosynthesis” (ko00940) and “MAPK signaling pathway” (ko04016), which indicated an important role of these miRNAs in regulating Cr stress response in *A. marina*. Based on the findings, a Cr stress-responsive regulatory model was developed, offering new insights into the molecular regulatory mechanisms of Cr response. In conclusion, this study shows the identity and potential role of miRNAs in the heavy metal stress response of *A. marina*, and provides the foundation for future research.

## 1. Introduction

Heavy metal pollution can cause osmotic stress, ion toxicity, membrane permeability changes and physiological metabolism disorders, which could seriously restrict plant growth, production and cultivation. Chromium (Cr) is a common industrial metal, which is characterized by accumulations that are long-term, hidden, difficult to remove, and cause irreversible damage [1]. Among the six toxic heavy metals, Cr ranks second and is extremely harmful. The common forms of Cr in soils are Cr^3+^, which is the main form [2]. Cr (III) compounds are known to have strong oxidative activity, and their excessive accumulation in plants can cause serious harm to plant morphology, physiology and biochemical processes, including inhibition of seed germination and plant growth, damage of cell ultrastructure, oxidative stress, and interference with mineral nutrient absorption [3,4]. Therefore, it is of great practical significance to explore the molecular mechanism of Cr resistance in plants, including regulations by microRNAs (miRNAs), identify Cr-tolerance related genes and new germplasm resources, and utilize them in breeding new varieties to alleviate the problem of soil metal pollution.

miRNAs are a class of endogenous, approximately 19–24 nt non-coding small RNAs that negatively regulate gene expression at transcriptional and post-transcriptional levels, mainly through binding to targets by a complementary base pairing mechanism [4]. miRNAs are widely distributed in plant genomes and play a key role in gene expression regulatory networks. Studies have shown that miRNAs play an important regulatory role in plant growth and development, hormone secretion, signal transduction and other biological processes [5]. In particular, multiple miRNAs are now known to regulate various physiological activities of plants in response to metal stress. For instance, miR-396, miR-398, miR-399 and miR-408 are responsive to cadmium (Cd) and arsenic stresses in *Oryza stiva* [6,7]. miR393 regulates root sensitivity to aluminum by changing auxin signal output in barley [8]. Transgenic *Arabidopsis thaliana*, overexpressing a miR156, reduces Cd accumulation in aerial parts, thereby enhancing plant tolerance to Cd stress [9]. So far, many studies have demonstrated that miRNAs are sensitive to multiple heavy metals such as Cd, Hg and Al in plant species, including *A. thaliana*, *O. stiva* and *Medicago truncatula* [9,10,11]. In recent years, multiple studies have claimed that miRNAs could regulate Cr stress responses in plants. For instance, in rice, 13 conserved miRNAs are preferentially expressed under Cr stress [12]. Liu et al. [13] identified 81 known miRNAs and 72 novel miRNAs in radish by high-throughput transcriptome sequencing, of which 54 known and 16 novel miRNAs have significant differential expression under Cr stress. Bukhari et al. [14] have analyzed two tobacco genotypes with different Cr tolerance levels by Illumina/Solexa1G genome analyzer (Illumina) deep sequencing method to study the potential role of miRNAs in Cr tolerance. However, miRNA involvement in mangrove tolerance response against Cr stress remains uncharacterized.

Mangroves are woody plants that grow in tropical intertidal zones and have diversified ecological functions, such as wave protection, silting promotion, and purification of water and air. However, due to the special geographical environment of the wetlands and their unique ecological systems, mangrove areas are often easy to become “high incidence places” for heavy metal accumulations [15]. Mangrove plants can absorb metals within a certain range, and thus reduce their environmental toxicity [16]. But the accumulation of excessive metal ions affects the dynamic ion balance in the plants and inhibits their normal growth and development [17]. Through long-term adaptation to high-metal intertidal habitats, mangroves are likely to have evolved a unique regulatory system that markedly differs from those of model plants. However, it is noteworthy that current studies on miRNA-mediated Cr responses are predominantly focused on glycophytic crops, such as rice and radish; these crops, in contrast to mangroves, are devoid of the specialized metal-tolerance mechanisms that are intrinsic to mangroves. *Avicennia marina* is a female shrub belonging to the genus Phallocarpa of the family Araceae. It has the characteristics of fast growth, strong metal tolerance and submergence tolerance. It is a pioneer species of the mangroves and the most widely distributed mangrove species in China. At present, most of the studies on heavy metal Cr toxicity of *A. marina* are limited to evaluating its toxic effect by morphological indicators, growth volume and growth status [18]. The identity of miRNAs involved in the Cr stress response of *A. marina* has not been analyzed yet. Consequently, the elucidation of the miRNA profiles in *A. marina* under chromium (Cr) stress will not only address a pivotal void in the field of mangrove molecular biology but also offer profound evolutionary insights into the convergence and divergence of heavy metal tolerance mechanisms across plants.

In this study, the impact of Cr stress on miRNA accumulation patterns of *A. marina* seedlings was evaluated with the help of small RNA (sRNA) sequencing technology, and bioinformatics methods were used to mine the important miRNAs involved in Cr stress-mediated regulation of various pathways. Finally, a Cr stress miRNA-target gene regulatory network was constructed. The study is helpful to further explore the molecular regulatory mechanism of heavy metal stress in mangrove plants. The understanding of the molecular regulatory mechanism of Cr stress in *A. marina* will be helpful for resistance breeding programs of mangrove plants.

## 2. Results

### 2.1. Phenotypic and Physiological Responses of A. marina to Chromium Stress

**Figure 1:**
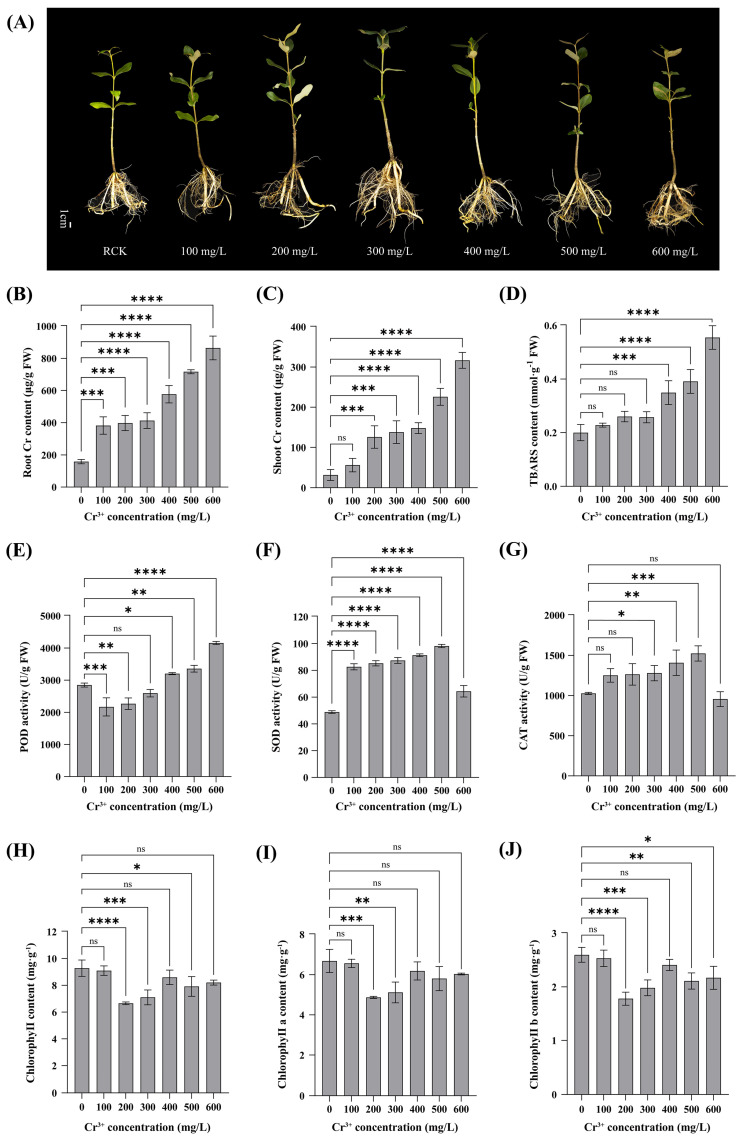
Effects of different concentrations of chromium treatment on physiological and biochemical indices in roots of *A. marina*. (**A**) Growth phenotype of *A. marina* under different concentrations. (**B**) Total Cr content in root. (**C**) Total Cr content in shoot. (**D**) TBARS content. (**E**) POD activity. (**F**) SOD activity. (**G**) CAT activity. (**H**) Total chlorophyll content. (**I**) Chlorophyll a content. (**J**) Chlorophyll b content. ns, are not significant difference between treatment and control groups; * significant difference at *p* < 0.05; ** *p* ≤ 0.01, *** *p* ≤ 0.001, **** *p* ≤ 0.0001; “ns” indicates no significant difference (*p* > 0.05).

To observe the growth performance of *A. marina* under chromium stress at different concentrations, the growth phenotypes of the plants were observed and recorded after 14 days of Cr treatment. As shown in Figure 1A and Table S1, compared with the control group (RCK), plants treated with 200 mg/L Cr^3+^ (RT1) maintained green and healthy leaves, with no significant differences in plant height or biomass, and no obvious stress symptoms were observed. At 400 mg/L Cr^3+^ (RT2), the leaves began to exhibit slight chlorosis, the leaf area was slightly reduced, and some leaf margins became wrinkled. Quantitatively, at this concentration, plant height, root length, and root dry biomass were not significantly different from the control (Table S1). Under 600 mg/L Cr^3+^ stress (RT3), leaf chlorosis was obvious, leaf number decreased, some leaves withered, plant height was slightly reduced, although the difference was not statistically significant compared to the control (*p* > 0.05). It should be noted that although leaf chlorosis, reduced leaf number, and partial wilting were visually observed in the 600 mg/L Cr^3+^ treatment (RT3), shoot dry biomass did not differ significantly from the control (*p* > 0.05, Table S1). This apparent discrepancy may be attributed to compensatory mechanisms such as increased thickness or density of the remaining leaves and stems, which helped maintain total dry mass. Additionally, chlorophyll degradation (as indicated by chlorosis) may precede actual tissue loss. In the root system, severe browning and a substantial loss of fibrous roots were visually observed, indicating significant damage caused by Cr^3+^ stress. However, the overall root dry biomass was not significantly affected (*p* > 0.05, Table S1). This discrepancy suggests that the visual loss of fine, fibrous roots may be compensated by an increase in the biomass or density of the remaining thicker root axes. The severe browning, while a clear sign of tissue damage, does not necessarily equate to an immediate reduction in dry mass.

With increasing Cr^3+^ concentration, Cr content in both roots and shoots of *A. marina* increased significantly compared with the control (Figure 1B,C). Root Cr content was much higher than that in shoots, reaching a maximum of 864.35 μg/g at 600 mg/L, while the maximum in shoots was 316.58 μg/g; Thus, root Cr content was approximately 2.73 times that in shoots. Thiobarbituric acid reactive substances (TBARS) content showed a continuous increasing trend with elevated Cr^3+^ concentration and began to differ significantly from the control at 400 mg/L (Figure 1D). Compared with the control, POD activity decreased significantly at 100 and 200 mg/L, showed no obvious change at 300 mg/L, increased markedly at 400 mg/L, and peaked at 600 mg/L (Figure 1E). SOD activity was the lowest in the control, approximately 48.82 U/g and reached a peak of 98.12 U/g at 500 mg/L, nearly twice that of the control. CAT activity first increased and then decreased with increasing Cr concentration, but remained higher in the 100, 200, 300, 400, and 500 mg/L treatments than in the control (Figure 1F). CAT activity peaked in the 500 mg/L treatment, showing a significant increase of 48.46% compared with the control (*p* < 0.05). In contrast, the 600 mg/L treatment exhibited a 6.89% reduction relative to the control, with no significant difference (*p* > 0.05) (Figure 1G). Additionally, chlorophyll content in leaves was also markedly affected by Cr stress (Figure 1H–J). Under Cr stress, total chlorophyll content decreased from 9.28 (RCK) to 6.65 mg/g FW at 200 mg/L (RT1), recovered partially to 8.59 mg/g FW at 400 mg/L (RT2), and was 8.20 mg/g FW at 600 mg/L (RT3) (Figure 1H). Although chlorophyll levels in RT2 and RT3 showed a partial recovery relative to RT1, they remained consistently lower than the control. A similar non-monotonic trend was observed for both chlorophyll a and chlorophyll b (Figure 1I,J). Notably, leaf chlorosis was visually observed in the RT2 and RT3 treatments, which is consistent with the measured reduction in chlorophyll content.

### 2.2. Analysis of sRNA Sequencing Data of A. marina

After being treated with different concentrations of CrCl_3_ (0, 200, 400, 600) mg/L for 14d, *A. marina* samples were collected, and a total of 12 miRNA libraries were sequenced. As shown in Table 1 and Figure S1, about 361,751,395 reads were originally obtained, from which 313,960,410 clean reads were retained after filtering. Q20 and Q30 were up to 98.5% and 94.6%, respectively, indicating the high quality of the sequencing data from these samples. The sequencing data were aligned to the reference genome using Bowtie software (v1.1.2) [19] with an average alignment rate of 72.78% (Table 1). Pearson correlation coefficients were high (above 0.95) between all three biological replicates for each condition (Figure S2). These parameters indicated that the sequencing quality of the 12 samples was relatively high and that the data were reliable for subsequent differential expression analysis.

### 2.3. Identification of Known miRNAs in A. marina

In order to identify known miRNAs, we aligned the sRNA sequences with the genome of *A. marina*. Also, we aligned all the known plant miRNAs with the reference genome sequences and miRBase, RNAcentral and Rfam databases. The classification and annotation of sRNAs revealed that the majority of sRNAs were annotated as rRNA, miRNA, snoRNA, and tRNA. Specifically, 1,027,878 sRNAs, representing 1.87% of the entire sRNA population, were annotated as miRNAs (Figure S3A).

**Figure 2:**
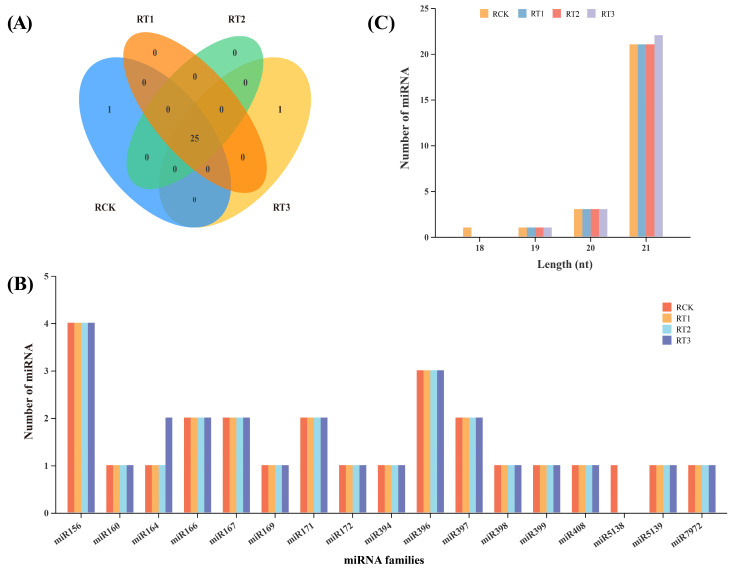
Identification of conserved miRNAs in *A. marina*. (**A**) Distribution of conserved miRNAs in *A. marina* under the control and 200, 400 and 600 mg/L Cr stress conditions. (**B**) Length distribution of known miRNAs in RCK, RT1, RT2 and RT3 libraries. (**C**) Distribution of known miRNA family members in *A. marina*.

In total, 27 known miRNAs representing 17 miRNA families were identified in *A. marina* (Figure 2A,B; Data S1). In RCK, 26 known miRNAs were found, while 25, 25, and 26 miRNAs were detected in RT1, RT2, and RT3, respectively, after Cr stress at different concentrations. Ama-miR164-1 was expressed only in RT3 and Ama-miR5138 was only present in RCK libraries. The lengths of the known miRNAs span a range from 18 to 24 nucleotides, with 21 nt accounting for the highest proportion (22, 81.48%), which is in full accordance with the length-distribution pattern of sRNAs (Figure 2C and Figure S3B). The diversity of the known (conserved) miRNA families in *A. marina* was determined by the number of members. For example, the two largest families, miR156 and miR396, contained 4 and 3 members, respectively. Certain miRNA families like miR164, miR166 and miR397 comprised two members per family; whereas those such as miR160, miR169 and miR7972 consisted of a single member each (Figure 2B).

### 2.4. Identification of Novel miRNAs in A. marina

**Figure 3:**
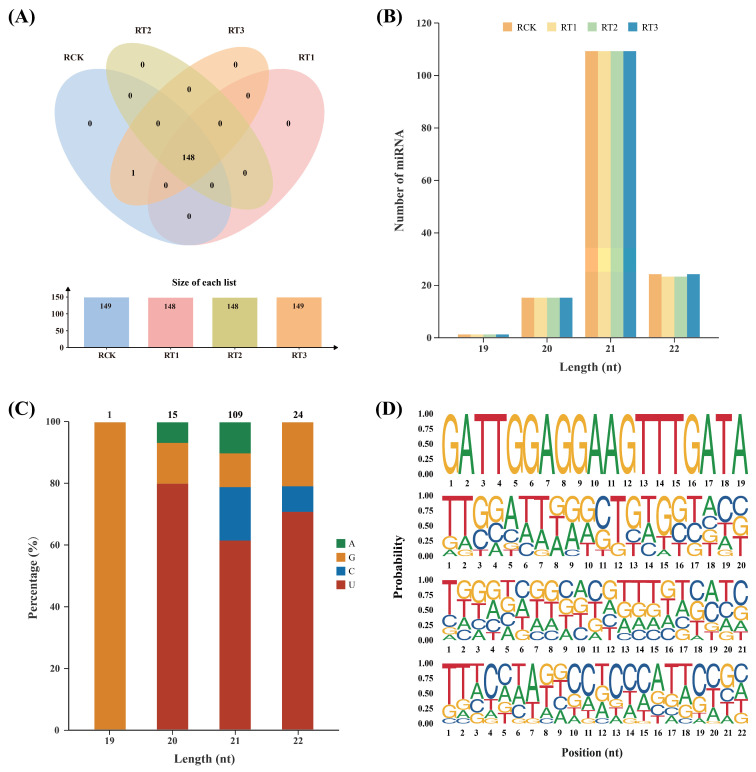
Identification of novel miRNAs in *A. marina*. (**A**) Distribution of novel miRNAs in the control and 200, 400 and 600 mg/L Cr stress groups. (**B**) Length distribution of novel miRNAs in *A. marina*. (**C**) First base preference analysis of newly predicted miRNAs in *A. marina*. (**D**) Base distribution at various positions of predicted miRNA.

Notably, more than 29.53% of sRNA sequences lacked annotation (Figure S3A); among these unannotated sequences, 149 novel miRNAs were identified with the help of miRDeep-P2 (v1.1.2) software [20] (Figure 3A; Data S2). These novel miRNAs ranged in length from 19 to 22 nt, which is consistent with the size of miRNA fragments generated by AGO-guided cleavage [21]. The length-distribution profile of the newly identified exhibited a high degree of consistency with that of sRNAs, with 21-nt miRNAs accounting for the largest share, at 73.15% (Figure 3B and Figure S3B). Ama-nmiR92-5p was only present in RCK and RT3 libraries, while the remaining 148 novel miRNAs were detected in RCK, RT1, RT2 and RT3. The abundance of these novel miRNAs was relatively low compared to the conserved miRNAs (Data S2), which is consistent with the general characteristics of species-specific miRNAs [22].

Digestion by Dicer-like (DCL) enzyme is generally believed to complete the generation of mature miRNAs. Due to the specificity of DCL restriction sites, the first base of miRNA sequences is biased towards uracil (U). Further, the tenth base is considered to be the cleavage point of miRNA-mediated interactions, which could be generally biased towards an adenine (A) [23]. The first base preference analysis of the newly discovered miRNA sequences in *A. marina* showed that uracil (U) was indeed the predominant first base, followed by guanine (G), while cytosine (C) and adenine (A) accounted for a relatively small proportion. Similarly, the 29.53% (highest proportion) of miRNAs contained A at the 10th base-position (Figure 3C,D). This analysis shows that the predicted miRNA sequences were consistent with the theory that the first base of miRNA/RISC complex tends to be a U [24]. Particularly, with the help of RNAfold web server (http://www.tbi.univie.ac.at/RNA/ (accessed on 13 May 2025)) [25], all the novel miRNA precursors were able to form the classical stem-loop structure and mature sequences were located on the arms of the hairpin structure, further confirming the authenticity of the novel miRNA (Figure S4).

### 2.5. Analysis of miRNA Accumulation Patterns

**Figure 4:**
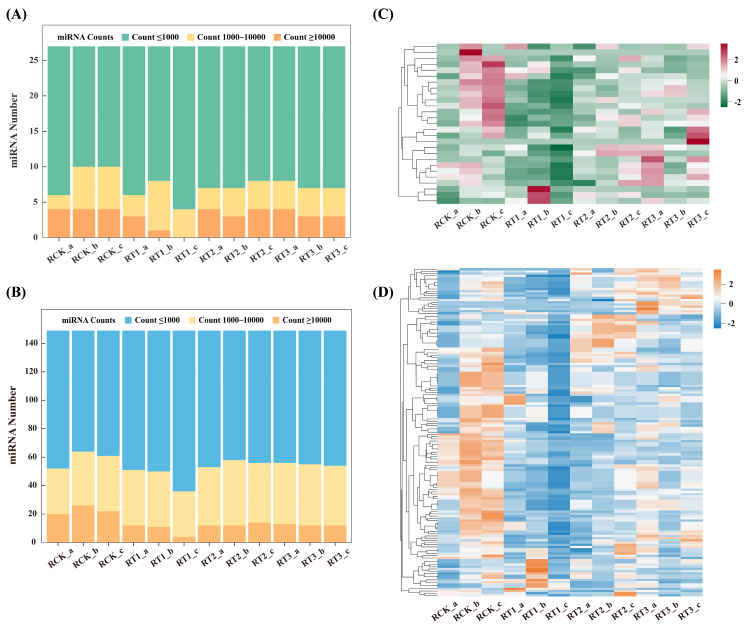
Analysis of miRNA abundances in *A. marina*. (**A**) Statistics of known miRNA abundances; (**B**) statistics of abundances of novel miRNAs; (**C**) clustering of known miRNA on the basis of their abundances; (**D**) clustering of novel miRNA on the basis of their abundances. Lines within C and D represent the hierarchical clustering results and relationships between samples. Closer branches indicate higher similarity in expression patterns, grouping similar samples into the same cluster.

In this study, miRNA abundances varied significantly across the 12 samples (Figure 4A,B; Datas S1 and S2). Among the known miRNAs, Ama-miR166a was the most abundant, with 38,745, 12,891, 23,645 and 35,464 reads accumulated in the RCK, RT1, RT2 and RT3 pools, respectively (Data S1). Among the newly predicted miRNAs, Ama-nmiR29-3p showed the highest abundance in the RCK, RT1, RT2 and RT3 samples with 140,157, 41,056, 52,908 and 80,823 reads, respectively (Data S2). Some miRNAs, such as Ama-miR156b, Ama-miR166b and Ama-nmiR144-3p, showed abnormally high abundances in the four sample groups, with their abundances reaching more than 5550 reads, while Ama-miR156-2, Ama-miR164-2, Ama-nmiR114-5p and Ama-nmiR136-3p showed moderate abundance levels, ranging from 519 to 8779. However, Ama-miR164, Ama-miR5138, Ama-nmiR24-5p, and Ama-nmiR92-5p were extremely low in abundance in all the sampled libraries, with their average levels being as low as 0.33 (Datas S1 and S2). The variation in miRNA abundances among different libraries (Figure 4C,D) indicated that different miRNAs may have biological functions during *A. marina* response to Cr stress.

### 2.6. Screening of miRNAs Differentially Expressed in Response to Chromium in A. marina

**Figure 5:**
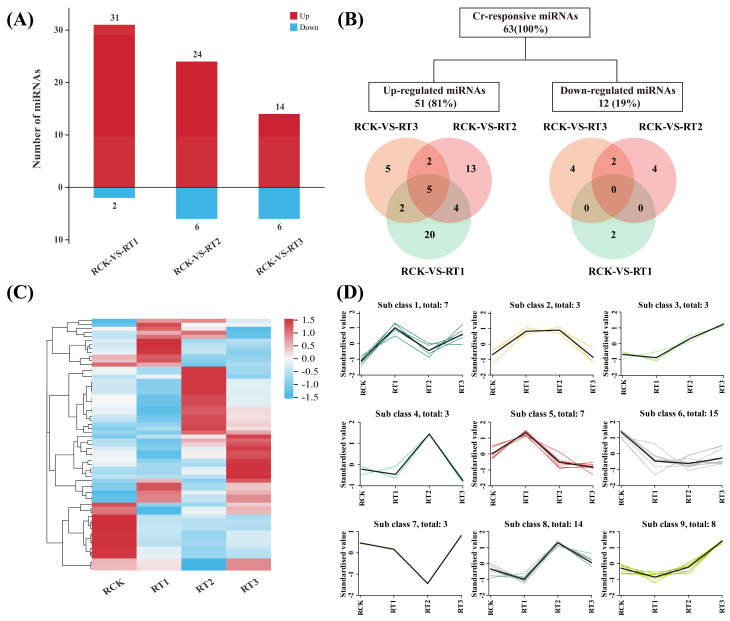
Analysis of miRNA expression in *A. marina* under Cr stress. (**A**) The number of up- and down-regulated miRNAs in *A. marina* after Cr stress; (**B**) Venn diagram shows the miRNA numbers before and after treatments with different concentrations of Cr; (**C**) heat map of all the miRNAs after Cr stress, drawn on the basis of their abundances; (**D**) differential miRNA expression trend analysis.

To further characterize the dynamic expression patterns of *A. marina* miRNAs in response to different concentrations of chromium (Cr) exposure, differential expression analysis was performed between the untreated control group (RCK) and the Cr-stressed groups (RT1, RT2, and RT3). A total of 63 miRNAs were identified as significantly differentially accumulated under Cr stress, including 6 known miRNAs and 57 novel miRNAs (q-value < 0.001). Among these, 51 miRNAs were up- and 12 were down-regulated (Figure 5A; Table S2). Specifically, in the RCK vs. RT1 comparison, 31 miRNAs were up- and 2 were down-regulated, with a maximum absolute log_2_ fold change of 2.61 and a minimum of 1.0. In the RCK vs. RT2 comparison, 24 miRNAs were found to be up-regulated, whereas 6 miRNAs were down-regulated. The maximum |log_2_FC| was 2.43 for up-regulated miRNAs and 1.80 for down-regulated miRNAs. Similarly, in the RCK vs. RT3 comparison, 14 and 6 miRNAs exhibited increased and decreased accumulations, with the maximum |log_2_FC| of 4.90 and 1.52, respectively (Figure 5B; Table S2). Overall, the number of upregulated differentially expressed miRNAs (DE-miRNAs) was greater than that of the downregulated, indicating that most miRNAs exhibited increased accumulation upon treatment with Cr. Moreover, the number of DE-miRNAs gradually decreased with the elevation of Cr stress (Figure 5A–C; Table S2). Notably, among these DE-miRNAs, Ama-miR408, Ama-nmiR16-5p, Ama-nmiR50-5p, Ama-nmiR81-5p, and Ama-nmiR113-3p were consistently up-regulated across all three comparison groups (Table S2).

The accumulation pattern of 63 differential miRNAs was further analyzed, and 9 enriched miRNA-modules were obtained (Figure 5D). The sub-class 6 contained the largest numbers (15) of enriched miRNAs, which included 1 known and 14 novel miRNAs. Amongst these, Ama-miR160 was continuously down-regulated. Then, sub-class 8 comprised the second most enriched module of 14 miRNAs. The accumulation of these miRNAs followed the pattern of RT1 < RCK < RT3 < RT2. Among all the differential miRNAs, only Ama-nmiR8-3p was enriched in the sub-class 3, which showed a sustained up-regulation (Figure 5D). These results indicate that Cr stress response in *A. marina* is not regulated by a single miRNA. These differences in miRNA abundance changes indicate they may regulate the expression level of their target genes in a way that helps the physiological state of the plants to adapt to various degrees of stress.

### 2.7. Prediction and Functional Annotation of Differential miRNA Target Genes, and RT-qPCR Verification

**Figure 6:**
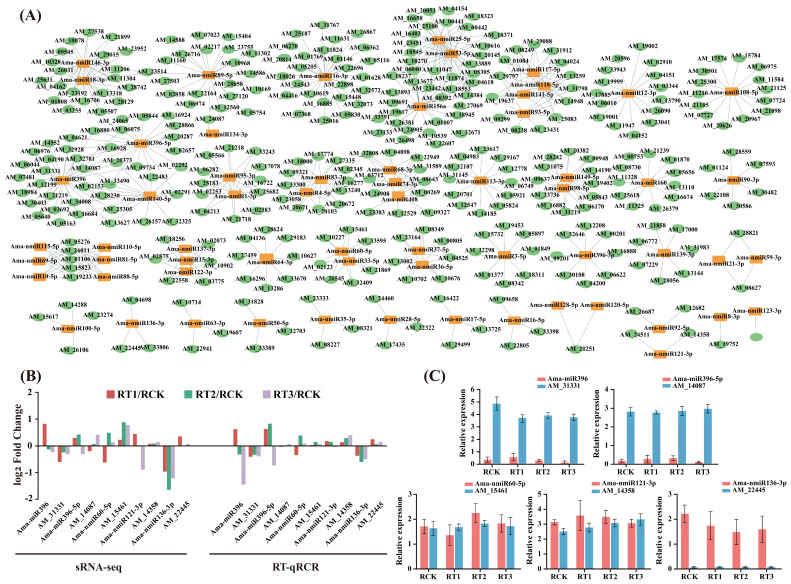
Analysis of miRNA-target gene network and RT-qPCR validation under Cr stress. (**A**) miRNA-target gene interaction network. Orange nodes represent the miRNAs, whereas green nodes represent their target genes. miRNAs and targets are marked by yellow squares and green ovals, respectively; lines link miRNA and its respective targets. (**B**) Expression changes in miRNAs and target genes after Cr stress were obtained by real-time quantitative polymerase chain reaction (RT-qPCR) and sRNA sequencing, respectively. Five miRNAs and their target genes were randomly selected from the expression network for RT-qPCR validation. (**C**) Expression levels of 5 miRNA-target genes obtained by RT-qPCR in control (RCK) and Cr-treated (RT1, RT2 and RT3) groups.

Identifying target genes of miRNAs could be helpful in elucidating their biological functions. This study employed psRobot [26] and TargetFinder [27] software for miRNA target prediction, and the miRNA-target relationships supported by both tools were selected as the final results. Predictions for the 63 DE-miRNAs yielded a total of 355 targets, which formed 571 regulatory pairs. Among these, known and novel miRNAs formed 103 and 468 regulatory interactions, respectively (Figure 6A; Data S3). This analysis also showed that most miRNAs could target multiple genes, and a single target gene might be regulated by multiple miRNAs. For example, Ama-miR156a could regulate 28 target genes, including AM_26381, AM_24905, and AM_18945, whereas the target gene AM_01106 could be co-regulated by 6 miRNAs, which include, for example, Ama-nmiR10-5p, Ama-nmiR69-5p, and Ama-nmiR81-5p. Targets for one miRNA (Ama-nmiR124-3p) were not predicted, which could be due to methodological limitations [28].

To explore the regulatory pathways where the DE-miRNAs could play a role in Cr stress response, functional annotation analysis was further performed on the predicted target genes. The target genes primarily encode for proteins related to primary metabolism, transcriptional regulation, signal transduction, stress response, ion transport, and other processes (Data S3). It is inferred that *A. marina* miRNAs could be broadly involved in regulating biological processes such as nuclear formation, membrane composition, DNA binding, enzyme activity regulation, transcription factor modulation, substance binding, and the synthesis of various functional proteins and enzymes (Data S3). Further analysis showed that 25 genes (target of 25 miRNAs) exhibited significant changes in their expressions after exposure to Cr stress (Table S3; Figure S5; Data S4). These findings indicate that miRNAs play important regulatory roles in the Cr stress response of *A. marina*. The miRNA-mRNA interaction network also provides valuable clues for investigating the complex regulatory mechanisms underlying Cr stress response in *A. marina*.

Five miRNA-mRNA pairs were randomly selected from the predicted interaction network for further validation using RT-qPCR. The results demonstrated consistency between the RT-qPCR data and the high-throughput sequencing results (Figure 6B,C). For instance, both detection methods indicated that the accumulation levels of Ama-miR396 and Ama-miR396-5p had remained consistently low across all the samples, while their target genes AM_31331 (*TM9SF1*) and AM_14087 (*WRKY1*) maintained relatively high expression levels in all treatment groups. Additionally, Ama-nmiR121-3p exhibited increased expression under RT1 and RT2 treatments but decreased under RT3 treatment, whereas its target gene AM_14358 (*WRKY33*) showed an upregulated expression under Cr stress (Figure 6C). These findings further confirm the reproducibility and reliability of the high-throughput sequencing data and suggest that *A. marina* may adapt to stress conditions by modulating miRNA expression.

### 2.8. GO and KEGG Analysis of Differential miRNA Target Genes

**Figure 7:**
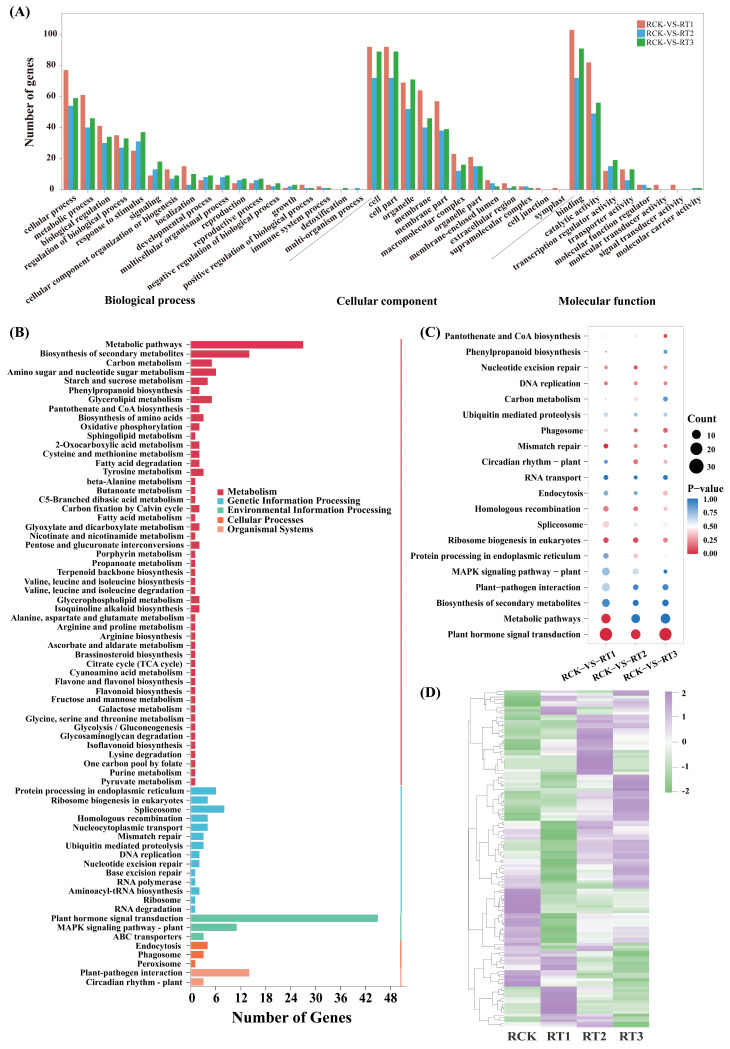
GO annotation and KEGG enrichment of miRNA target gene in *A. marina* roots after Cr treatment. (**A**) GO annotations of predicted target genes of the miRNA. (**B**) KEGG pathway annotations of predicted target genes of the miRNA. The three major categories of biological processes, cellular components and molecular functions are divided by black slant lines. (**C**) The 20 KEGG pathways with the largest number and their enrichment statistics. The vertical and the horizontal axes represent the KO entry names and the enrichment factors of the corresponding pathways, respectively. (**D**) Cluster analysis of expression of target genes of top 20 KEGG pathways (with the largest number of enriched genes).

To further explore the functions of genes responsive to Cr treatment in *A. marina*, Gene Ontology (GO) annotation and KEGG enrichment were performed on the target genes of differentially accumulating miRNAs. A total of 287 target genes were annotated to 38 GO terms (Figure 7A; Data S5), which are classified into three major categories of biological processes (50), cellular components (32), and molecular functions (19). In the category of biological processes, the predominantly enriched classes were cellular process (GO: 0009987), metabolic process (GO: 0008152), and biological regulation (GO: 0065007). This indicates that for mitigating the stress-induced damage, the roots of *A. marina* could have modulated its gene expression, activated stress-responsive pathways, adjusted metabolic routes, and regulated substance transport and organelle functions within the cells. In the context of cellular components, the enriched classes included cell (GO: 0005623), cell part (GO: 0044464), and organelle (GO: 0043226). These genes are closely associated with the structure and function of cellular components, implying the potential role of miRNAs in the perception and response to external signals under Cr stress. Similarly, molecular functions were primarily enriched in processes such as binding (GO: 0005488), catalytic activity (GO: 0003824), and transcription regulator activity (GO: 0140110). The proteins encoded by these target genes possess functions such as catalyzing biochemical reactions, participating in redox processes, and transporting substances, which collectively contribute to the regulation of Cr stress adaptation.

Through KEGG enrichment analysis, a total of 141 target genes corresponding to 71 pathways were enriched, with the majority associated with metabolism (Figure 7B; Datas S6 and S7). Among these, the pathways with the highest enrichment included metabolic pathways (ko01100; 27 genes), plant hormone signal transduction (ko04075; 45 genes), biosynthesis of secondary metabolites (ko01110; 14 genes), plant-pathogen interaction (ko04626; 14 genes), and MAPK signaling pathway (ko04016; 11 genes) (Figure 7B). Within the “metabolism” category, the most representative pathways were related to the phenylpropanoid biosynthesis (ko00940). In the “environmental information processing” category, three signaling pathways were identified: plant hormone signal transduction (ko04075), MAPK signaling pathway in plants (ko04016), and ABC transporters (ko02010). These results indicated an important role of the target genes in Cr stress resistance. Clustering analysis was performed on the target genes participating in the top 20 KEGG pathways. The results revealed significant changes in the expression levels of these target genes following treatments with different concentrations of Cr (Figure 7C,D; Datas S4, S6 and S7), implying that these genes may play a role in regulating the metal tolerance of *A. marina*. These pathways represent the most likely candidates that could explain the mechanisms involved in the Cr stress response of *A. marina*.

### 2.9. Identification of miRNA Key Regulatory Pathways in Response to Cr Stress in A. marina

**Figure 8:**
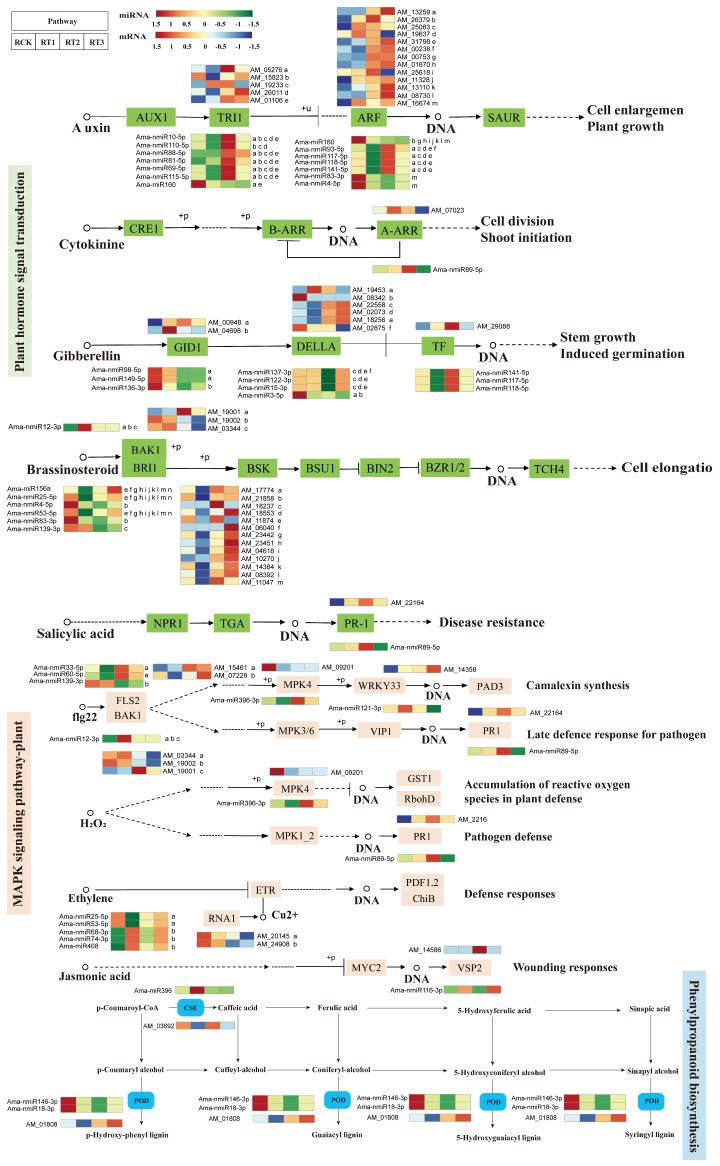
Key pathways of *A. marina* that responded to Cr stress. In the figure, letters like “a, b, c, d” in the figure represent corresponding numbering identifiers for different target genes.

**Figure 9:**
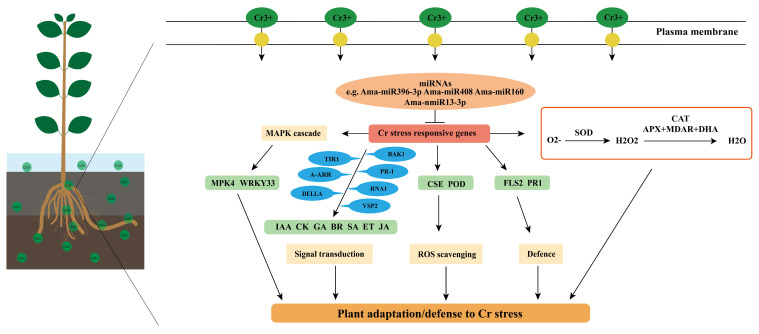
Regulation model of Cr stress response in *A. marina*.

The following three key pathways, plant hormone signal transduction (ko04075), MAPK signaling (ko04016), and phenylpropanoid biosynthesis (ko00940), were selected for a detailed analysis during *A. marina*’s response to Cr stress (Figure 8 and Figure 9). Among the genes of plant hormone signal transduction pathway, a total of 45 were identified as potentially involved in regulating Cr stress response in *A. marina* (Table S4). Target genes coding for the auxin receptor TIR1 displayed distinct expression patterns during Cr treatment. For instance, AM_01106 was up-regulated under all the Cr concentrations, whereas AM_05276 and AM_26011 were down--regulated under RT1 but up-regulated under RT2 and RT3. *Auxin response factor* (*ARFs*) genes, AM_26379, AM_25083, and AM_31798, potentially regulated by Ama-miR160 and Ama-nmiR9-5p, were up-regulated after Cr stress. In contrast, AM_19637 and AM_25618 were down-regulated under RT3. In the gibberellin signaling pathway, Ama-nmiR98-5p and Ama-nmiR149-5p co-target AM_00948, which is annotated as a gibberellin receptor, GID1. The expression of AM_00948 was elevated under RT1, RT2, and RT3 compared to the RCK control. In gibberellin signaling, GID1 typically binds to gibberellin to form a complex that interacts with DELLA proteins [29]. In this study, the expression levels of downstream DELLA proteins (such as AM_02073, AM_02875, and AM_08342) and a transcription factor TF (AM_29088) fluctuated synchronously with GID1, indicating their coordinated involvement in regulating gibberellin signal transduction. Next, brassinosteroid signaling was investigated, where the *brassinosteroid insensitive 1 receptor* (*BRI1*) genes (AM_04618, AM_06040, AM_08392) were down-regulated under RT1 but were up-regulated under RT2 and RT3 (following Cr stress). The genes encoding the receptor kinase BAK1 (AM_19002 and AM_03344), and potentially targeted by Ama-nmiR12-3p, did not show significant expression changes between RCK and RT1 but were down-regulated under RT2 and RT3 after Cr exposure.

When we examined the MAPK signaling pathway (Figure 8; Table S4), the *flagellin receptor kinase FLS2* gene (AM_15461), potentially targeted by Ama-nmiR33-5p and Ama-nmiR60-5p, was up-regulated post-Cr stress. Conversely, the expression of mitogen-activated protein kinase *MPK4* (AM_09201) was observed to be decreased, which may lead to the accumulation of reactive oxygen species (ROS). Corresponding to these changes, the expression levels of downstream genes in the MAPK cascade, the transcription factor *WRKY33* (AM_14358) and the *PR1 protein* gene (AM_22164), were up-regulated, indicating certain correlations among the expression patterns of these genes. The gene AM_14586, a vegetative storage protein VSP2 and a predicted target of Ama-nmiR116-3p showed higher expression under RT1 than in the other three groups. Within the phenylpropanoid biosynthesis pathway (Figure 8; Table S4), the expression level of AM_03692, a caffeoyl shikimate esterase and potentially regulated by Ama-miR396, increased under RT2 compared to the control but decreased under RT1 and RT3. The *peroxidase* (*POD*) gene AM_01808, potentially co-regulated by Ama-nmiR18-3p and Ama-nmiR146-3p, showed clear differences among treatment groups: its expression was slightly lower than the control under RT1 but exhibited a gradual increasing trend under RT2 and RT3.

## 3. Discussion

### 3.1. Sequencing of sRNA and miRNA Identification of A. marina

Heavy metal pollution is an important factor affecting plant growth, but the mechanism for plants to overcome this stress is very complex [30]. In order to cope with heavy metal stress, plants regulate a large number of genes at transcriptional and post-transcriptional levels [31]. miRNAs, as universal regulators of gene expression, have been confirmed to play a key role in plant stress response to heavy metals by modulating gene expression regulatory network [32]. *A. marina*, as a pioneer mangrove species, plays an important role in maintaining the ecological balance of coastal zones. To date, miRNA-mediated regulatory responses to heavy metal stress have been well-documented across model and crop species [32]. However, very few mangrove plants, specifically *Kandelia candel* [33], *Bruguiera gymnorrhiza* [34] and *Sonneratia apetala* [35], have been investigated in the context of stress resistance-related miRNA, while the research on Cr stress-regulated miRNA and its target gene in *A. marina* is still lacking. In this study, 12 sample banks of *A. marina* from the control group and the Cr stress treatment group were analyzed by sRNA sequencing technology, and about 361,751,395 reads were obtained. After filtering, 313,960,410 clean reads were obtained, laying a foundation for subsequent miRNA identification analysis (Table 1). The sRNA of *A*. *marina* includes various types, such as rRNA, miRNA, snoRNA, tRNA and other types of RNA (Figure S3A). Many sRNA sequencing data have not been successfully annotated, indicating that there is still a large amount of sRNA to be identified in *A. marina*. During annotation of *A. marina* miRNAs, it was apparent that sequences of 21 nt lengths were dominant (Figure 2C and Figure 4B), which is similar to reports in cotton [36], *Ipomoea batatas* L. [21], and other mangrove species such as *Kandelia* [33] and *Bruguiera* [34].

The diversity of *A. marina* miRNAs was also reflected in the different changes in miRNA expression under different concentrations of chromium stress. It is worth noting that known conserved miRNAs generally accumulate at higher levels than newly discovered miRNAs (Datas S1 and S2). The general nature of this plant miRNA has been supported by previous studies [22]. Notably, several novel miRNAs exhibited higher accumulation levels than the conserved known miRNAs in this study. This observation may be attributed to the current limitations in genomic and transcriptomic resources for *A. marina*, a non-model mangrove species. The incomplete annotation of small RNA datasets likely results in a substantial portion of sRNA sequences remaining uncharacterized, leaving many species-specific miRNAs yet to be identified and catalogued. Consequently, the apparent higher abundance of novel miRNAs may reflect, at least in part, the underrepresentation of known miRNA annotations rather than an absolute biological predominance. The expression pattern of miRNAs also showed significant changes under different concentrations of Cr. For example, the accumulation levels of Ama-nmiR12-3p in cells increased with Cr concentration. Its predicted target (AM_03344) showed a negative correlation in expression. Nonetheless, the miRNAs with positive correlation with their predicted target genes were also identified in this study, possibly due to the possibility that a potential target gene could be regulated by multiple miRNAs, which shows a complex regulatory relationship between the miRNAs and their target genes. Nevertheless, this study lays a solid foundation for identification of miRNAs involved in Cr stress response in *A. marina*.

### 3.2. Characterization of miRNAs and Target Genes in Response to Chromium Stress in A. marina

The adaptation of plants to heavy metals is a long-term and dynamic process involving various changes at morphological, physiological, molecular, and cellular levels [37]. Identifying differentially expressed miRNAs and analyzing their functions can help to reveal plant response mechanisms under Cr stress. For instance, in radish plants, studies have shown that 54 conserved miRNAs and 16 novel miRNAs change their accumulation significantly under Cr stress [13]. Exposure of tobacco (*N. tabacum*) roots to Cr stress revealed that 29 conserved miRNAs and 14 novel miRNAs were differentially expressed [14]. In *Miscanthus*, high-throughput miRNA sequencing identified 104 conserved miRNAs and 158 non-conserved miRNAs that showed preferential accumulation under Cr stress [38]. In the present study, a total of 63 miRNAs, including 6 known miRNAs and 57 novel miRNAs, showed significant differential expression under Cr stress (q-value < 0.001; Figure 6, Table S2). In addition, this study also identified miRNAs such as miR156, miR164, miR166, miR167, miR169, miR397, and miR398, which have been demonstrated to play important roles in plant responses to Cr stress [12,14]. These results indicate the existence of common Cr stress response pathways across different species and that these miRNAs may target shared genes in Cr responses among various plants. Moreover, we observed that some stress-regulated miRNAs may play fine-tuning roles in plant adaptive responses to different stresses. For example, miR169 acts as a bridging molecule linking plant responses to abscisic acid (ABA), drought, and metal stress [39]; miR398 has been confirmed to play critical roles under multiple stresses, including water deficit, oxidative stress, and salt stress [40]. This may be attributed to the ability of a single miRNA to target multiple genes and the existence of shared response pathways in plants under various adverse conditions. Previous studies on different plant species have shown that known miRNA families are generally down-regulated under heavy metal stress [41]. In this study, miR156 also exhibited a downregulation under Cr stress, implying that complex miRNA-mediated gene regulation mechanisms are involved in plant responses to metal stress.

The identification and functional analysis of miRNA-target genes is a key step in elucidating miRNA regulatory mechanisms in plant response to heavy metal stress [42]. In this study, a total of 355 target genes and 571 miRNA-target gene regulatory pairs were predicted to be involved in regulating Cr-stress response (Figure 6A; Table S2; Data S3). Plant responses to Cr-stress are coupled to a range of intracellular processes, including signal sensing and transduction, transcriptional reprogramming, regulation of protein biosynthesis, and ultimately the physiological changes in response to the stress. GO enrichment analysis revealed that predicted target genes are involved in many biological processes associated with stress, such as stimulus response, organelle, and active catalysis (Figure 7A). Further, the KEGG pathway analysis indicated that miRNA could mainly regulate plant resistance response to chromium (Cr) stress by participating in plant hormone signal transduction, MAPK signal pathway, phenylpropane biosynthesis and other pathways (Figure 7B–D). In addition, under Cr stress, ABC transporter, splice body, protein processing and other pathways may activate Cr-induced signal molecules, ion channels, ROS and other stress-related metabolites by activating various enzymes [43]. Functional analysis of miRNA target genes indicated that these genes were widely involved in a variety of biological processes, such as metal ion binding, DNA binding, transcription regulation, signal transduction, redox homeostasis, membrane structure stability and material metabolism, and heavy metal absorption and detoxification (Data S3, Table S4). Some of these target genes have functions of encoding transcription factor family members like ARF and WRKY. These transcription factors have been shown to activate stress-related genes [44]. In addition, some target genes, such as AM_01106, AM_29088 and AM_03344, have probable functions of antioxidant enzymes, heavy metal transporters and stress-related transcription factors, which participate in plant tolerance and response to Cr stress by mediating active oxygen scavenging, Cr-ion transmembrane transport and intracellular chelation, and regulating the expression of key genes in stress resistance pathway (Table S4). Genes like AM_16888, AM_19001 and AM_07023 that are potential targets of Ama-miR396-3p, Ama-nmiR12-3p and Ama-nmiR89-5p are involved in multiple tolerance responses, including for Cr (Data S3, Table S4). AM_18371, a predicted target of Ama-nmiR53-5p, is rich in DNA-binding domains. It is speculated that *AM_18371* could resist DNA damage induced by Cr stress and strand breaks induced by oxidative stress by targeting DNA repair enzymes and histone modification-related proteins [45]. Thus, it may help to maintain chromatin structural stability and genetic information integrity. AM_17774, the predicted target of Ama-nmiR83-3p, is annotated as a calcium-dependent protein kinase (CDPK) and other specific signaling components [46]. The regulation of such genes may enhance the signal transduction efficiency of chromium stress and enhance the activation of downstream defense responses by inhibiting the expression of negative regulators of the pathway.

### 3.3. Analysis of miRNA-Mediated Key Regulatory Pathways in Response to Cr Stress in A. marina

Although a large number of stress-related miRNAs are evolutionarily conserved in plants, the regulatory patterns of some miRNAs vary among different species [47]. Combined with the functional annotations of target genes, miRNAs involved in the three pathways of plant hormone signal transduction (ko04075), MAPK signaling (ko04016) and phenylpropane biosynthesis (ko00940) were analyzed (Figure 8 and Figure 9). Signal transduction is an important process that transforms upstream signals into downstream complex reactions [48] and forms the core component of a plant’s heavy metal stress response regulatory network. In this study, Ama-nmiR10-5p and Ama-miR160 were annotated to be a part of the auxin signaling pathway, and showed specific expression characteristics under Cr stress. Based on the correlation between the expression changes in Ama-nmiR10-5p and Ama-miR160 and their target genes, it is inferred that they may be involved in auxin signaling response to Cr stress by regulating *TRI1* gene expression and ARF-mediated *SAUR* gene expression. Further, there have been many reports that miRNAs participate in stress response by regulating GA signaling [49]. Herein, we found that the expression of Ama-nmiR98-5p, Ama-nmiR149-5p and Ama-nmiR136-3p was down-regulated under different concentrations of Cr stress compared with RCK, and the expression of their predicted target gene, *GID1*, changed accordingly. Therefore, we hypothesize that these three miRNAs could co-regulate *GID1* expression, which may further mediate the binding process between GA and GID1, and then trigger the ubiquitination degradation of DELLA protein and release its inhibition of transcription factors [50]. Similarly, brassinosteroids (BRs) are an important hormone that regulates plant growth and development. Basit et al. [51] found that the interaction between BRI1 and BAK1 is the key signal initiation link for BR to exert its heavy metal stress resistance function. In this study, the expression level of Ama-nmiR12-3p was up-regulated during Cr-stress compared with that of the control group (RCK), where its abundance was the highest in the RT1 treatment group, while its potential target, BAK1, showed variable expression characteristics. On the contrary, the expression level of Ama-nmiR25-5p was the highest in the control group (RCK), and it was down-regulated throughout the Cr-stress conditions. The expression level of its target gene, BRI1, decreased to the lowest level in RT1 treatment group, while it increased in RT2 and RT3 treatment groups. Therefore, we speculate that after the targeted regulation of *BAK1* by Ama-nmiR12-3p and *BRI1* by Ama-nmiR25-5p, they interact with each other, which thus results in fluctuation in the trend of expression under different concentrations of Cr stress. It may affect BRs’ signal transduction and trigger the BR-mediated downstream pathway, and finally may change the Cr resistance of *A. marina* (Figure 8 and Figure 9).

MAPKs, as one of the highly conserved signaling molecules in eukaryotes, are responsible for coordinating cellular responses to maintain normal plant growth and development [52]. When plants are subjected to heavy metal stress, they activate multiple signaling pathways, including MAPK cascade reactions. In the roots of *Broussonetia papyrifera*, MAPK-related genes were down-regulated at 3 h and up-regulated at 6 h with the extension of Cd stress time [53]. Herein, a large number of novel miRNAs have been identified that may play key roles in Cr stress response in *A. marina*. In the KEGG annotation of this study, the genes associated with the MAPK pathway, such as *WRKY33* (AM_14358), *PR1* (AM_22164), and *FLS2* (AM_15461), are predicted as targets of the novel miRNAs, Ama-nmiR121-3p, Ama-nmiR89-5p, Ama-nmiR33-5p, and Ama-nmiR60-5p (Data S6). We noticed that the conserved and novel miRNAs could often synergistically be involved in the regulation of key pathways in Cr stress response in *A. marina*. In this study, the conserved Ama-miR396-3p was predicted to target AM_09201 (encoding MPK4 protein). MEKK1-MKK4/5-MPK3/6 and MEKK1-MKK2-MPK4/6 are located downstream of ROS, and play a role in abiotic and biotic stress responses [54]. Further, an AM_14358 (coding a WRKY33 protein) could be targeted by a novel miRNA, Ama-nmiR121-3p. This result indicates that the conserved and the novel miRNA could regulate the levels of ROS and H_2_O_2_ through synergistic regulation of the expression of their downstream target genes, during the response of *A. marina* to Cr stress (Figure 8 and Figure 9). The phenylpropane biosynthetic pathway plays an important role in regulating plant growth and development and stress response [55]. CSE is a key enzyme in lignin synthesis in *A*. *thaliana* [56]. Lignin, as one of the main components of the plant cell secondary wall, which plays an important role in heavy metal ion fixation. Under Cd stress, lignin fixes Cd^2+^ to the cell walls through phenolic hydroxyl, carboxyl and other functional groups, thus reducing the ROS burst caused by Cd^2+^ entry into the cytoplasm of rice [57]. In this study, the expressions of AM_01808 (annotated as a CSE homolog) and AM_01808 (POD) were changed under different Cr treatment conditions, among which AM_03692 could be regulated by Ama-miR396, and AM_01808 by Ama-nmiR18-3p and Ama-nmiR146-3p. Changes in the expression of Ama-miR396 under Cr stress may induce a decrease in *CSE* gene expression in *A. marina* at RT1 and an increase in expression at RT2 and RT3, which in turn would affect the decrease and then increase in root lignin content. The initial decrease in lignin content may play a defensive role in mitigating Cr-induced ROS toxicity, but with the increase in Cr concentration, lignin content in roots might increase and ROS accumulation might decrease accordingly. This phenomenon would indicate that the defense ability of the phenylpropanoid biosynthesis pathway in roots to high Cr concentration stress is enhanced, and this process may be related to the up-regulation of POD-related genes. Additionally, POD plays a key role in phenylpropanoid biosynthesis [58]. Combined with the characteristics of expression changes in this gene, it is speculated that its up-regulation may have a positive effect on lignin synthesis [59]. This may indicate that lignin synthesis in roots is an important feature of *A. marina* in response to Cr stress. In conclusion, a large number of miRNAs responding to Cr stress were identified in this study, indicating that there is a complex regulatory network for heavy metal response in *A. marina*.

## 4. Materials and Methods

### 4.1. Chromium Stress Treatment and RNA Extraction

*A. marina* seeds were harvested from a mangrove coastal belt in Techeng Island (21°09′~21°10′ N, 110°25′~110°27′ E) in Guangdong, China. The collected seeds of *A. marina* were sown in artificially prepared seedling bags containing nutrient soil. After about 90 days of growth, seedlings with a height of 10–16 cm were transplanted to the seedling pots. The experiment was conducted in a naturally lit and ventilated greenhouse, without artificial light supplementation or temperature/humidity control. To ensure optimal ion uptake, plants were cultivated in a weakly acidic nutrient substrate (pH 6.0 ≈ 0.1) followed the local natural climatic conditions. Six-month-old seedlings of *A. marina*, with comparable consistent growth, were selected and set into the control group and the Cr stress treatment groups, respectively. Preliminary dose–response assays revealed that *A. marina* plants treated with 100–200 mg/L Cr^3+^ exhibited no significant phenotypic inhibition, whereas distinct physiological stress was observed at 400 mg/L, and severe toxicity symptoms appeared at 600 mg/L (Figure 1A; Table S1). Thus, three representative concentrations—200 mg/L, 400 mg/L, and 600 mg/L—were selected for subsequent sRNA sequencing to capture the full spectrum of molecular events from adaptive regulation to toxic damage. CrCl_3_ solution was applied to the three Cr stress treatment groups (RT1-RT3) at 3-day intervals over a 14-day experimental period, while the control group (RCK) received an equivalent volume of deionized water following the same irrigation schedule. Root tissues of the control and treated groups were collected and washed three times with deionized water. All the samples collected were frozen in liquid nitrogen and then transferred to a −80 °C refrigerator for subsequent RNA extraction.

Total RNA from each sample was extracted with the help of the RNeasy Plant mini kit (Qiagen, Hilden, Germany) according to the manufacturer’s instructions. RNA quality was checked using an Agilent 2100 Bioanalyzer (Agilent Technologies, Santa Clara, CA, USA) and the results were presented in Figure S6. RNA samples with RNA integrity number (RIN) greater than 7.0 and 28S/18S ratios of 1.8–2.2 were used for further analysis.

### 4.2. Growth Parameters and Chromium Content Measurements

After 14 days of Cr treatment, plant height (from stem base to apical bud) and root length (length of the longest root) were measured using a vernier caliper. For biomass determination, roots and shoots were separated, oven-dried at 70 °C for 72 h to a constant weight, and then weighed to determine dry biomass. Root dry biomass was calculated by subtracting shoot dry biomass from the total dry biomass of each seedling.

For Cr content measurement, shoot and root samples from control and Cr-treated groups were harvested separately. Samples were rinsed with running tap water to remove adhering soil particles, followed by three rinses with deionized water. Cleaned samples were oven-dried at 105 °C for 30 min and then at 70 °C to constant weight. Approximately 0.2 g of dried sample was ground and passed through a 2 mm sieve. The powder was transferred to a digestion tube, moistened with a small volume of deionized water, and then a mixed acid of HNO_3_ and HClO_4_ (4:1, *v*/*v*) was added. The mixture was allowed to stand overnight. Subsequently, the digestion tubes were placed on a hot plate and heated at 200 °C until the solution became clear and transparent and white fumes subsided. After cooling, the digest was transferred to a 50 mL volumetric flask and brought to volume with deionized water. The solution was filtered through quantitative filter paper, and the total Cr concentration was determined using an Atomic Absorption Spectrophotometer (Analytik Jena AG, Jena, Germany) following standard procedures. All reagents used were of guaranteed reagent (GR) grade. As the plants were cultivated in soil medium and no speciation pretreatment was performed, the AAS results strictly represent the total Cr concentration measured, which is assumed to primarily represent Cr (III) accumulation. Results are expressed as total Cr content per gram dry weight (µg/g DW).

### 4.3. TBARS Content, Antioxidant Enzyme Activity and Chlorophyll Level Determination

TBARS content was determined by the thiobarbituric acid (TBA) colorimetric method described by Velikova et al. [60]. Briefly, 0.5 g of fresh root tissue was homogenized in 5 mL of 0.1% (*w*/*v*) trichloroacetic acid (TCA) and centrifuged at 10,000× *g* for 10 min. The supernatant was mixed with 0.5% (*w*/*v*) TBA in 20% TCA, heated at 95 °C for 30 min, and then quickly cooled on ice. Absorbance was measured at 450, 532, and 600 nm. For antioxidant enzyme assays, 0.5 g of fresh root tissue was homogenized in 5 mL of ice-cold 50 mM phosphate buffer (pH 7.8) containing 1% polyvinylpyrrolidone (PVP) and 0.1 mM ethylenediaminetetraacetic acid (EDTA). The homogenate was centrifuged at 12,000× *g* for 20 min at 4 °C, and the resulting supernatant was collected as the crude enzyme extract for analysis. SOD activity was measured spectrophotometrically following the method of Tian et al. [61]. One unit of SOD activity was defined as the amount of enzyme required to cause 50% inhibition of NBT reduction at 560 nm. CAT activity was determined using the method of Kato and Shimizu [62]. The reaction mixture contained 50 mM phosphate buffer (pH 7.0) and 10 mM H_2_O_2_, and the decrease in absorbance at 240 nm was measured for 2 min. POD activity was measured using a modified method of Rao et al. [63]. The reaction mixture contained 50 mM phosphate buffer (pH 7.0), 20 mM guaiacol, and 10 mM H_2_O_2_. The increase in absorbance at 470 nm was recorded for 2 min. Chlorophyll content was determined following the method by Lichtenthaler and Wellburn [64]. Briefly, approximately 0.5 g of fresh leaf tissue was homogenized in 10 mL of 95% (*v*/*v*) ethanol and extracted in the dark at 4 °C for 48 h until the tissue became completely white. Then the absorbance of the supernatant was measured at 645 nm and 663 nm using a UV-Vis spectrophotometer. Chlorophyll a (Chl a), chlorophyll b (Chl b), and total chlorophyll (Chl a+b) contents were expressed as mg/g fresh weight (FW).

### 4.4. Construction of sRNA Library and Bioinformatics Analysis of miRNA

The method for constructing and sequencing the sRNA library comprises the following steps: (1) extracting total RNA from root tissues of *A. marina*; (2) purifying and separating RNA of 18–30 nt by PAGE electrophoresis; (3) connecting low molecular weight RNA with 5′ and 3′ sequencing adapters, and purifying the connected products; (4) carrying out PCR amplification on sRNA with 5′and 3′ junction sequences; (5) recovering and purifying the PCR products; (6) connecting, reverse transcribing and constructing cDNA libraries; (7) carrying out sequencing by utilizing BGISEQ-500 sequencing platform and SE50 bp sequencing type.

The raw sequencing data were filtered using SOAPnuke1.5.0 software [65] to remove the reads with low sequencing quality, 5′ linker contamination, no 3′ linker sequences, no inserts, polyA and sequences less than 18 nt. The resulting ‘CleanReads’ were aligned with the *A. marina* genome sequence with the help of Bowtie software (v1.1.2) [19]. The annotation of non-coding RNA was based on CleanReads and miRBases V22 (http://www.mirbase.org/), RNAcentral V16.0 (https://rnacentral.org/) and Rfam V13.0 (https://rfam.xfam.org/) databases. First, all CleanReads of the sample were aligned with the non-coding RNA sequence information recorded in the miRBase and RNAcentral databases. The rest of the Reads that did not match miRBase and RNAcentral were aligned to the Rfam database for homologous non-coding RNA annotation by using the homology alignment methods such as cmsearch [66]. The unannotated sequences were predicted by miRDeep-P2 software (v1.1.2) [20].

### 4.5. Differentially Accumulated miRNAs Identification, Target Gene Prediction and Function Analysis

The sequence data were aligned with the known or the newly predicted miRNA precursor sequences, by allowing 0 mismatches, and the counts of the statistically best matching reads in the mature region were regarded as its abundance value. Using the AASRA [67] method, the optimal match results for each Read were selected, followed by calculating the Read count for each non-coding RNA and normalizing the expression levels by the library’s Total Clean Reads. The transcripts per million (TPM) method was used as following: TPM = Reads of a non-coding RNA × 10^6^/library size of the sample. Based on miRNA expression (TPM), the DESeq2 [68] method was used to calculate the difference in fold change, and significance of the value was determined as follows: |foldchange, FC| ≥ 1 and q-value < 0.001. These were used as criteria for screening differentially accumulating miRNAs in various comparisons (RCK vs. RT1, RCK vs. RT2 and RCK vs. RT3, respectively). miRNA target gene prediction was performed with the help of psRobot (v1.2) [26] and TargetFinder (v1.0) software [27], and the intersection of the results was taken as the final outcome of target prediction. miRNA-mRNA network was constructed using Cytoscape (v3.10.0). GO and KEGG enrichment pathway analysis was performed by using the phyper function in the R software package (v4.2.3), and the correction (Q) value was set to 0.05. GO enrichment analysis of miRNA target genes of *A. marina* was performed against the Gene Ontology database (http://www.geneontology.org/); KEGG pathway analysis was performed by using the following KEGG database website, http://www.genome.jp/kegg/ (accessed on 25 August 2025).

### 4.6. Quantitative Analysis of miRNA Expression Levels by Real-Time Quantitative Polymerase Chain Reaction

cDNA was reverse transcribed from RNA of 12 samples of *A. marina* (RCK_a, RCK_b, RCK_c, RT1_a, RT1_b, RT1_c, RT2_a, RT2_b, RT2_c, RT3_a, RT3_b and RT3_c). Real-time quantitative PCR (RT-qPCR) was performed using the SYBR^®^ Premix Ex TaqTM II kit (TaKaRa Bio Inc., Kusatsu, Shiga, Japan) through a CFX Connect™ Real-Time PCR Detection System (Bio-Rad Laboratories, Inc., Hercules, CA, USA). The miRNA stem-loop reverse transcription primers, forward amplification primers, universal reverse primers, target-gene-specific forward and reverse amplification primers, as well as the primer pairs for the internal reference genes U6 and 18S rRNA employed in the RT-qPCR assays are presented in Table S5. The PCR program included an initial denaturation at 95 °C for 30 s, and 40 cycles of 5 s at 95 °C, and 60 °C for 30 s. Specificity of the amplified fragments was verified by analyzing the generated melting curves, and the relative expression levels of each miRNA and target gene were calculated using the 2^−△△Ct^ method, with normalization to U6 and 18S rRNA [69]. All the RT-qPCR amplifications were performed in triplicate (technical replicates) to ensure repeatability and reliability.

## 5. Conclusions

In summary, this study employed sRNA sequencing and bioinformatic approaches to systematically elucidate the response mechanisms of miRNAs and their target genes in *A*. *marina* under chromium (Cr) stress. By analyzing the differential accumulation profiles of miRNAs between the control and Cr-treated groups, a regulatory network linking differentially accumulated miRNAs and their target genes was constructed. The study further provided a detailed analysis of the specific regulatory roles of miRNAs in three key pathways: “Plant hormone signal transduction” (ko04075), “MAPK signaling pathway” (ko04016), and “Phenylpropanoid biosynthesis” (ko00940). Based on the findings, a Cr stress-responsive regulatory model in *A. marina* was developed, offering new insights into the molecular regulatory mechanisms underlying its response to Cr. These results lay a foundation for elucidating the miRNA-mediated genetic regulatory mechanisms in plants under chromium stress.

## Figures and Tables

**Table 1:** Statistical details of high-throughput sRNA sequencing data of *A. marina* roots.

Sample ID	Raw Reads	Total Clean Reads	Total UMI Reads	Mapped Reads	Clean Rate (%)	Mapped Ratio (%)	Q20 (%)	Q30 (%)
RCK_a	29,634,273	27,804,655	27,082,590	19,491,219	93.83	71.97	98.60	94.80
RCK_b	29,513,299	26,811,629	26,404,150	20,139,825	90.85	76.28	98.50	94.60
RCK_c	30,071,143	24,301,766	23,796,246	19,071,198	80.81	80.14	98.50	94.50
RT1_a	29,477,128	27,816,684	27,267,866	19,275,716	94.37	70.69	98.50	94.70
RT1_b	29,481,310	26,511,028	25,839,728	18,765,043	89.92	72.62	98.30	93.80
RT1_c	28,755,655	27,505,483	26,736,207	18,068,893	95.65	67.58	98.30	94.00
RT2_a	29,298,241	25,087,205	24,517,428	17,824,186	85.63	72.70	98.30	94.00
RT2_b	33,802,816	23,767,194	23,081,243	17,694,680	70.31	76.66	98.60	95.10
RT2_c	28,987,867	27,027,638	26,589,376	17,965,834	93.24	67.57	98.60	94.90
RT3_a	27,804,891	25,172,596	24,757,000	17,772,056	90.53	71.79	98.50	94.60
RT3_b	29,630,655	28,167,379	27,745,846	18,539,128	95.06	66.82	98.50	94.80
RT3_c	35,294,117	23,987,153	23,405,602	18,375,654	67.96	78.51	98.80	95.70

Note: RCK refers to mock control group; RT1, RT2 and RT3 refer to groups of plants treated with Cr for 14 days; a, b and c refer to 3 biological replicates; Q20 and Q30 are the proportion of base numbers with mass value ≥ 20 and 30, respectively, as compared to the total base numbers.

## Data Availability

The raw sequence data reported in this study have been deposited into the Genome Sequence Archive (https://ngdc.cncb.ac.cn/gsa/ (accessed on 17 July 2025)), the National Genomics Data Center, China National Center for Bioinformation/Beijing Institute of Genomics, Chinese Academy of Sciences. Sequence data are available under accession number CRA028092.

## Supplementary Materials

The following supporting information can be downloaded at: https://www.mdpi.com/article/10.3390/plants15101451/s1, Table S1: Effects of different Cr concentrations on the growth of *A. marina* seedlings; Table S2: Differentially expressed miRNAs in *A. marina* after chromium treatment (q-value < 0.001); Table S3: Differentially expressed target genes for *A. marina* in control and Cr treated plants (q-value < 0.05); Table S4: Functional annotation of expressed genes that are predicted to be involved in response to Cr stress; Table S5: Real-time PCR primer sequences used for miRNAs and their corresponding targets; Figure S1: Distribution of clean reads base quality across the samples; Figure S2: Pearson correlation coefficients amongst the samples; Figure S3: Annotations and analysis of sRNA sequence length characteristics; Figure S4: Hairpin structures of novel miRNAs identified in *A. marina*; Figure S5: Heatmap of expression levels for differentially expressed target genes in *A. marina*; Figure S6: RNA quality assessment using Agilent 2100; Data S1: Details of known miRNAs identified in *A. marina*; Data S2: Detailed information of novel miRNAs identified in *A. marina*; Data S3: Target prediction for in *A. marina* differentailly accumualted miRNAs with the help of psRobot and TargetFinder softwares; Data S4: Expression levels of target genes corresponding to the differentially accumulated miRNAs; Data S5: GO annotation of target genes of differentially expressed miRNAs; Data S6: KEGG annotation of target genes of differentially expressed miRNAs; Data S7: A list of pathways for target genes of differentially expressed miRNAs.
