# Supplementary material for: miRNA as Modifiers of Chromium (Cr) Stress in Mangrove Avicennia marina
Source: Plants (Basel). 2026 May 9;15(10):1451. doi: 10.3390/plants15101451 (PMC13211104; doi:10.3390/plants15101451)
Supplement: Supplementary file 1 [file plants-15-01451-s001.zip › Supplementary Tables and Figures.pdf]

Supplementary Materials

miRNA as modifiers of chromium (Cr) stress in mangrove *Avicennia marina*

Beibei Chen[1], Quanhu Zhao[1], Yujian Mo[1], Qingzhi Liang[1], Lishan Zhen[1], Jian Yang[1], Xiao Xiao[2*]

[1] College of Coastal Agricultural Sciences, Guangdong Ocean University, Zhanjiang, 524088, PR China

[2] School of Chemistry and Environment, Guangdong Ocean University, Zhanjiang, 524088, PR China

* Correspondence: xxiao8922@hotmail.com

The following Supporting Information is available for this article:

Supplementary Table S1. Effects of different Cr concentrations on the growth of *A. marina* seedlings

Supplementary Table S2. Differentially expressed miRNAs in *A. marina* after chromium treatment (q-value < 0.001).

Supplementary Table S3. Differentially expressed target genes for *A. marina* in control and Cr treated plants (q-value < 0.05).

Supplementary Table S4. Functional annotation of expressed genes that are predicted to be involved in response to Cr stress.

Supplementary Table S5. Real-time PCR primer sequences used for miRNAs and their corresponding targets.

Supplementary Figure S1. Distribution of clean reads base quality across the samples.

Supplementary Figure S2. Pearson correlation coefficients amongst the samples.

Supplementary Figure S3. Analysis of sRNA sequence length characteristics and classification (annotations).

Supplementary Figure S4. Hairpin structures of novel miRNAs identified in *A. marina.*

Supplementary Figure S5. Heatmap of expression levels for differentially expressed target genes in *A. marina*.

Supplementary Figure S6. RNA quality assessment using Agilent 2100.

Table S1. Effects of different Cr concentrations on the growth of *A. marina* seedlings

Cr concentration (mg/L)	Plant height (cm)	Root length (cm)	Biomass (g)		Root / shoot ratio
			Shoot	Root	
RCK	29.67±0.15 bc	12.90±0.66 c	2.63±0.66 a	1.59±0.62 a	0.63±0.32 a
100	29.07±1.15 c	13.73±0.50 bc	2.45±0.70 a	1.27±0.51 a	0.51±0.07 a
200	31.43±0.66 ab	14.17±0.75 b	2.61±0.55 a	1.42±0.23 a	0.55±0.09 a
300	32.43±0.32 a	15.53±0.31 a	2.54±0.27 a	1.61±0.86 a	0.64±0.10 a
400	30.20±1.14 bc	13.60±0.10 bc	2.89±0.44 a	1.93±0.54 a	0.66±0.09 a
500	29.80±0.44 bc	13.57±0.46 bc	2.89±0.74 a	1.89±0.38 a	0.66±0.15 a
600	28.53±0.31 c	13.60±0.26 bc	2.73±0.54 a	1.79±0.30 a	0.67±0.11 a

Note: Within the same column, means with different letters are significantly different ($P < 0.05$). Root / shoot ratio = root dry weight / shoot dry weight.

Table S2. Differentially expressed miRNAs in *A. marina* after chromium treatment (q-value < 0.001).

miRNA ID	RCK-VS-RT1					RCK-VS-RT2					RCK-VS-RT3				
	**RCK**	**RT1**	**q-value**	**Up/ Down**	**p-value**	**RCK**	**RT2**	**q-value**	**Up/ Down**	**p-value**	**RCK**	**RT3**	**q-value**	**Up/ Down**	**p-value**
Ama-miR156a	/	/	/	/	/	/	/	/	/	/	43	109	2.40242813559562e-12	Up	5.46404616389598e-12
Ama-miR160	/	/	/	/	/	/	/	/	/	/	42	14	0.000759522958974386	Down	0.00280710221023418
Ama-miR396	2615	4798	0	Up	0	/	/	/	/	/	/	/	/	/	/
Ama-miR396-3p	/	/	/	/	/	39	67	5.28196471469107e-07	Up	9.24496616662511e-07	/	/	/	/	/
ama-miR396-5p	1025	1309	5.76676690466529e-178	Up	6.53386912685063e-178	1025	1351	4.17646222567786e-65	Up	3.50880817043137e-65	/	/	/	/	/
Ama-miR408	108	259	6.41408613266806e-64	Up	1.14421248798538e-63	108	177	1.29059707912987e-14	Up	1.6625638099044e-14	108	220	1.82108629830933e-17	Up	3.88298540090891e-17
Ama-nmiR1-3p	/	/	/	/	/	/	/	/	/	/	431	161	6.53870055379302e-18	Down	1.3787139726699e-17
Ama-nmiR3-5p	/	/	/	/	/	/	/	/	/	/	7328	2005	0	Down	0
Ama-nmiR4-5p	/	/	/	/	/	3570	952	8.93056377868489e-169	Down	4.12660246950207e-169	/	/	/	/	/
Ama-nmiR8-3p	247	290	5.47889076801287e-37	Up	1.14908477563511e-36	/	/	/	/	/	247	391	7.0767474572249e-19	Up	1.42510001522225e-18

Ama-nmiR10-5p	/	/	/	/	/	259	541	1.89699041230391e-57	Up	1.67342211018889e-57	/	/	/	/	/
Ama-nmiR12-3p	749	1217	1.39858649345503e-215	Up	1.51719715479828e-215	/	/	/	/	/	/	/	/	/	/
Ama-nmiR15-3p	106	344	1.70240394668027e-11	Up	5.45826270593659e-11	/	/	/	/	/	/	/	/	/	/
Ama-nmiR16-5p	63	133	1.55655473744446e-05	Up	6.37901311388827e-05	63	122	3.57380863176139e-13	Up	4.90407593959674e-13	63	355	6.3888528381837e-66	Up	1.02925863408088e-65
Ama-nmiR17-5p	/	/	/	/	/	/	/	/	/	/	132	3070	0	Up	0
Ama-nmiR18-3p	/	/	/	/	/	69	13	5.64399462028446e-07	Down	1.00366811975553e-06	69	25	0.000125630233075386	Down	0.000416691769788631
Ama-nmiR21-3p	/	/	/	/	/	/	/	/	/	/	4403	7803	0	Up	0
Ama-nmiR25-5p	1692	331	2.10298692221738e-52	Down	3.90361944914631e-52	/	/	/	/	/	/	/	/	/	/
Ama-nmiR28-5p	1042	2176	0	Up	0	/	/	/	/	/	/	/	/	/	/
Ama-nmiR33-5p	/	/	/	/	/	16511	22510	0	Up	0	/	/	/	/	/
Ama-nmiR35-3p	/	/	/	/	/	715	228	2.36493800963263e-25	Down	2.48359537401773e-25	/	/	/	/	/
Ama-nmiR36-5p	170	334	2.57789808832867e-71	Up	4.47443719872926e-71	/	/	/	/	/	/	/	/	/	/
Ama-nmiR37-5p	170	334	2.57789808832867e-71	Up	4.47443719872926e-71	/	/	/	/	/	/	/	/	/	/

Ama-nmiR50-5p	141	324	1.61907041925308e-13	Up	4.95689403734961e-13	141	310	1.04057432809308e-35	Up	1.03450185428597e-35	141	789	2.91190589558491e-143	Up	3.31139733227694e-143
Ama-nmiR53-5p	1417	293	2.94861510451128e-39	Down	5.97086906943357e-39	/	/	/	/	/	/	/	/	/	/
Ama-nmiR59-3p	/	/	/	/	/	/	/	/	/	/	4403	7803	0	Up	0
Ama-nmiR60-5p	/	/	/	/	/	16489	22515	0	Up	0	/	/	/	/	/
Ama-nmiR63-3p	/	/	/	/	/	5758	8294	0	Up	0	5758	11716	0	Up	0
Ama-nmiR64-3p	226	278	9.36892281640587e-38	Up	1.94235357104429e-37	/	/	/	/	/	/	/	/	/	/
Ama-nmiR68-3p	61	98	3.75729952034428e-19	Up	9.41996593690203e-19	/	/	/	/	/	/	/	/	/	/
Ama-nmiR69-5p	/	/	/	/	/	255	527	2.57369699470506e-55	Up	2.37848911609564e-55	/	/	/	/	/
Ama-nmiR74-3p	61	98	3.75729952034428e-19	Up	9.41996593690203e-19	/	/	/	/	/	/	/	/	/	/
Ama-nmiR81-5p	48	59	1.17880188167405e-09	Up	3.92157100465593e-09	48	109	3.71153919869584e-14	Up	4.93716309940176e-14	48	78	1.73057021563849e-05	Up	5.16597727649415e-05
Ama-nmiR83-3p	/	/	/	/	/	3570	952	8.93056377868489e-169	Down	4.12660246950207e-169	/	/	/	/	/
Ama-nmiR87-5p	83	169	1.81522182362086e-06	Up	6.82644316237379e-06	/	/	/	/	/	/	/	/	/	/
Ama-nmiR88-5p	94	193	1.26576060904951e-10	Up	4.14983338776834e-10	94	151	2.21432538677557e-12	Up	3.22459069137345e-12	/	/	/	/	/

Ama-nmiR89-5p	68	108	1.13496862049067e-20	Up	2.70868950449228e-20	68	241	3.07825468900662e-42	Up	2.93098286758613e-42	/	/	/	/	/
Ama-nmiR90-3p	46	116	1.04197235063671e-30	Up	2.33603486034118e-30	/	/	/	/	/	/	/	/	/	/
Ama-nmiR92-5p	/	/	/	/	/	/	/	/	/	/	3	13	0.000664064577388245	Up	0.00242283454352731
Ama-nmiR93-5p	/	/	/	/	/	832	1533	4.56726648859262e-135	Up	2.94180581101375e-135	/	/	/	/	/
Ama-nmiR95-3p	/	/	/	/	/	/	/	/	/	/	431	161	6.53870055379302e-18	Down	1.3787139726699e-17
Ama-nmiR98-5p	1433	2380	4.37832668671137e-122	Up	5.27737971551368e-122	/	/	/	/	/	/	/	/	/	/
Ama-nmiR100-5p	99	175	2.31556066765036e-35	Up	4.91223360853259e-35	99	169	7.12988114353276e-15	Up	8.8853003787789e-15	/	/	/	/	/
Ama-nmiR108-5p	246	478	3.15117982563387e-100	Up	4.78579302983127e-100	/	/	/	/	/	/	/	/	/	/
Ama-nmiR110-5p	/	/	/	/	/	259	541	1.89699041230391e-57	Up	1.67342211018889e-57	/	/	/	/	/
Ama-nmiR113-3p	333	339	9.6270348424784e-35	Up	2.06548823831123e-34	333	939	1.53795734166142e-133	Up	1.01214320776029e-133	333	663	6.86211165935012e-47	Up	1.17053121172349e-46
Ama-nmiR115-5p	/	/	/	/	/	255	527	2.57369699470506e-55	Up	2.37848911609564e-55	/	/	/	/	/
Ama-nmiR116-3p	450	648	1.52743122056611e-102	Up	2.2092918460534e-102	/	/	/	/	/	450	712	1.02869325454874e-32	Up	1.85221844955476e-32
Ama-nmiR117-5p	/	/	/	/	/	2693	4105	6.92435662161391e-263	Up	2.4239254198127e-263	/	/	/	/	/

Ama-nmiR118-5p	/	/	/	/	/	2695	4107	6.92435662161391e-263	Up	2.41237010559948e-263	/	/	/	/	/
Ama-nmiR120-5p	/	/	/	/	/	101	158	2.26601721291208e-12	Up	3.36332540088054e-12	/	/	/	/	/
Ama-nmiR121-3p	41341	57598	0	Up	0	/	/	/	/	/	/	/	/	/	/
Ama-nmiR122-3p	106	243	1.70240394668027e-11	Up	5.45826270593659e-11	/	/	/	/	/	/	/	/	/	/
Ama-nmiR123-3p	64	88	4.48483317167979e-15	Up	1.24332397723765e-14	/	/	/	/	/	/	/	/	/	/
Ama-nmiR128-5p	/	/	/	/	/	101	158	2.26601721291208e-12	Up	3.36332540088054e-12	/	/	/	/	/
Ama-nmiR134-3p	120	140	8.57971149394004e-19	Up	2.17171164123171e-18	/	/	/	/	/	/	/	/	/	/
Ama-nmiR136-3p	/	/	/	/	/	26338	8209	0	Down	0	/	/	/	/	/
Ama-nmiR137-3p	105	255	6.47877227239761e-12	Up	2.04599320251633e-11	/	/	/	/	/	/	/	/	/	/
Ama-nmiR139-3p	893	1858	1.88168056697699e-76	Up	3.03921118230154e-76	/	/	/	/	/	/	/	/	/	/
Ama-nmiR140-5p	/	/	/	/	/	13	33	5.89384326365648e-06	Up	1.11412050757043e-05	13	43	2.4912262204668e-07	Up	6.72838353001646e-07
Ama-nmiR141-5p	/	/	/	/	/	2687	4093	8.48939926141401e-262	Up	3.0906521233506e-262	/	/	/	/	/
Ama-nmiR146-3p	/	/	/	/	/	69	13	5.64399462028446e-07	Down	1.00366811975553e-06	69	25	0.000125630233075386	Down	0.000416691769788631

Ama-nmiR149-5p	1427	2374	1.552166659 3622e-121	Up	1.908309018 01773e-121	/	/	/	/	/	/	/	/	/	/

Table S3. Differentially expressed target genes for *A. marina* in control and Cr treated plants (q-value < 0.05).

Gene ID	RCK-VS-RT1					RCK-VS-RT2					RCK-VS-RT3				
	RCK	RT1	p-value	Up/ Down	q-value	RCK	RT2	p-value	Up/ Down	q-value	RCK	RT3	p-value	Up/ Down	q-value
AM_01769	/	/	/	/	/	6.19	2.29	2.73E-05	Down	0.003715557	6.19	1.09	1.50E-05	Down	0.001018157
AM_02123	3.21	1.11	0.000759986	Down	0.011326388	/	/	/		/	/	/	/		/
AM_02383	/	/	/	/	/	/	/	/		/	2.20	5.84	0.001528101	Up	0.013959409
AM_04136	23.93	9.60	0.003458215	Down	0.024943293	/	/	/		/	/	/	/		/
AM_04151	/	/	/	/	/	/	/	/		/	101.96	23.41	0.002034688	Down	0.016773772
AM_04152	/	/	/	/	/	/	/	/		/	5.32	1.24	7.90E-05	Down	0.002053406
AM_09327	/	/	/	/	/	/	/	/		/	78.96	20.76	1.20E-05	Down	0.001018157
AM_09658	15.11	42.35	6.36E-06	Up	0.001078744	/	/	/		/	/	/	/		/
AM_11631	22.24	113.08	0.000596349	Up	0.011326388	/	/	/		/	/	/	/		/
AM_12529	/	/	/	/	/	/	/	/		/	657.38	162.97	0.003140922	Down	0.020093544
AM_12682	/	/	/	/	/	/	/	/		/	79.17	34.00	0.000746075	Down	0.008405779
AM_13110	/	/	/	/	/	/	/	/		/	1.92	5.14	4.96E-05	Up	0.001859518

AM_13259	/	/	/	/	/	/	/	/		/	9.96	21.49	0.00289486	Up	0.01956925
AM_14190	1.90	6.03	0.00029322	Up	0.008283458	/	/	/		/	/	/	/		/
AM_15404	/	/	/	/	/	6.32	14.28	6.50E-05	Up	0.003715557	6.32	17.54	5.30E-05	Up	0.001859518
AM_20145	/	/	/	/	/	4.93	1.43	0.004135405	Down	0.026732394	4.93	0.39	0.003374374	Down	0.020093544
AM_25083	0.47	2.84	0.006876752	Up	0.035864908	/	/	/		/	/	/	/		/
AM_25631	/	/	/	/	/	1.93	4.89	0.004969876	Up	0.028109915	1.93	5.44	0.002207192	Up	0.016955244
AM_25682	9.57	1.51	0.001533314	Down	0.014851245	/	/	/		/	9.57	3.86	0.00063026	Down	0.007681556
AM_26031	/	/	/	/	/	/	/	/		/	5.55	15.11	0.002697411	Up	0.019398399
AM_26867	103.08	38.55	0.000829621	Down	0.011326388	103.08	35.30	0.000310861	Down	0.008201952	103.08	26.32	0.001116209	Down	0.011096431
AM_27120	0.25	2.59	0.002219294	Up	0.018808514	/	/	/		/	/	/	/		/
AM_28129	/	/	/	/	/	/	/	/		/	6.23	15.26	0.006174013	Up	0.028946662
AM_28230	3.27	12.10	0.003779877	Up	0.025627563			/		/	3.27	9.18	0.000198395	Up	0.003529348
AM_30586	/	/	/	/	/			5.98E-05	Up	0.003715557	3.17	10.41	9.67E-05	Up	0.002335666

Table S4. Functional annotation of expressed genes that are predicted to be involved in response to Cr stress.

Gene ID	Abbreviation of gene	Annotated description	Cr-related pathway
AM_00238	ARF	hypothetical protein CDL12_09885	Plant hormone signal transduction
AM_00753	ARF	auxin response factor 18 isoform X1	Plant hormone signal transduction
AM_00948	GID1	probable carboxylesterase 18	Plant hormone signal transduction
AM_01106	TIR1	protein transport inhibitor response 1-like	Plant hormone signal transduction
AM_01808	POD	hypothetical protein CDL12_28560	Phenylpropanoid biosynthesis
AM_01870	ARF	hypothetical protein CDL12_27482	Plant hormone signal transduction
AM_02073	DELLA	scarecrow-like protein 27	Plant hormone signal transduction
AM_02875	DELLA	hypothetical protein F0562_028124	Plant hormone signal transduction
AM_03344	BAK1	brassionsteroid insensitive 1-associated receptor kinase 1 isoform X4	MAPK signaling pathway<br>Plant hormone signal transduction
AM_03692	CSE	alpha/beta-Hydrolases superfamily protein	Phenylpropanoid biosynthesis
AM_04618	BRI1	Squamosa promoter-binding-like protein 9	Plant hormone signal transduction
AM_04698	GID1	probable carboxylesterase 15	Plant hormone signal transduction
AM_05276	TIR1	protein transport inhibitor response 1-like	Plant hormone signal transduction
AM_06040	BRI1	hypothetical protein CDL12_12167	Plant hormone signal transduction
AM_07023	A-ARR	hypothetical protein SASPL_129150	Plant hormone signal transduction
AM_07229	FLS2	DNA-damage-repair/toleration protein drt100	MAPK signaling pathway
AM_08342	DELLA	hypothetical protein CRG98_002561	Plant hormone signal transduction
AM_08392	BRI1	squamosa promoter-binding-like protein 6	Plant hormone signal transduction

AM_08730	ARF	auxin response factor 18-like	Plant hormone signal transduction
AM_09201	MPK4	hypothetical protein JHK87_013503	MAPK signaling pathway
AM_10270	BRI1	squamosa promoter-binding-like protein 6	Plant hormone signal transduction
AM_11047	BRI1	hypothetical protein SASPL_144274	Plant hormone signal transduction
AM_11328	ARF	hypothetical protein CDL12_27482	Plant hormone signal transduction
AM_11874	BRI1	squamosa promoter-binding-like protein 9	Plant hormone signal transduction
AM_13110	ARF	hypothetical protein CDL12_18265	Plant hormone signal transduction
AM_13259	ARF	auxin response factor 6 isoform X3	Plant hormone signal transduction
AM_14358	WRKY33	hypothetical protein CDL12_23770	MAPK signaling pathway
AM_14384	BRI1	squamosa promoter-binding-like protein 6	Plant hormone signal transduction
AM_14586	VSP2	probable LRR receptor-like serine/threonine-protein kinase At1g06840 isoform X2	MAPK signaling pathway
AM_15461	FLS2	Serine/threonine protein kinase	MAPK signaling pathway
AM_15823	TIR1	protein auxin signaling F-box 3	Plant hormone signal transduction
AM_16674	ARF	hypothetical protein CDL12_18265	Plant hormone signal transduction
AM_17774	BRI1	probable LRR receptor-like serine/threonine-protein kinase At2g24230	Plant hormone signal transduction
AM_18237	BRI1	squamosa promoter-binding-like protein 7 isoform X2	Plant hormone signal transduction
AM_18256	DELLA	scarecrow-like protein 6	Plant hormone signal transduction
AM_18553	BRI1	squamosa promoter-binding-like protein 16-like	Plant hormone signal transduction
AM_19001	BAK1	hypothetical protein GOBAR_AA21013	MAPK signaling pathway<br>Plant hormone signal transduction
AM_19002	BAK1	hypothetical protein GOBAR_AA21013	MAPK signaling pathway<br>Plant hormone signal transduction
AM_19233	TIR1	protein transport inhibitor response 1-like	Plant hormone signal transduction

AM_19453	DELLA	DELLA protein RGL1-like	Plant hormone signal transduction
AM_19637	ARF	hypothetical protein CDL12_14827	Plant hormone signal transduction
AM_20145	RNA1	protein nuclear fusion defective 4-like	MAPK signaling pathway
AM_21858	BRI1	hypothetical protein CDL12_04042	Plant hormone signal transduction
AM_22164	PR1	pathogenesis-related protein 6	MAPK signaling pathway<br>Plant hormone signal transduction
AM_22558	DELLA	scarecrow-like protein 6	Plant hormone signal transduction
AM_23442	BRI1	squamosa promoter-binding-like protein 16-like	Plant hormone signal transduction
AM_23451	BRI1	squamosa promoter-binding-like protein 12	Plant hormone signal transduction
AM_24908	RNA1	copper-transporting ATPase PAA2, chloroplastic	MAPK signaling pathway
AM_25083	ARF	auxin response factor 6 isoform X3	Plant hormone signal transduction
AM_25618	ARF	hypothetical protein CDL12_27482	Plant hormone signal transduction
AM_26011	TIR1	Leucine rich repeat protein	Plant hormone signal transduction
AM_26379	ARF	auxin response factor 18	Plant hormone signal transduction
AM_29088	TF	Low quality protein transcription factor UNE10	Plant hormone signal transduction
AM_31798	ARF	hypothetical protein CDL12_09885	Plant hormone signal transduction

Table S5. Real-time PCR primer sequences used for miRNAs and their corresponding targets.

**Gene ID**	**Stem-loop primer**	**Forward primer（5′ → 3′）**	**Reverse primer（5′ → 3′）**
Ama-miR396	GTCGTATCCAGTGCAGGGTCCGAGGTATTCGCACTGGATACGACCAGTTC	GCGGCGGTTCCACAGCTTTCTT	
Ama-miR396-5p	GTCGTATCCAGTGCAGGGTCCGAGGTATTCGCACTGGATACGACAAGTTC	GCGGCGGTTCCACAGCTTTCTT	
Ama-nmiR60-5p	GTCGTATCCAGTGCAGGGTCCGAGGTATTCGCACTGGATACGACGGTGCT	GCGGCGGAAGCTCAGGAGGGAT	
Ama-nmiR121-3p	GTCGTATCCAGTGCAGGGTCCGAGGTATTCGCACTGGATACGACTAGGAA	GCGGCGGTTTCCAACTCCACCCA	
Ama-nmiR136-3p	GTCGTATCCAGTGCAGGGTCCGAGGTATTCGCACTGGATACGACTTGGGA	GCGGCGGTTTCCAATGCCGCCCA	
AM_31331		AAAAGTCTTGCCTTCAATAGC	ACATATCCGACCGTGCATAG
AM_14087		CGACAACTTTCGCAGGTAAC	TCAGGCTCTACTTCATAAACTC
AM_15461		ACTTTTTGTATTTGTAGAGAG	TTCCCGCATTGATGGTCTA
AM_14358		GAATCCGATGGCCGAGAACAA	CGAATGAATAGAAAAAAAAAG
AM_22445		TCCTCGAAGAAGCTGCTGAC	AATGCCGTCCATCCCCACAAA
Universal reverse primer for miRNA			GTGCAGGGTCCGAGGT
U6		GATAAAATTGGAACGATACAG	GGACCATTTCTCGATTTGTGCG
18s rRNA		CCGCCTCTGGTGTGCACCGGTC	CCCCCGGAACCCAAGGACTTTG

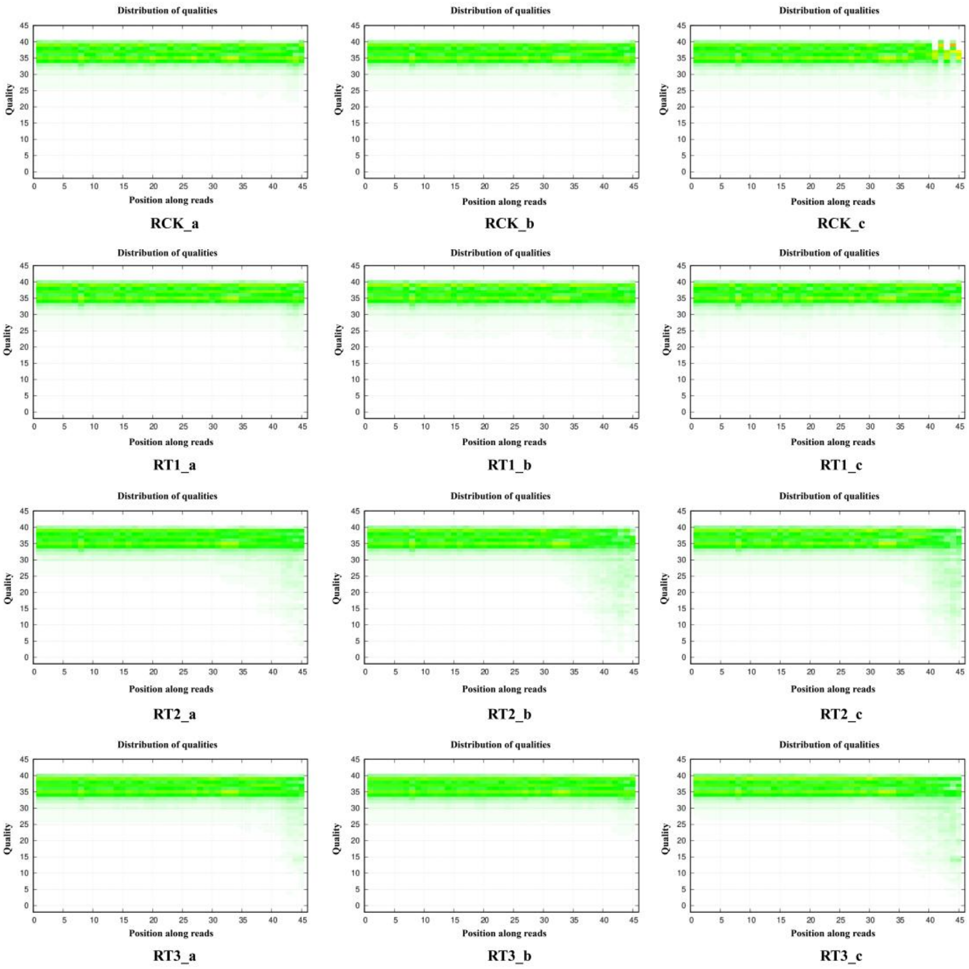

Figure S1. Distribution of clean reads base quality across the samples.

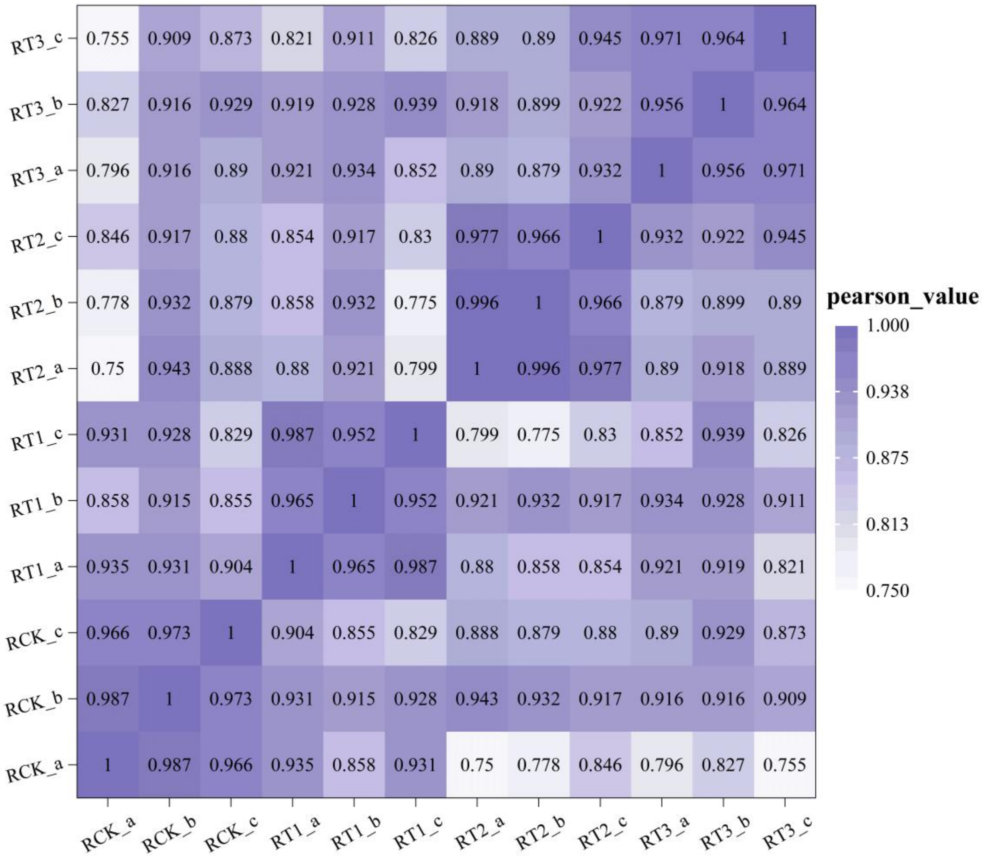

Figure S2. Pearson correlation coefficients amongst the samples.

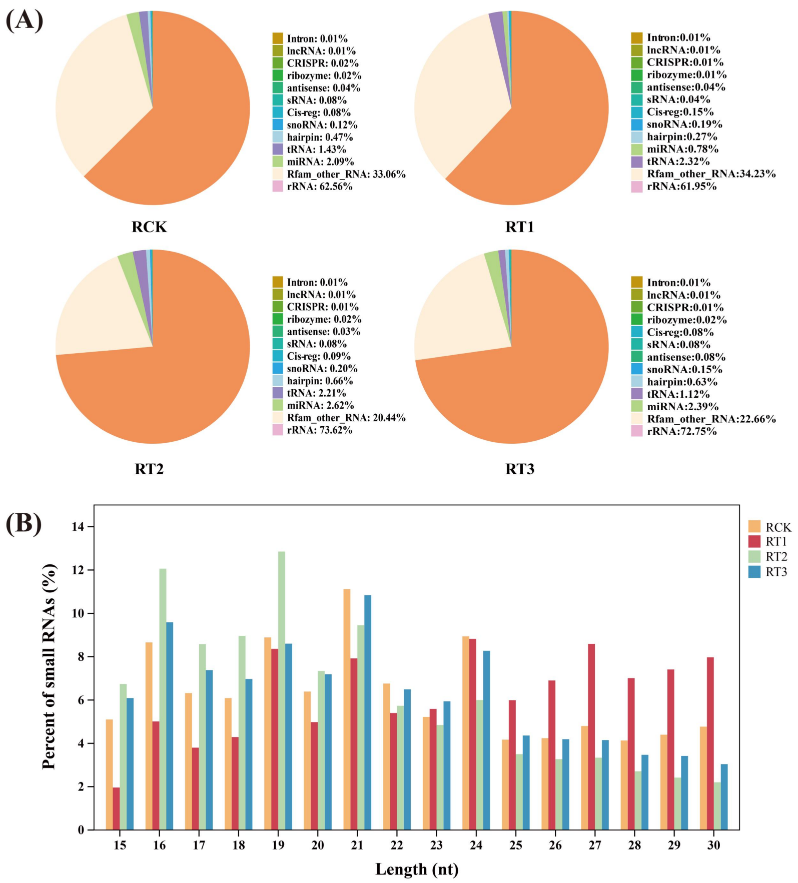

Figure S3. Analysis of sRNA sequence length characteristics and classification (annotations). (A) Size distribution of small RNA sequences in different libraries, nt denotes nucleotides. (B) Annotations of non-coding RNAs; different colors denote various non-coding RNA types.

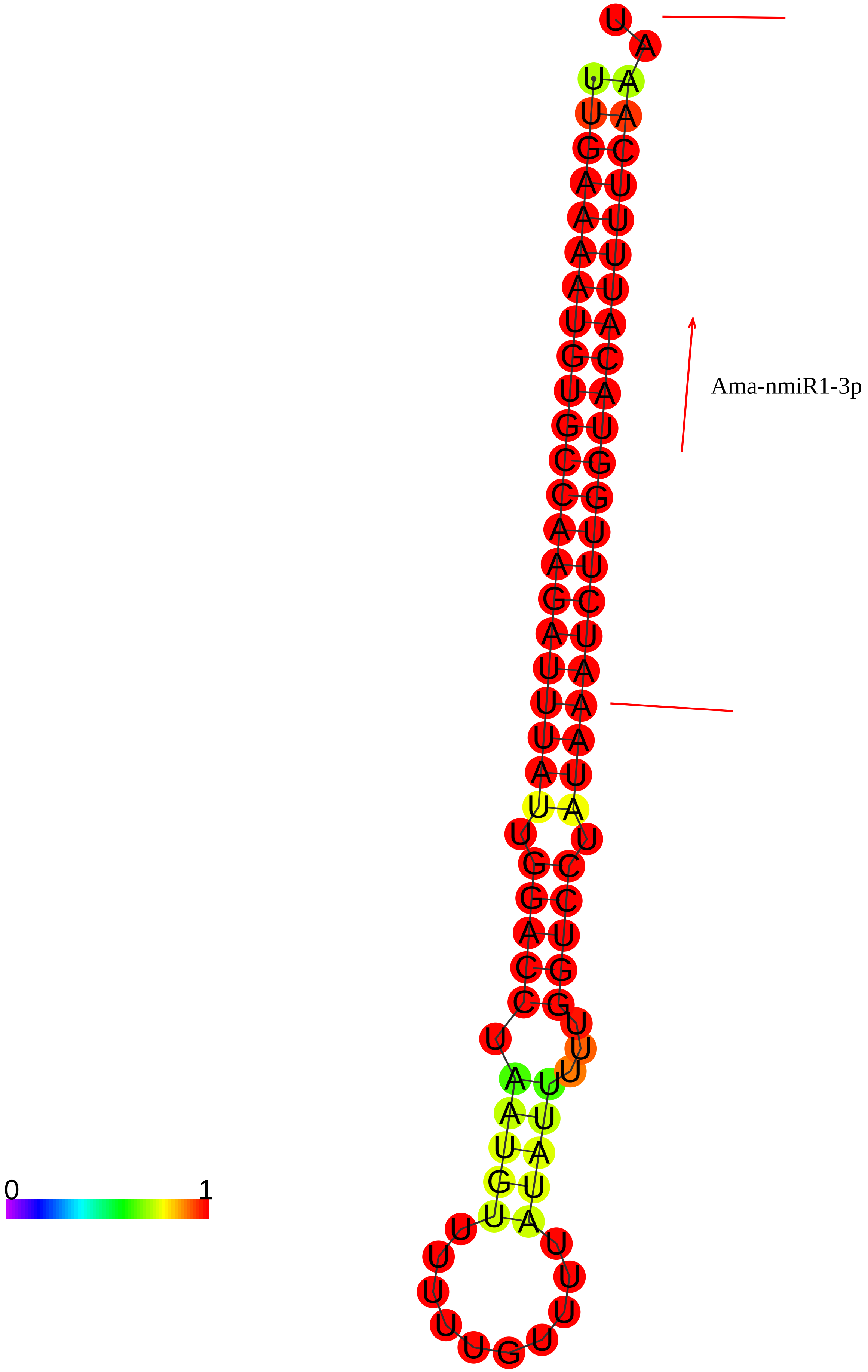

Ama-nmiR1-3p
0
1

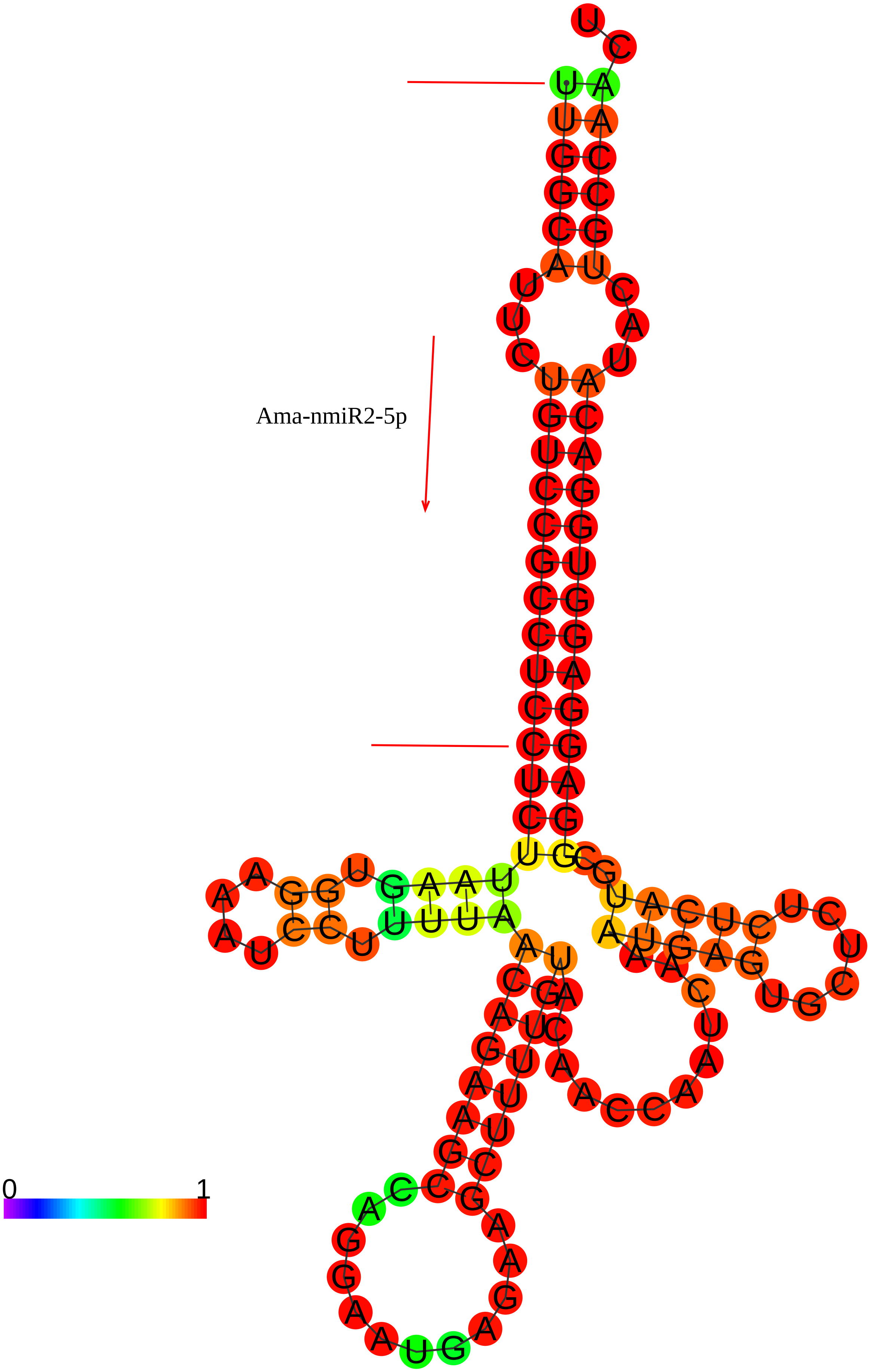
Ama-nmiR2-5p
0
1

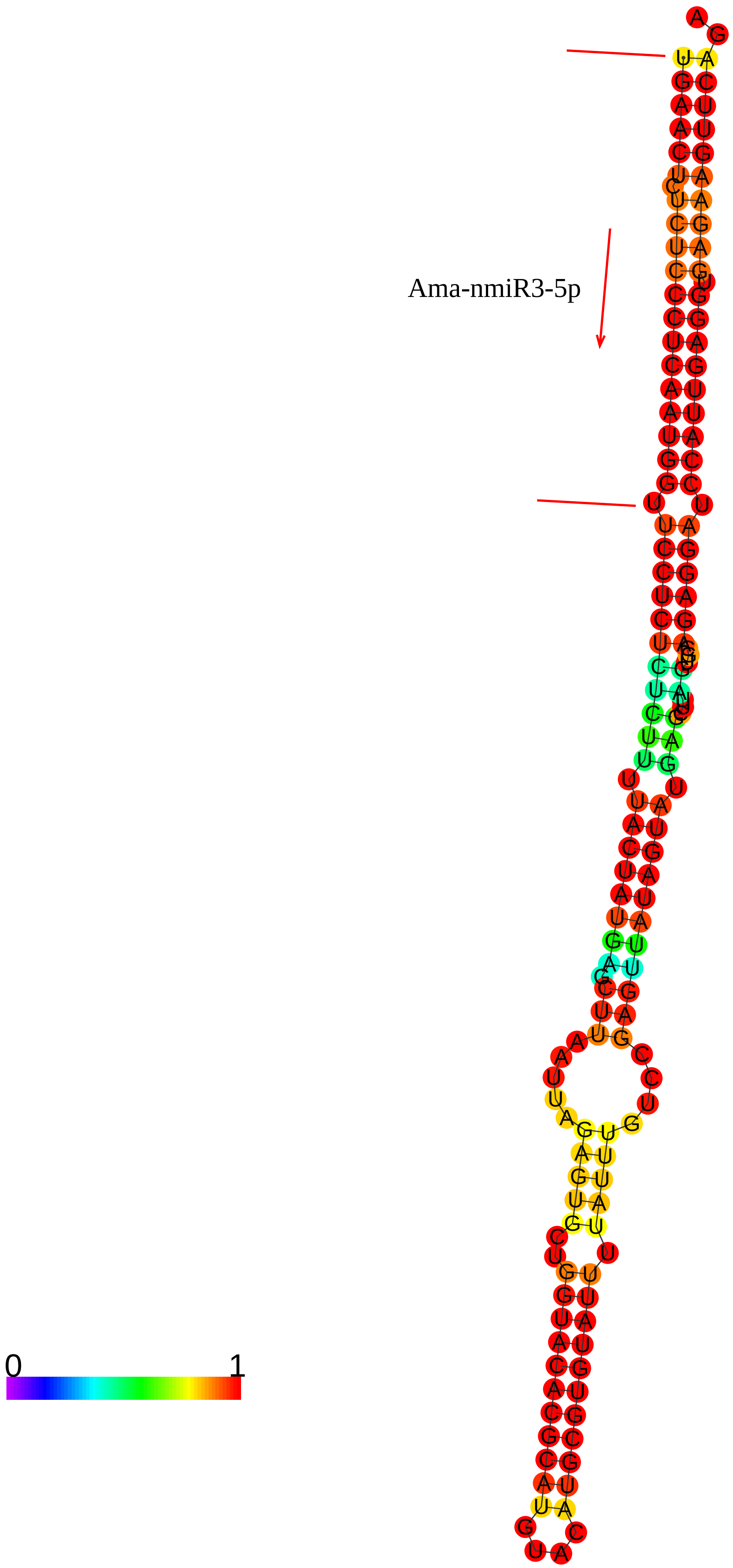
Ama-nmiR3-5p
0
1

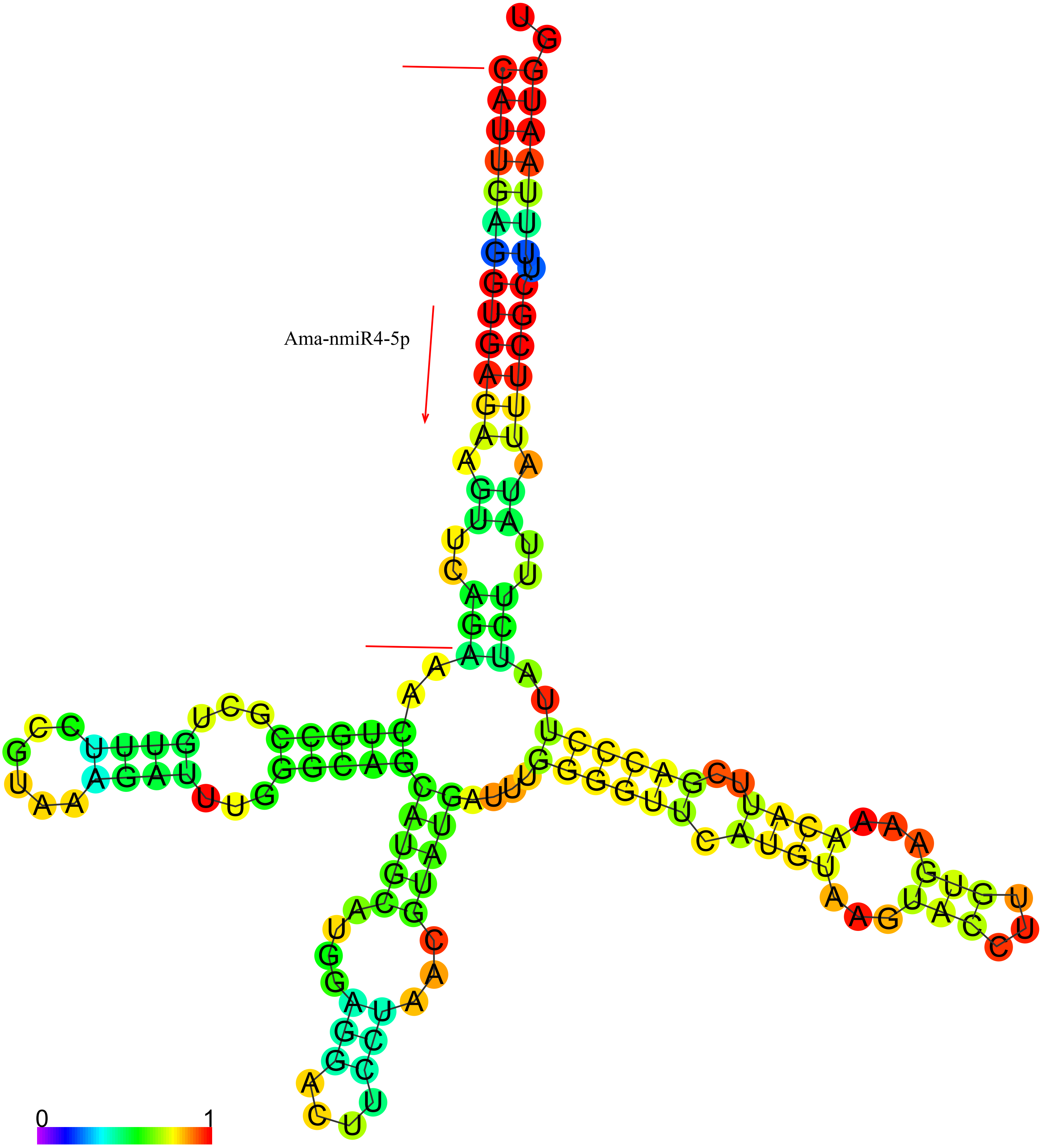

Ama-nmiR4-5p
0
1

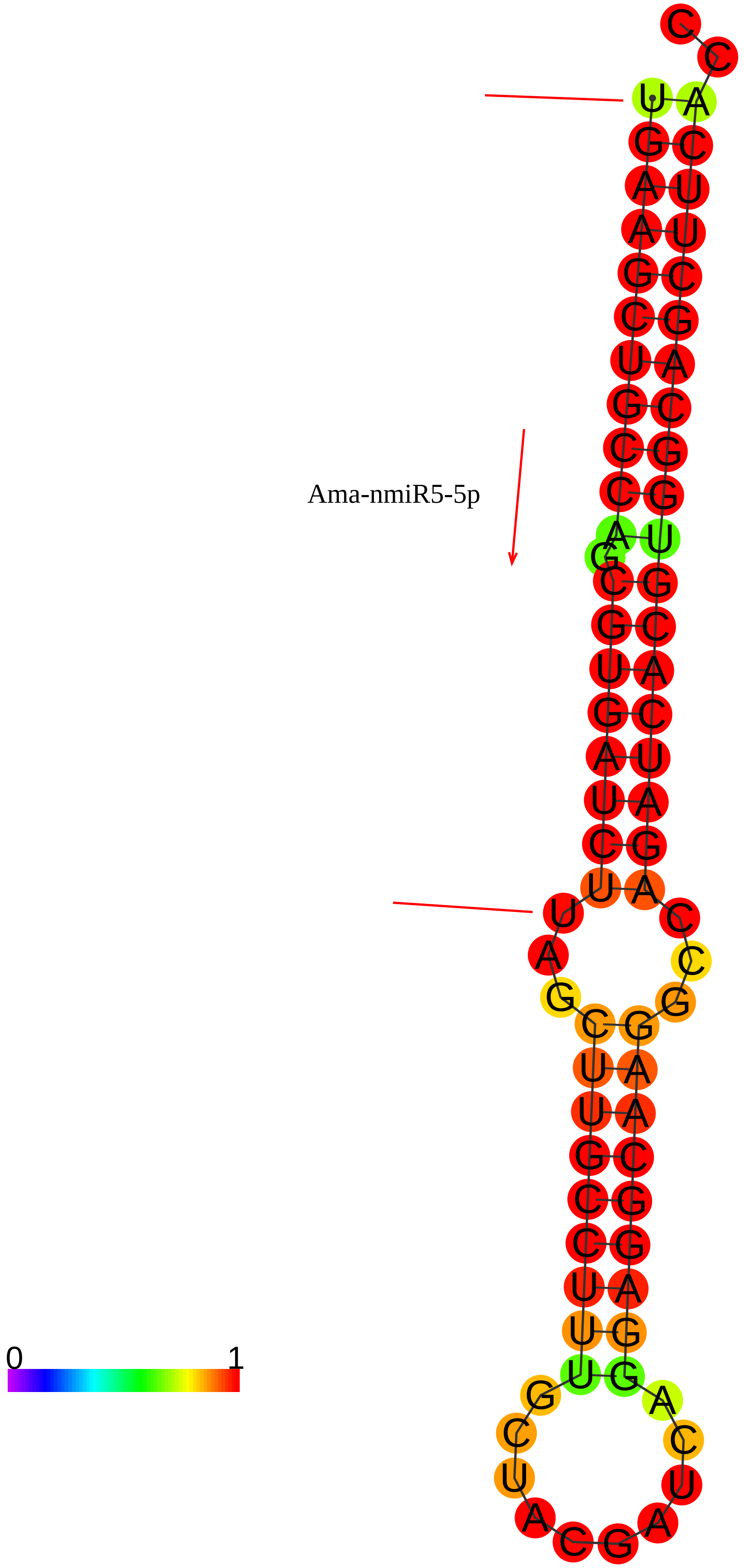

Ama-nmiR5-5p
0
1

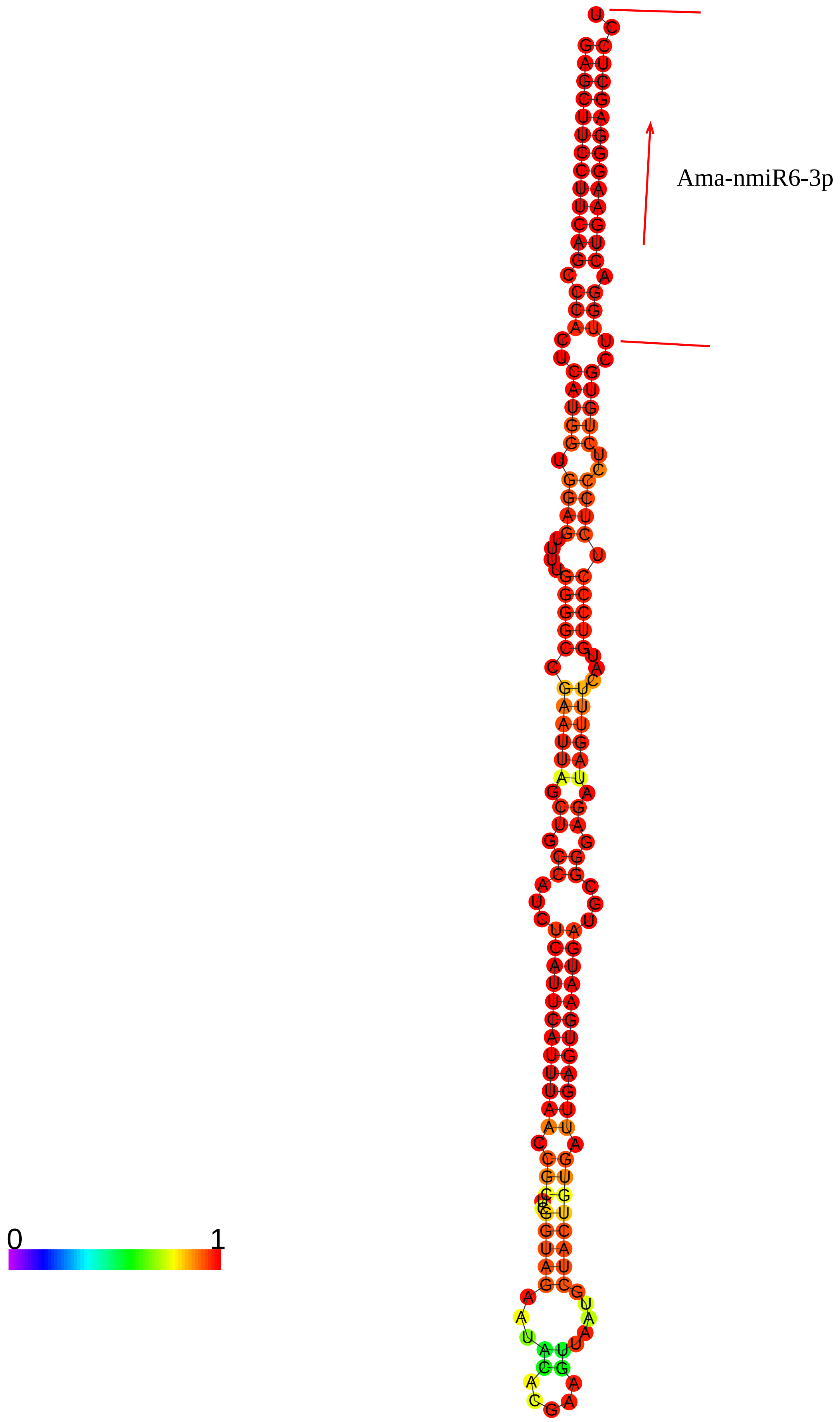

Ama-nmiR6-3p
0
1

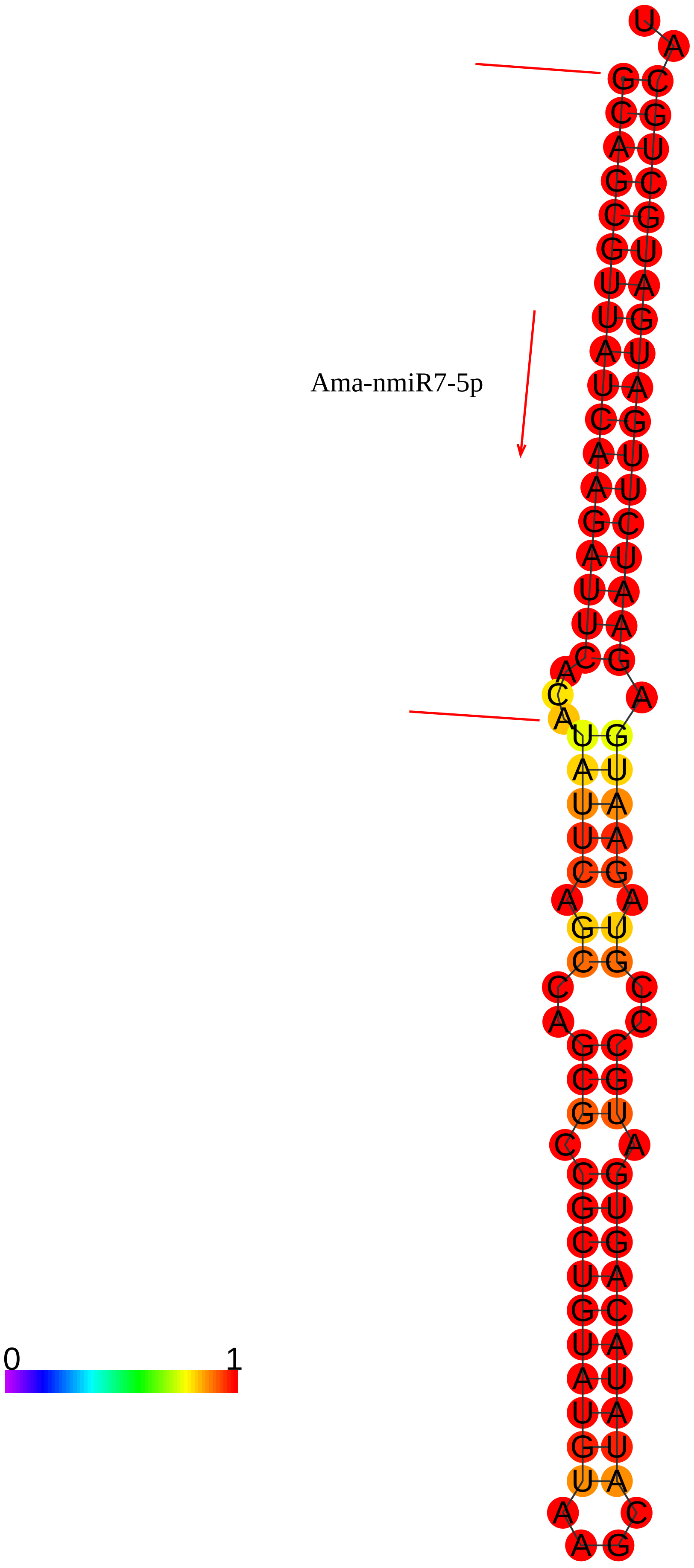
Ama-nmiR7-5p
0
1

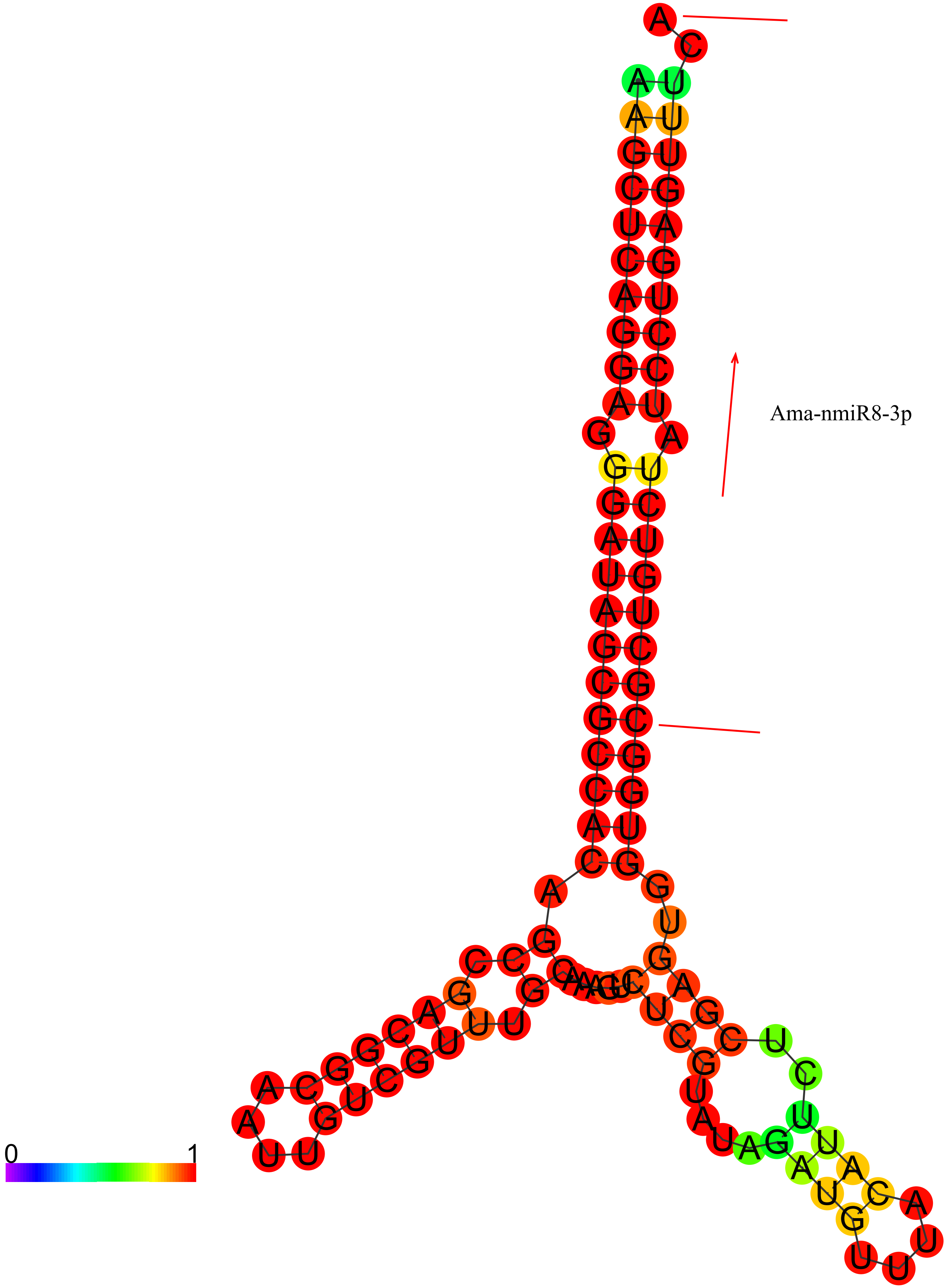
Ama-nmiR8-3p
0
1

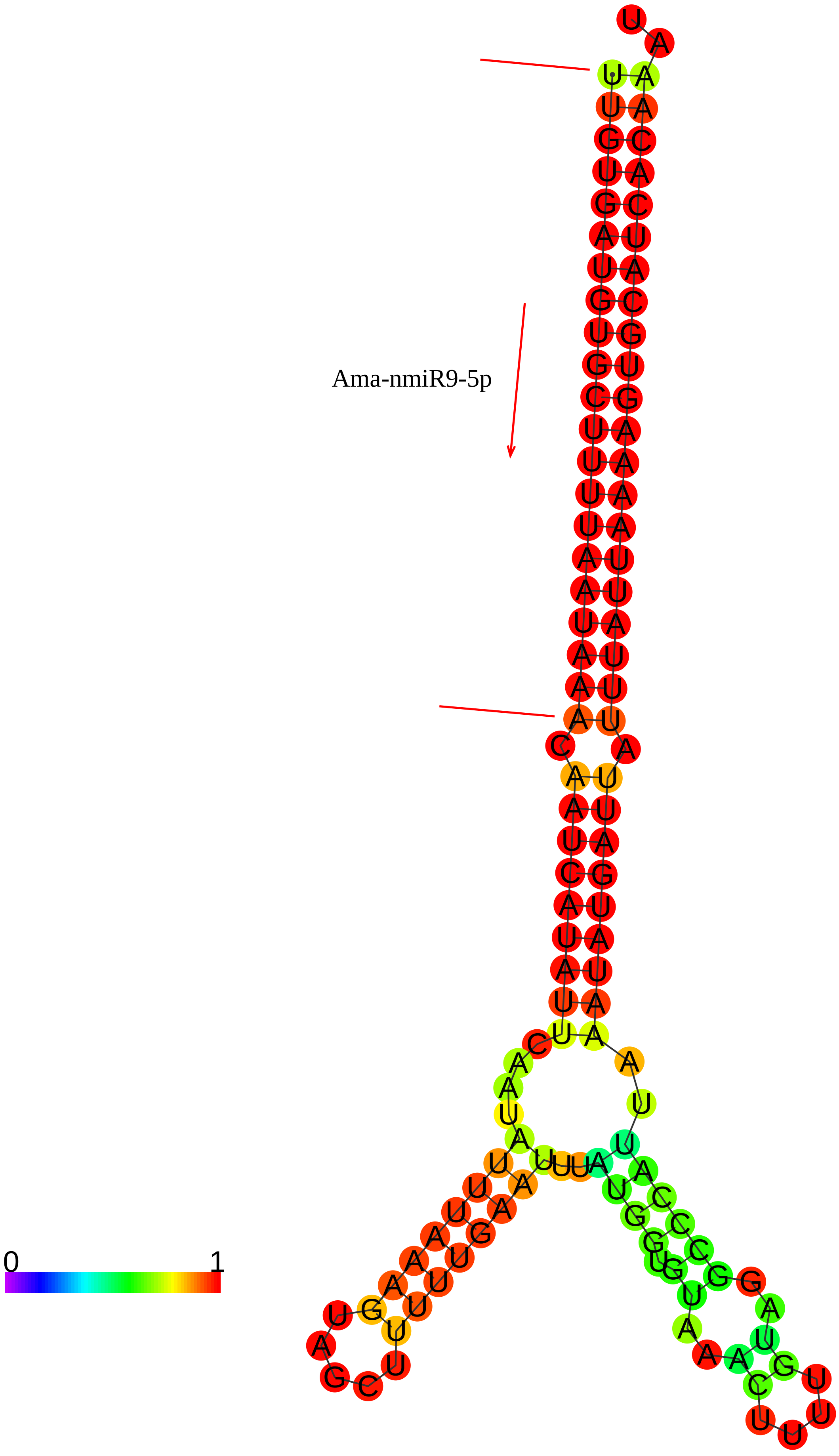
Ama-nmiR9-5p
0
1

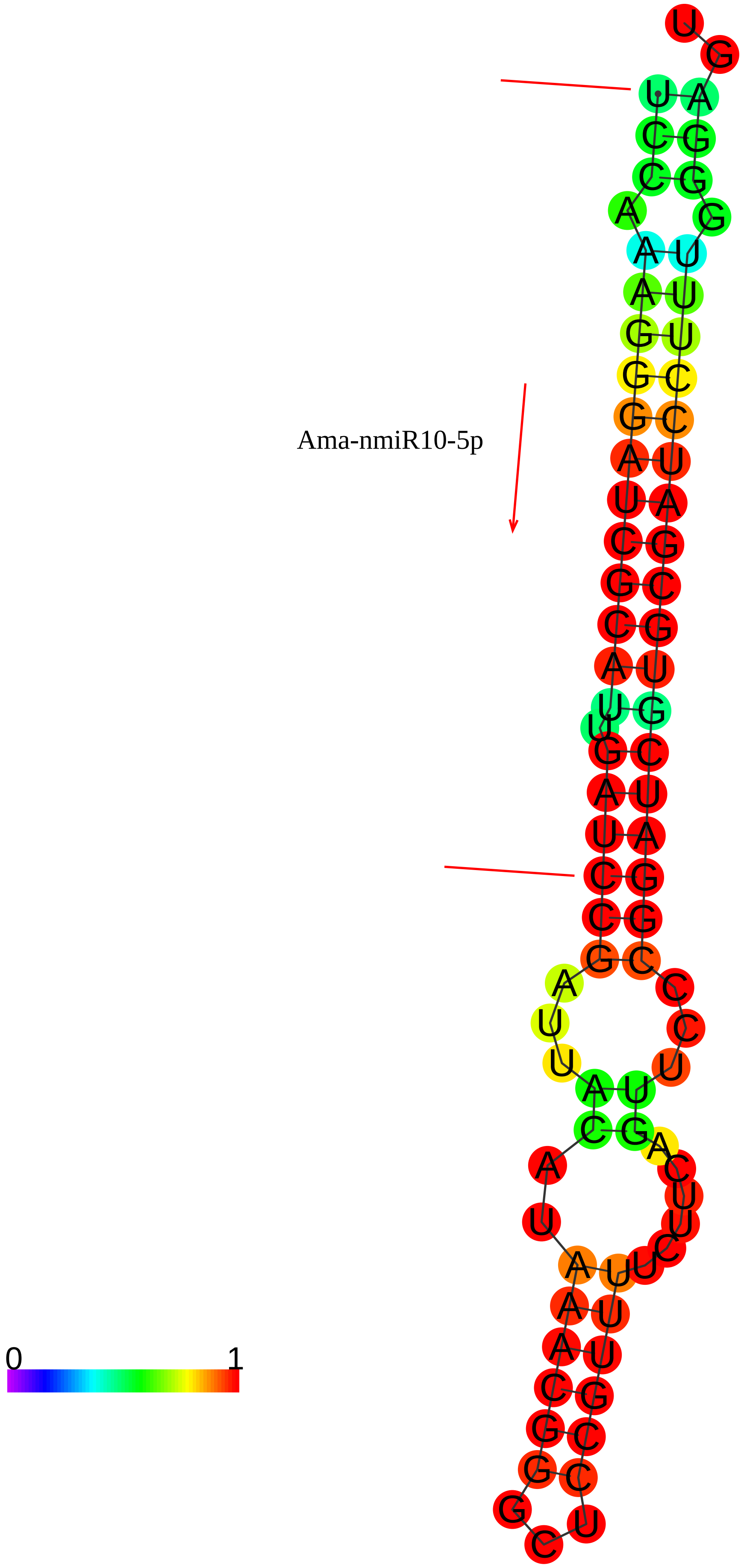
Ama-nmiR10-5p
0
1

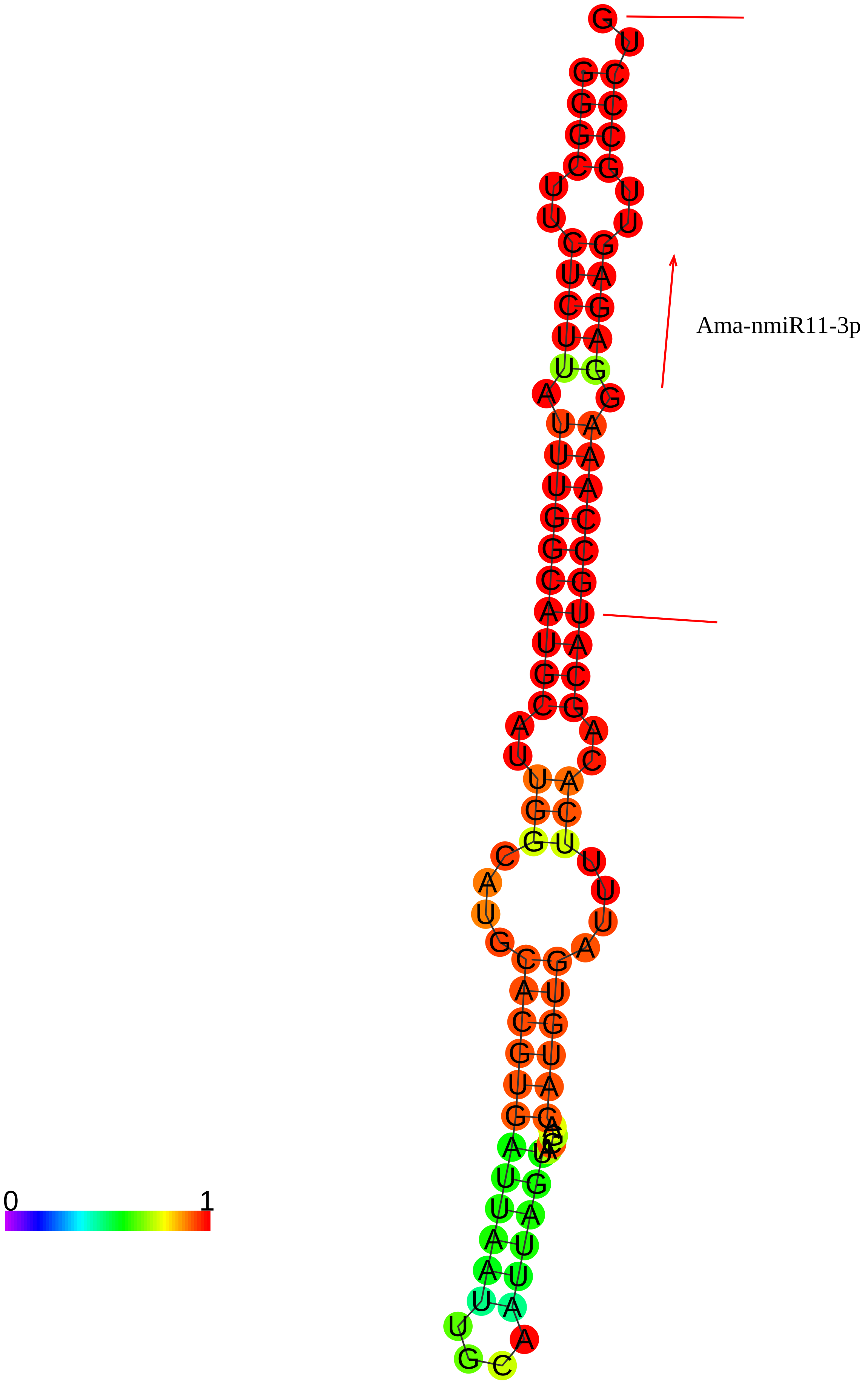
Ama-nmiR11-3p
0
1

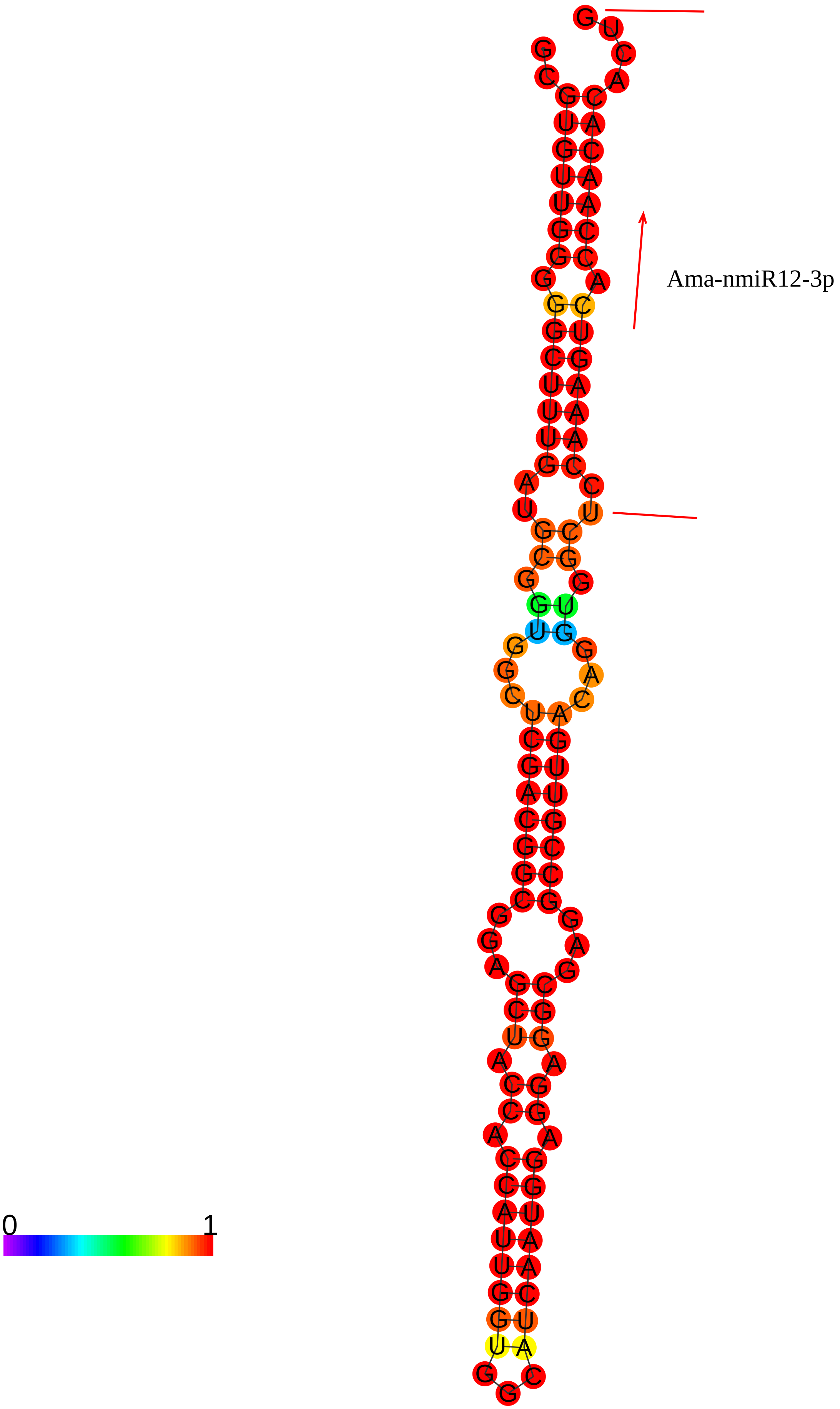
Ama-nmiR12-3p
0
1

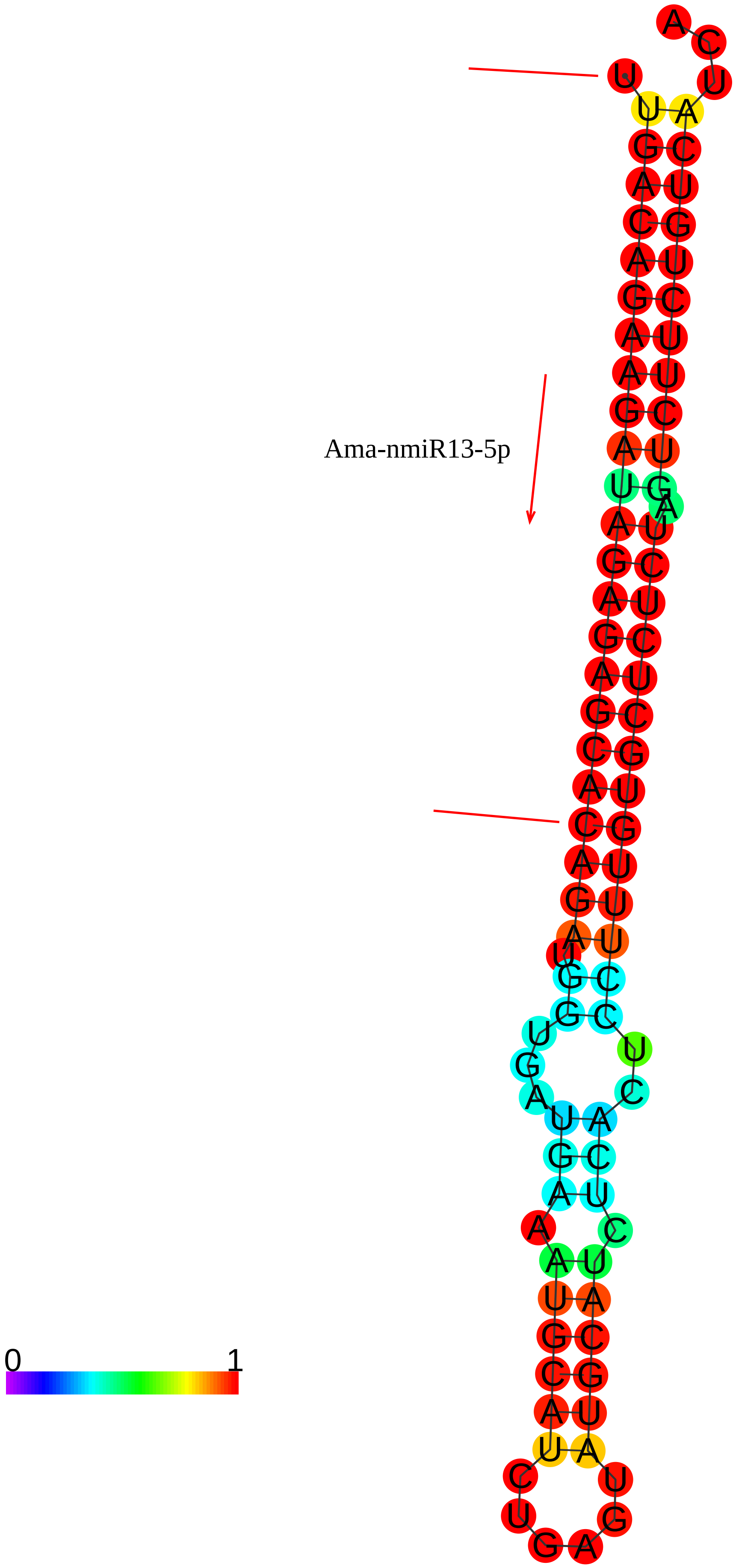
Ama-nmiR13-5p
0
1

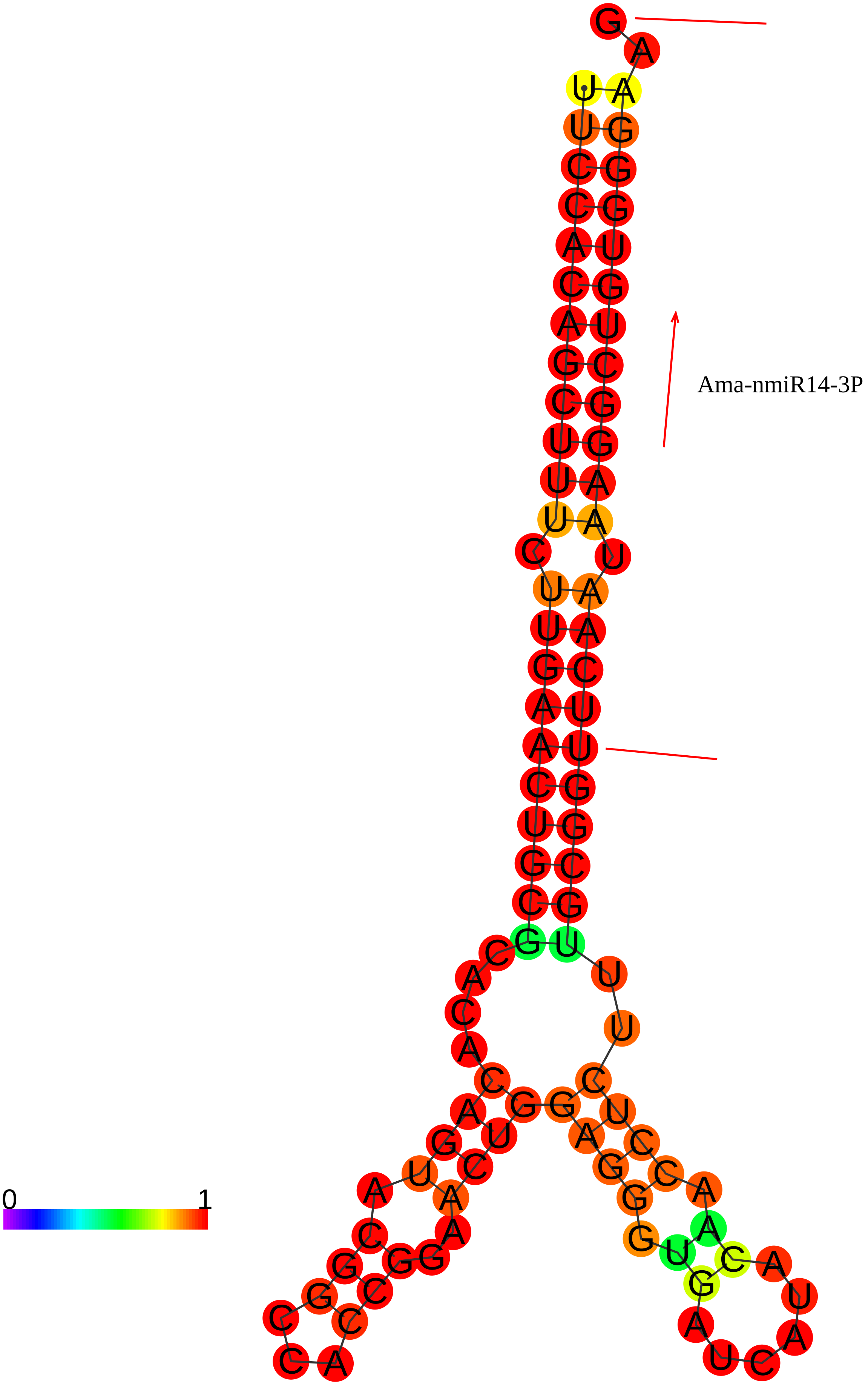
Ama-nmiR14-3P
0
1

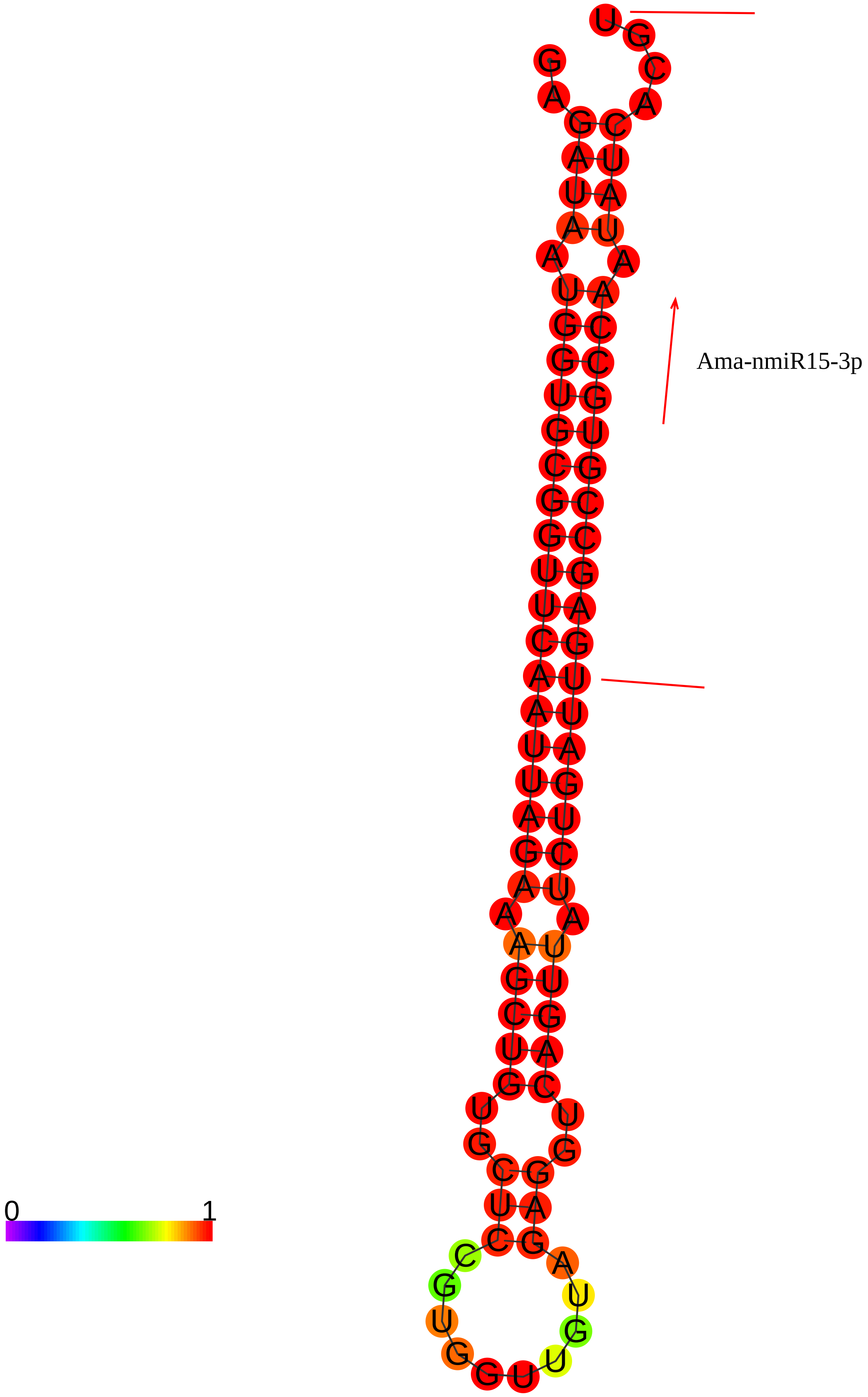
Ama-nmiR15-3p
0
1

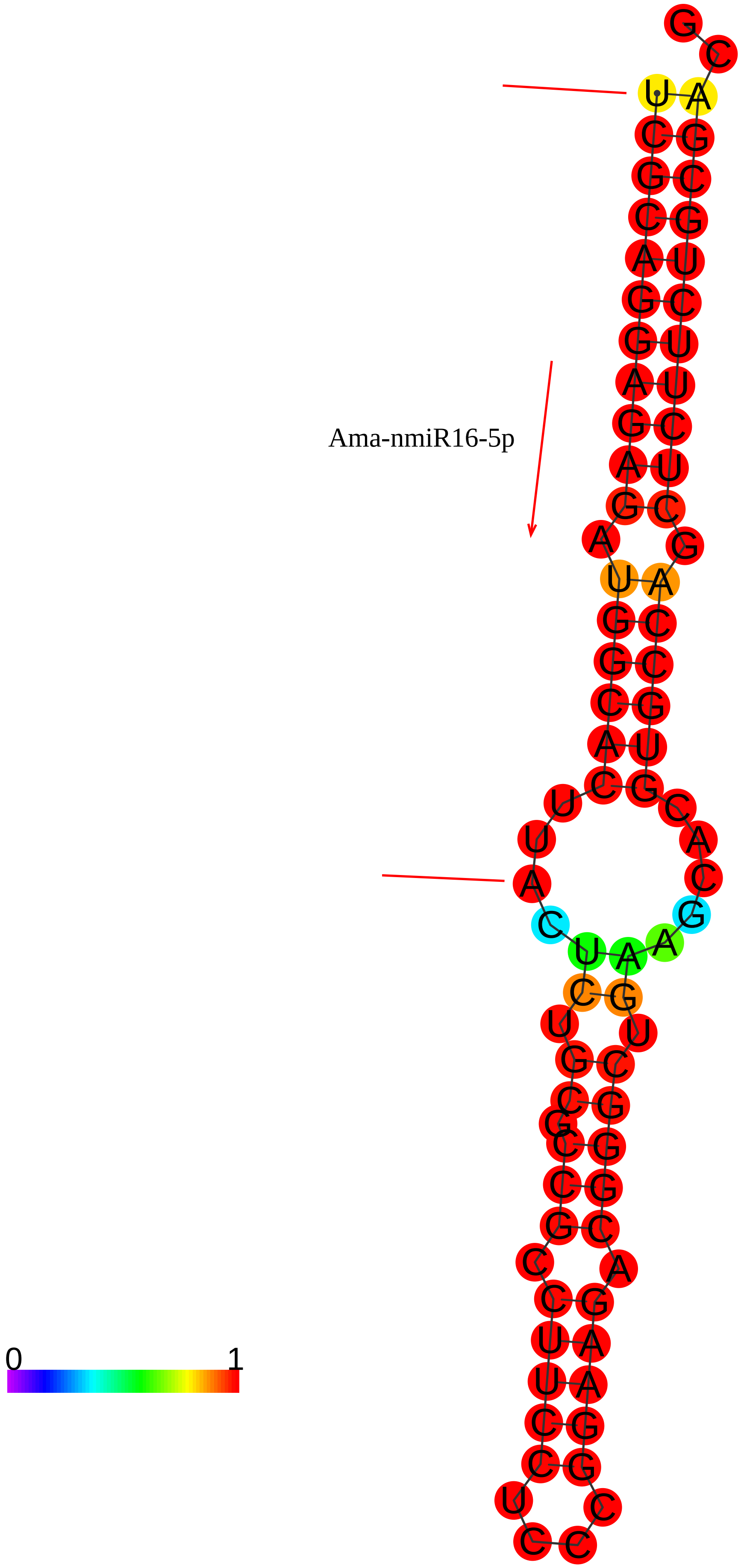
Ama-nmiR16-5p
0
1

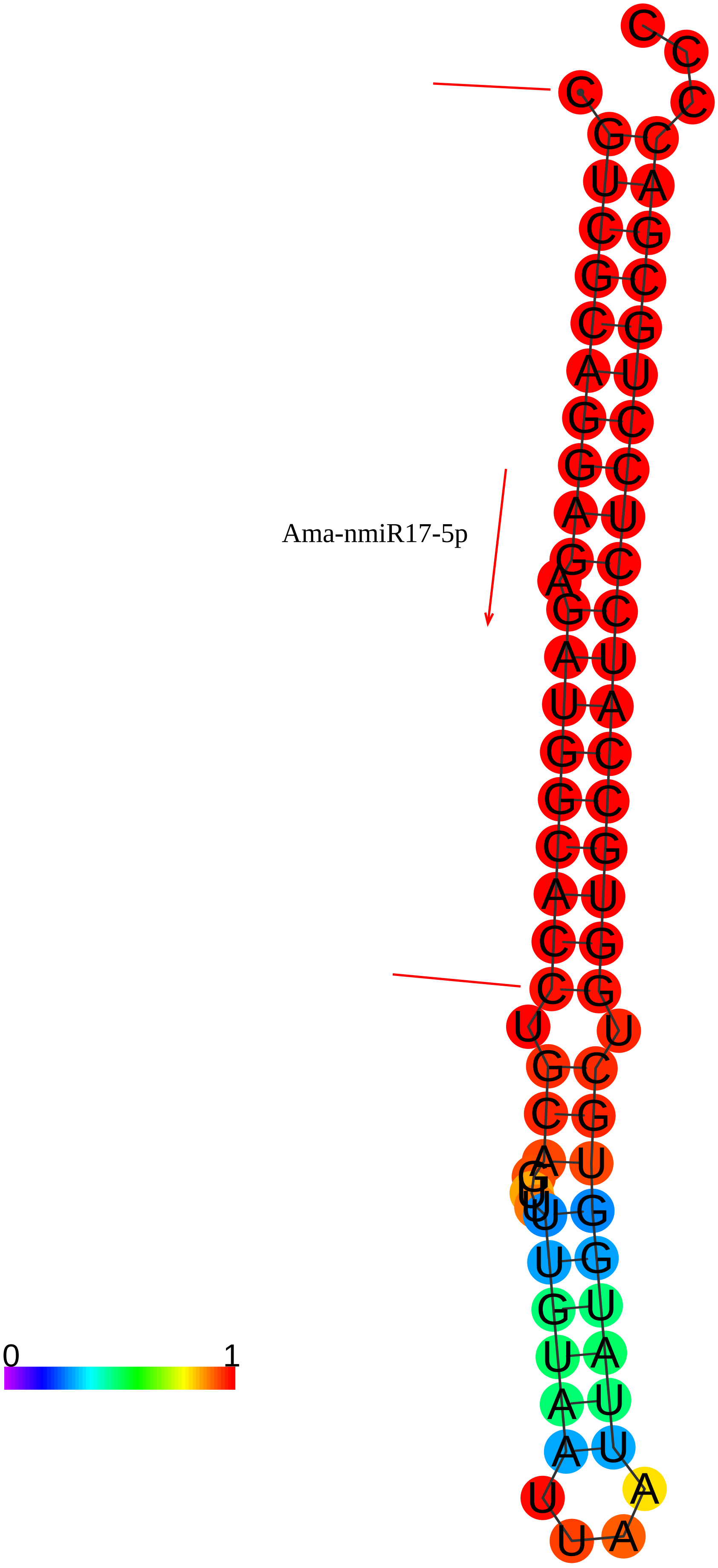
Ama-nmiR17-5p
0
1

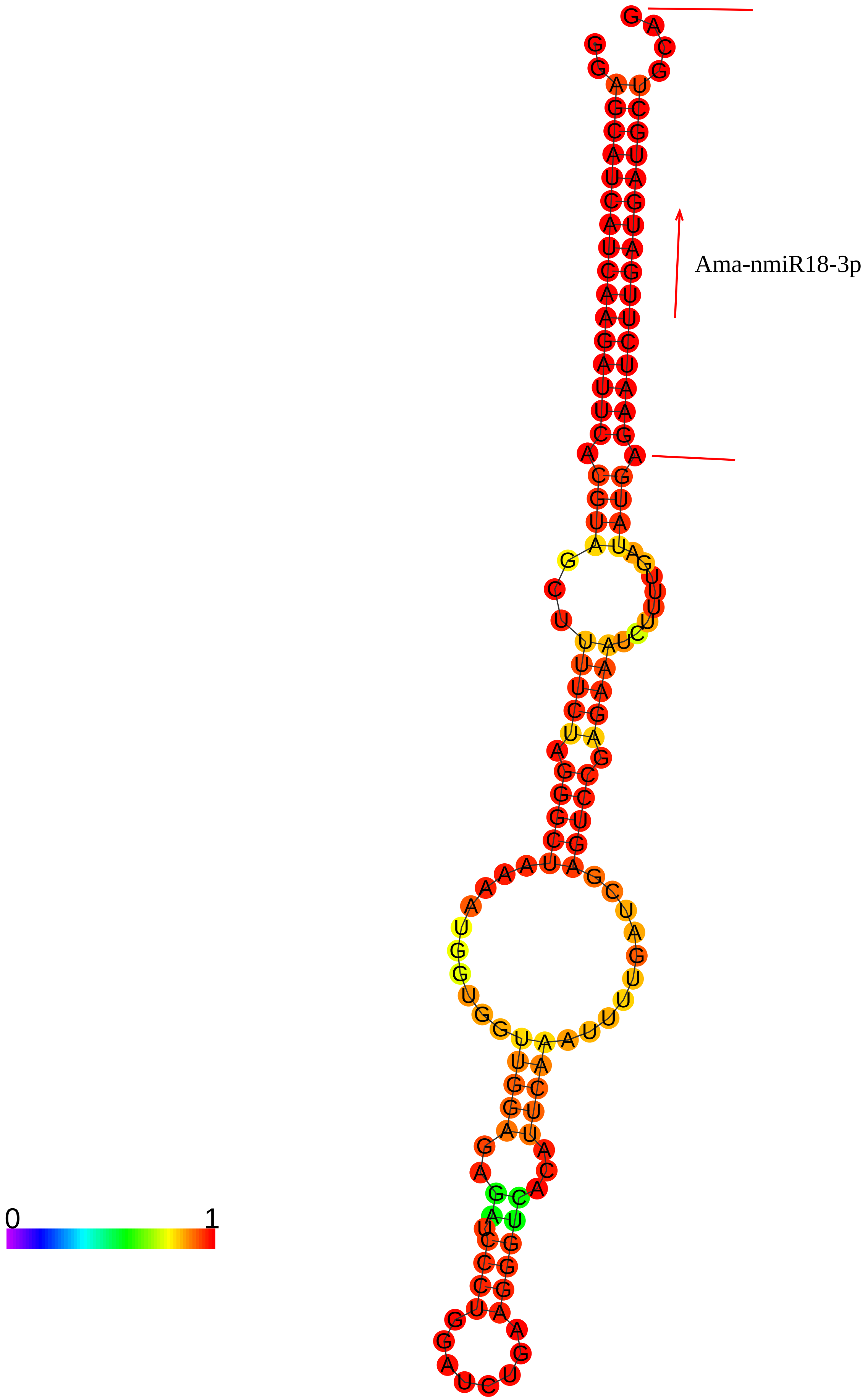
Ama-nmiR18-3p
0
1

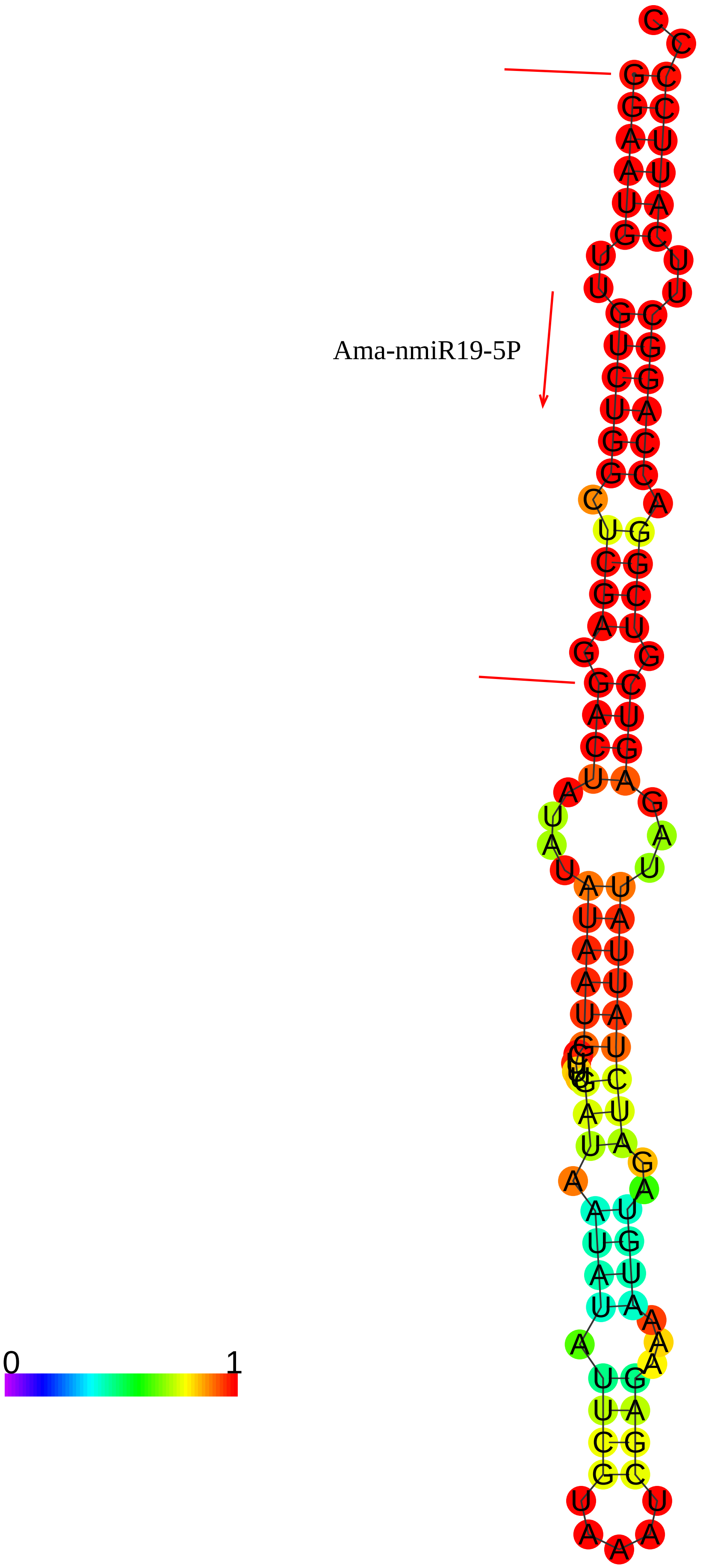

Ama-nmiR19-5P
0
1

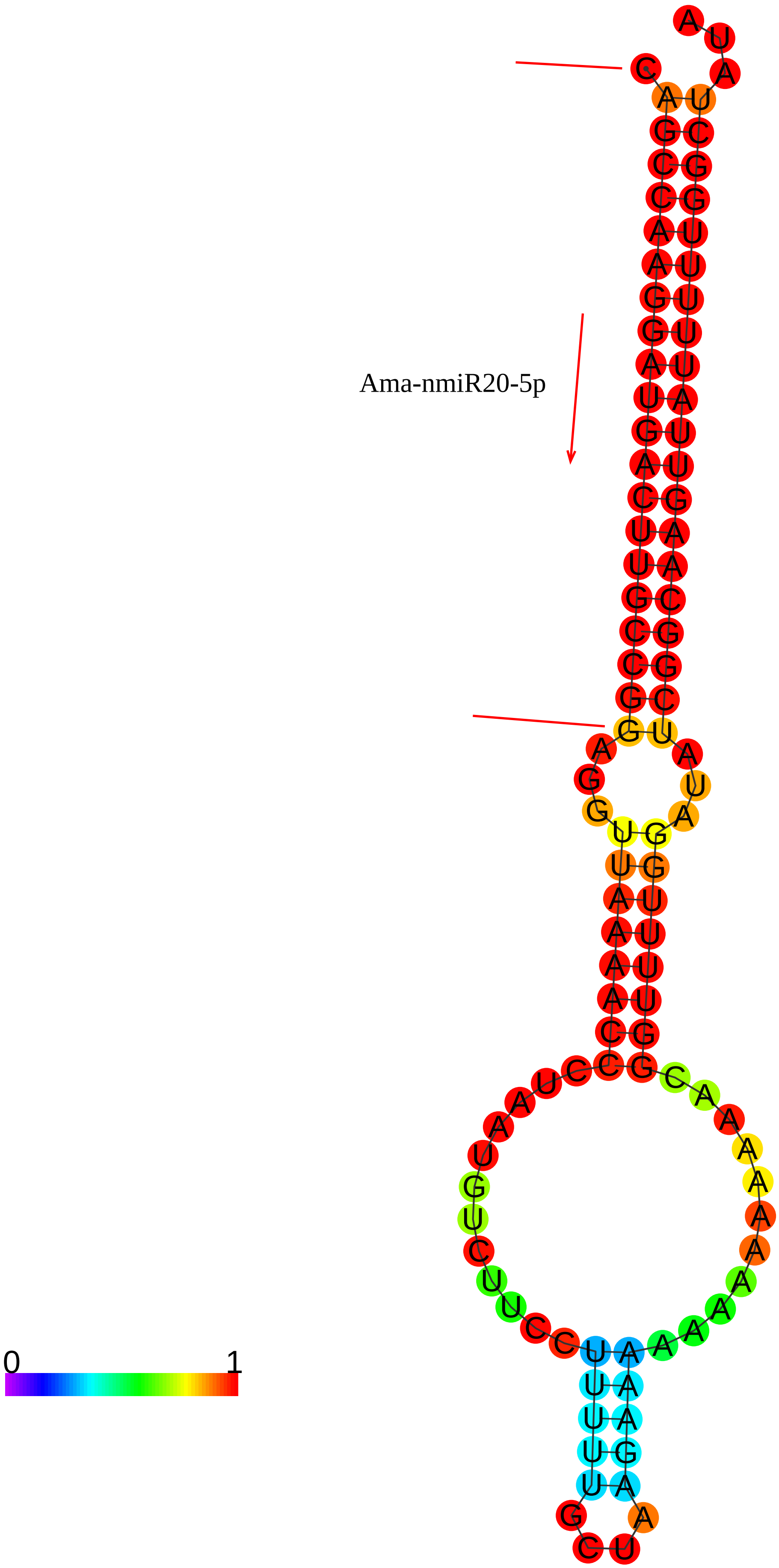

Ama-nmiR20-5p
0
1

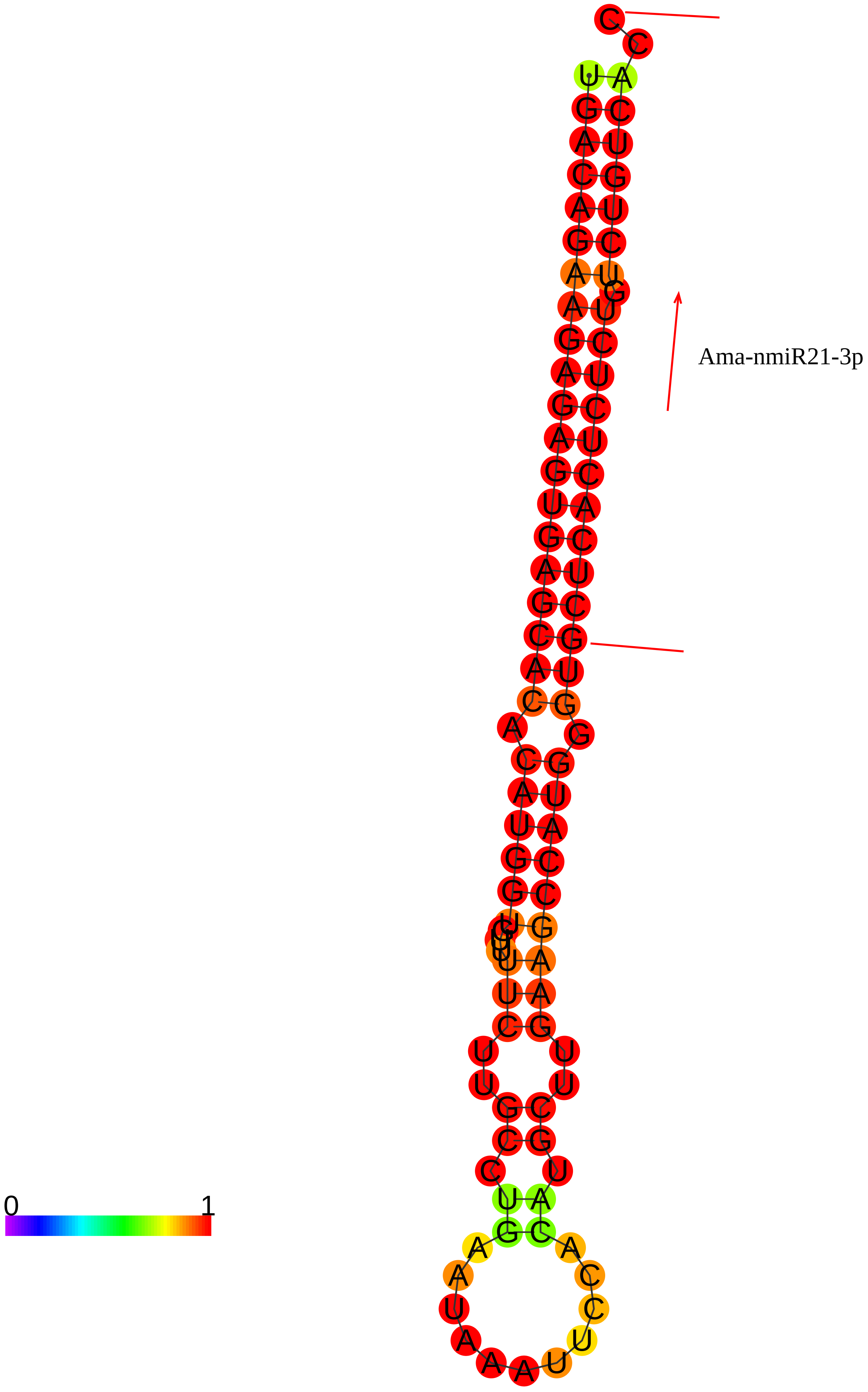
Ama-nmiR21-3p
0
1

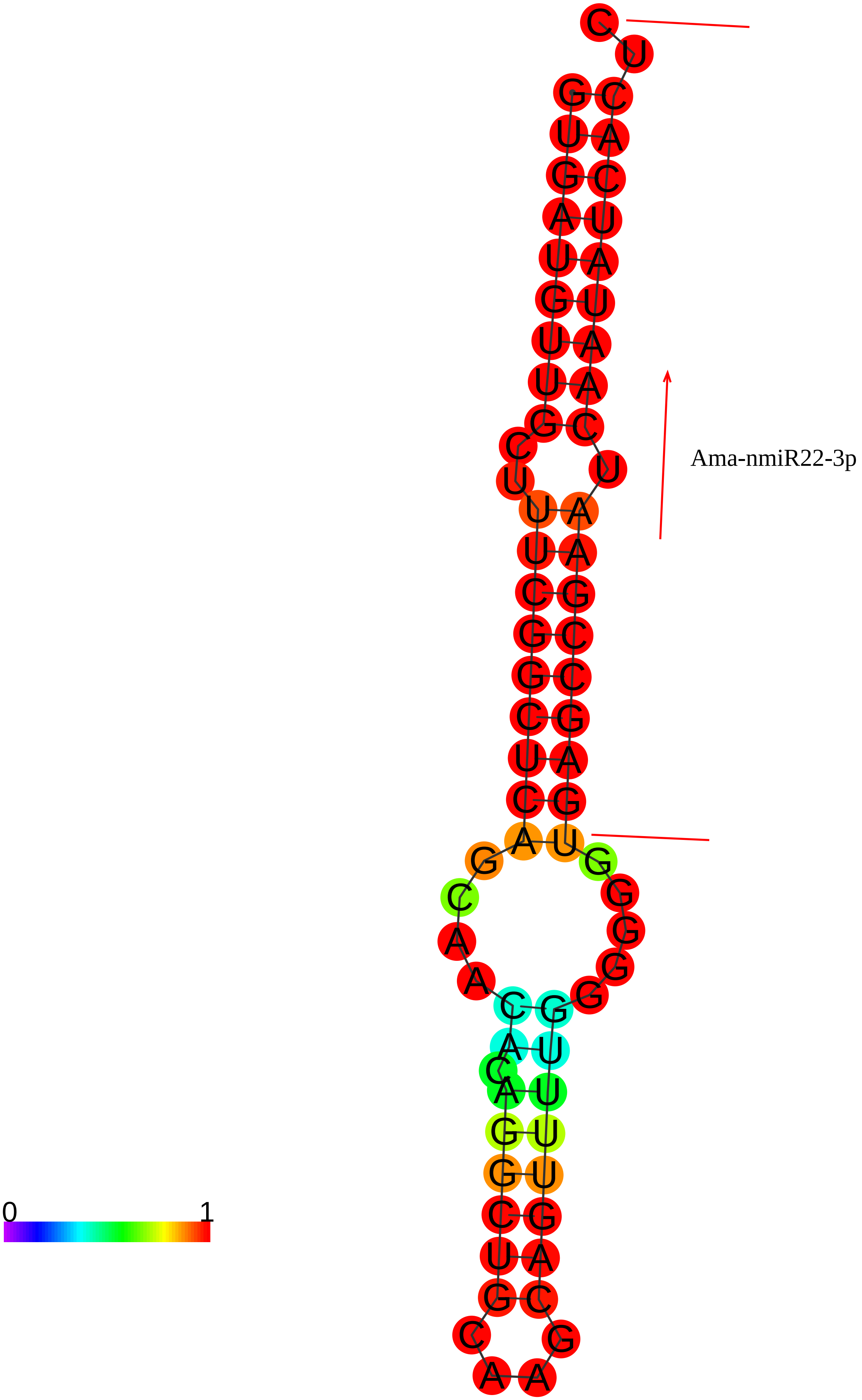
Ama-nmiR22-3p
0
1

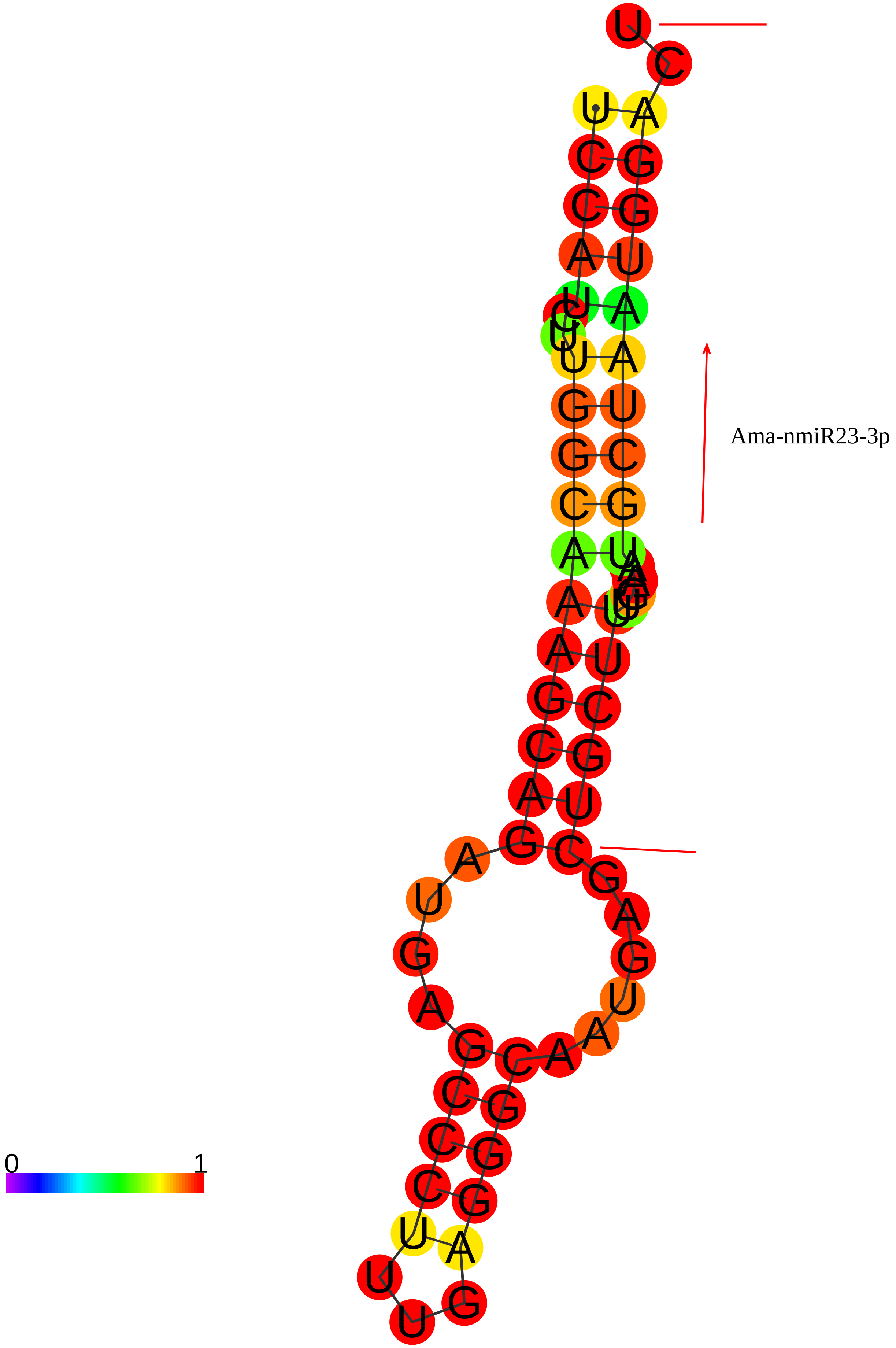
Ama-nmiR23-3p
0
1

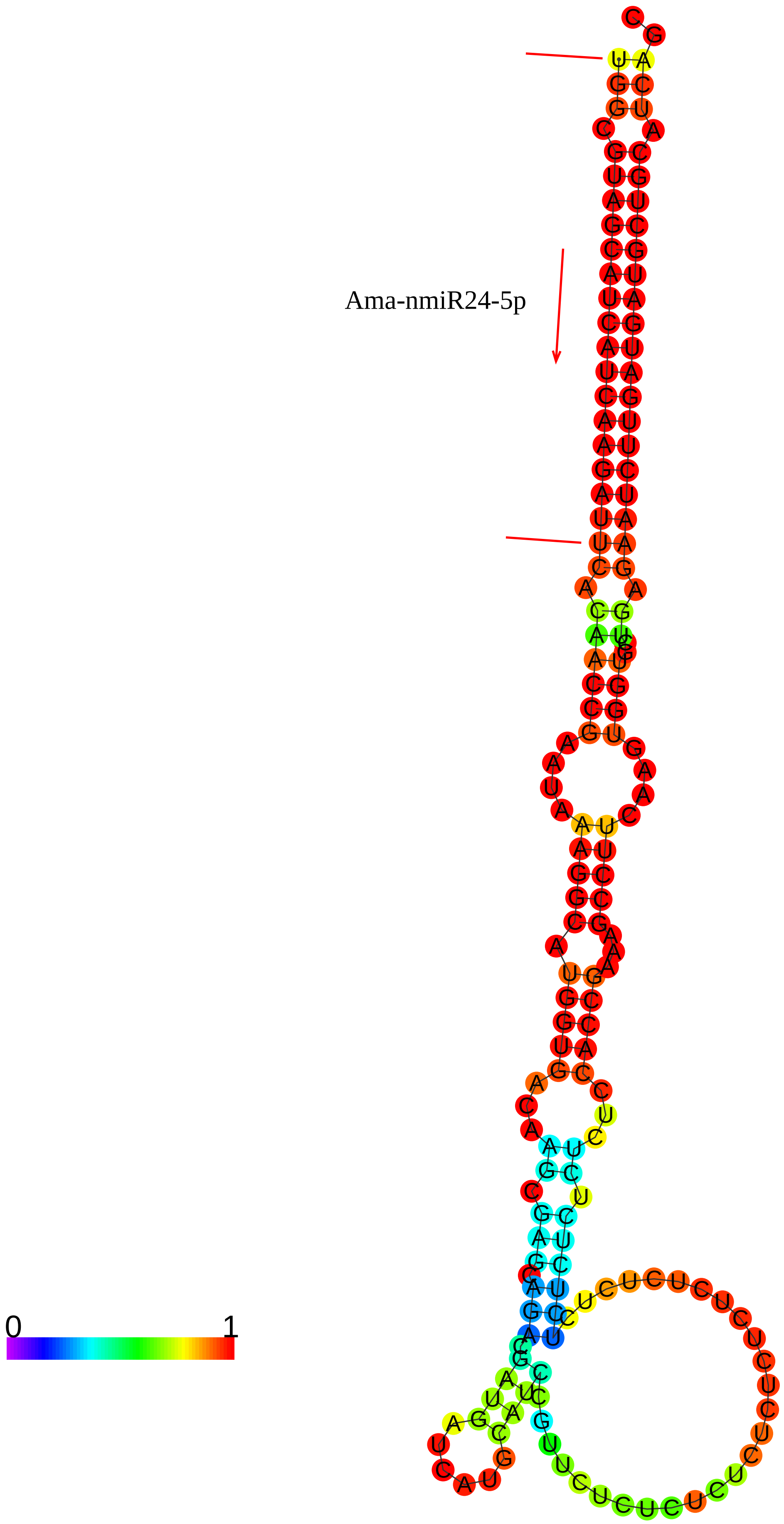
Ama-nmiR24-5p
0
1

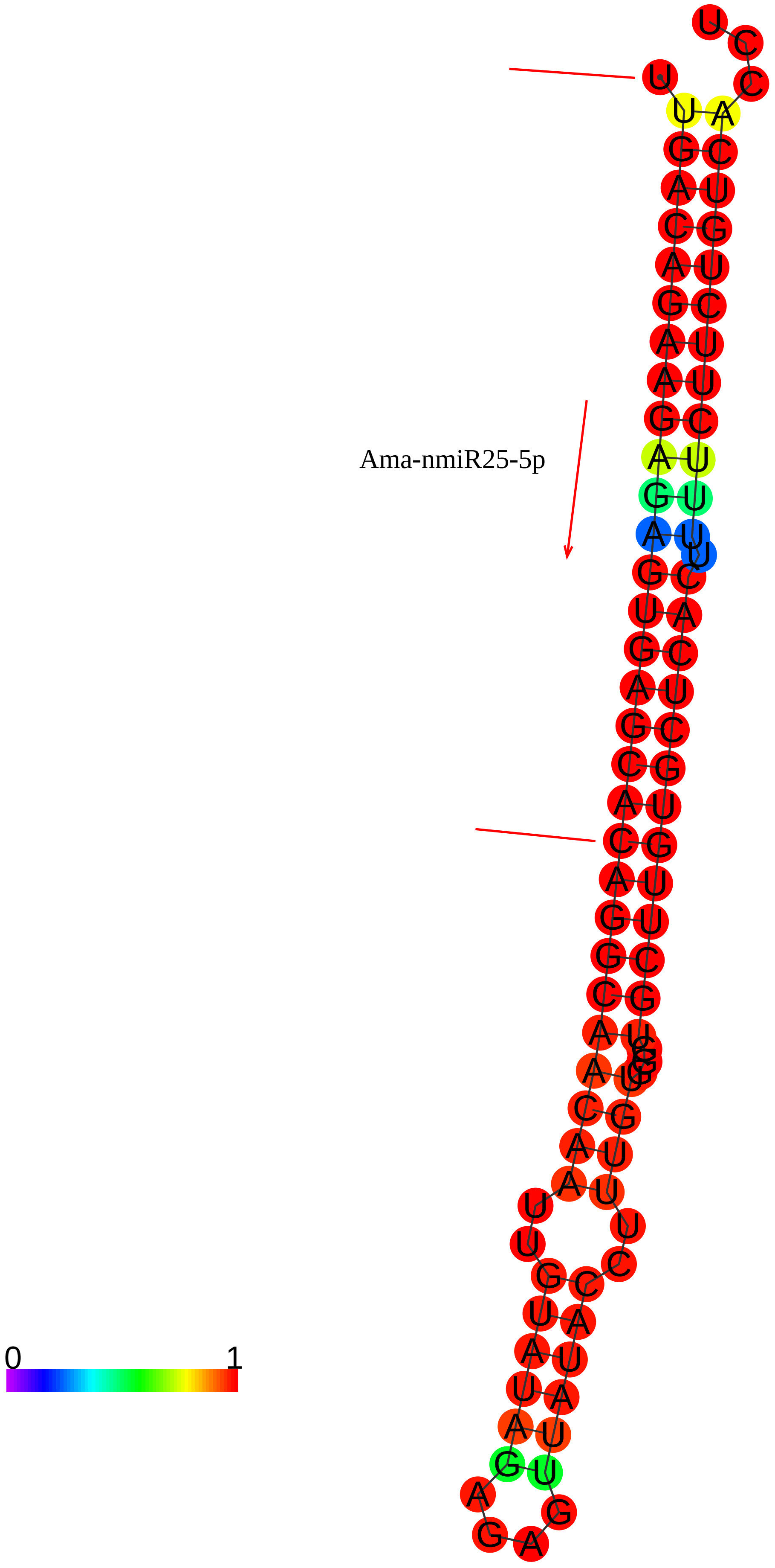
Ama-nmiR25-5p
0
1

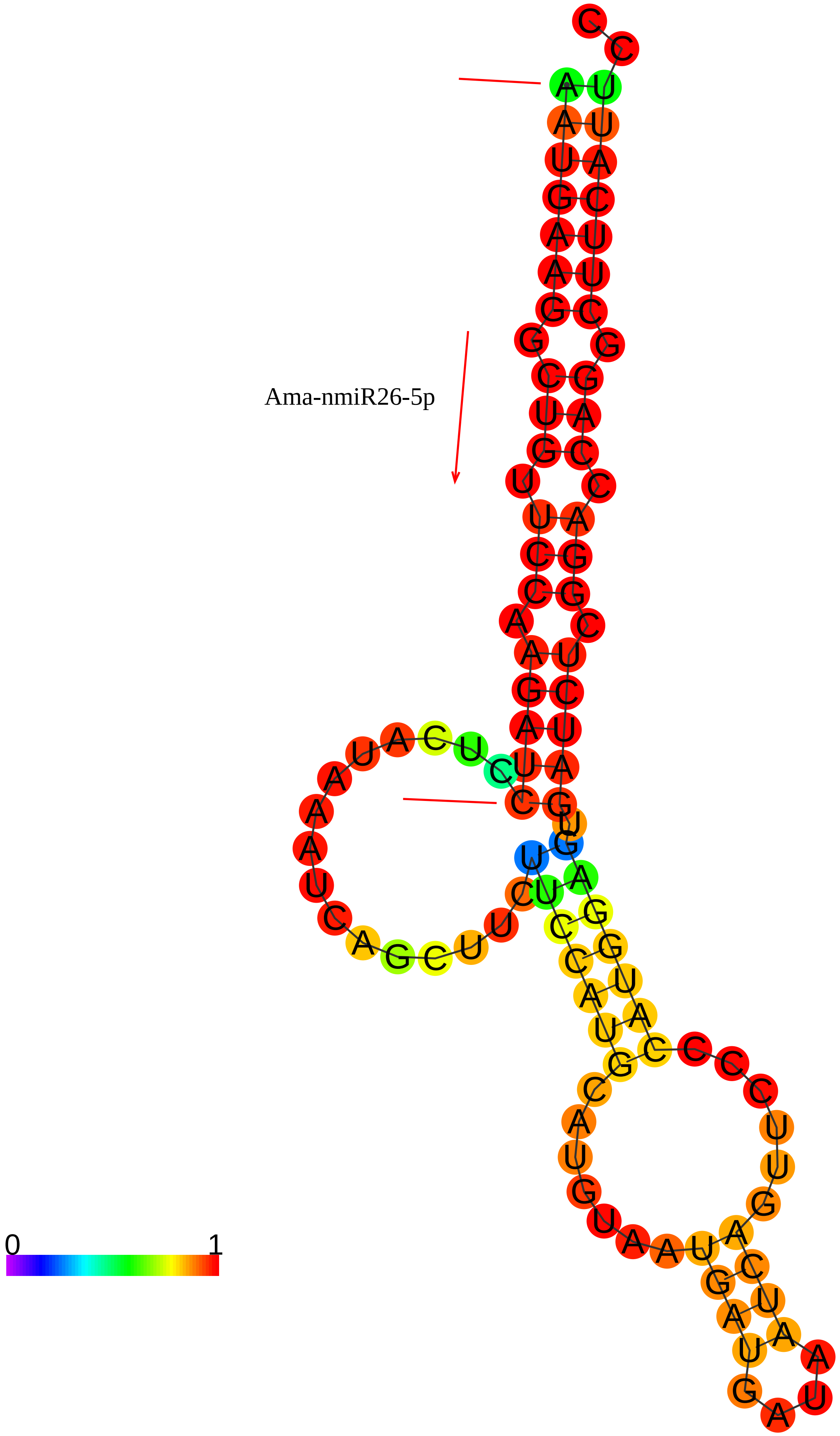
Ama-nmiR26-5p
0
1

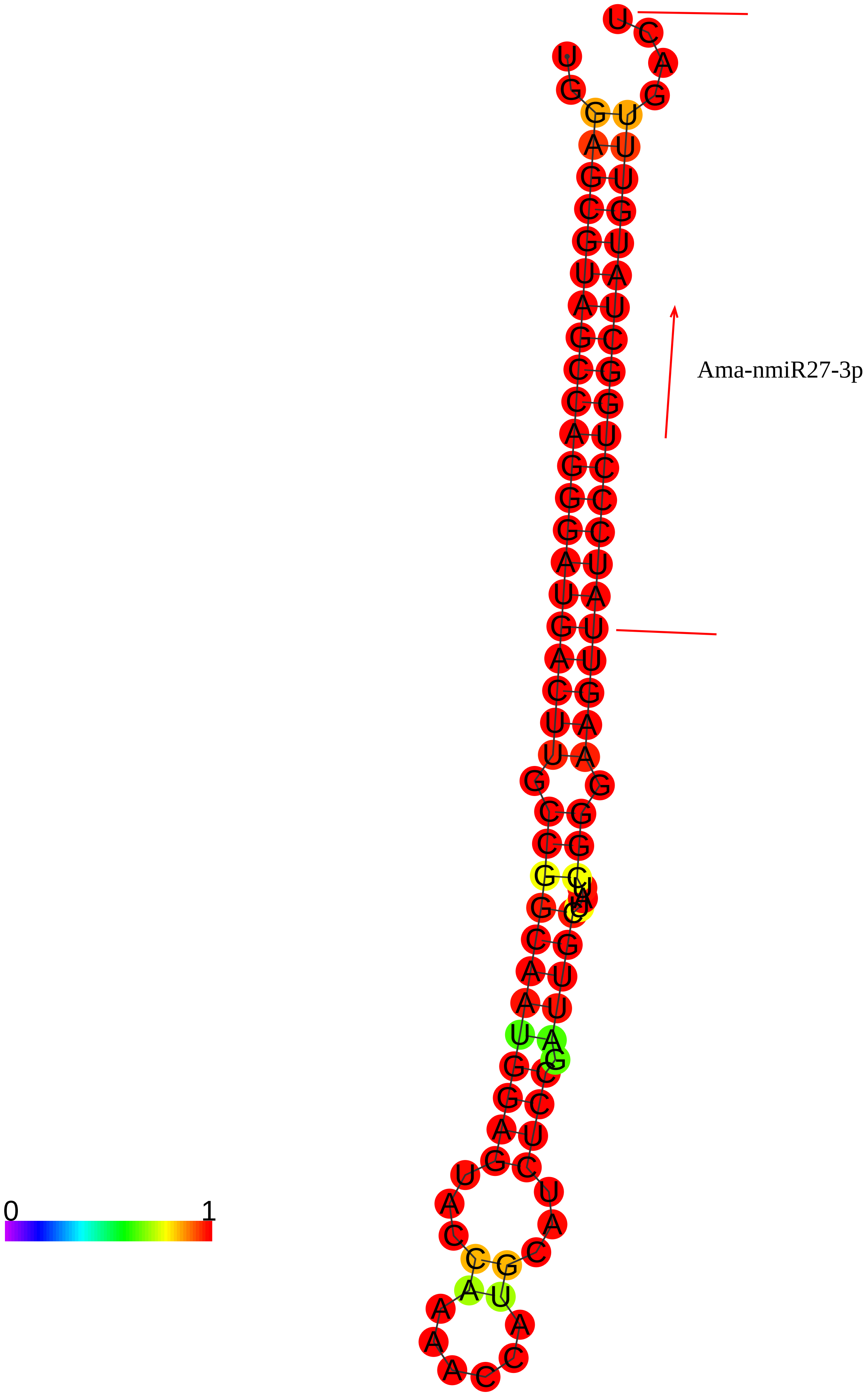
Ama-nmiR27-3p
0
1

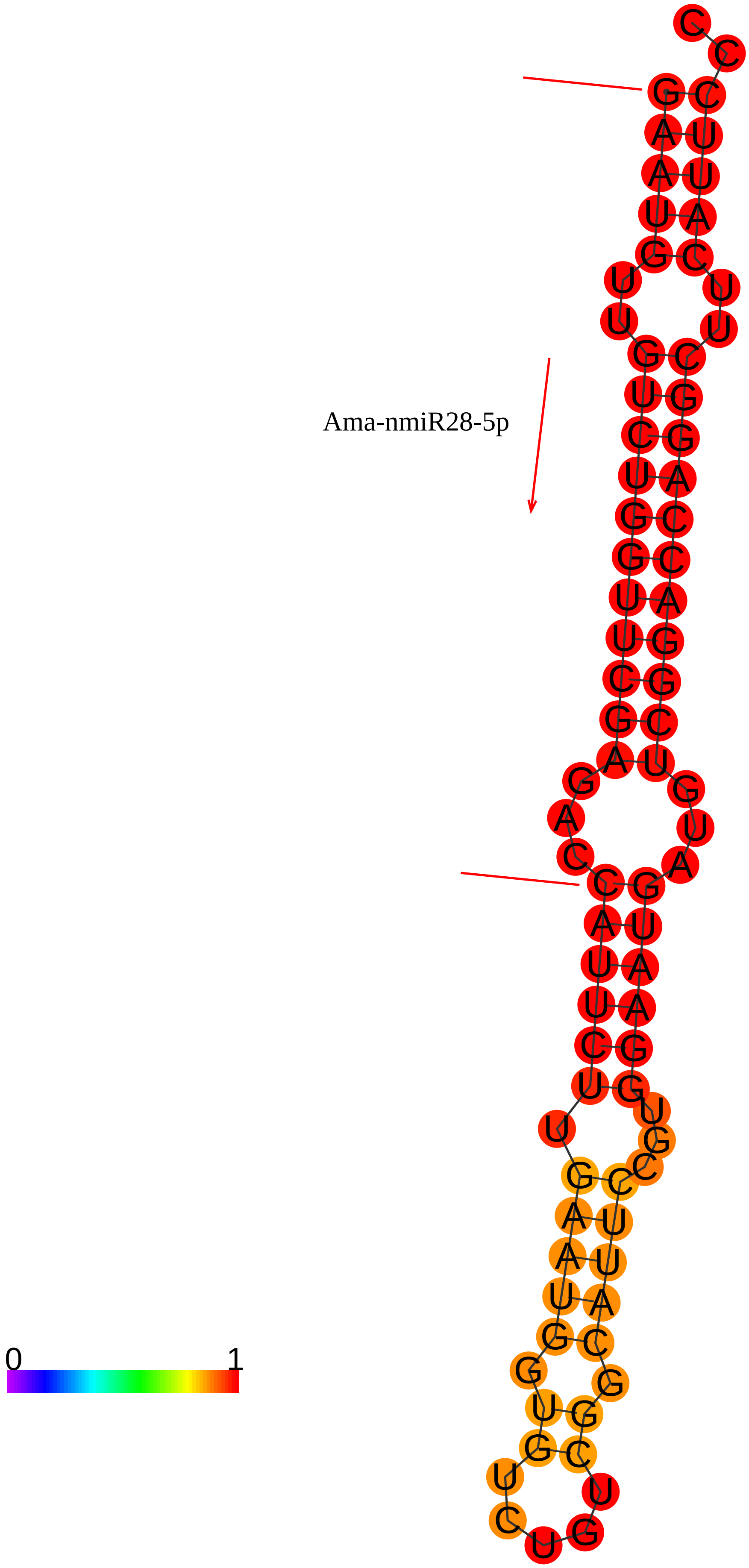
Ama-nmiR28-5p
0
1

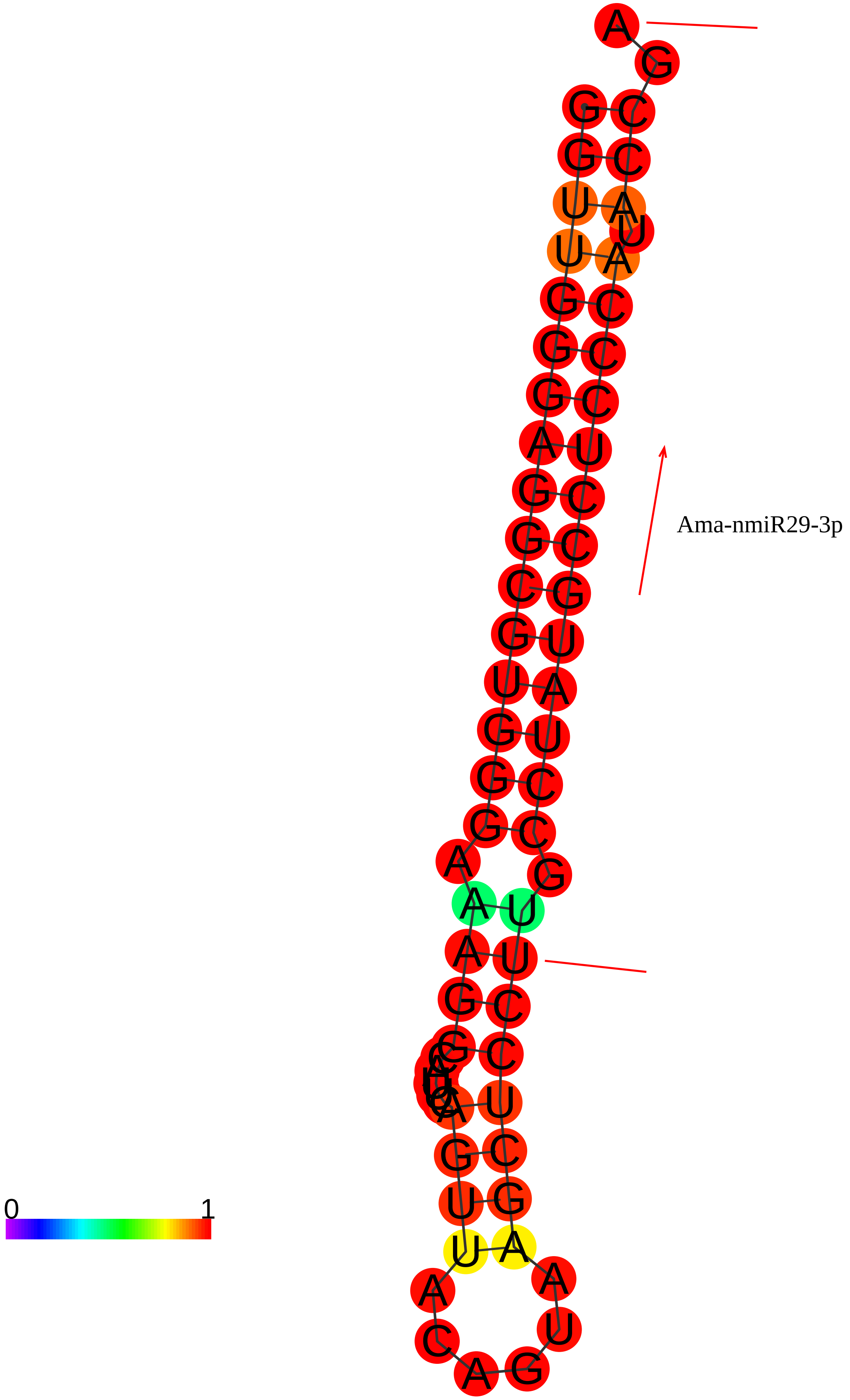
Ama-nmiR29-3p
0
1

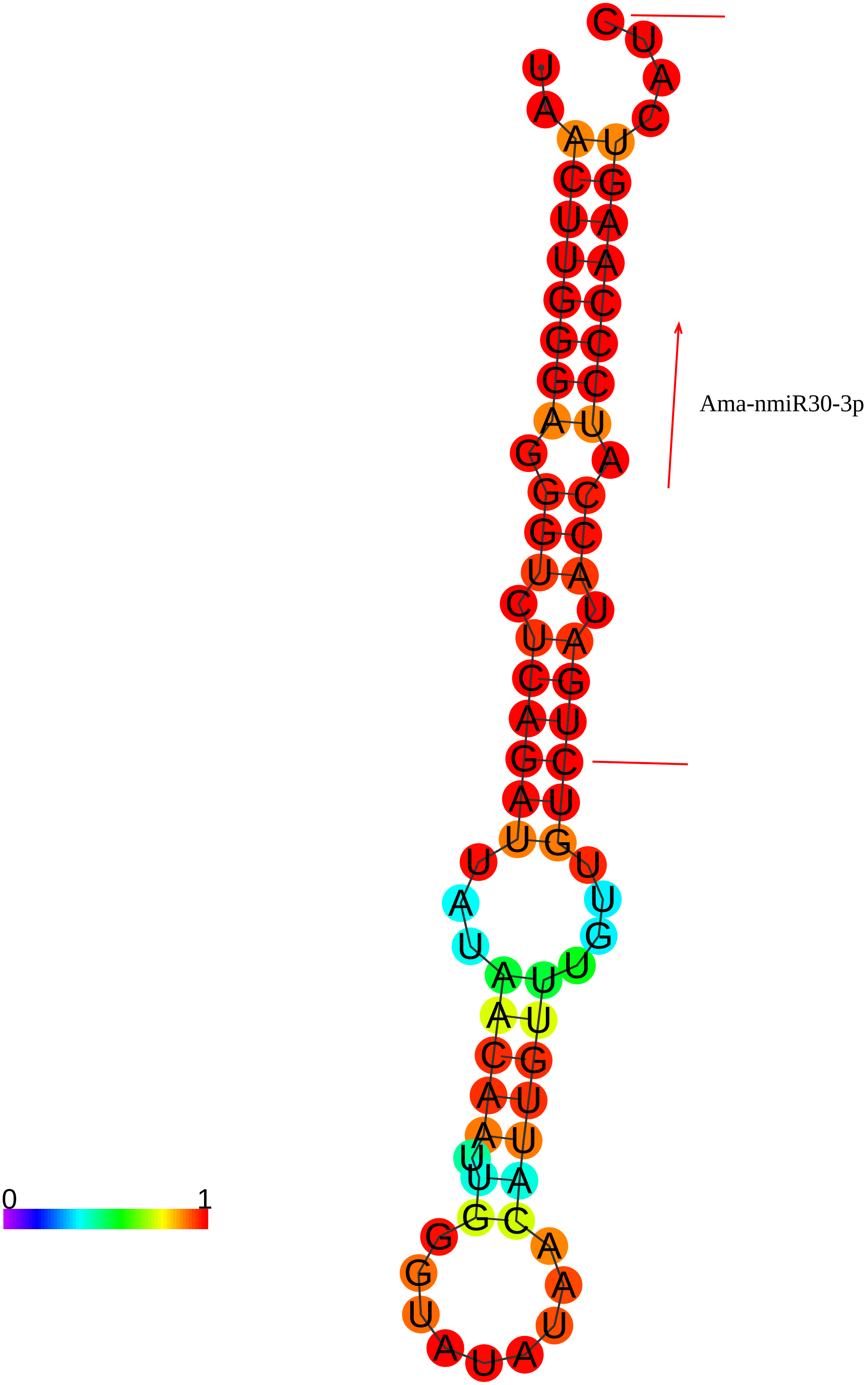
Ama-nmiR30-3p
0
1

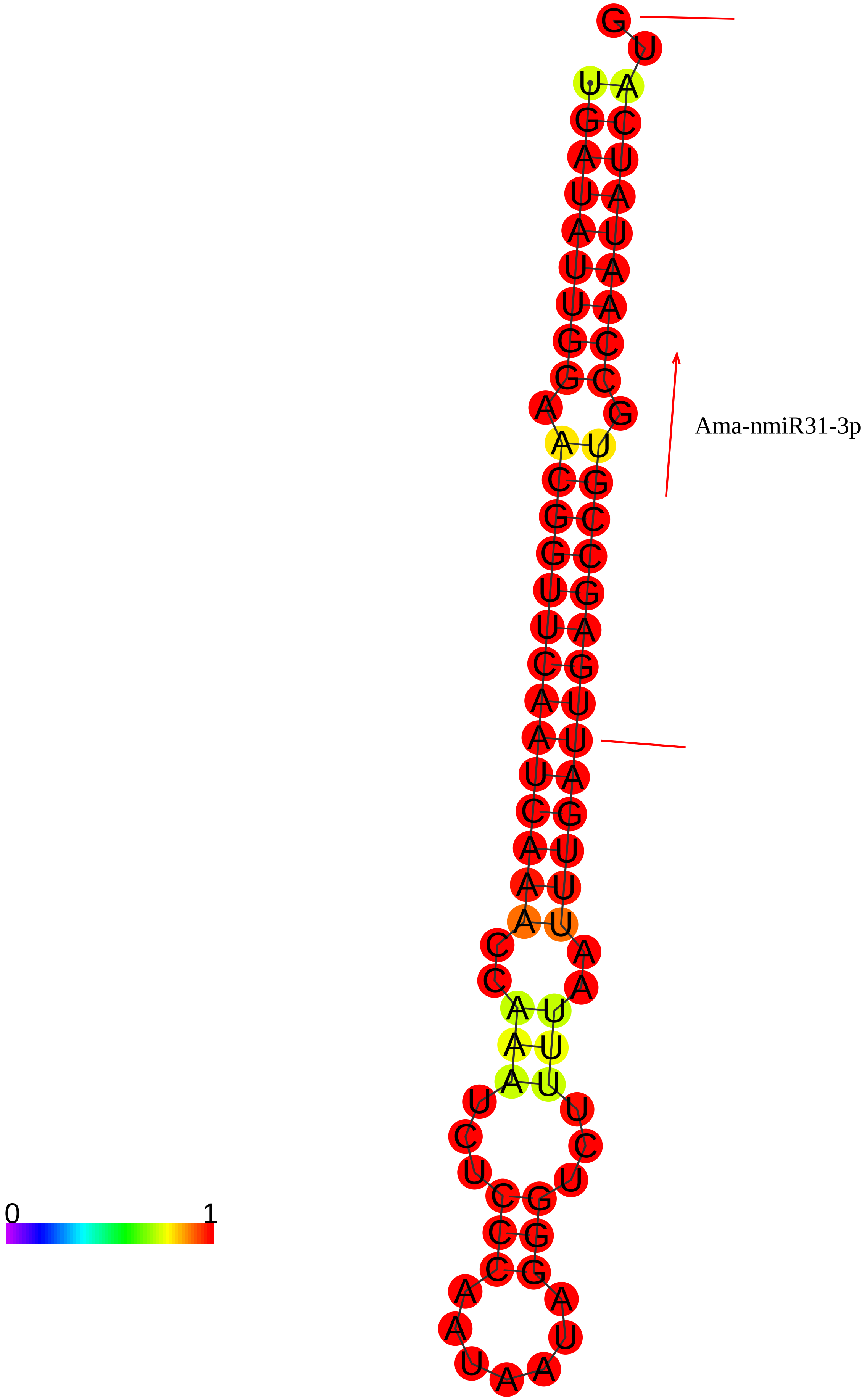
Ama-nmiR31-3p
0
1

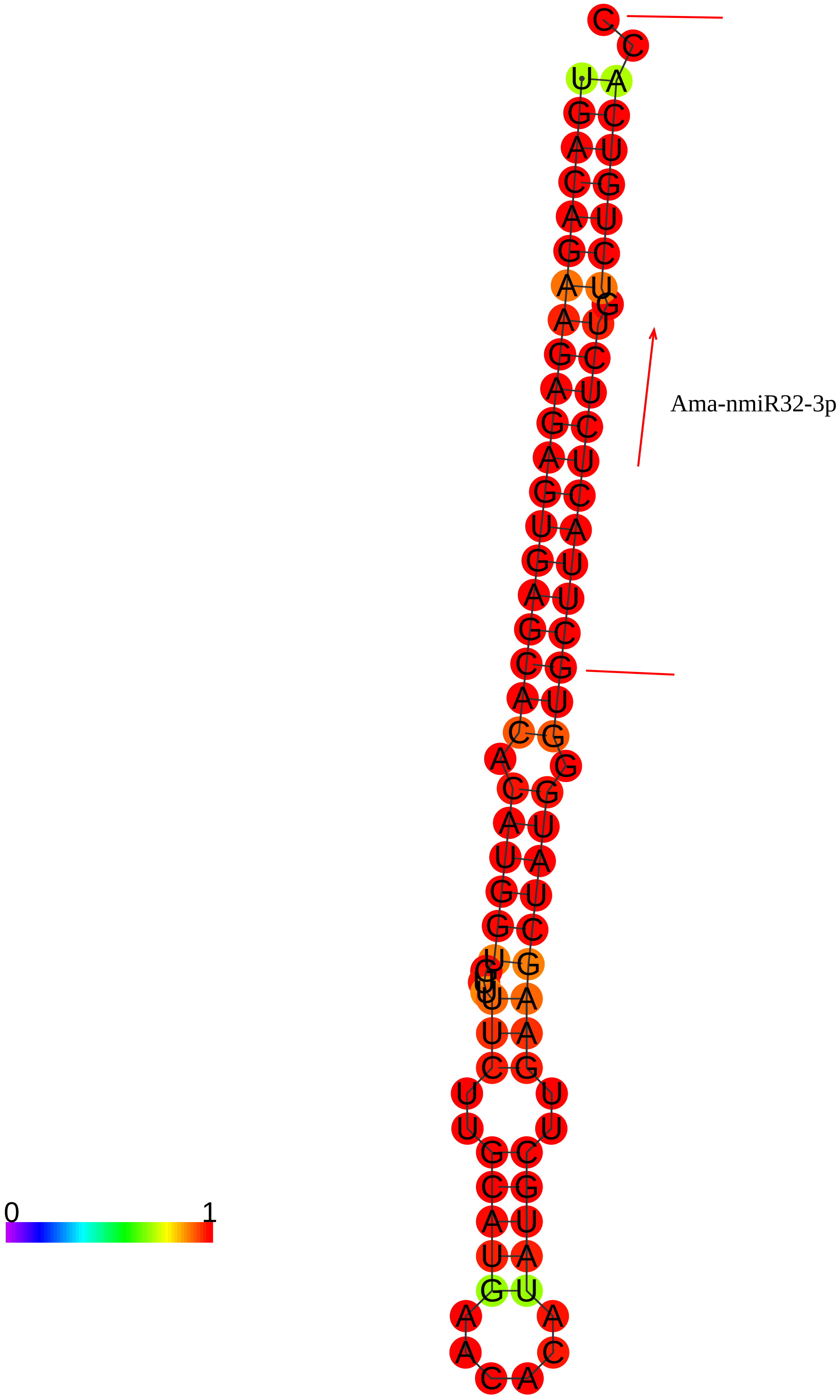

Ama-nmiR32-3p
0
1

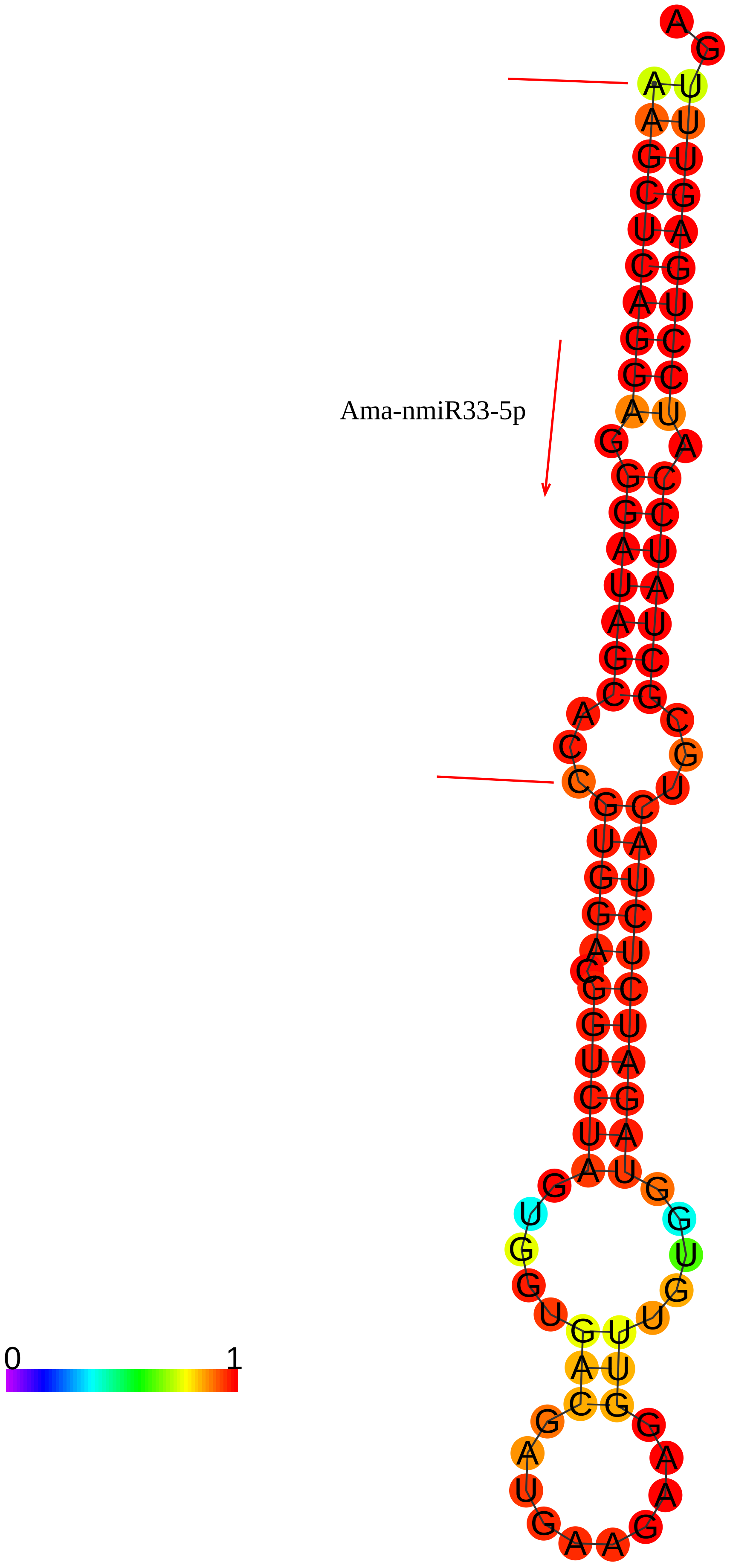
Ama-nmiR33-5p
0
1

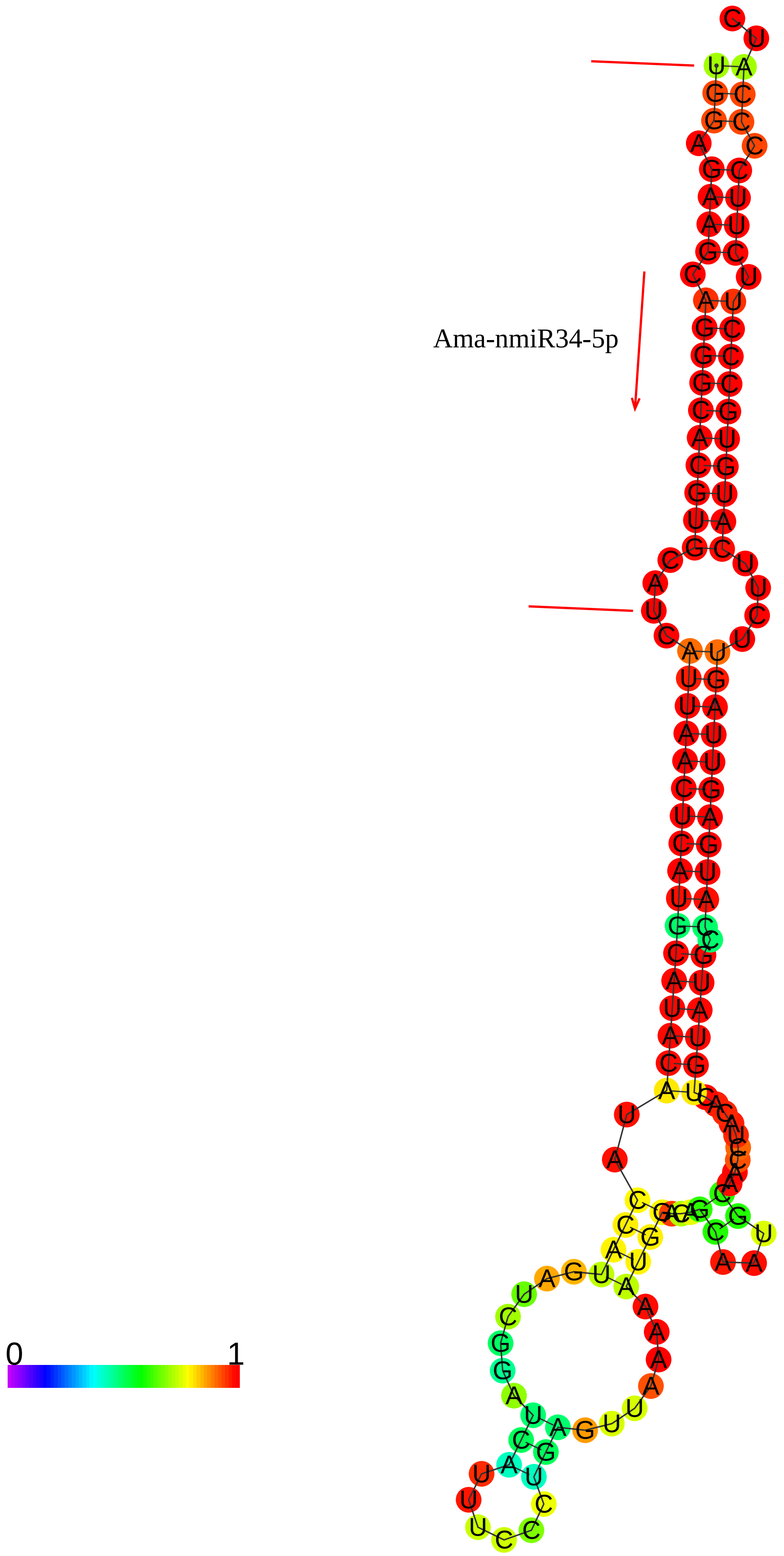
Ama-nmiR34-5p
0
1

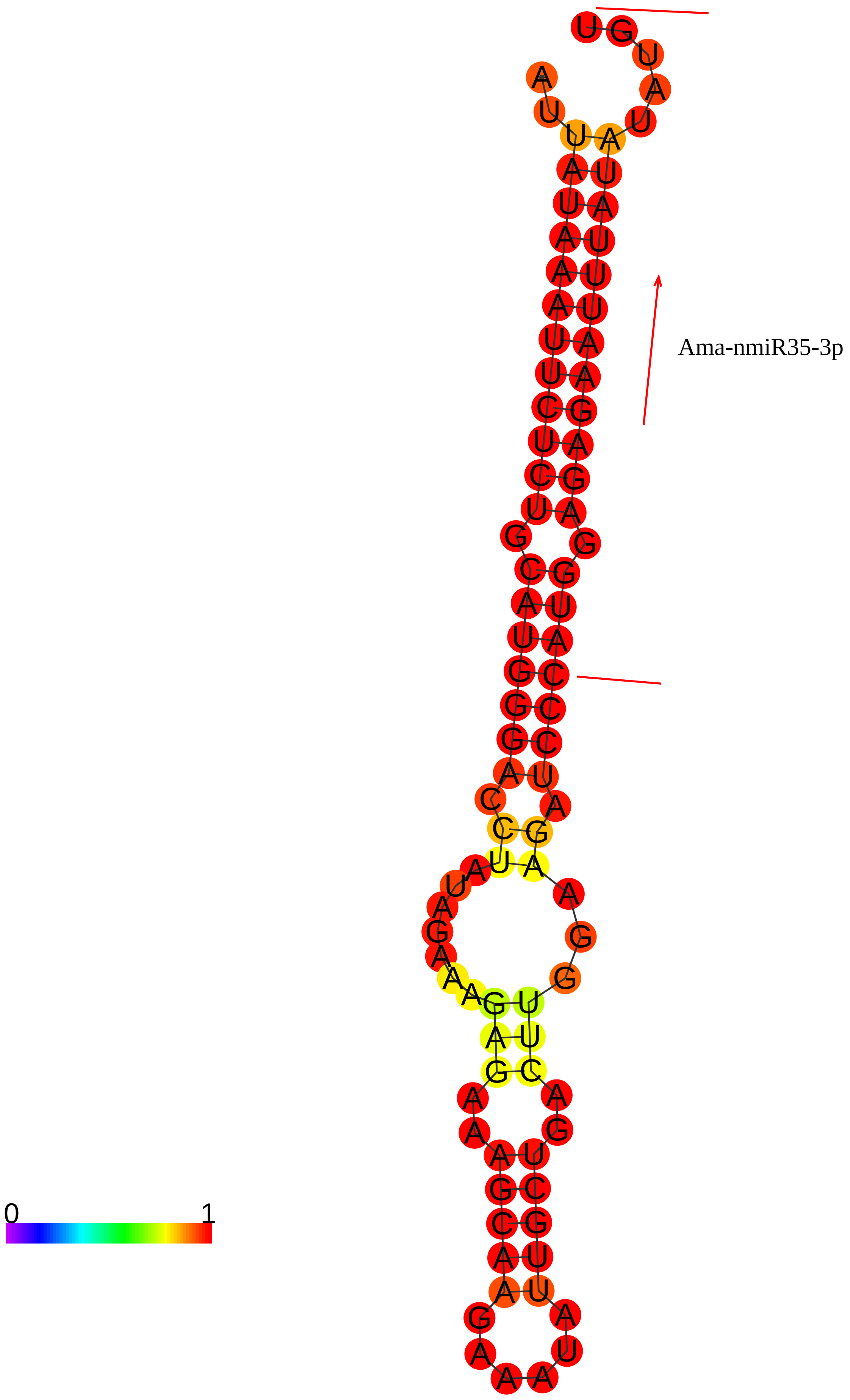
Ama-nmiR35-3p
0
1

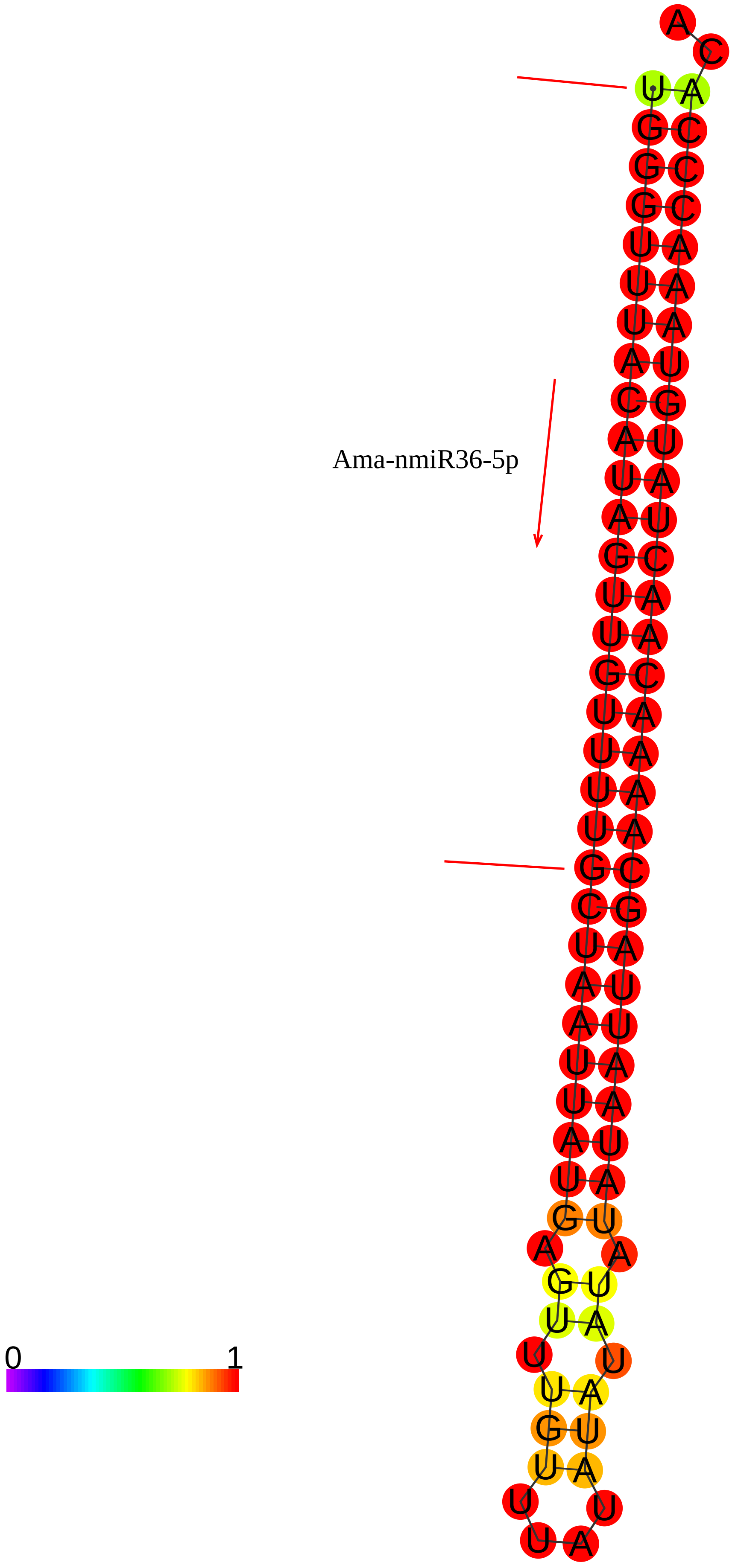
Ama-nmiR36-5p
0
1

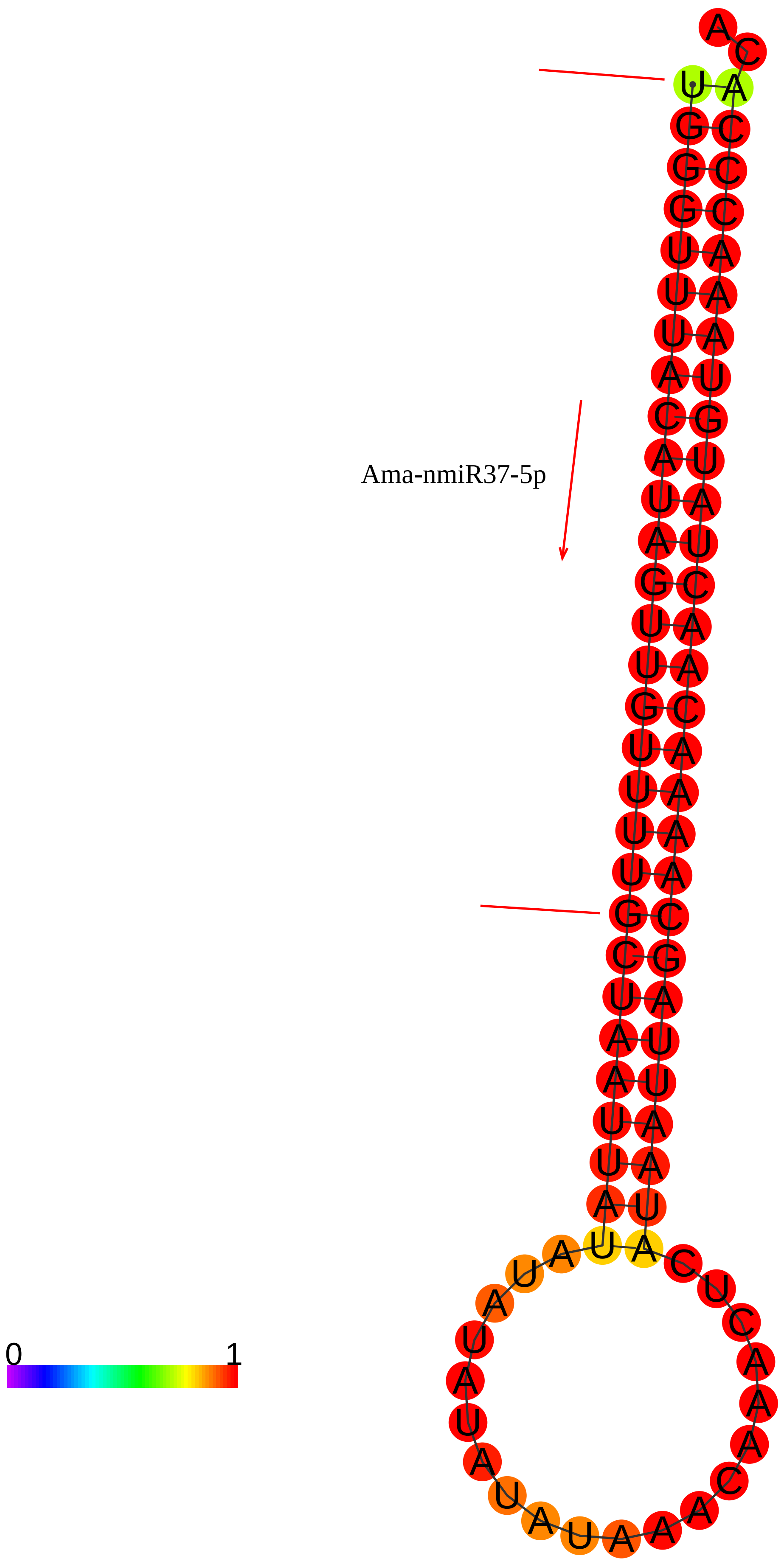
Ama-nmiR37-5p
0
1

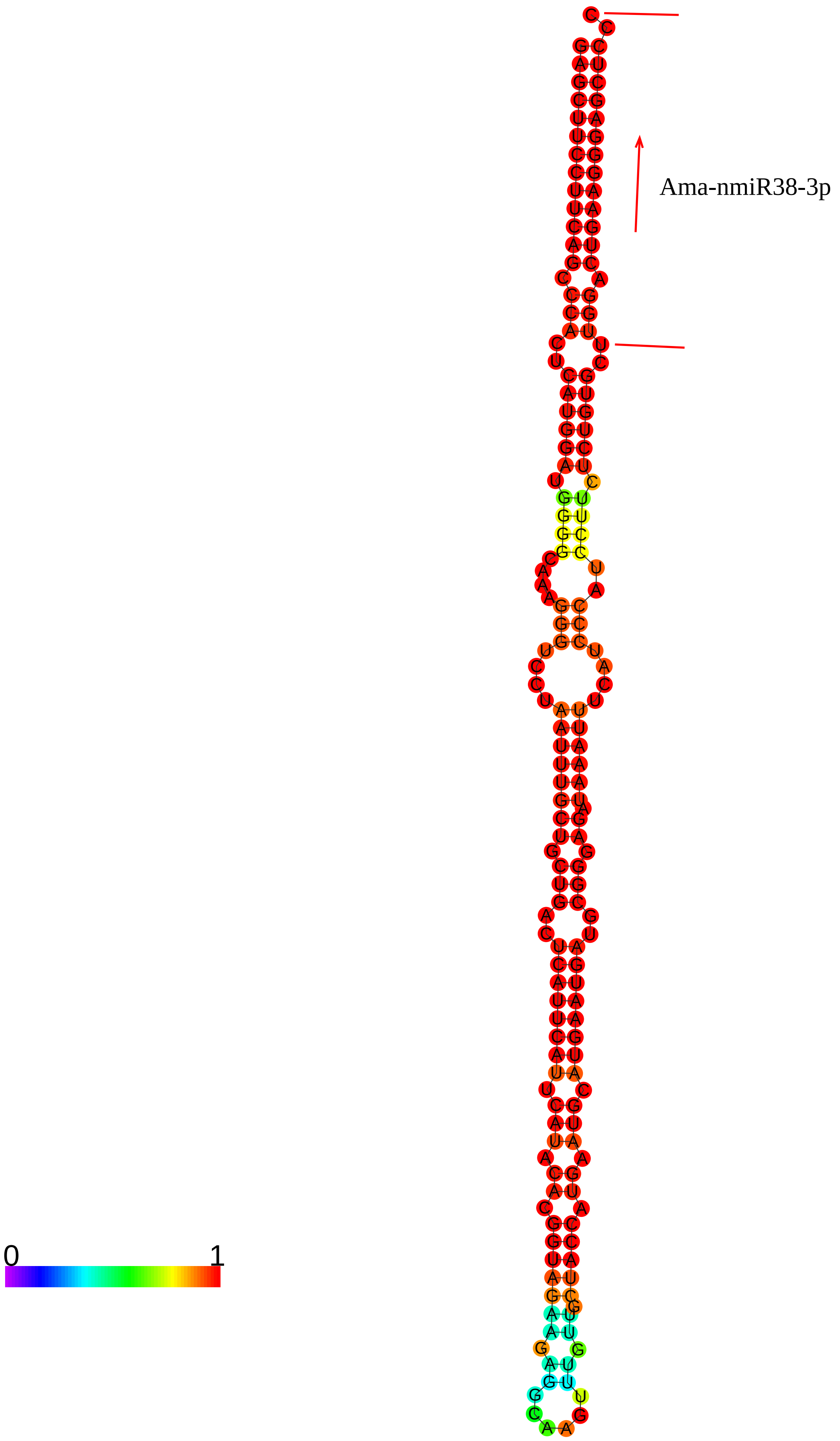
Ama-nmiR38-3p
0
1

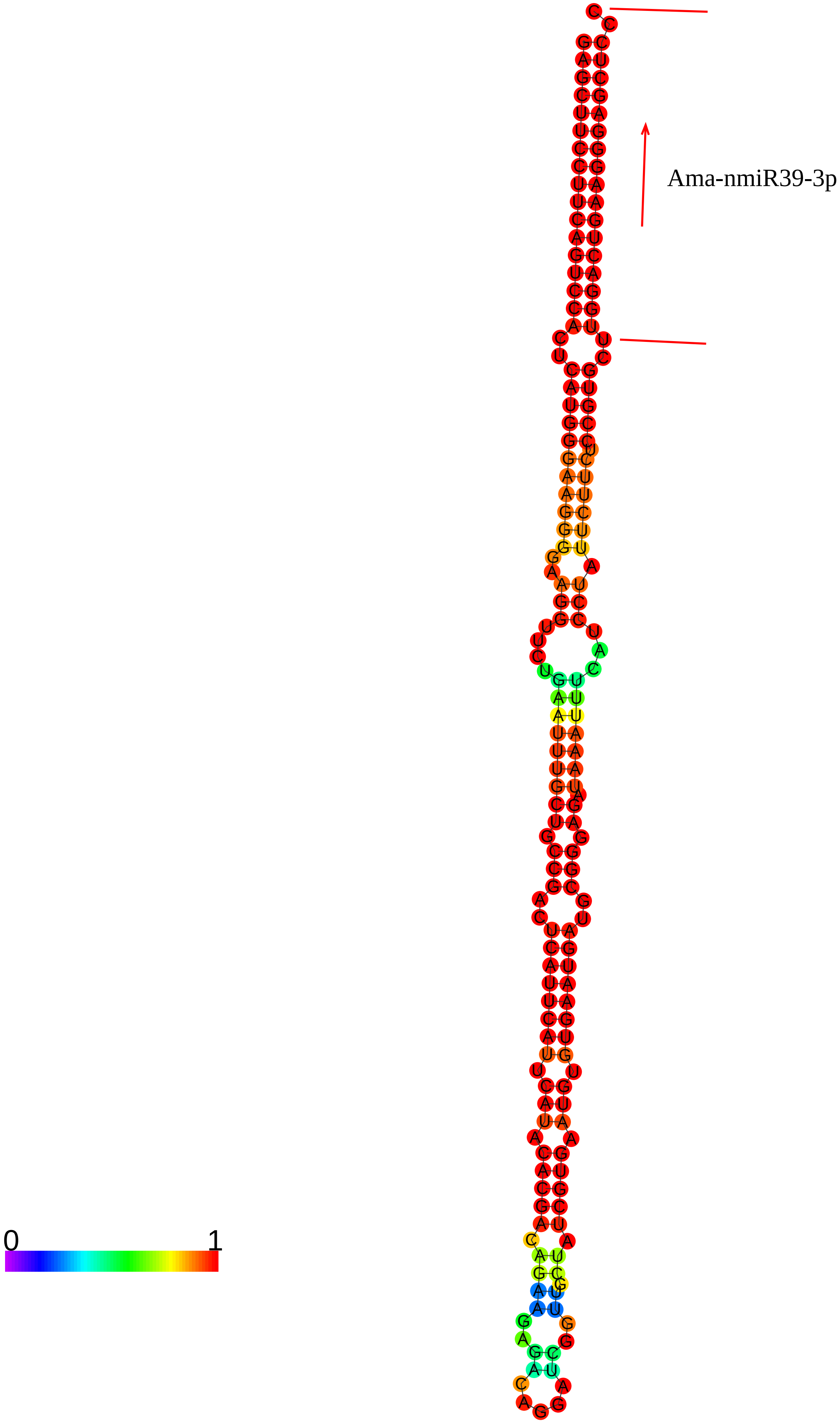

Ama-nmiR39-3p
0
1

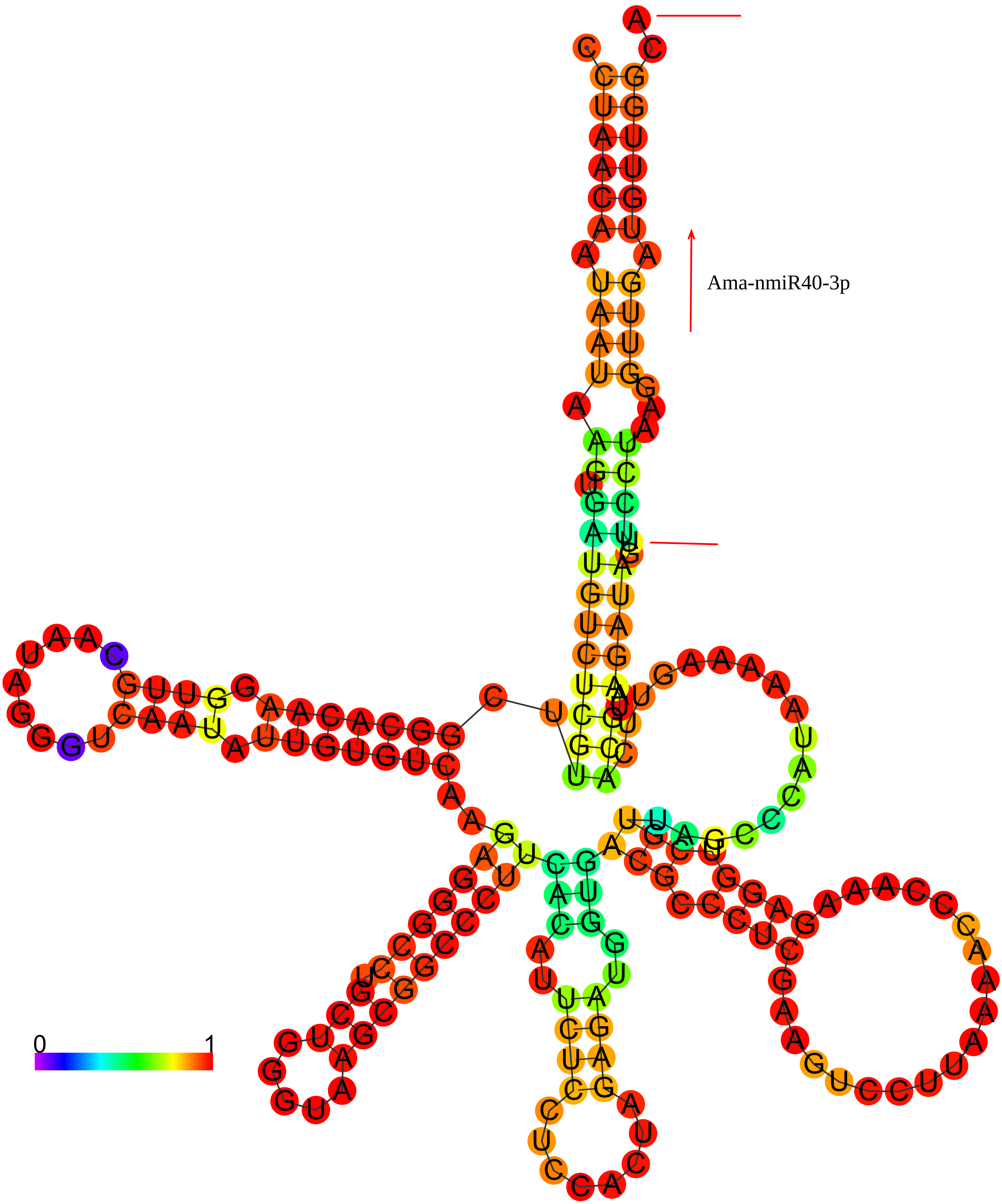
Ama-nmiR40-3p
0
1

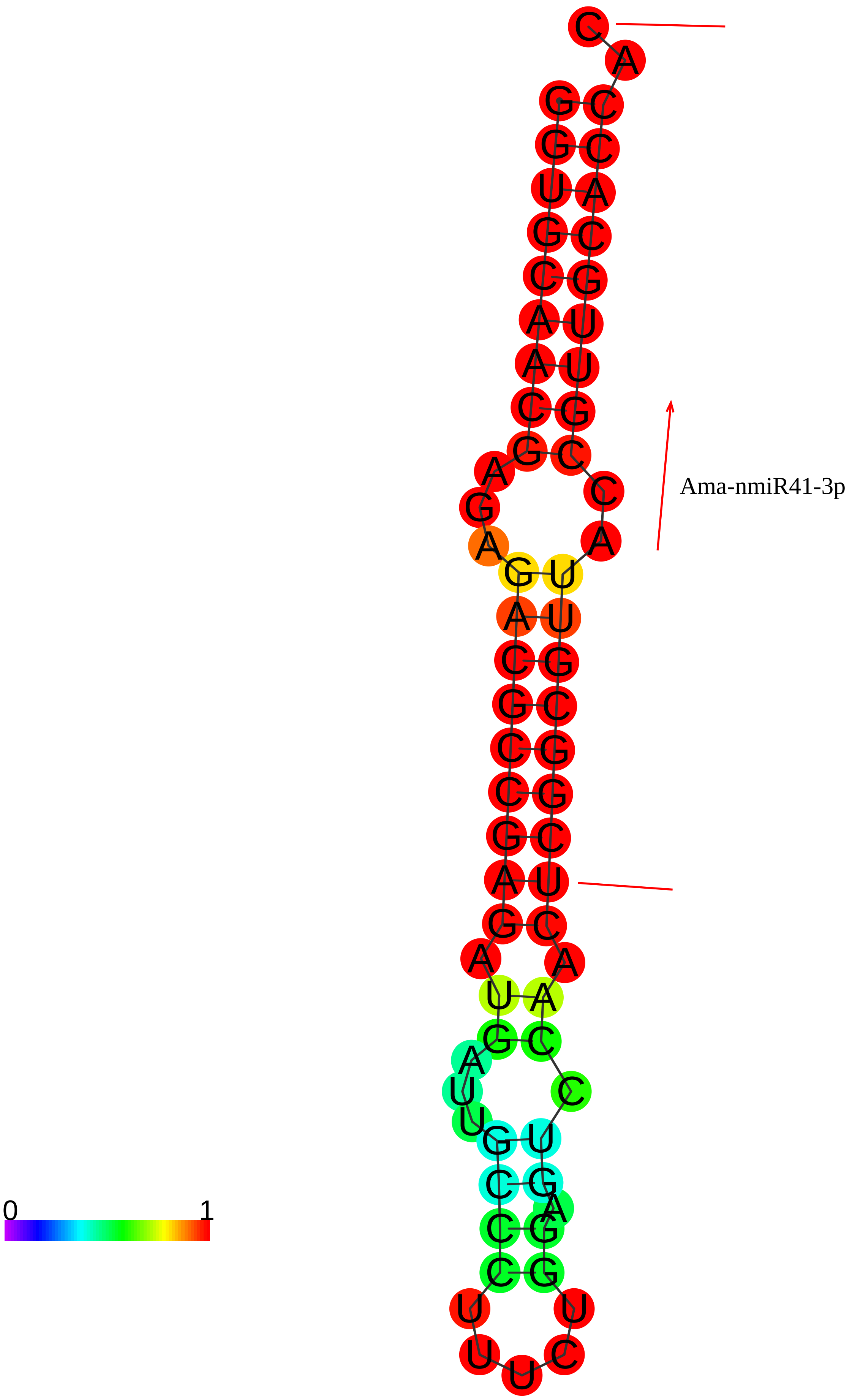
Ama-nmiR41-3p
0
1

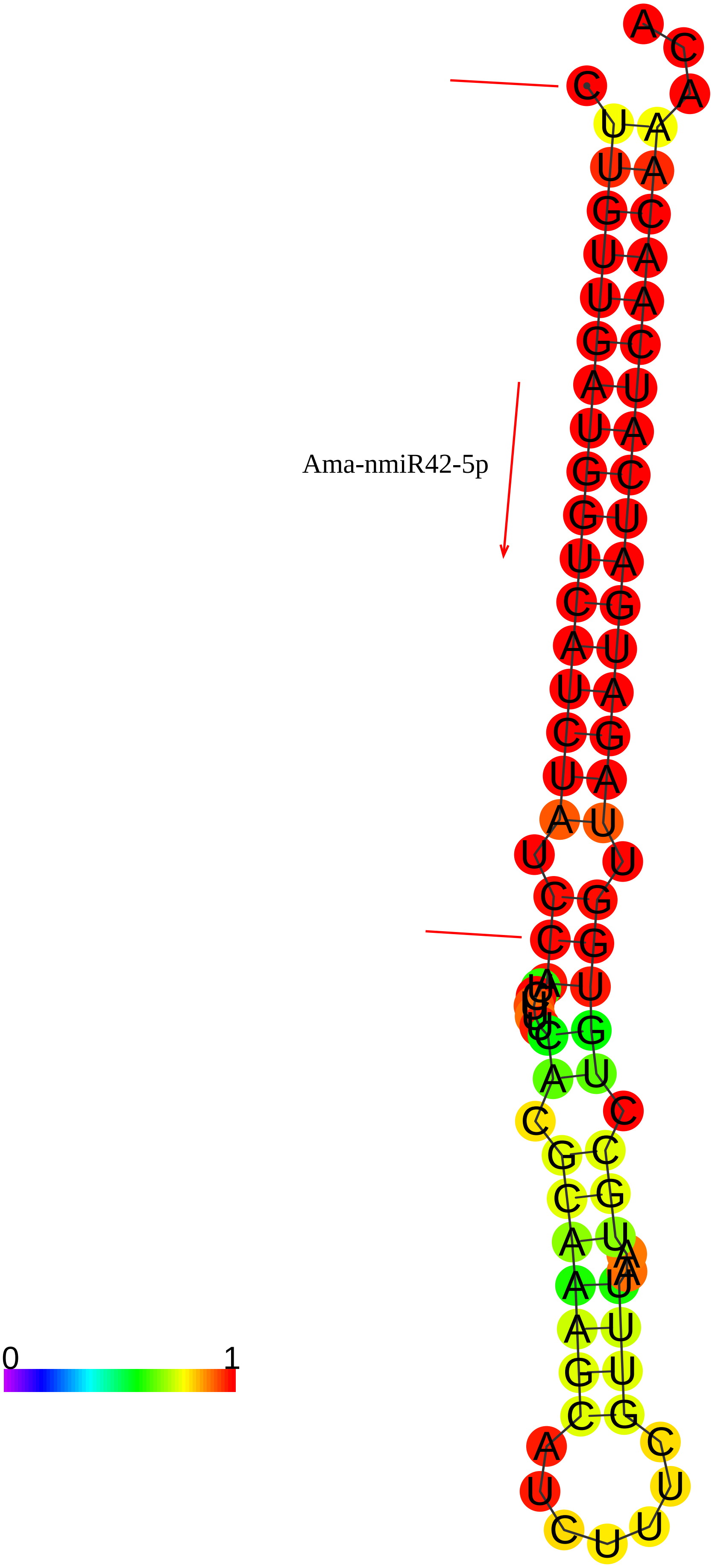
Ama-nmiR42-5p
0
1

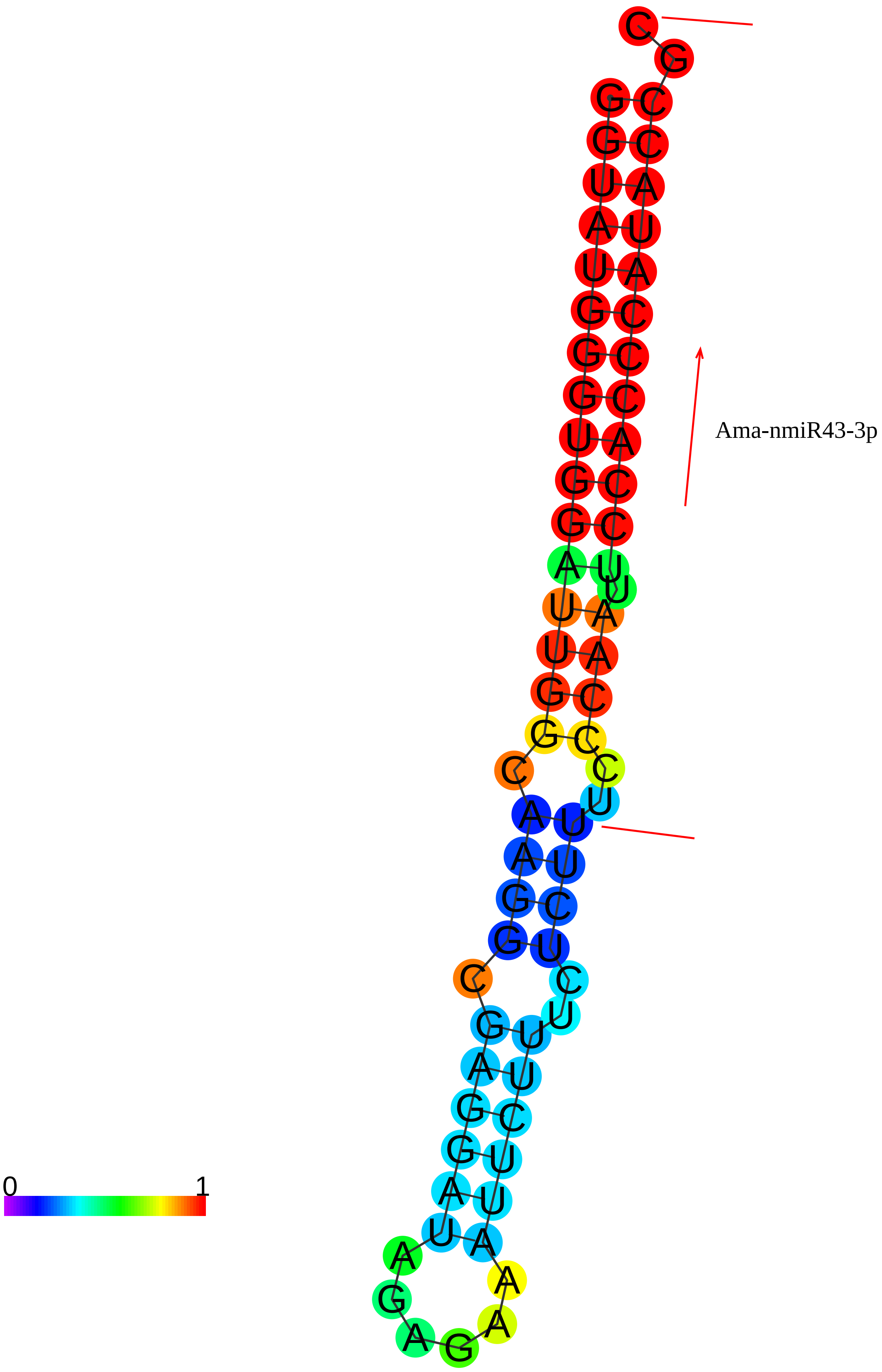

Ama-nmiR43-3p
0
1

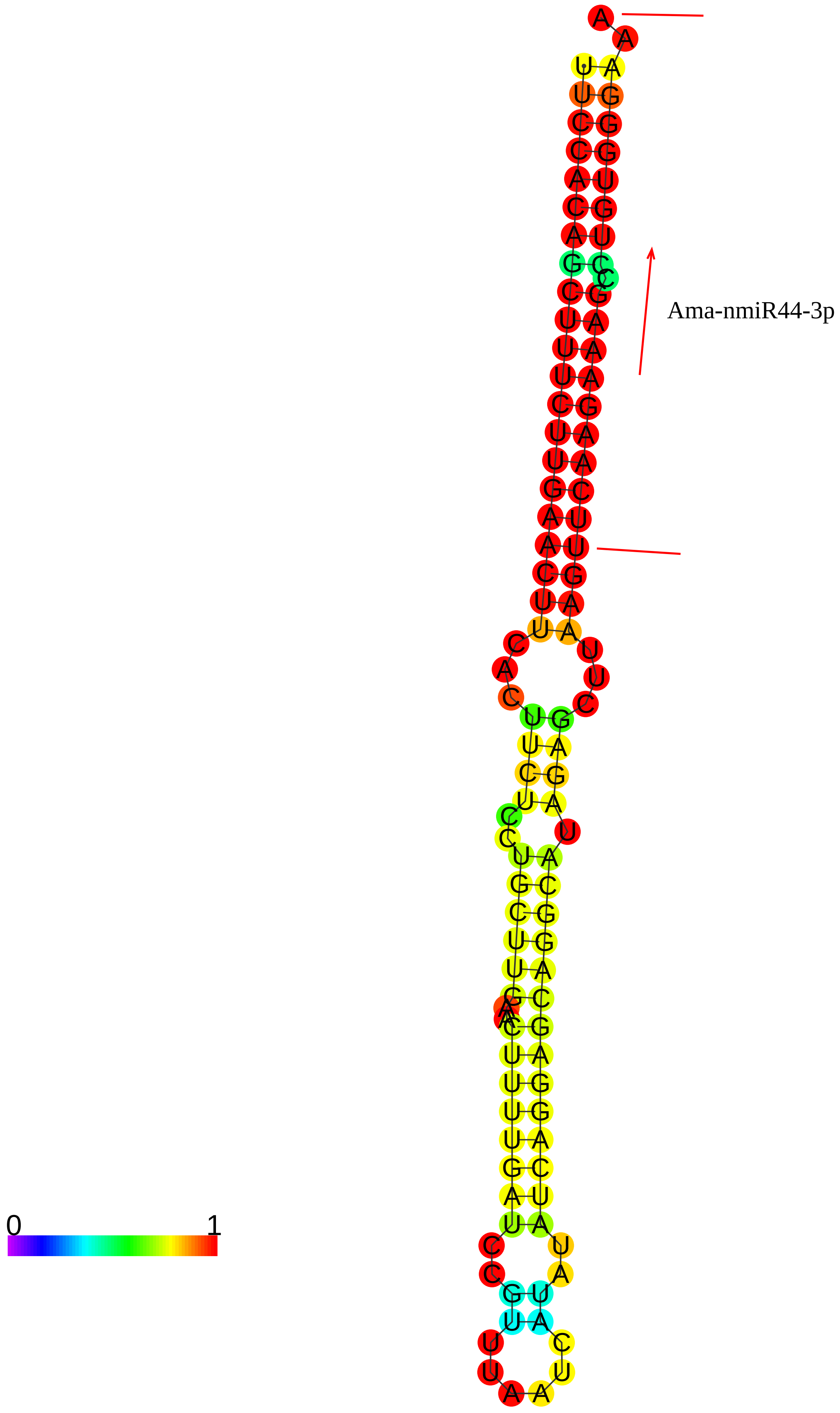
Ama-nmiR44-3p
0
1

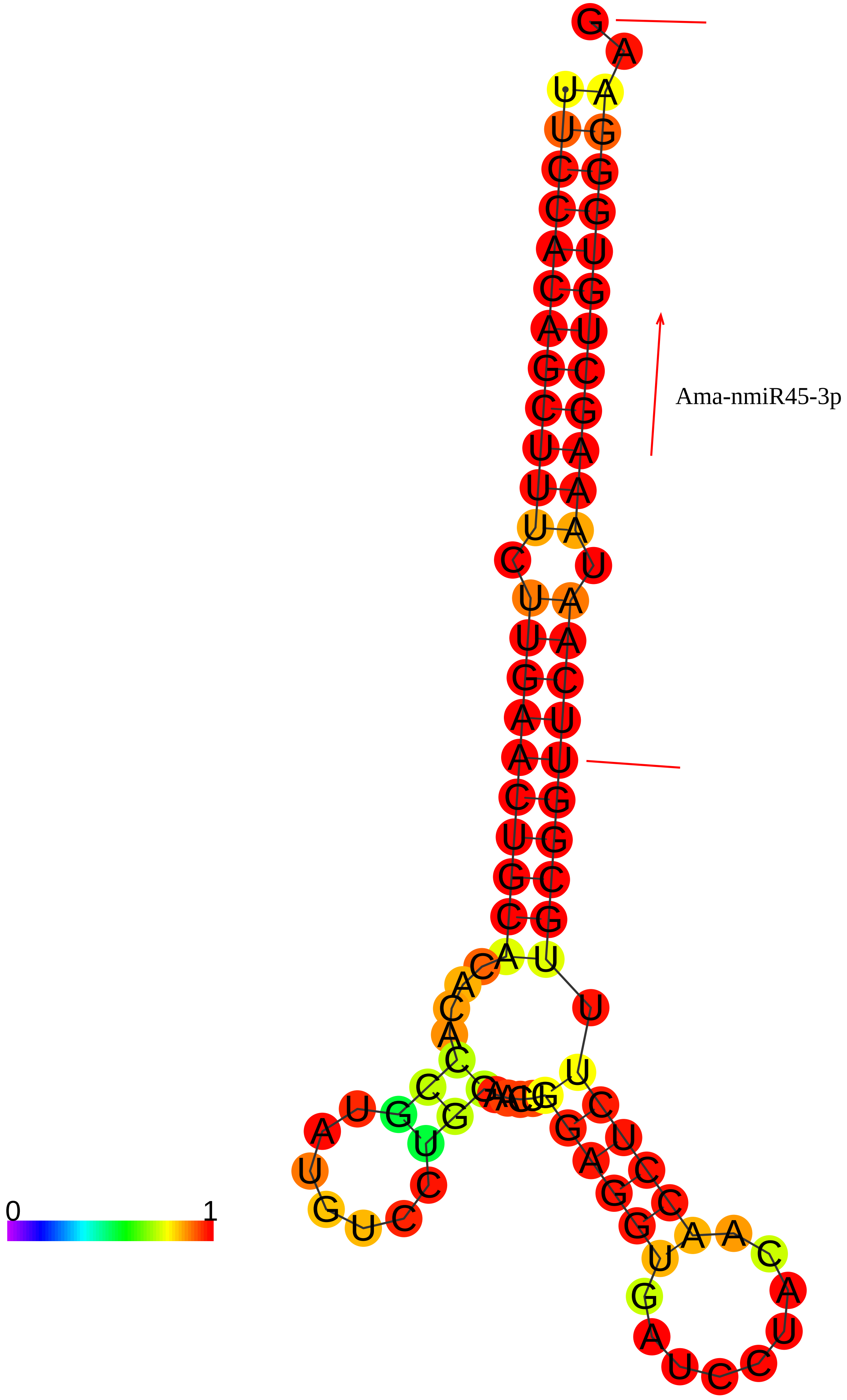
Ama-nmiR45-3p
0
1

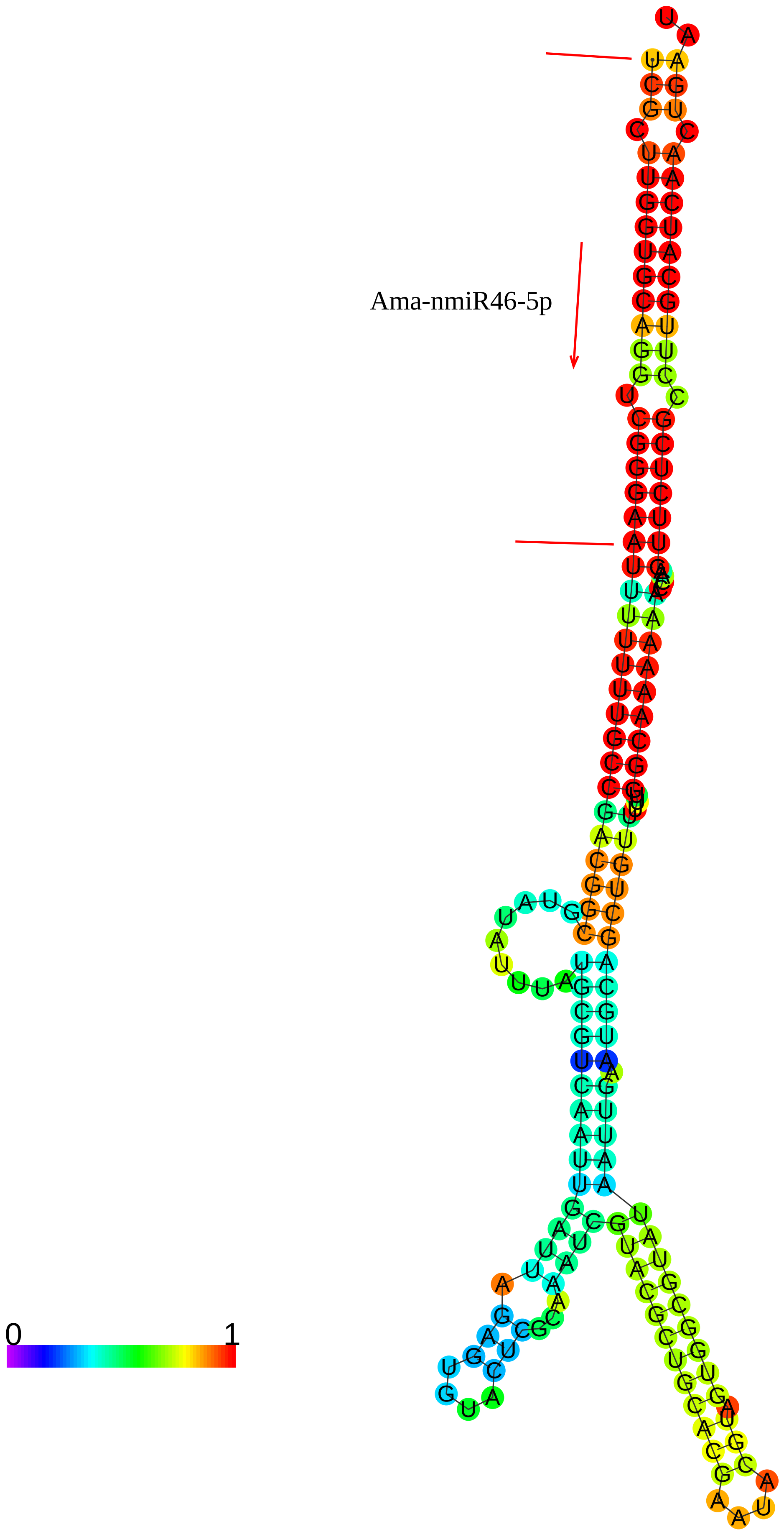
Ama-nmiR46-5p
0
1

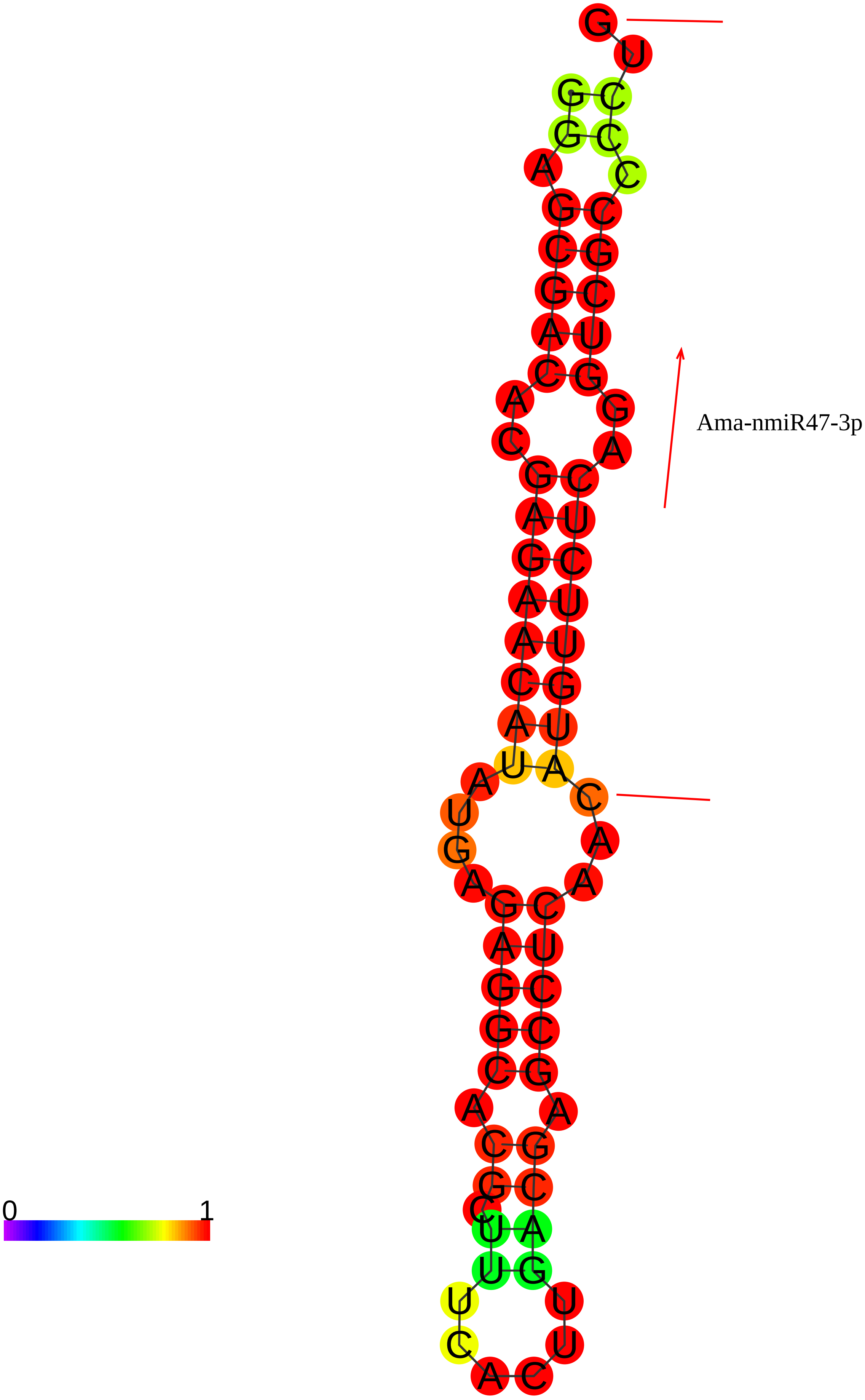
Ama-nmiR47-3p
0
1

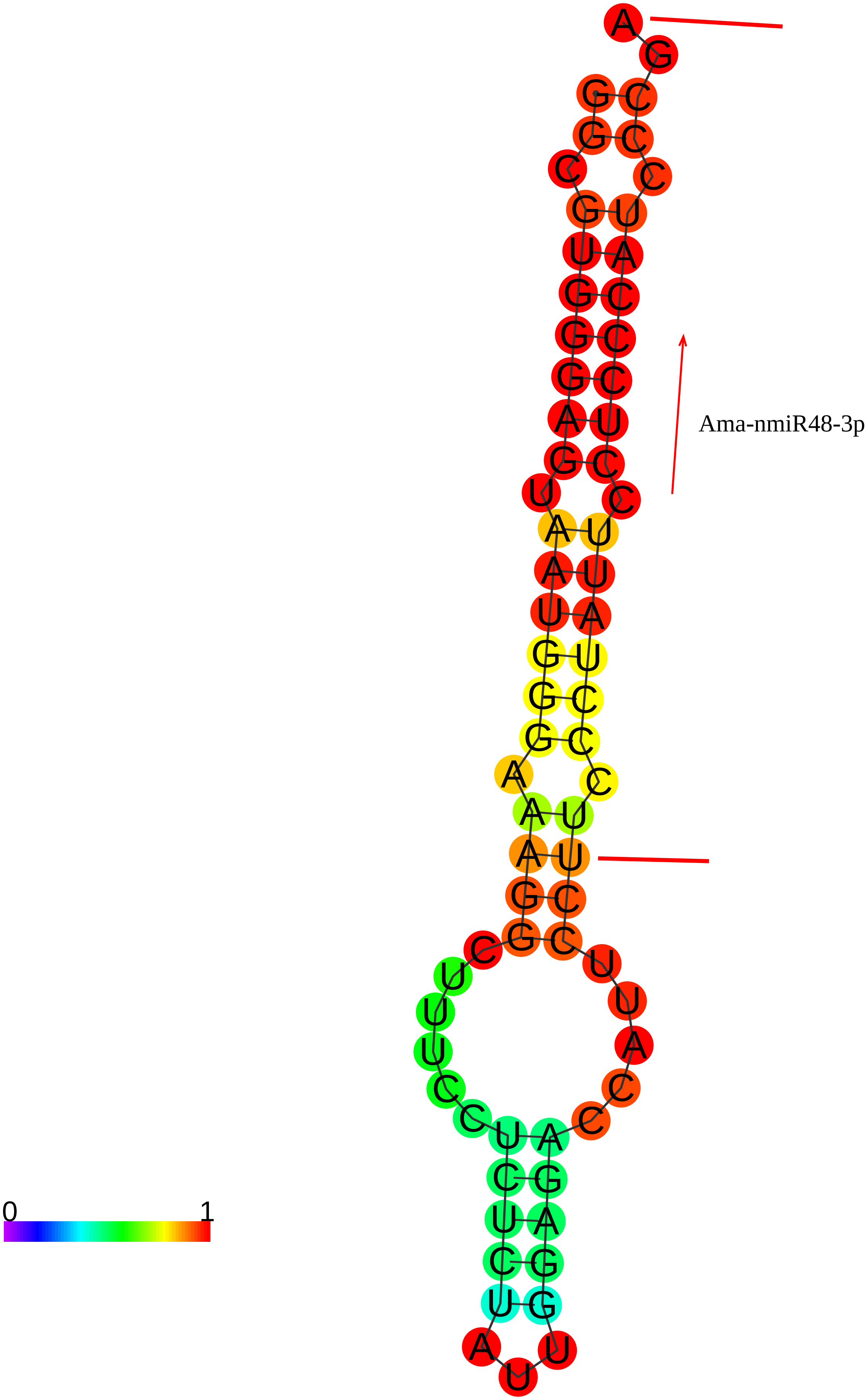
Ama-nmiR48-3p
0
1

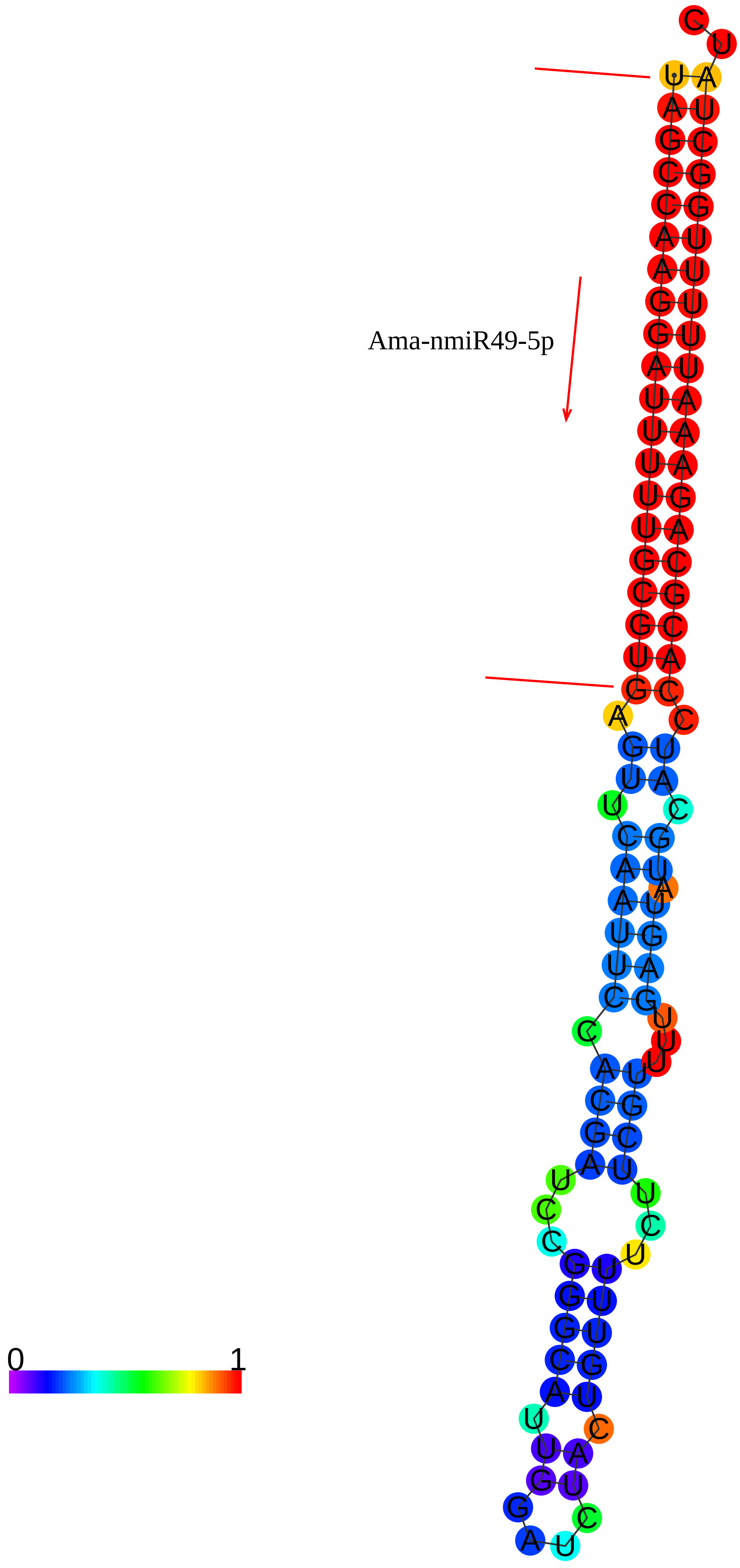
Ama-nmiR49-5p
0
1

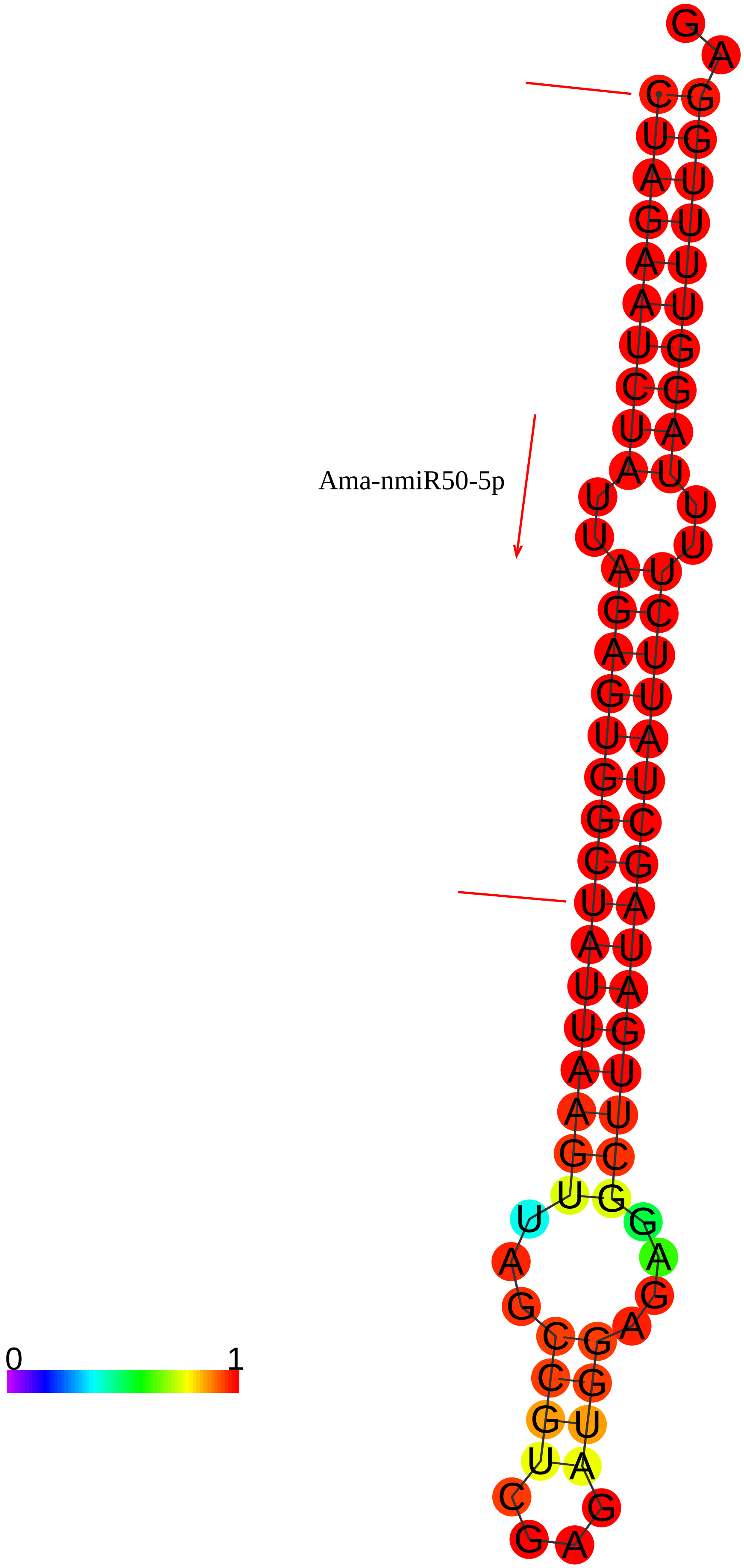
Ama-nmiR50-5p
0
1

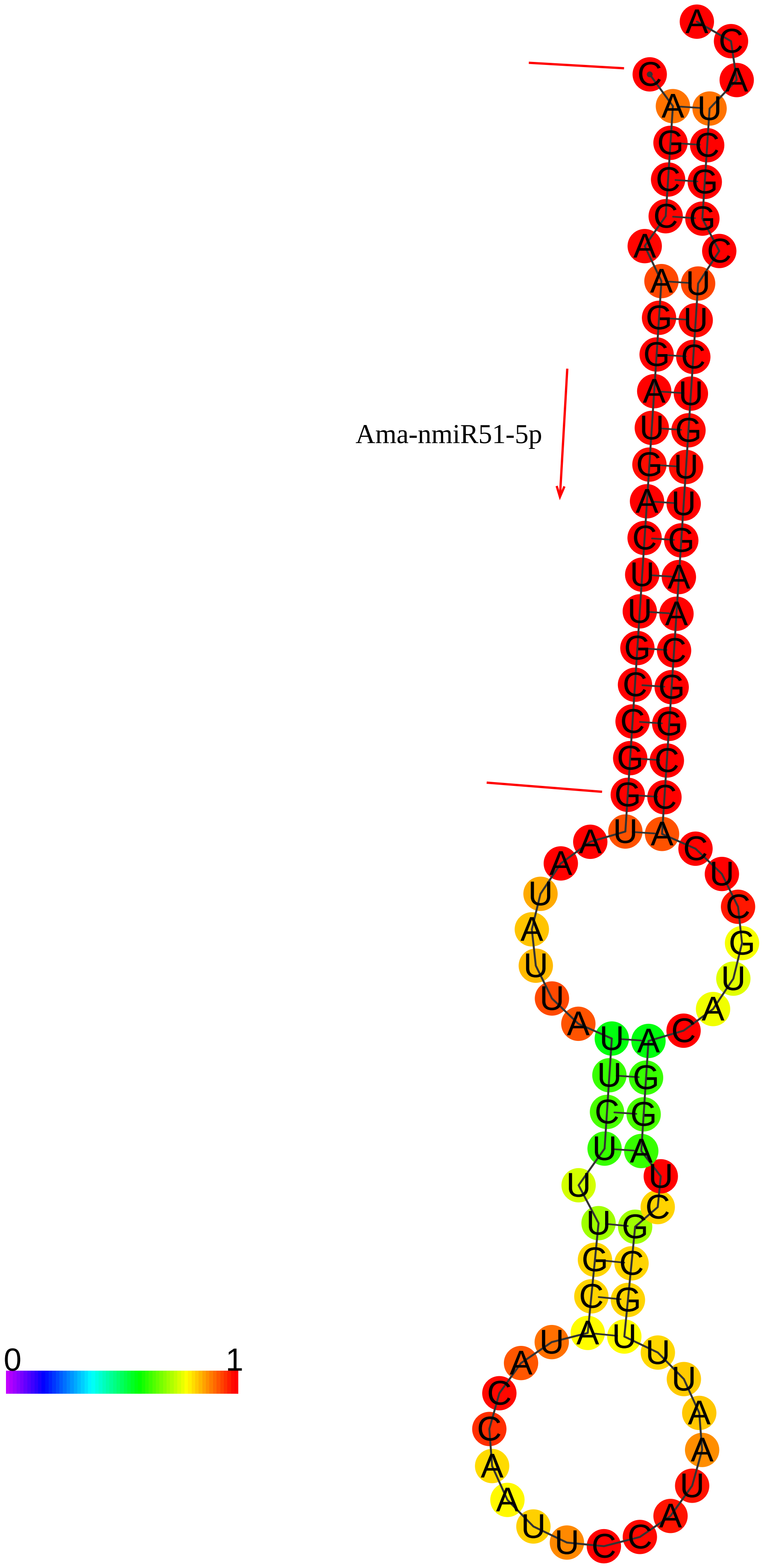
Ama-nmiR51-5p
0
1

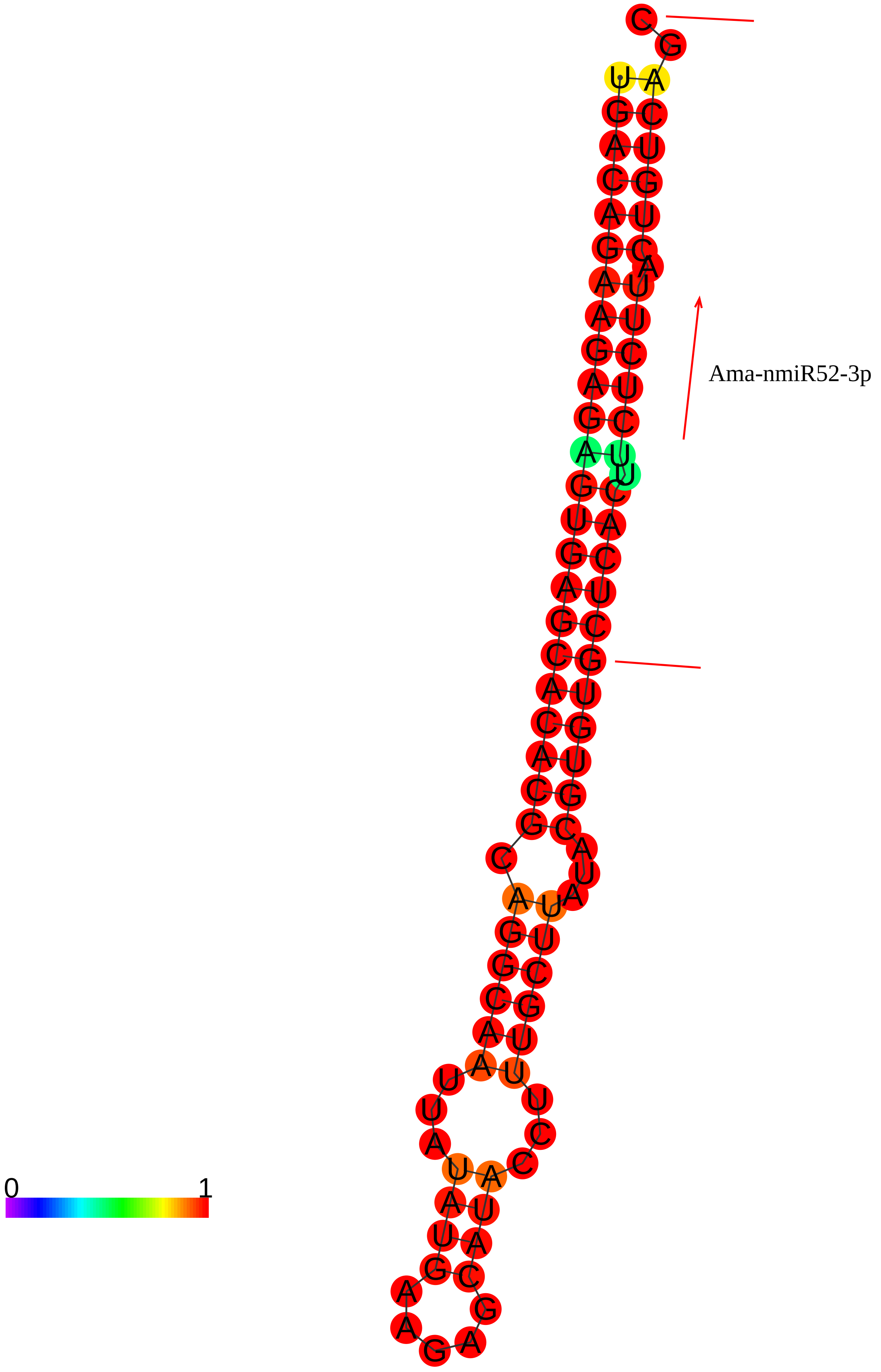
Ama-nmiR52-3p
0
1

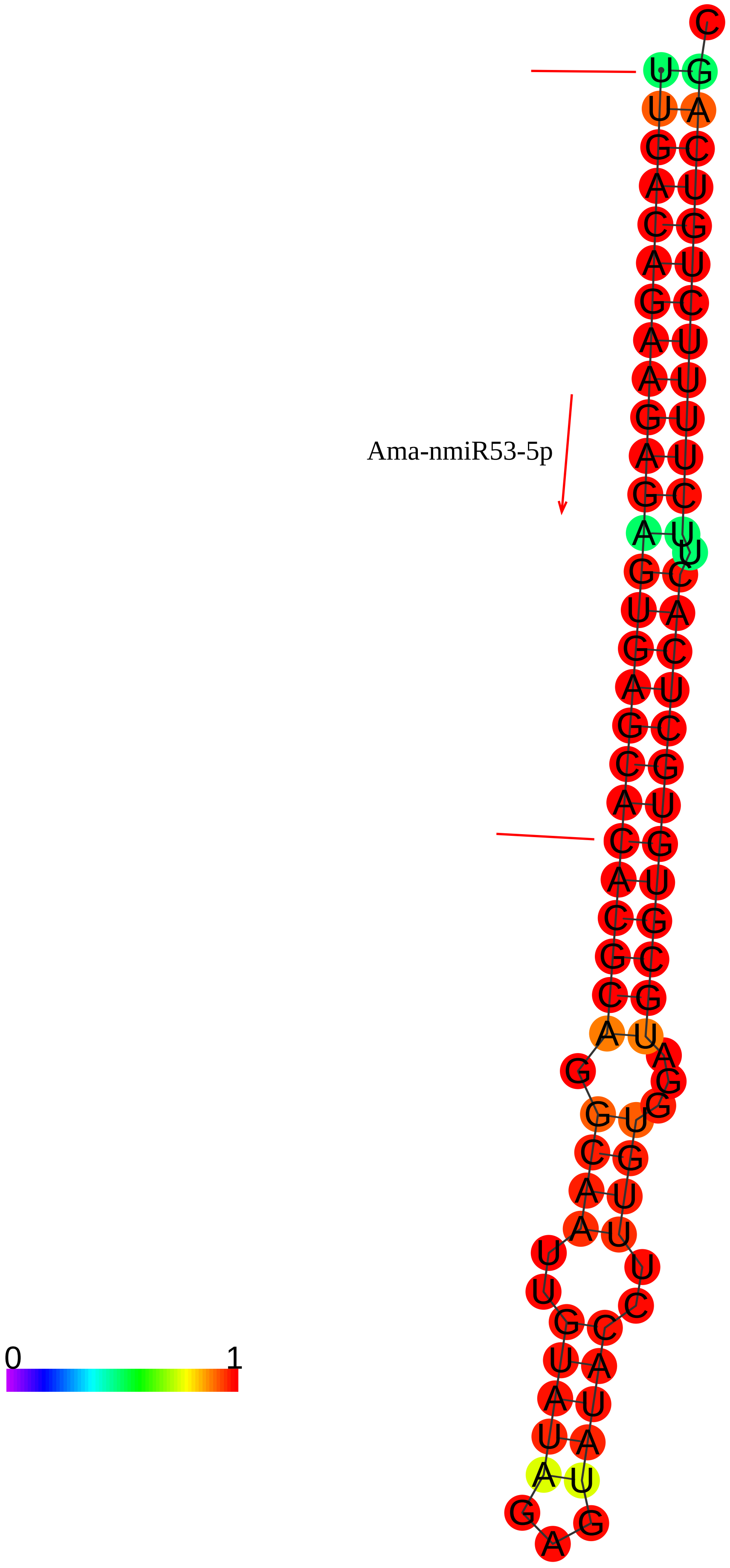
Ama-nmiR53-5p
0
1

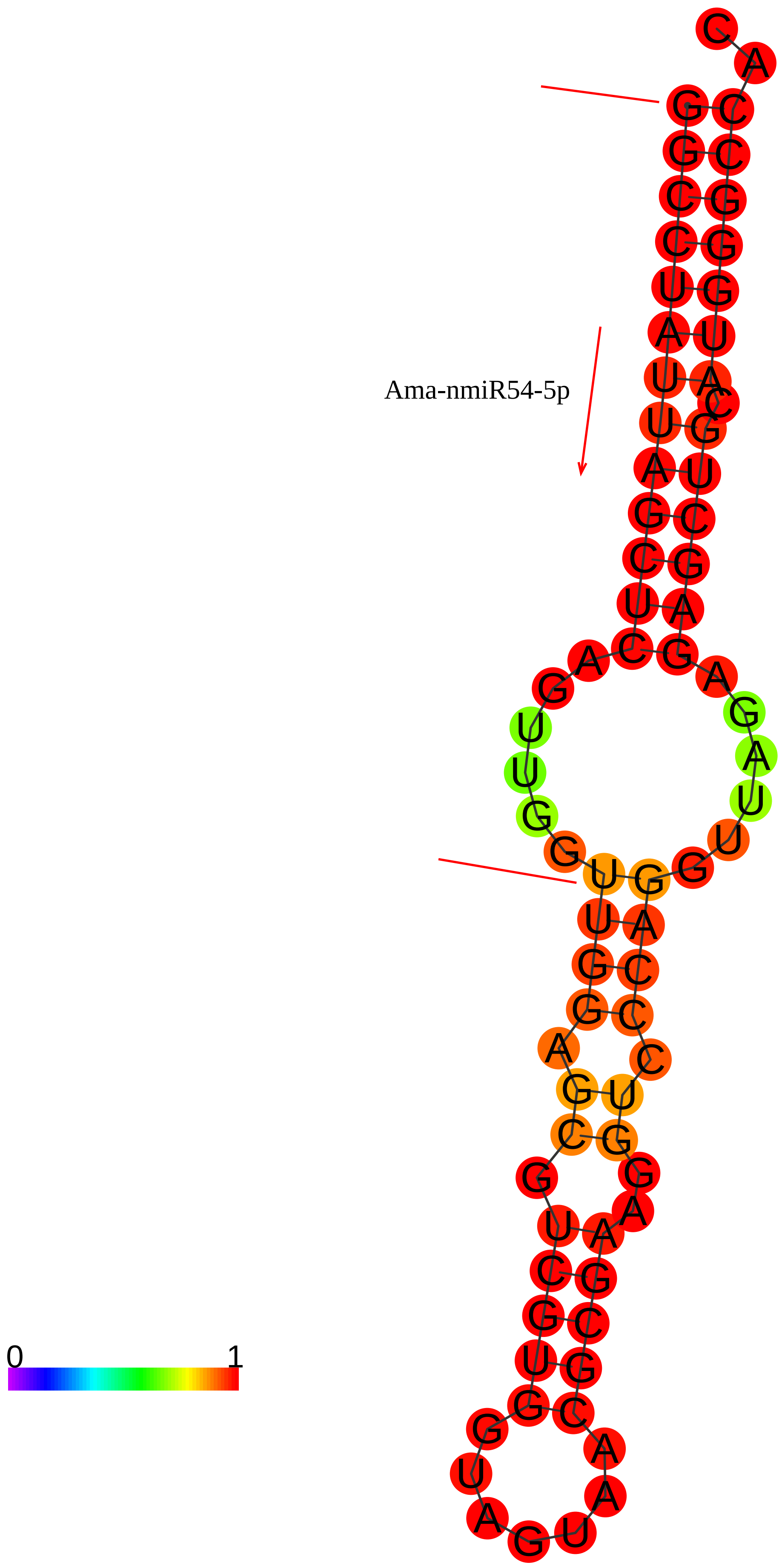
Ama-nmiR54-5p
0
1

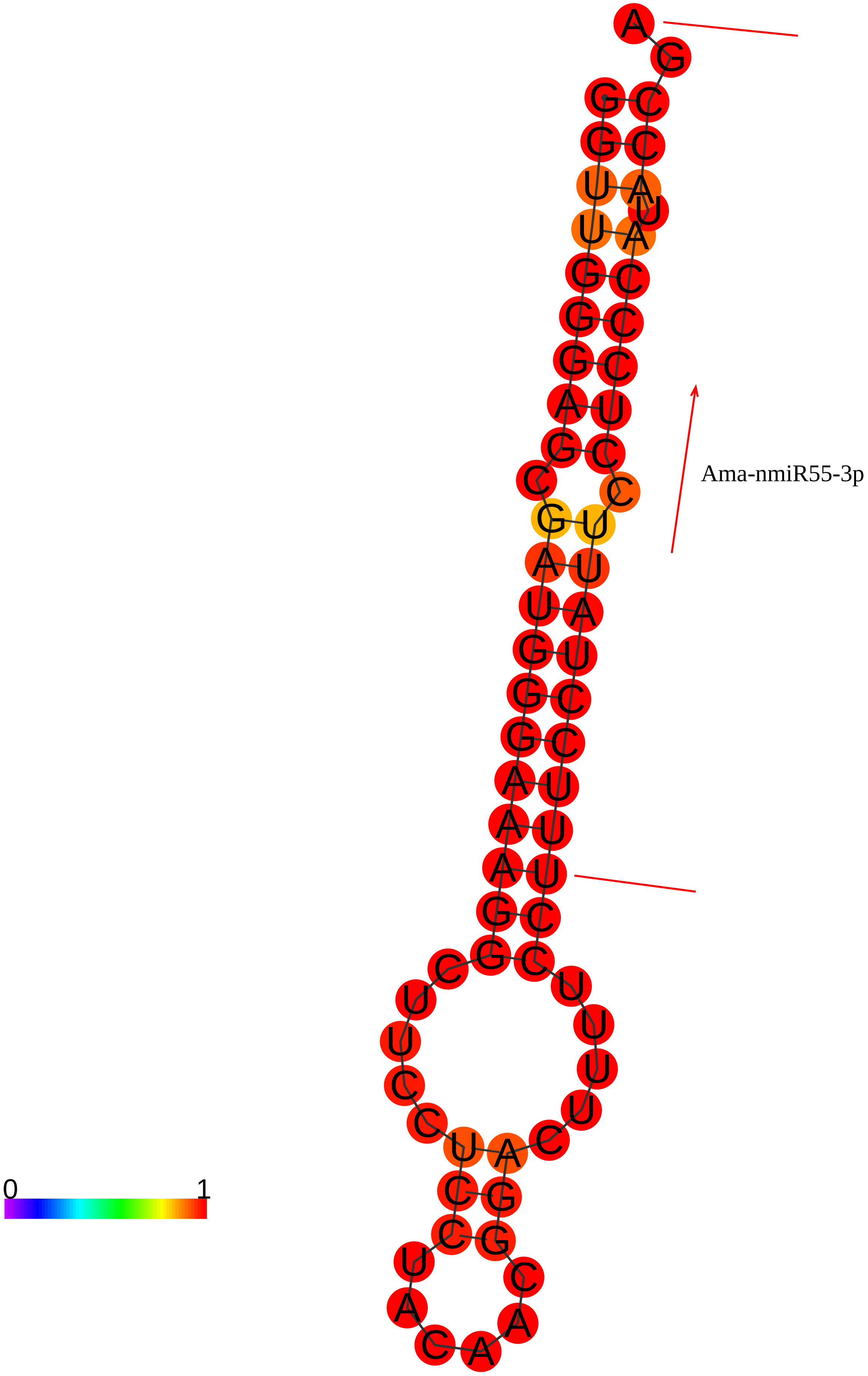
Ama-nmiR55-3p
0
1

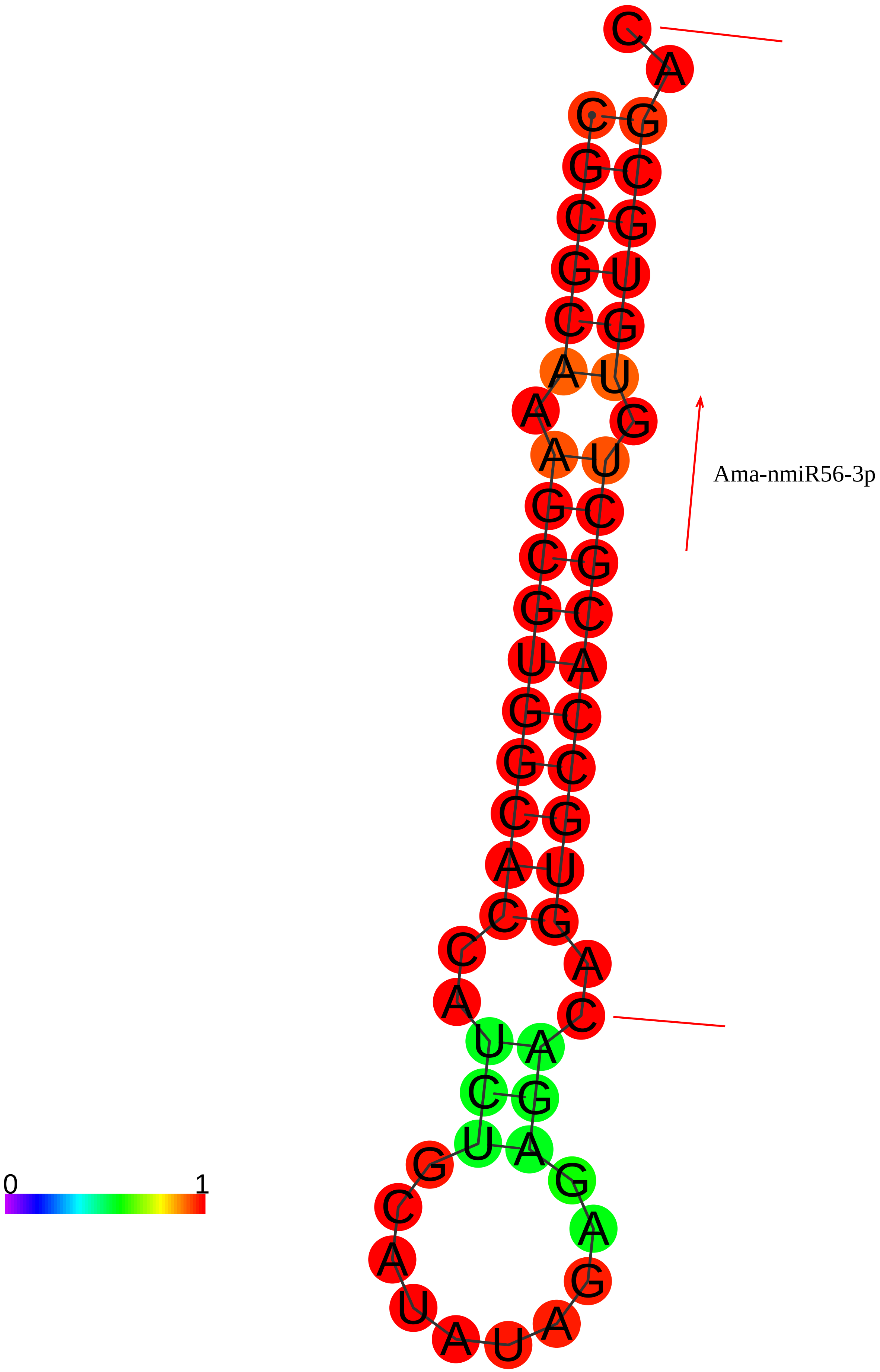

Ama-nmiR56-3p
0
1

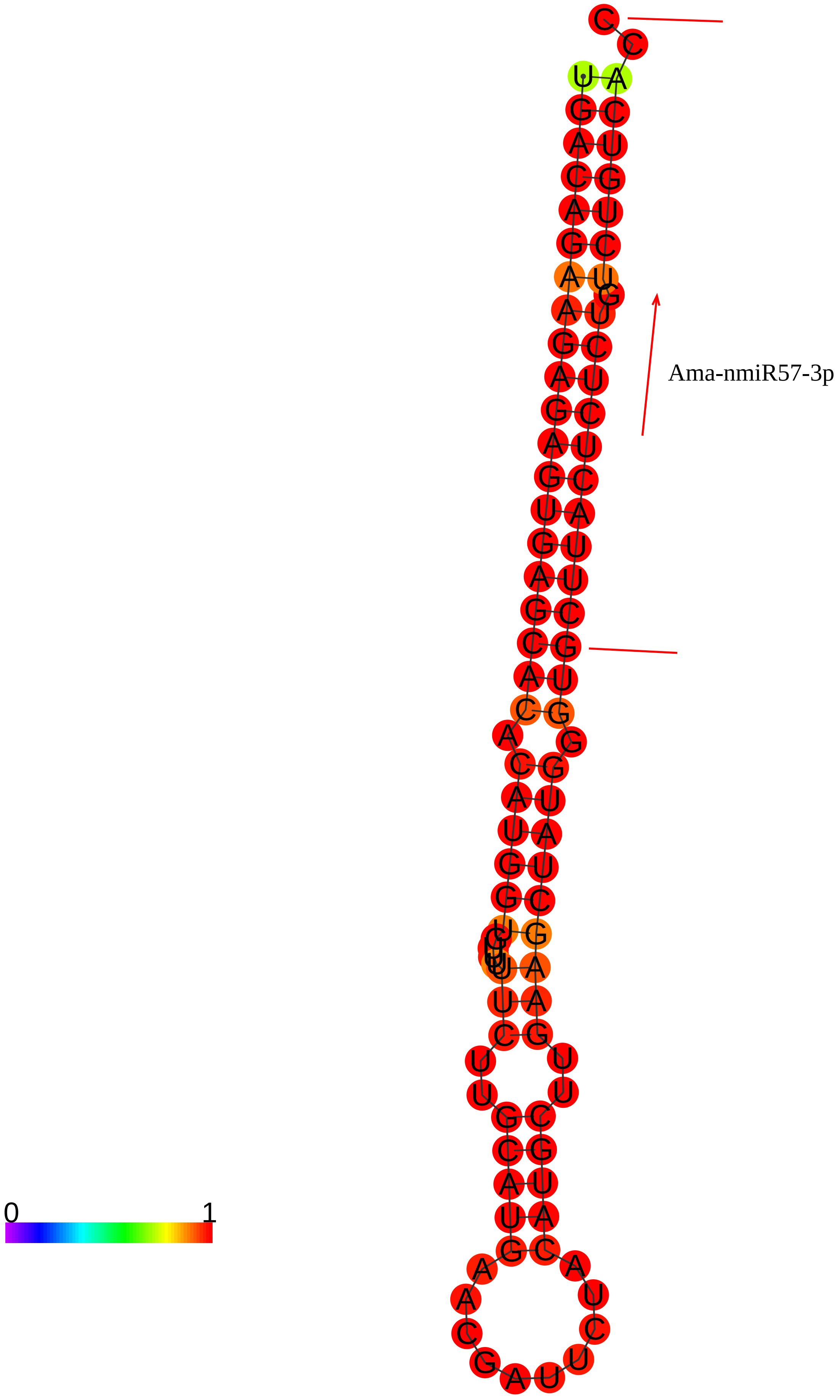
Ama-nmiR57-3p
0
1

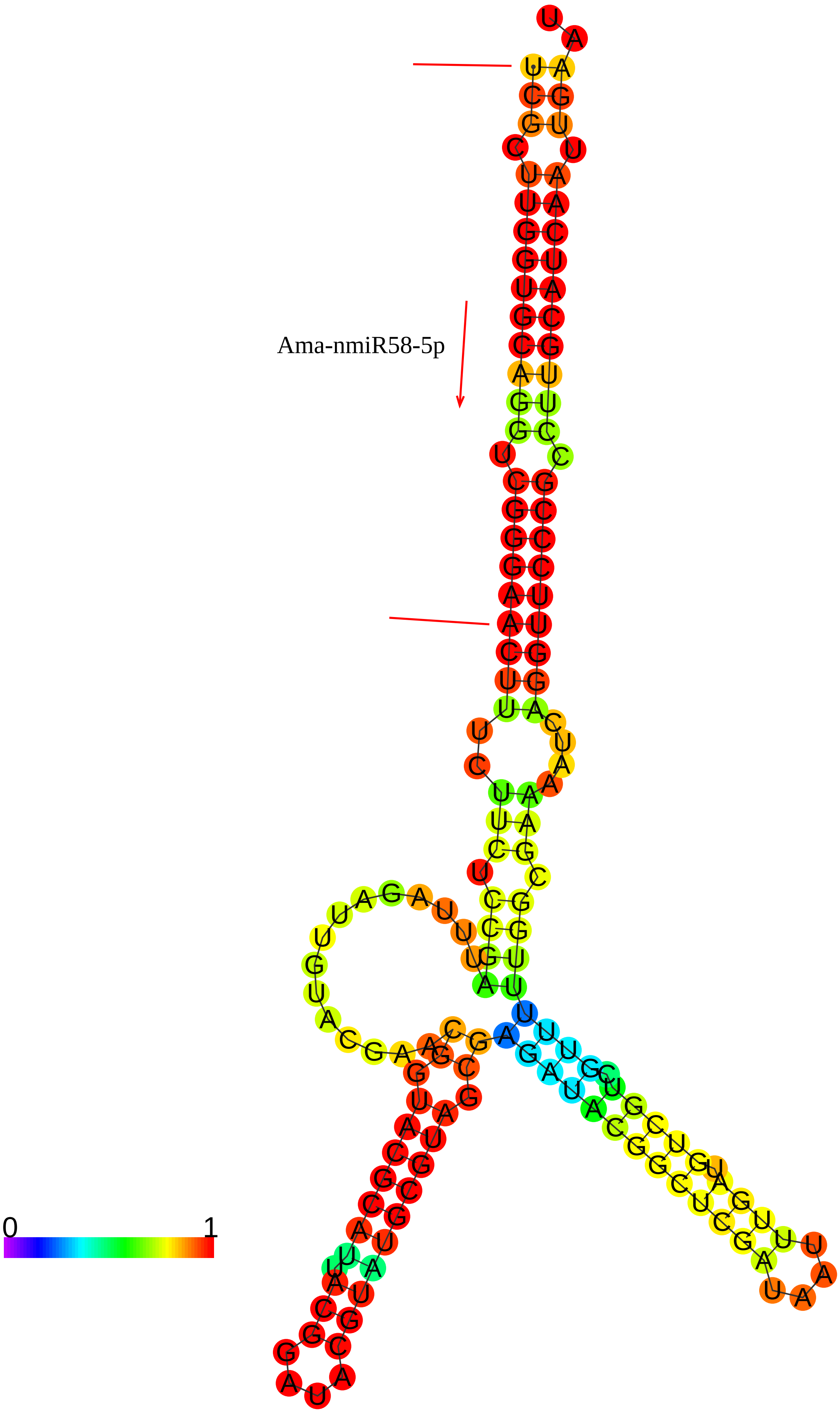
Ama-nmiR58-5p
0
1

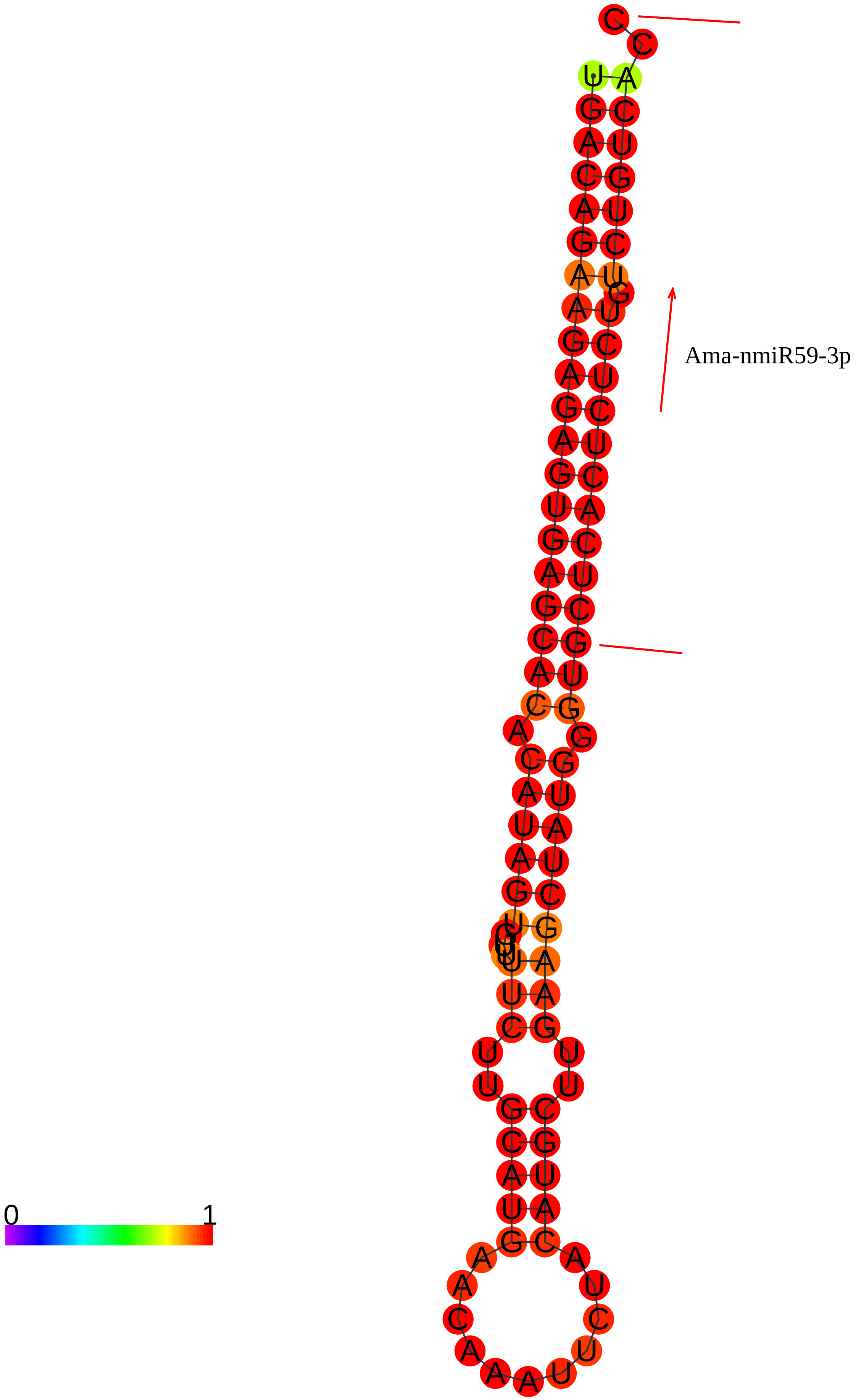
Ama-nmiR59-3p
0
1

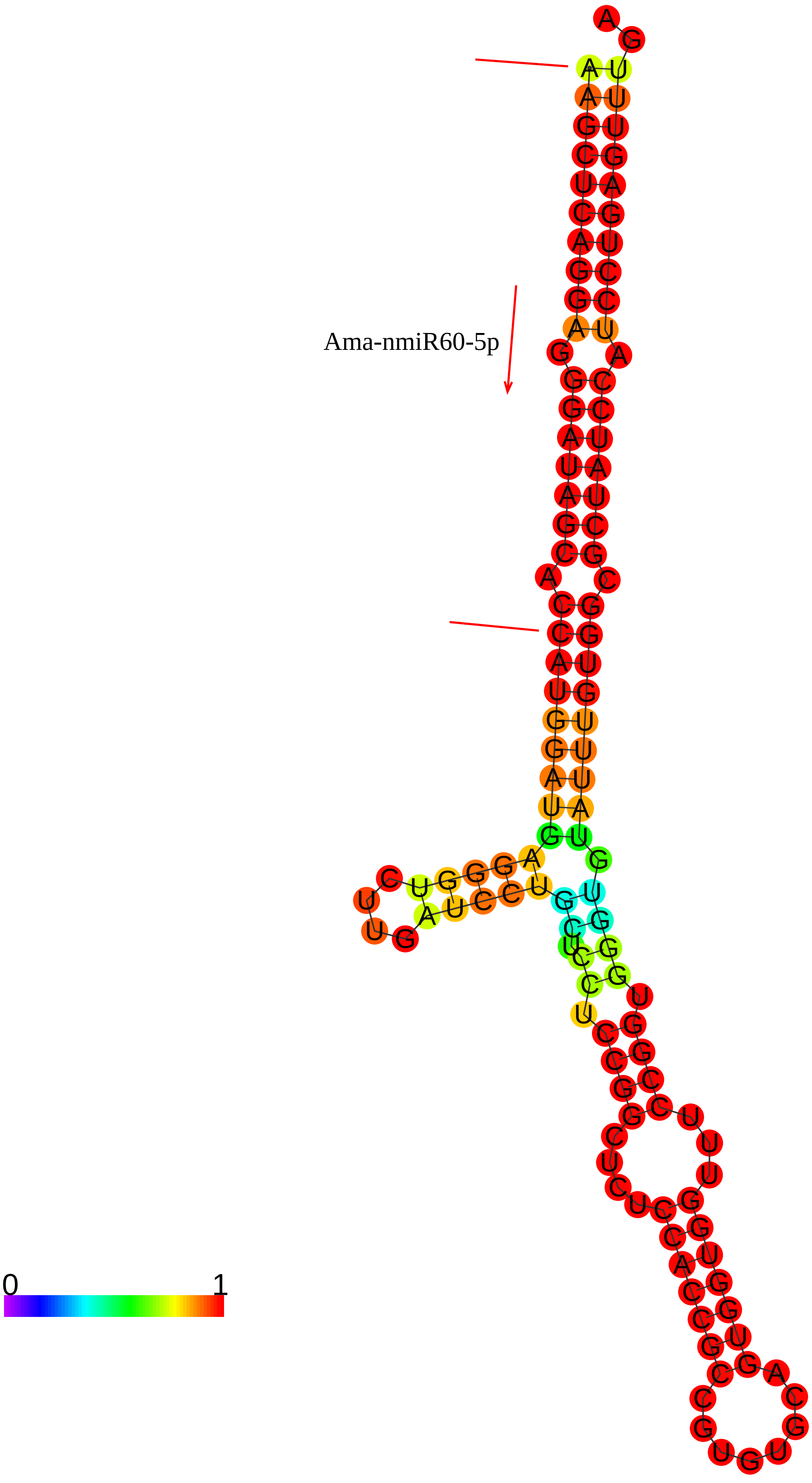
Ama-nmiR60-5p
0
1

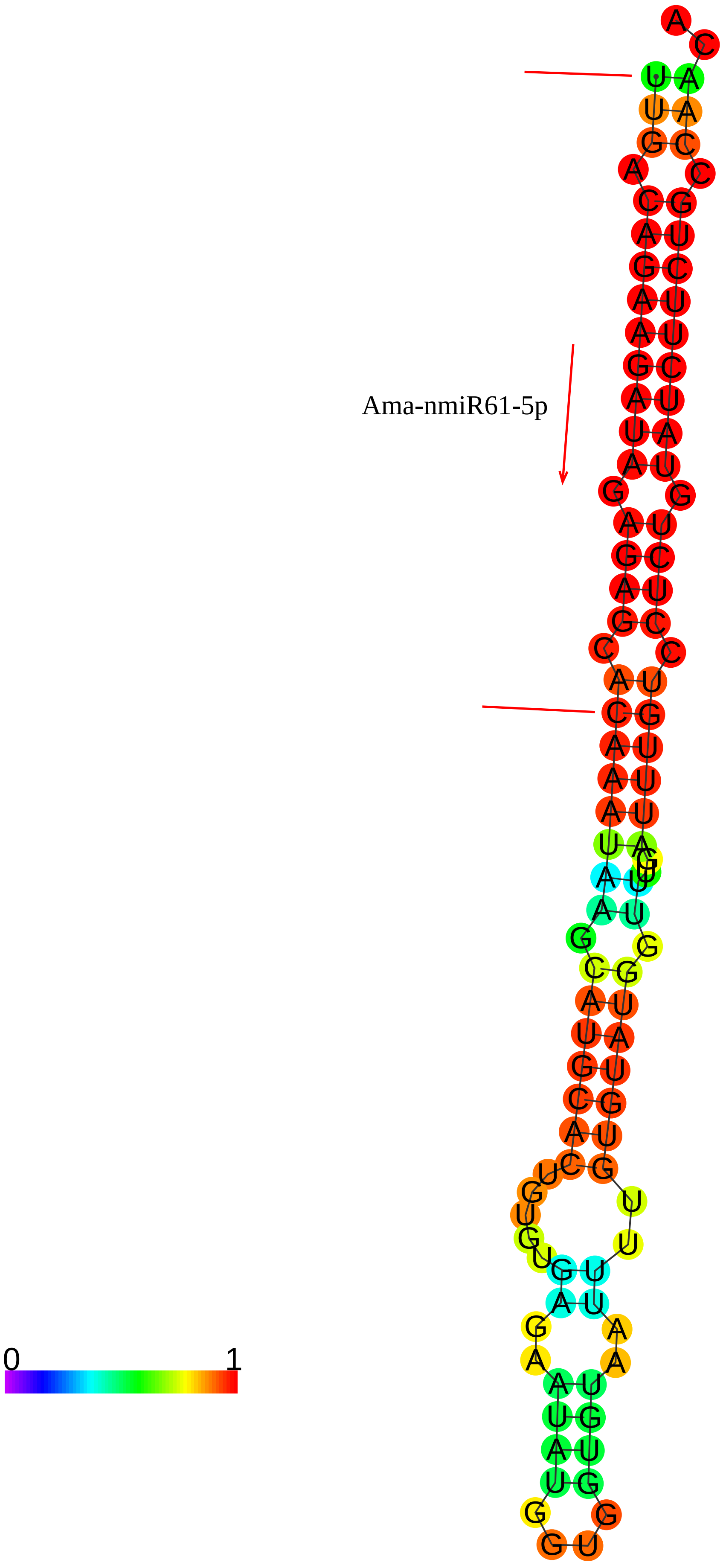
Ama-nmiR61-5p
0
1

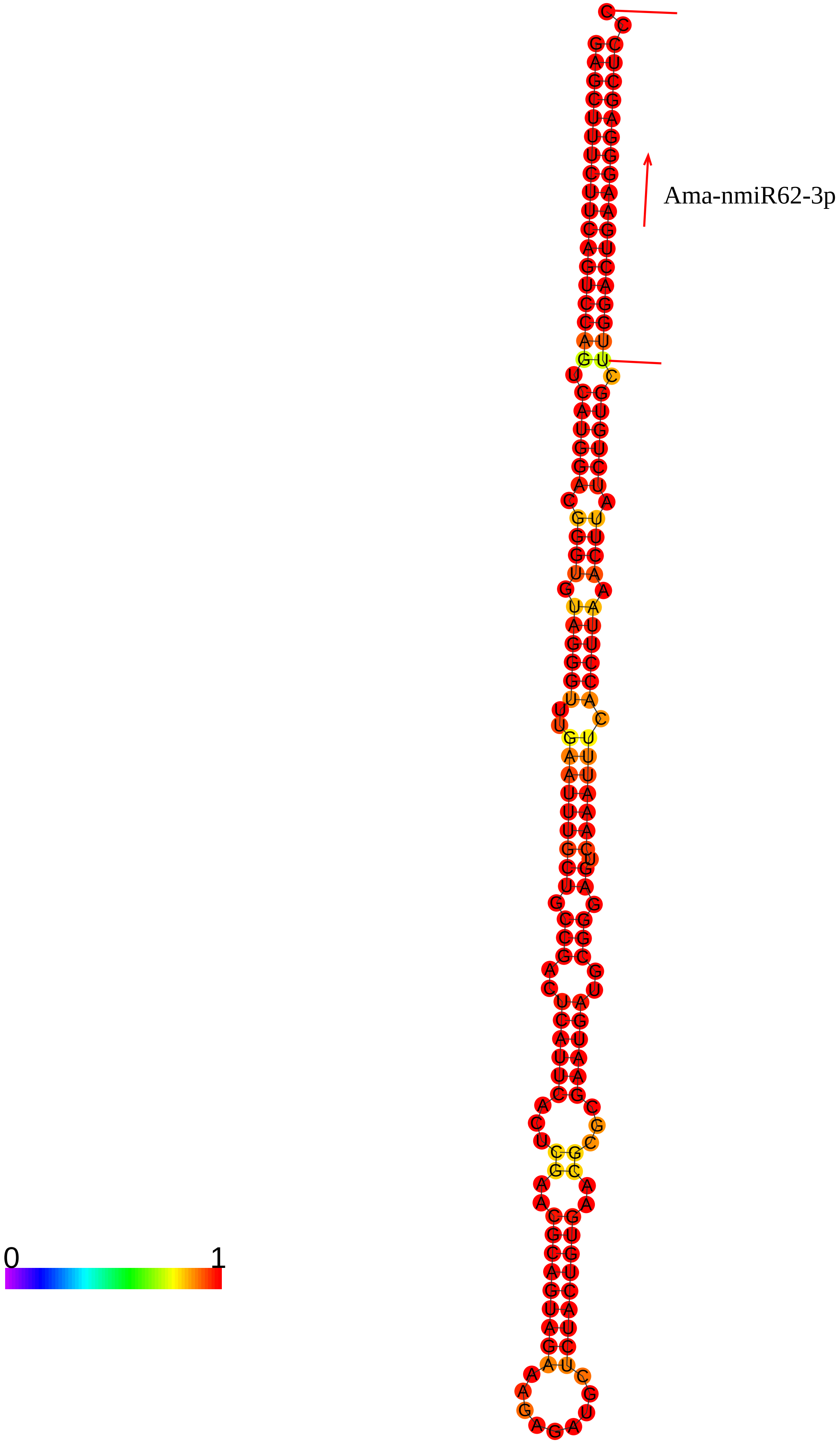

Ama-nmiR62-3p
0
1

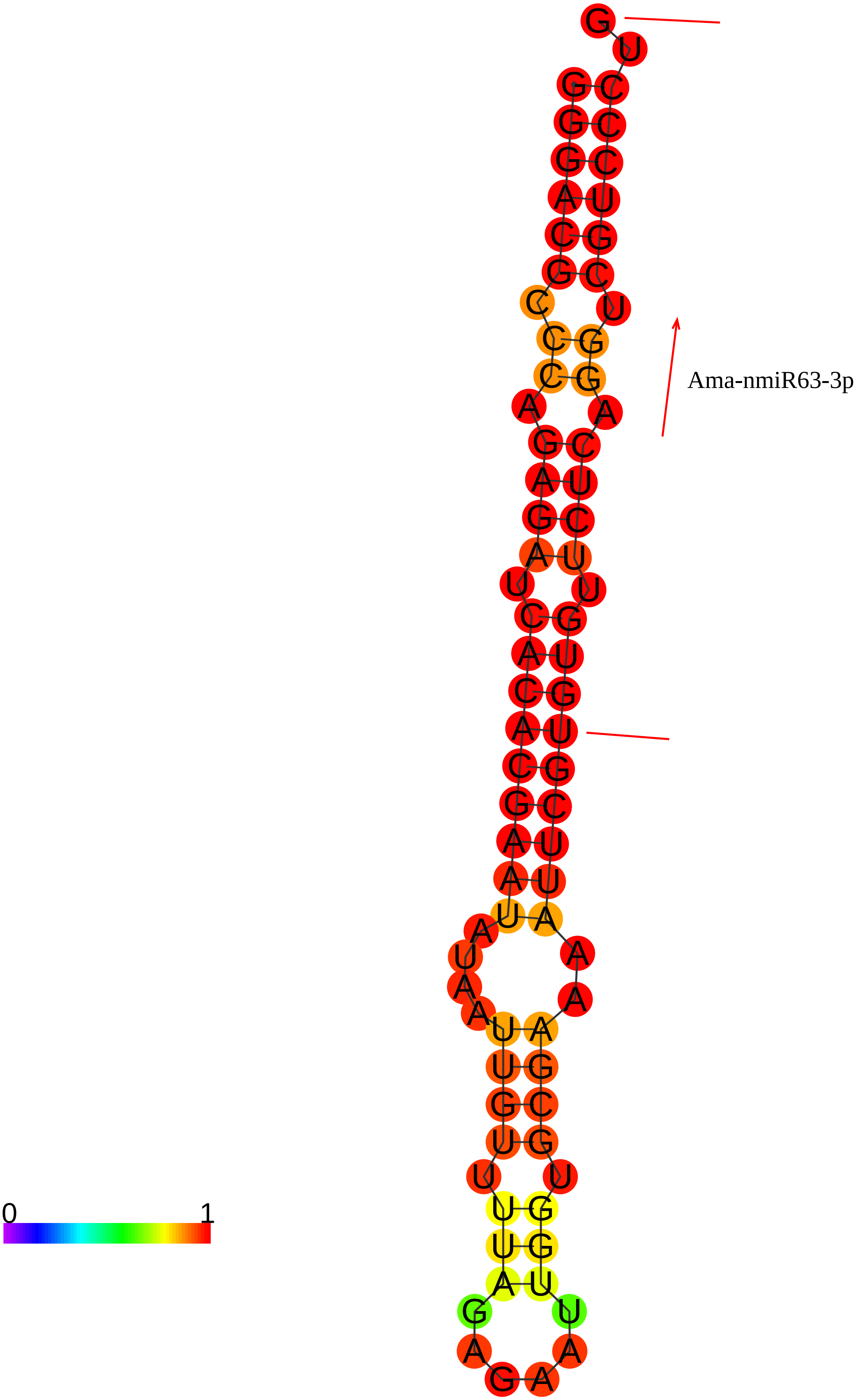
Ama-nmiR63-3p
0
1

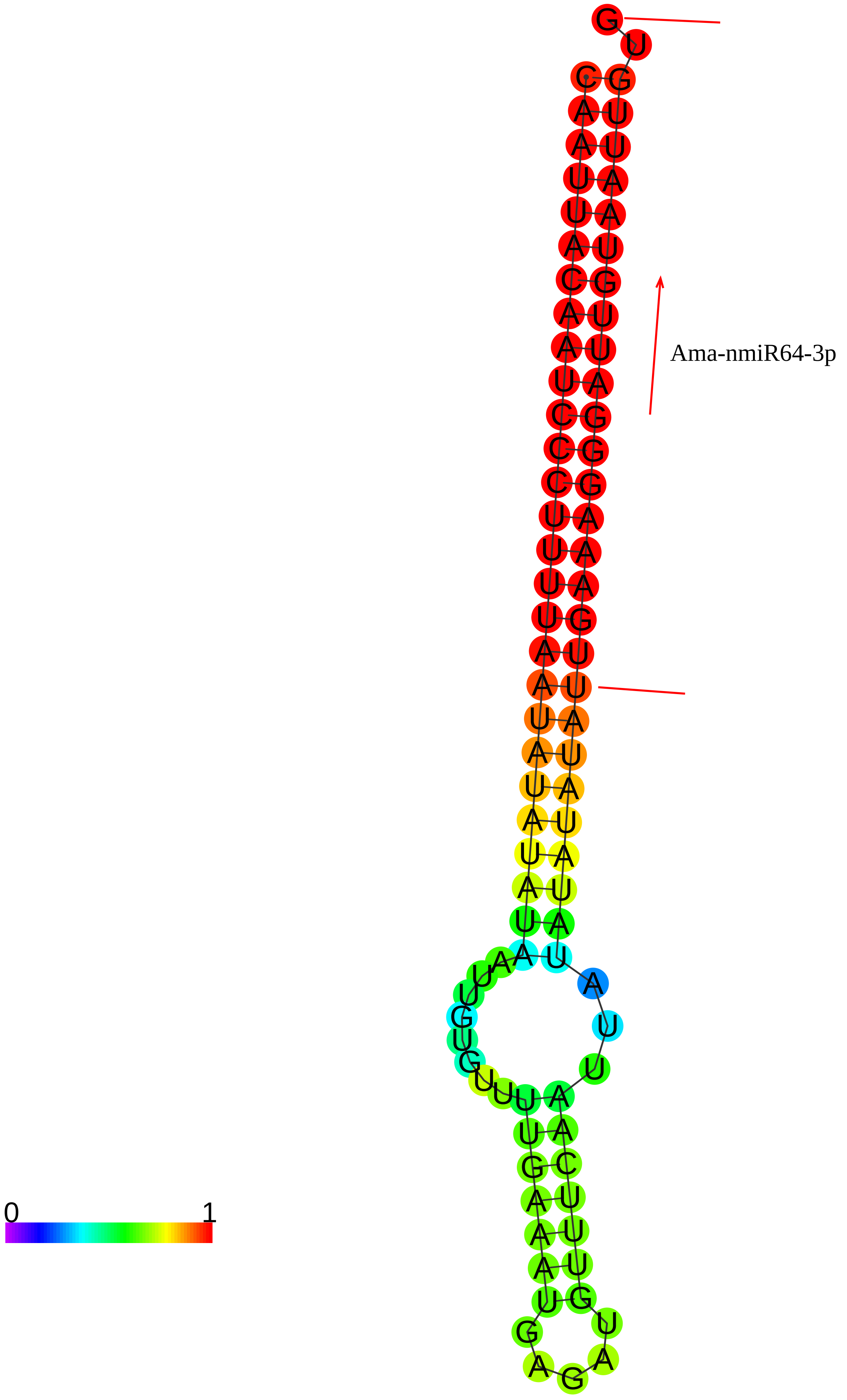
Ama-nmiR64-3p
0
1

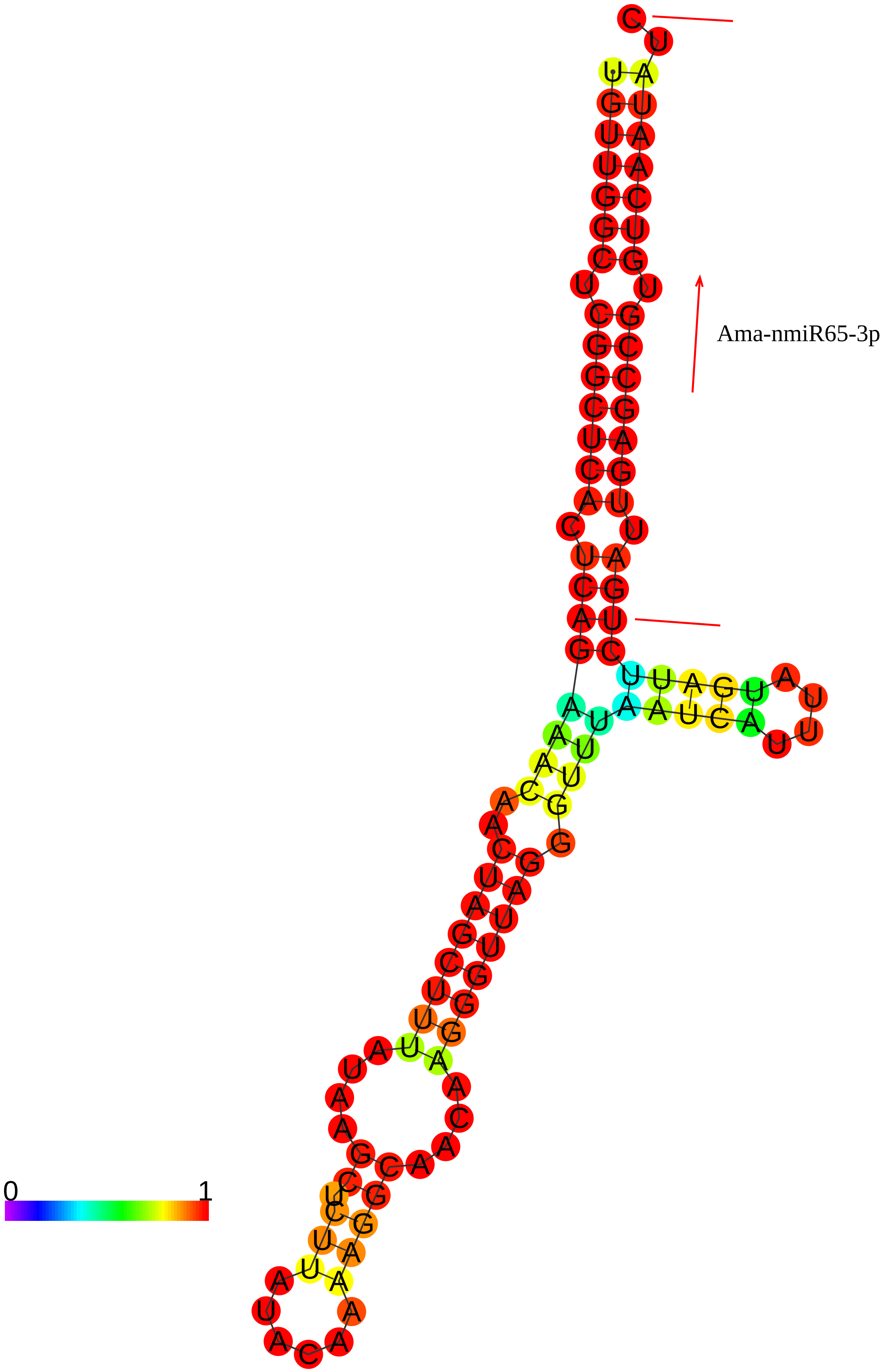
Ama-nmiR65-3p
0
1

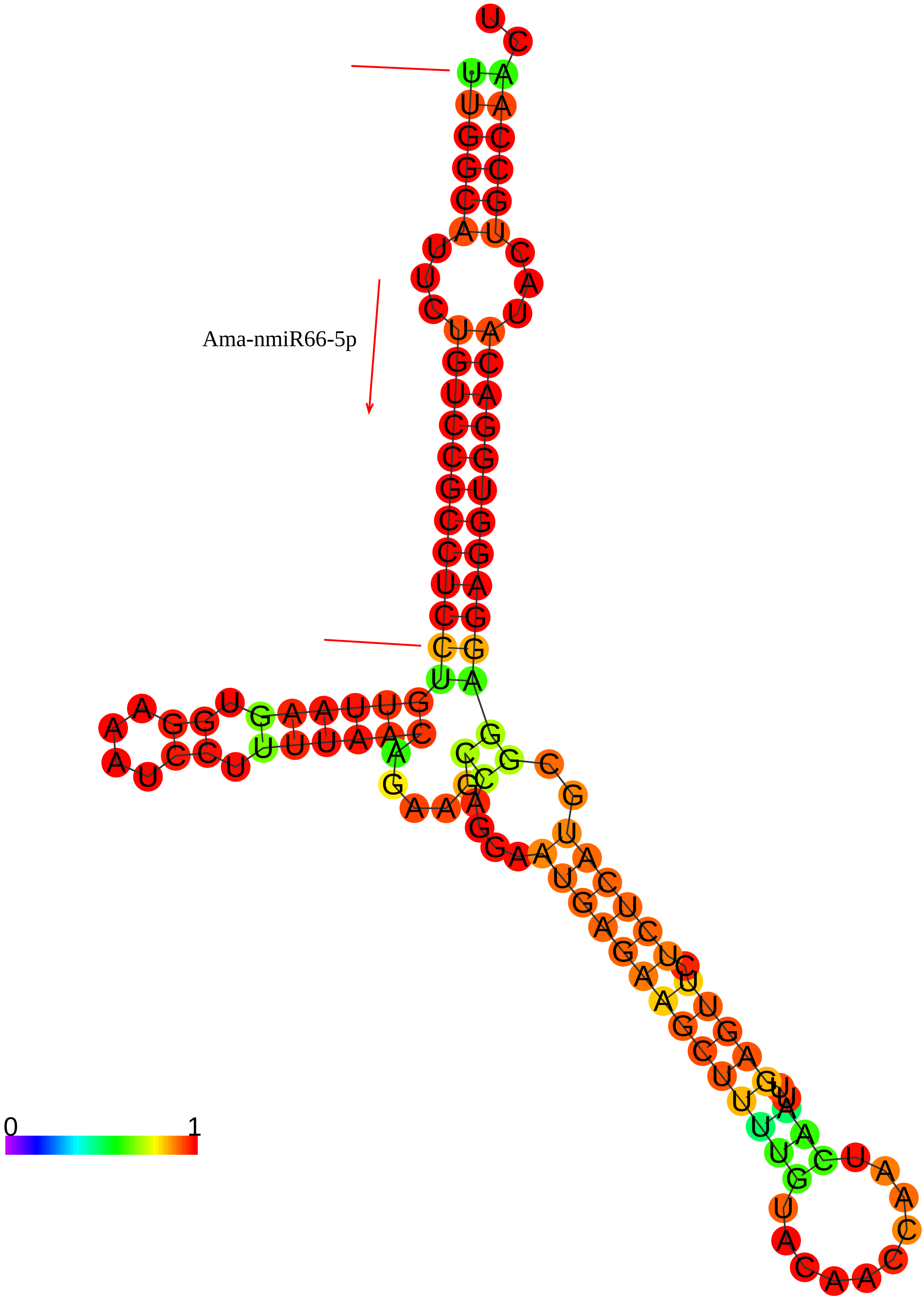
Ama-nmiR66-5p
0
1

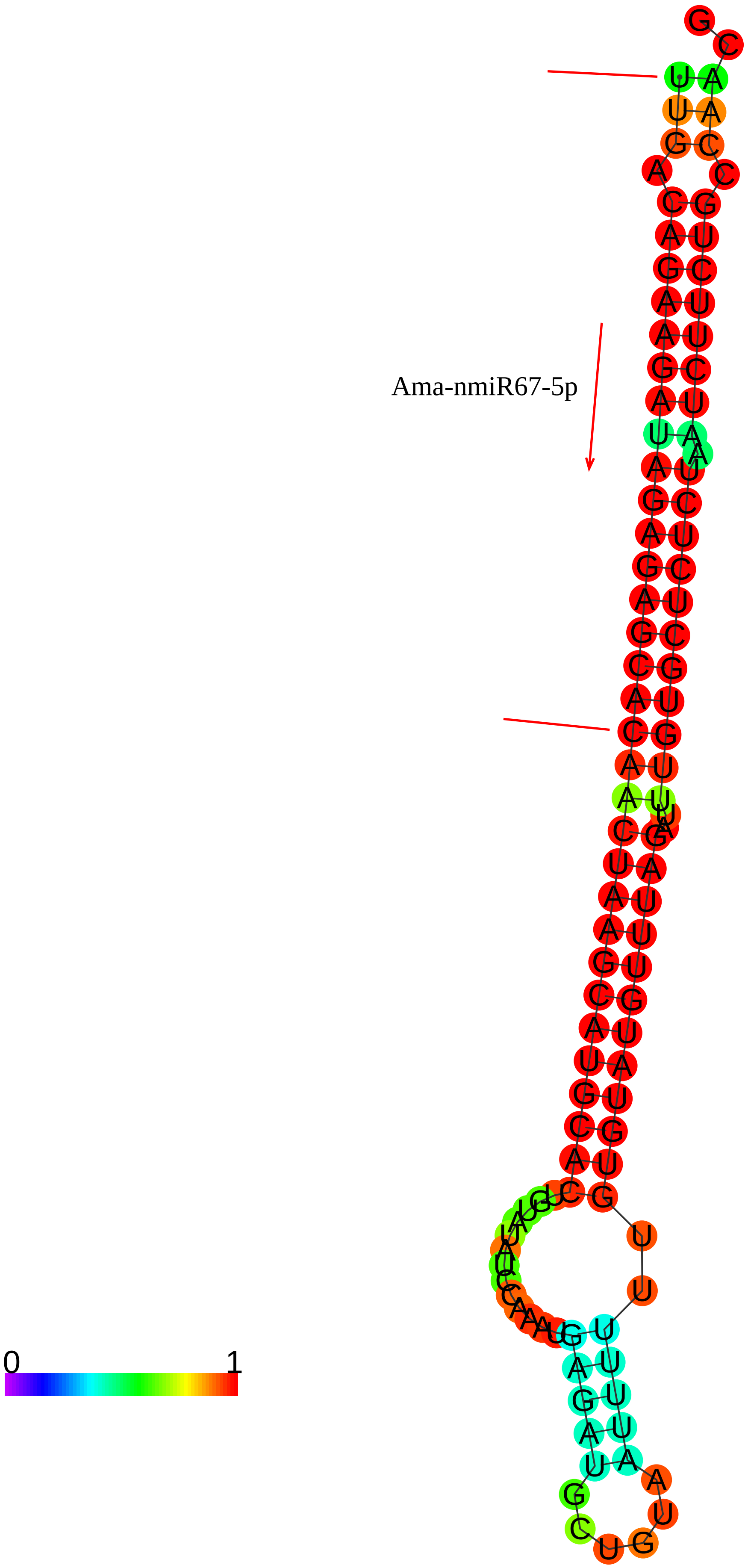
Ama-nmiR67-5p
0
1

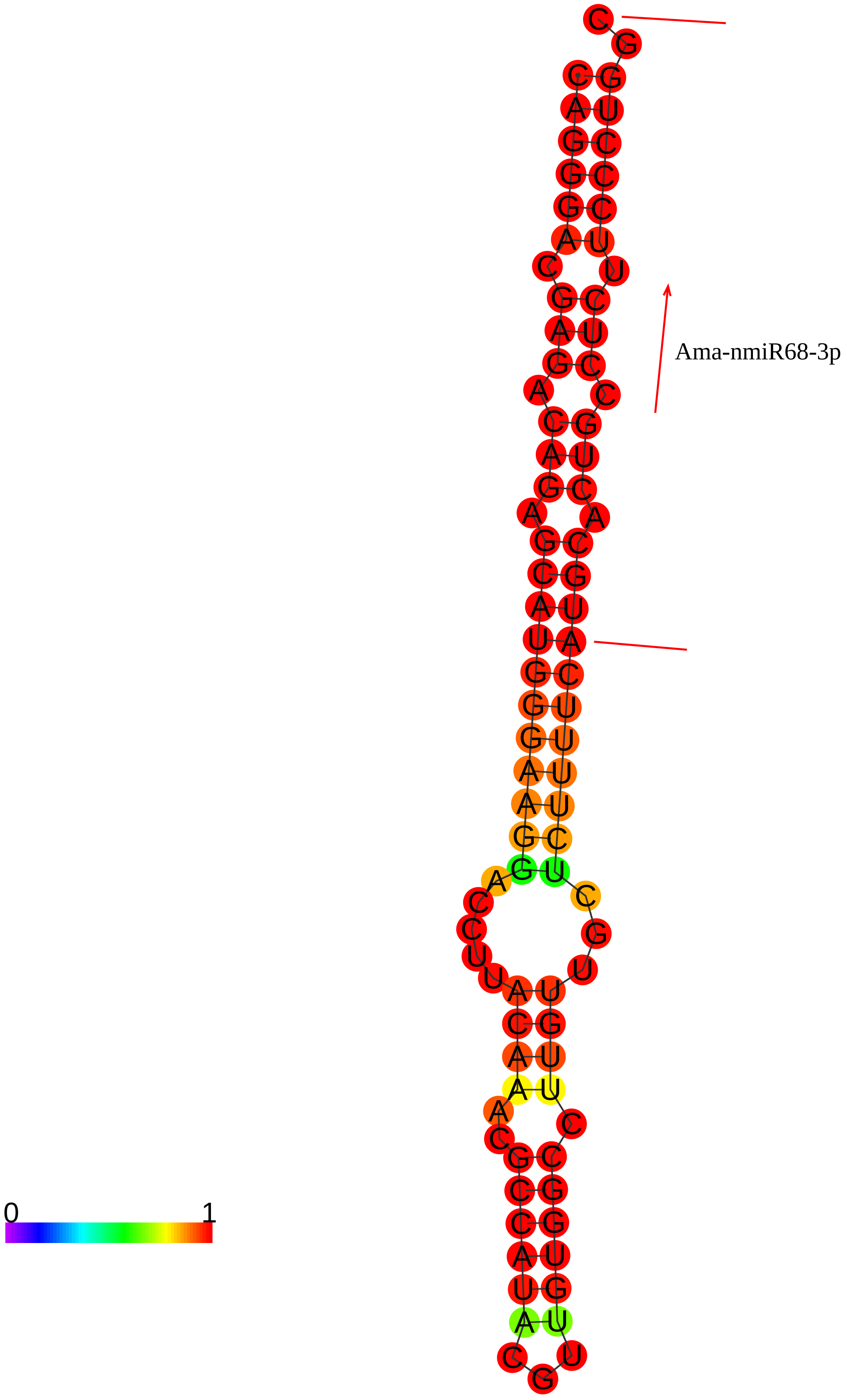
Ama-nmiR68-3p
0
1

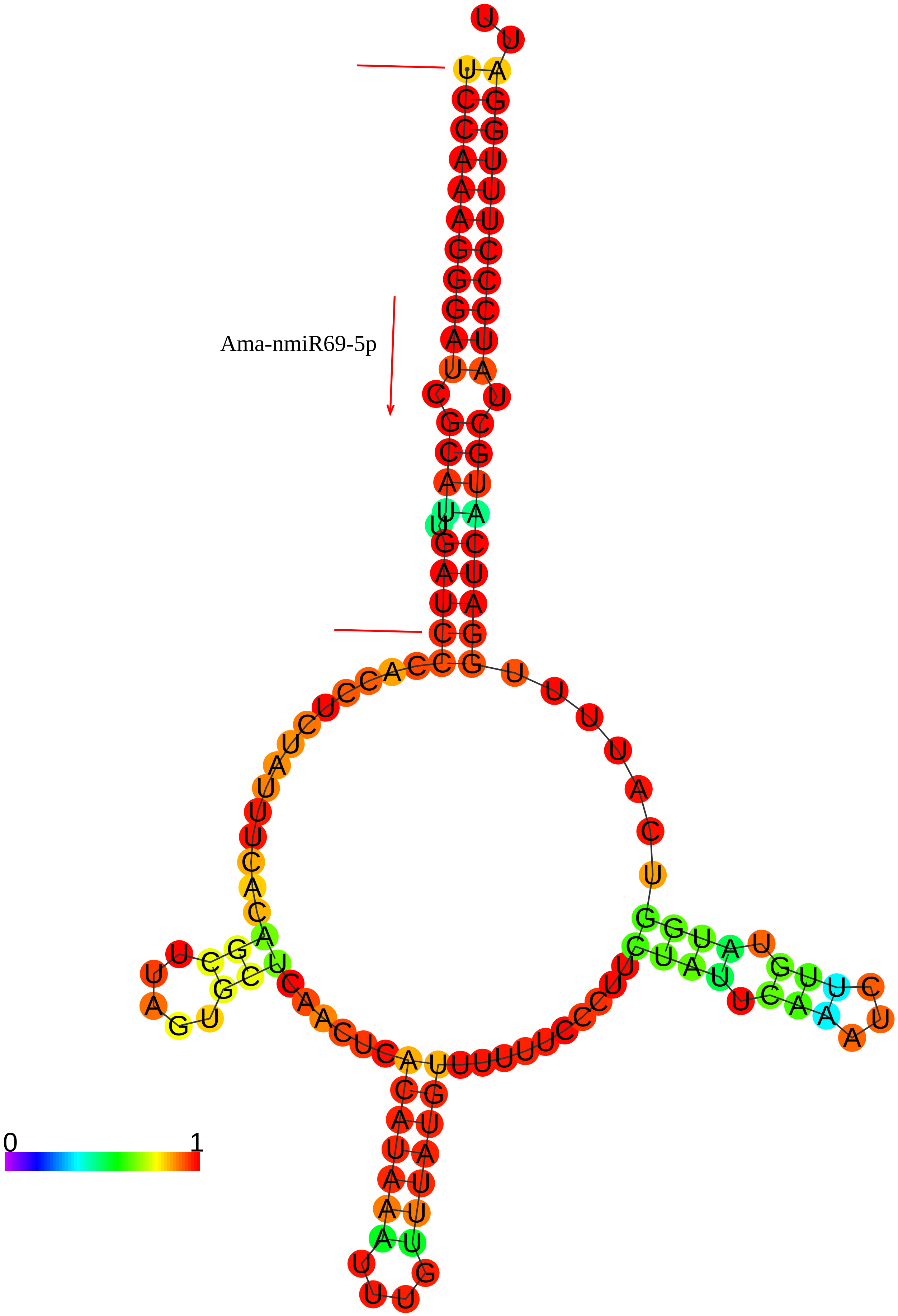
Ama-nmiR69-5p
0
1

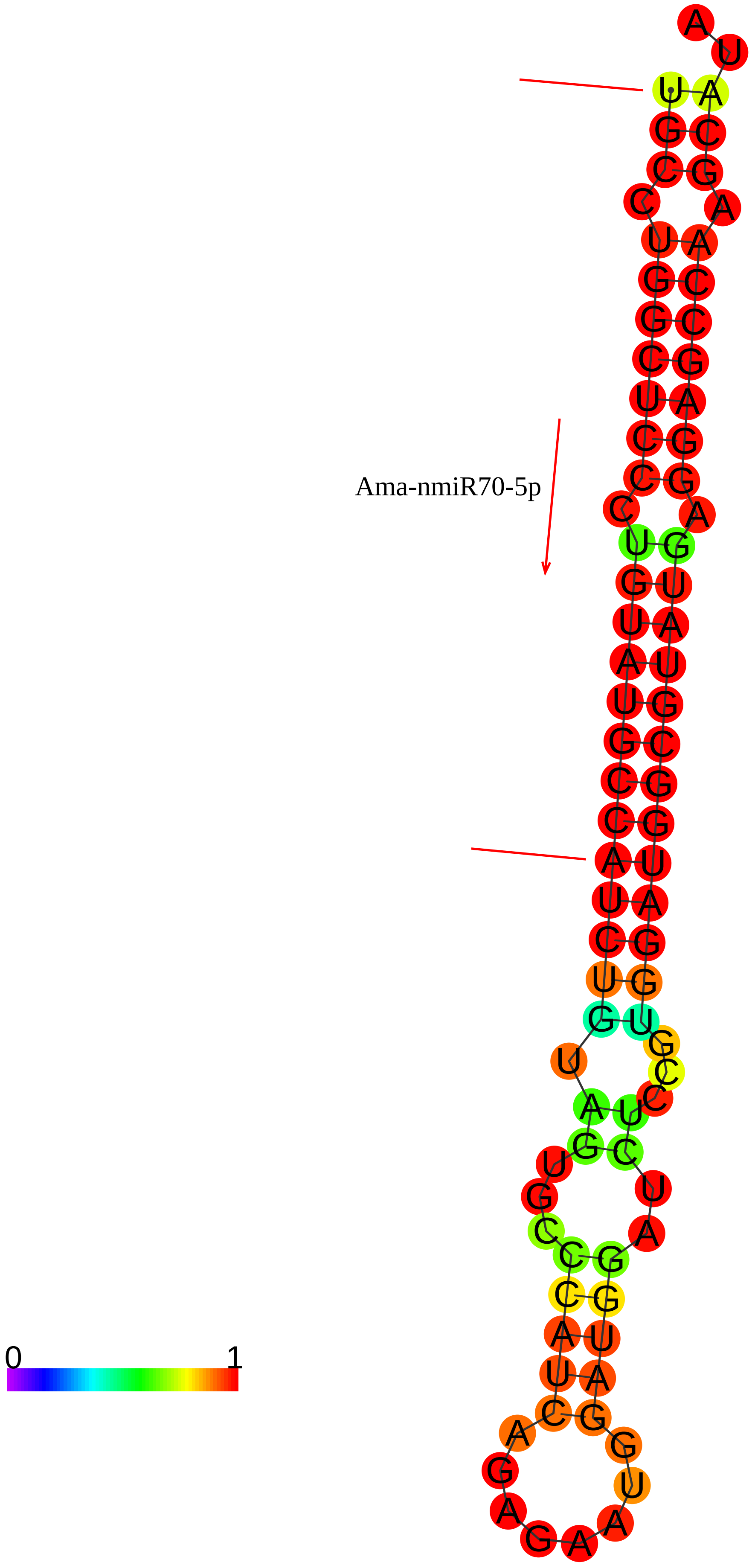
Ama-nmiR70-5p
0
1

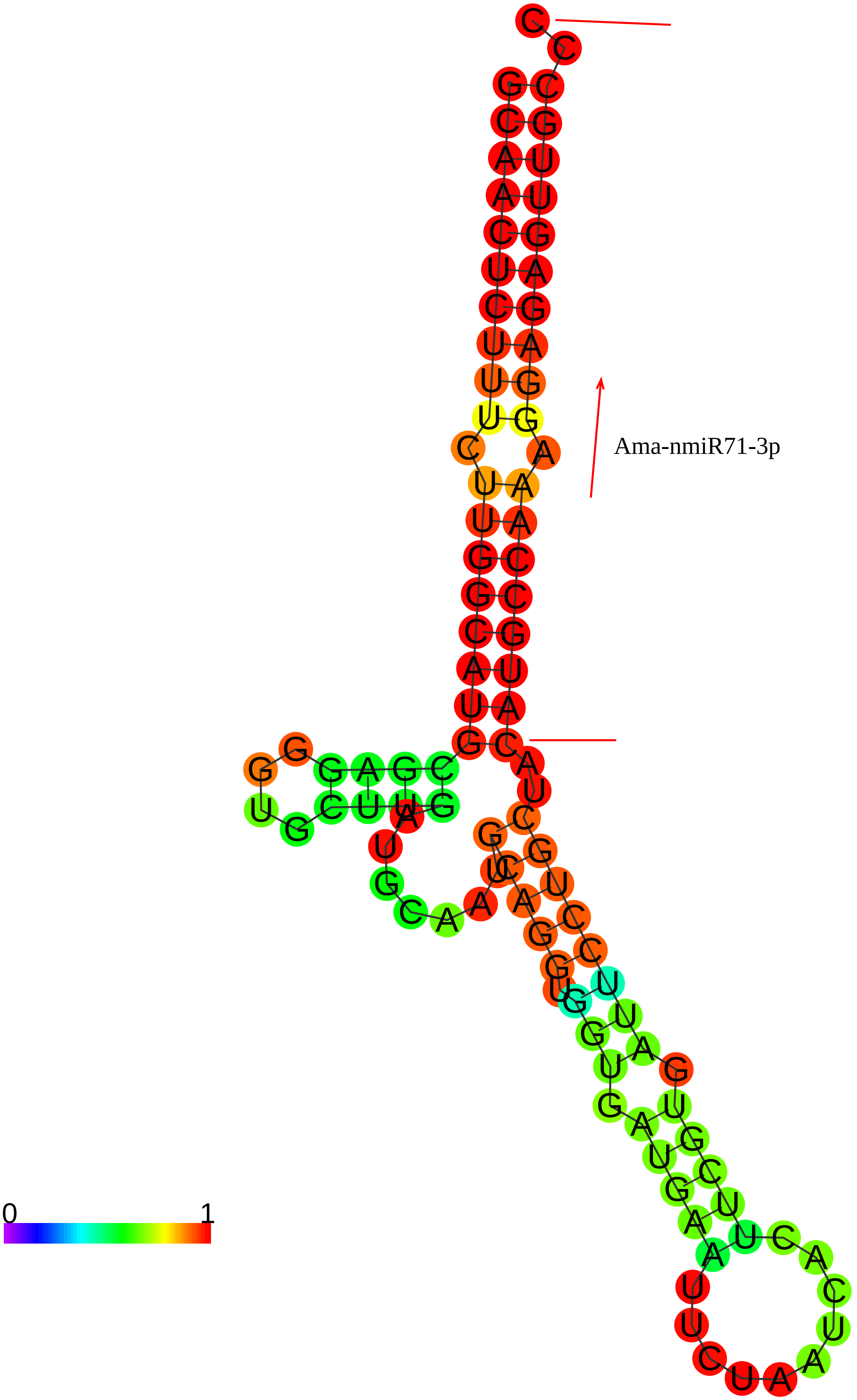
Ama-nmiR71-3p
0
1

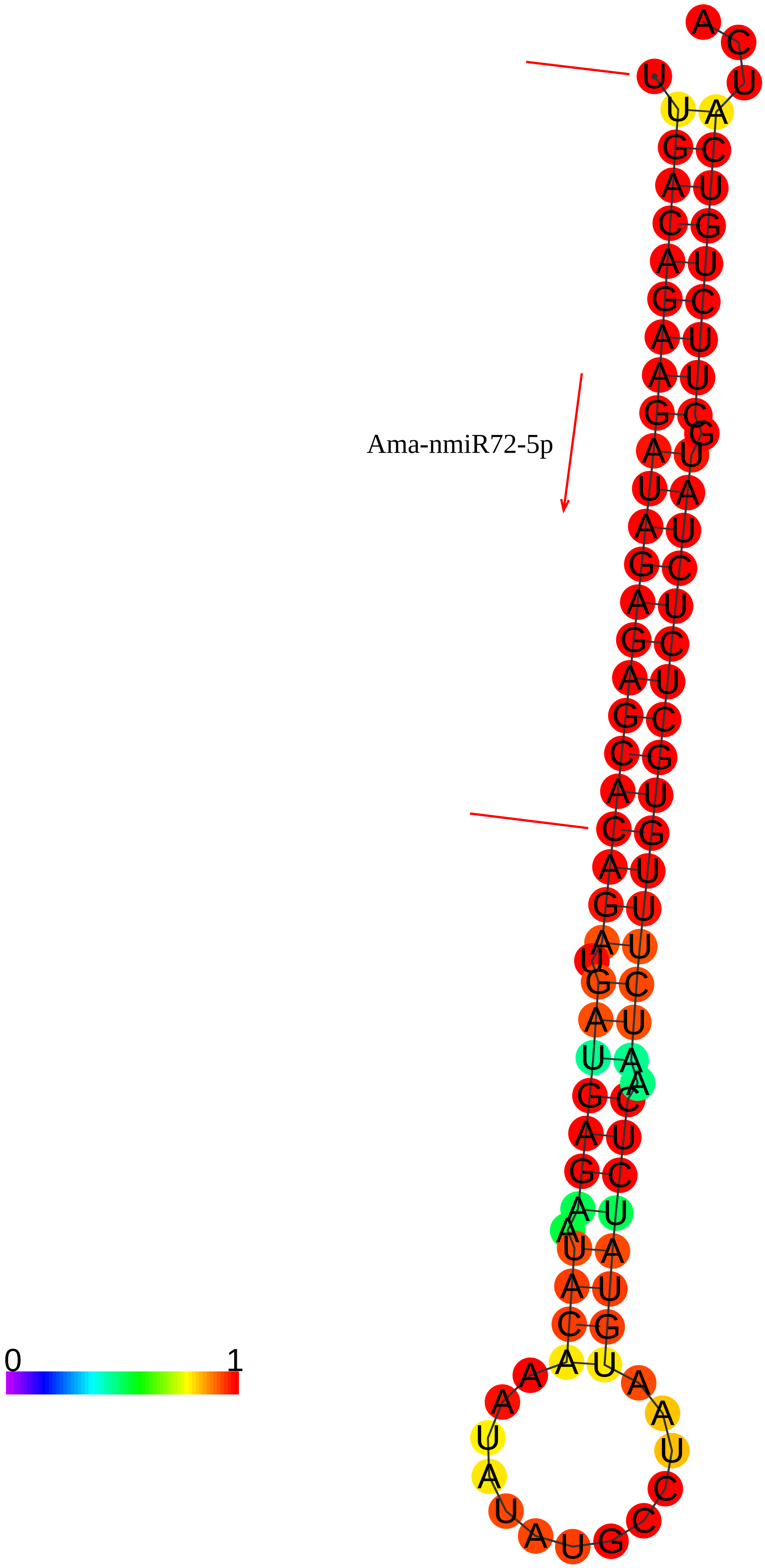
Ama-nmiR72-5p
0
1

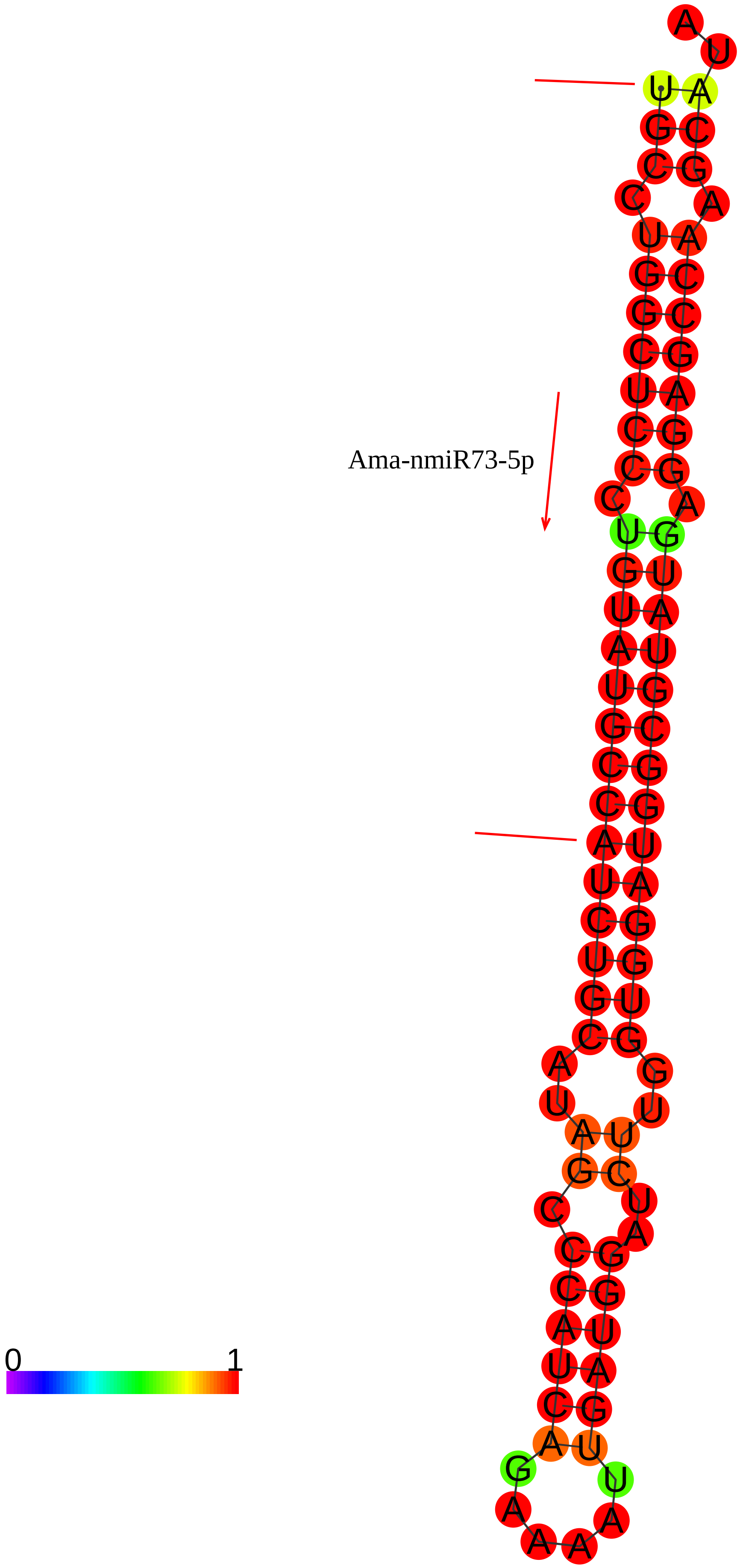
Ama-nmiR73-5p
0
1

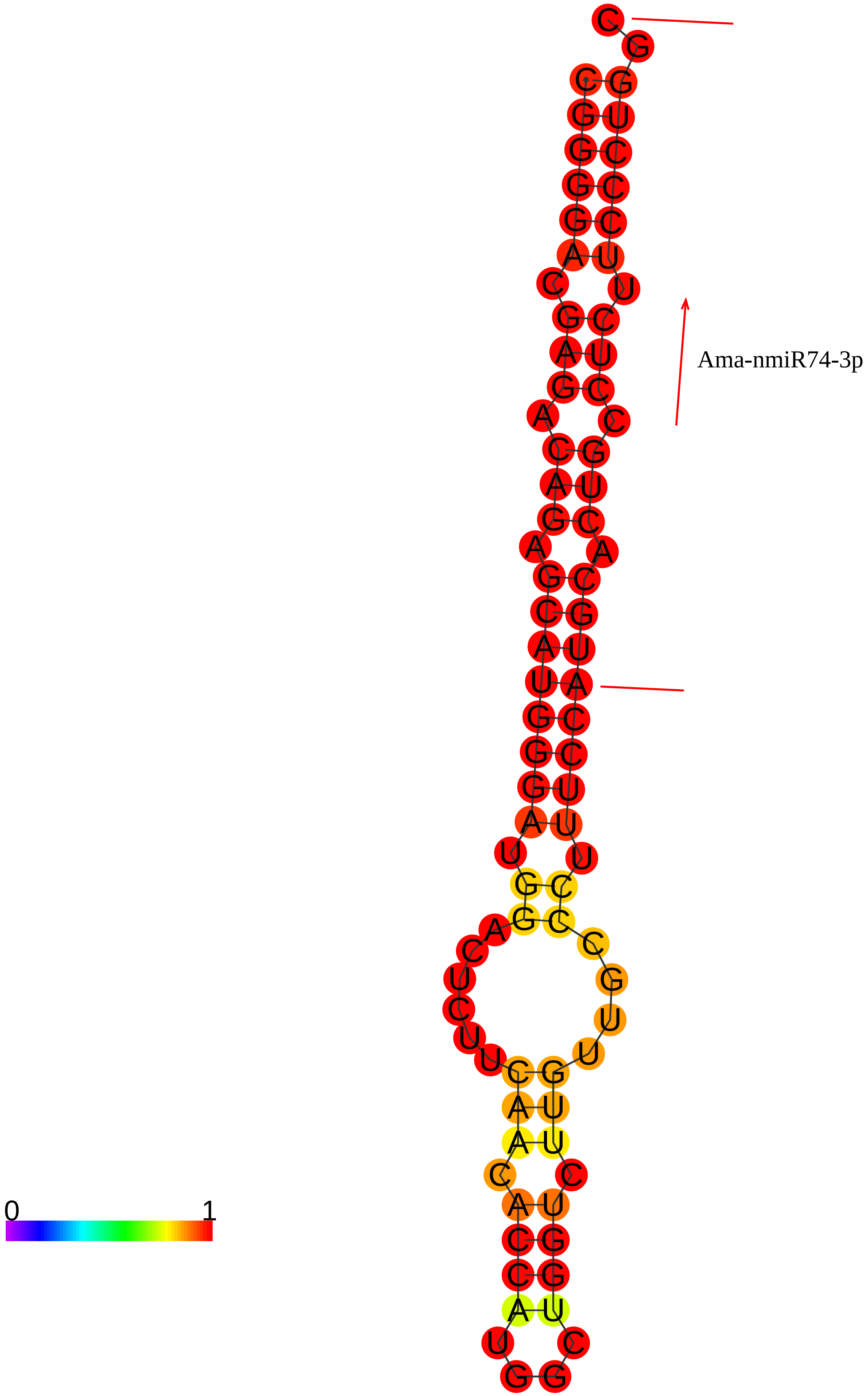
Ama-nmiR74-3p
0
1

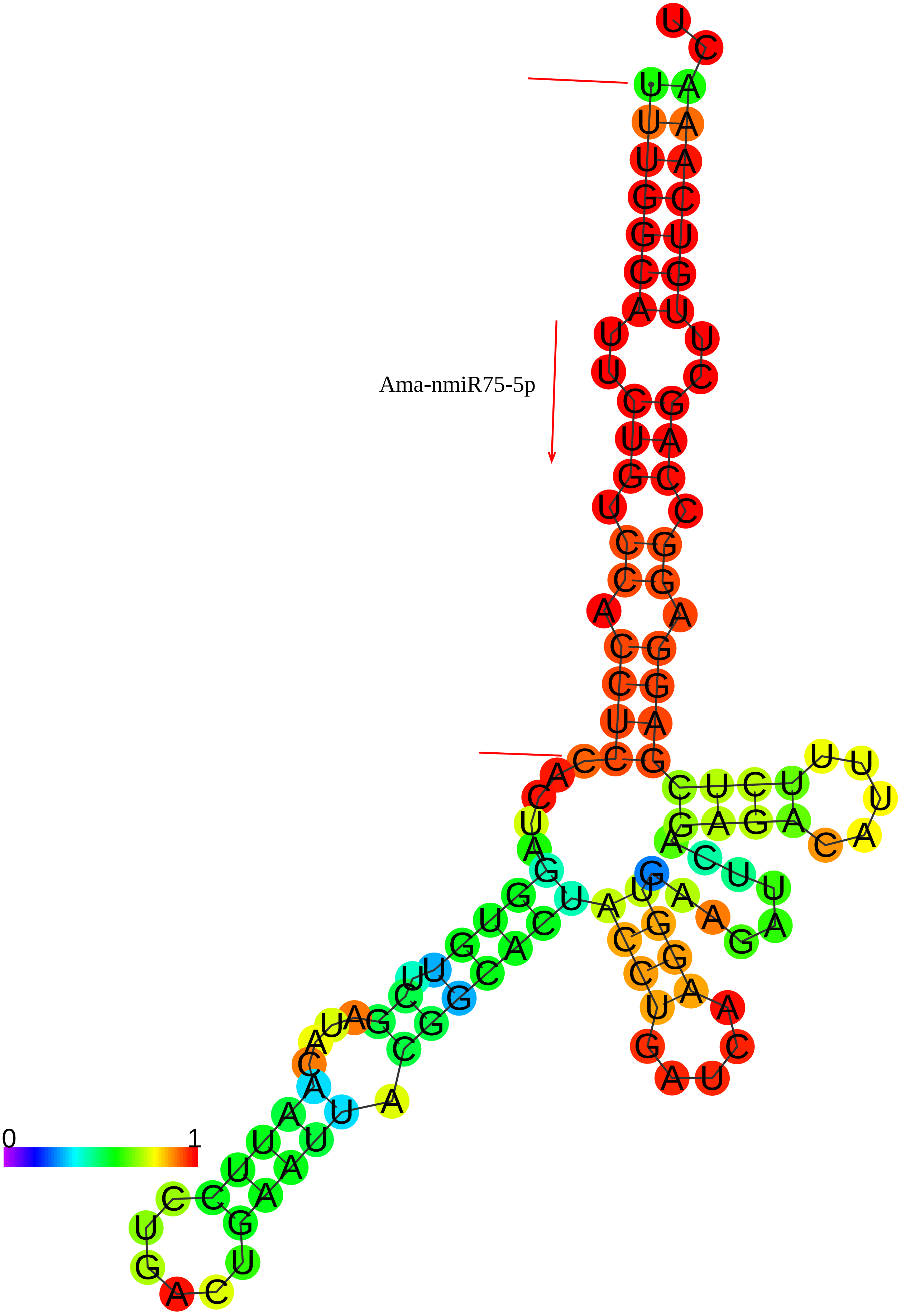
Ama-nmiR75-5p
0
1

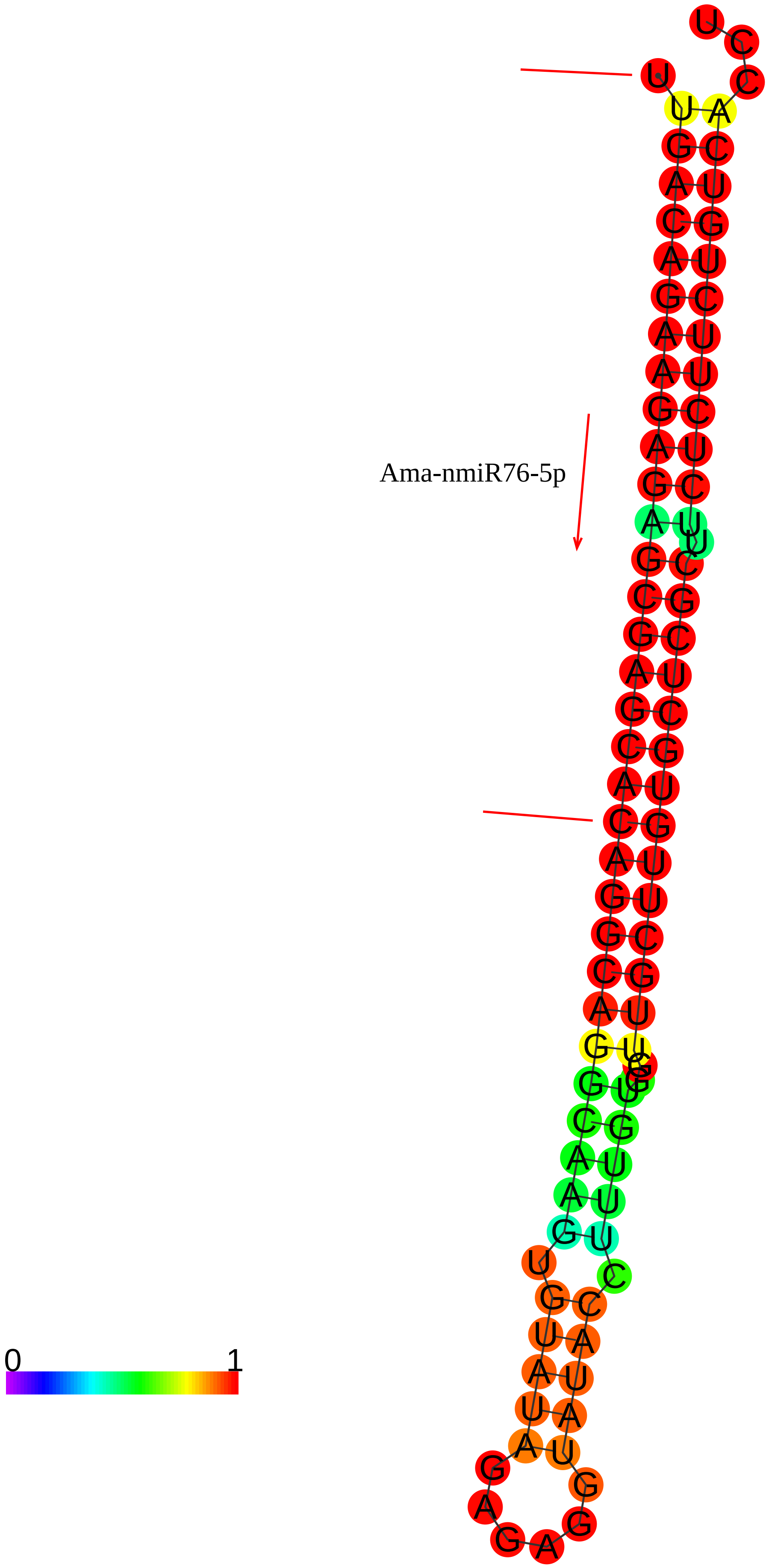
Ama-nmiR76-5p
0
1

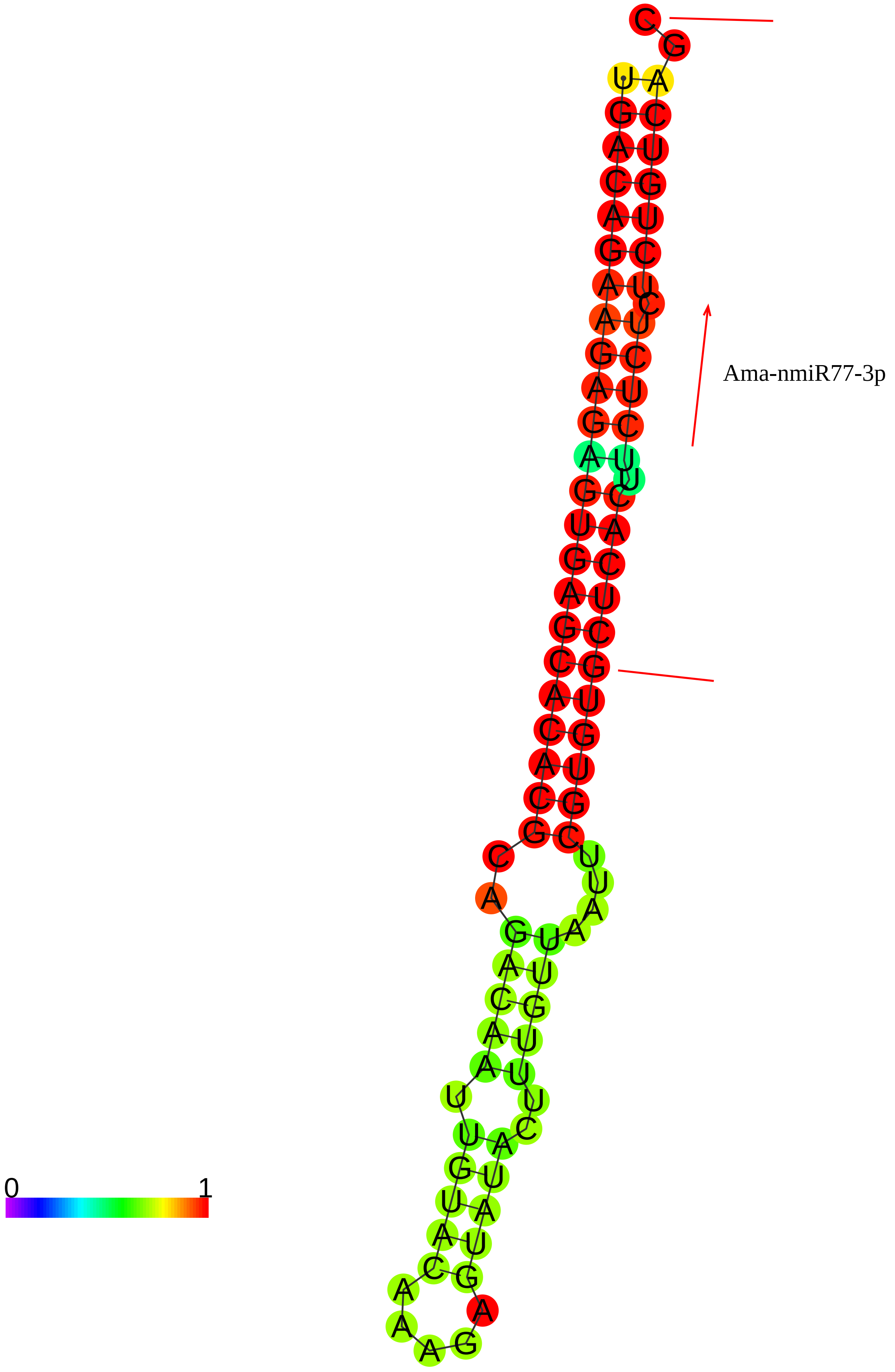
Ama-nmiR77-3p
0
1

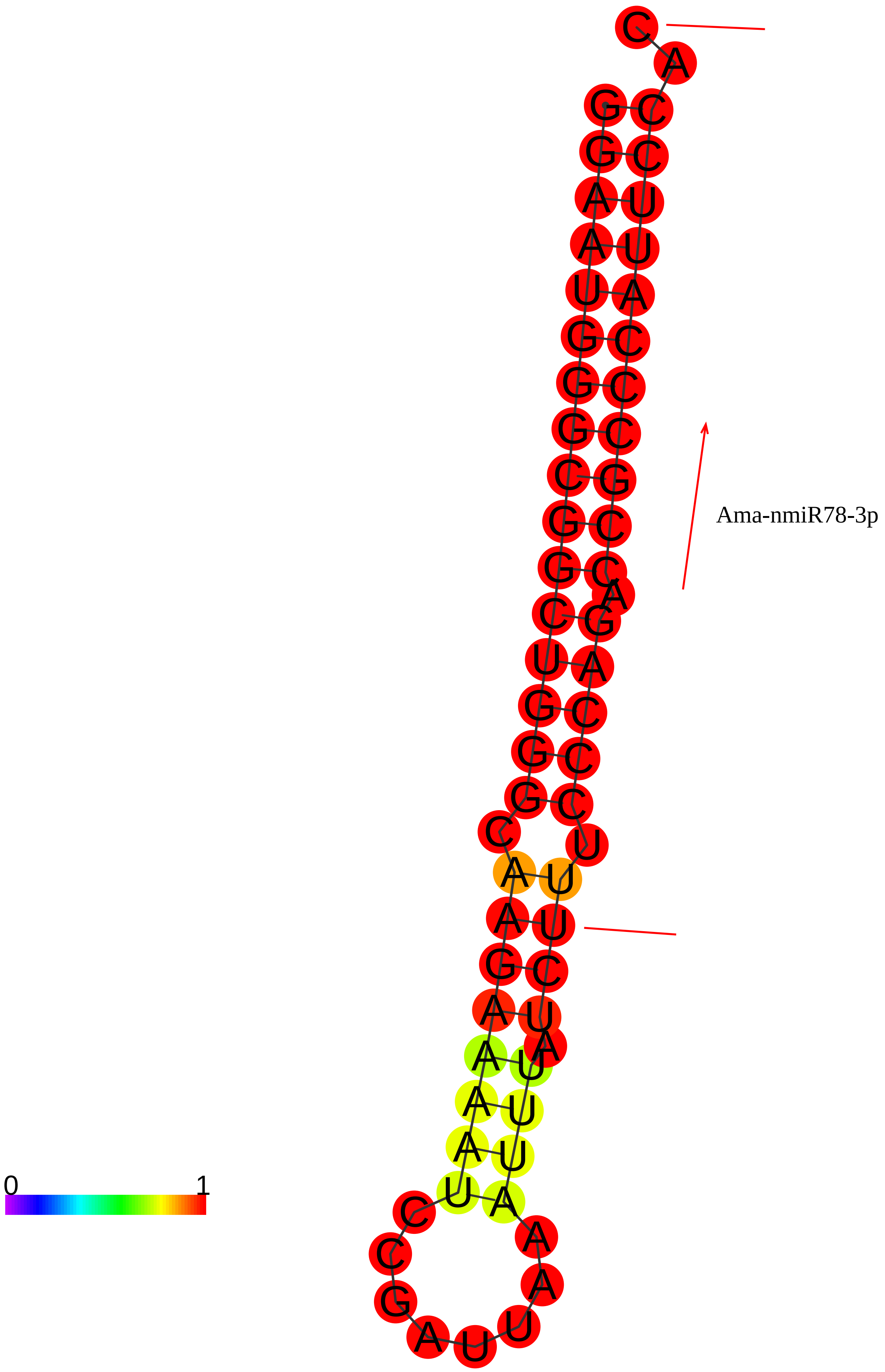
Ama-nmiR78-3p
0
1

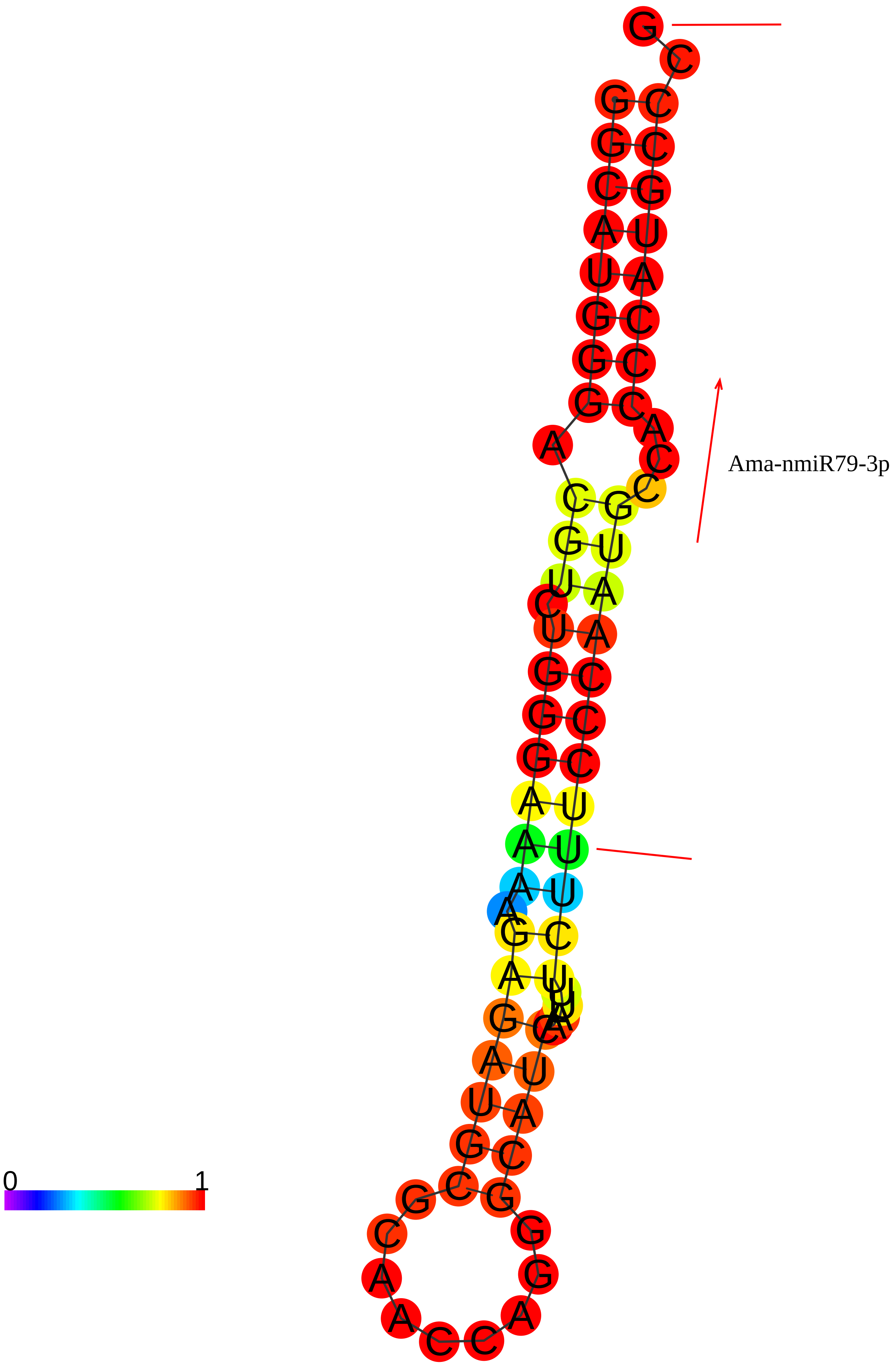
Ama-nmiR79-3p
0
1

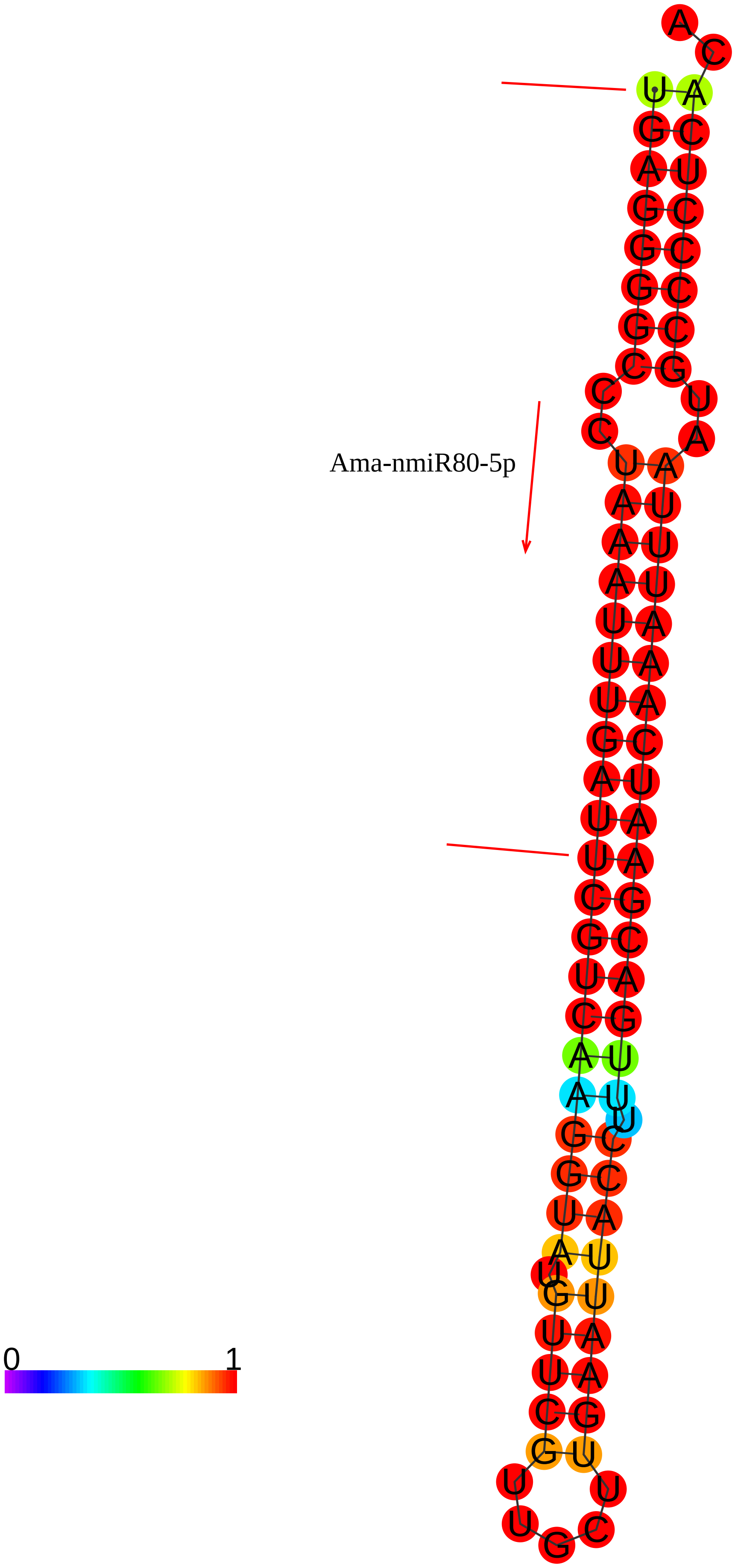
Ama-nmiR80-5p
0
1

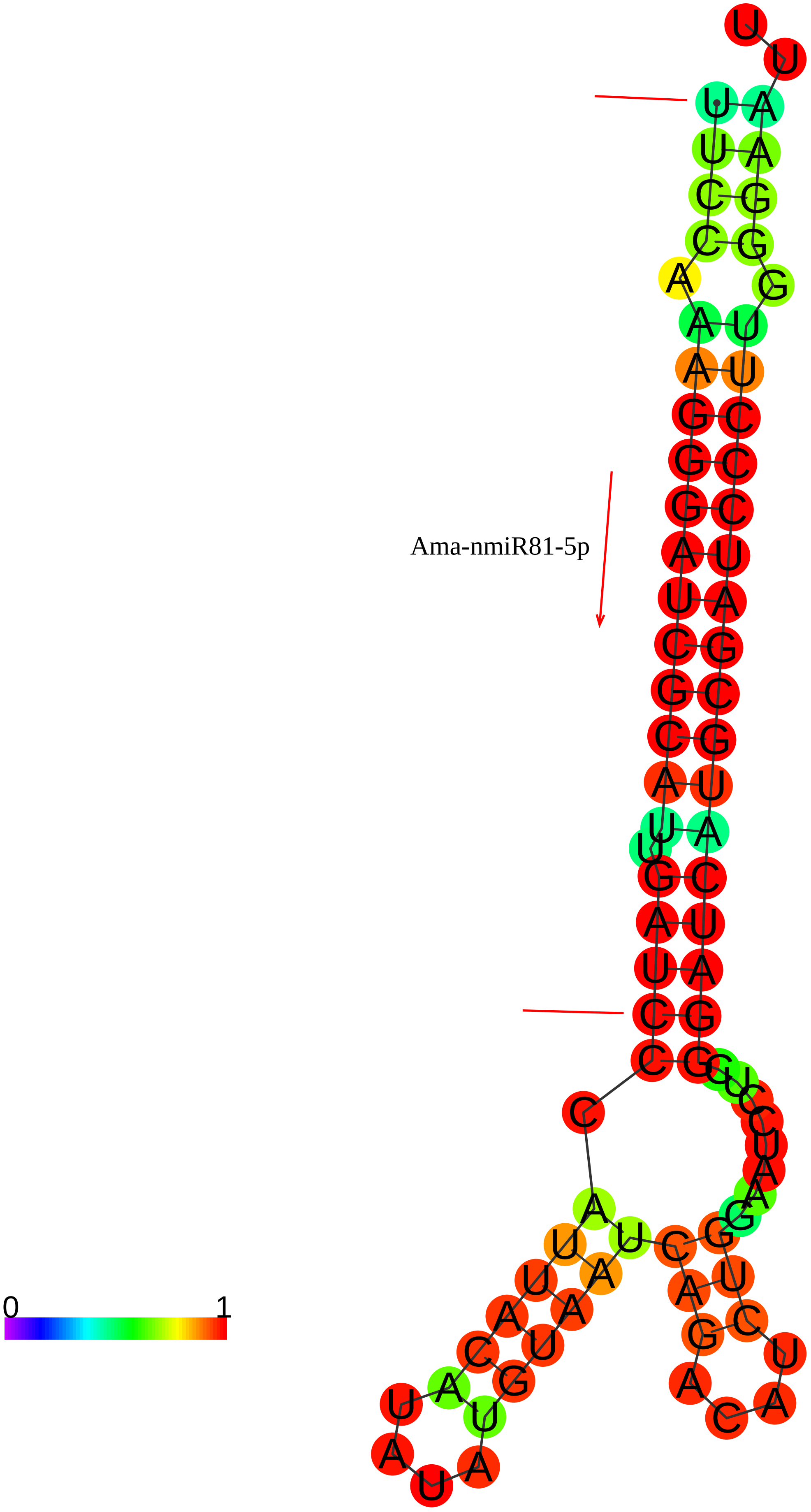
Ama-nmiR81-5p
0
1

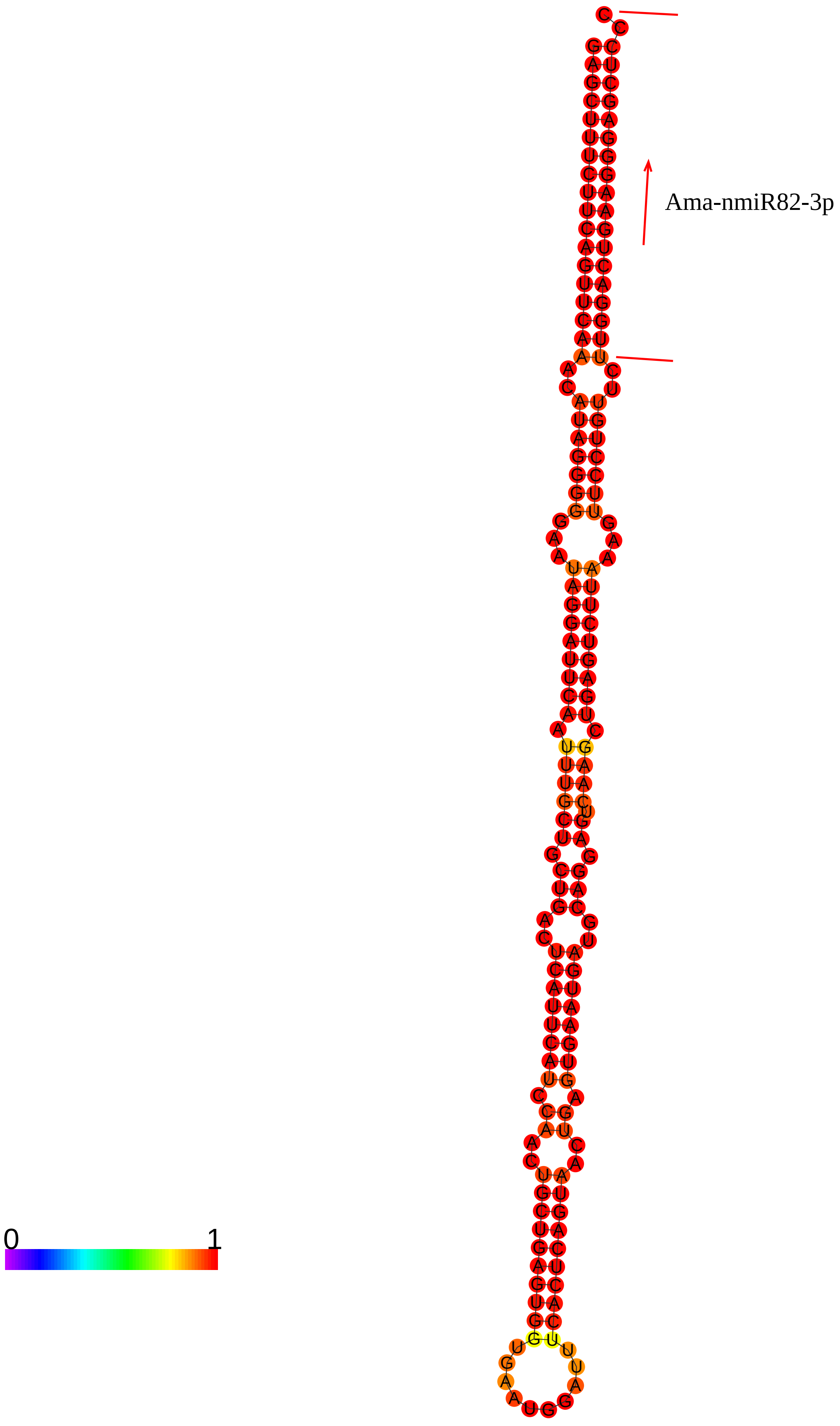
Ama-nmiR82-3p
0
1

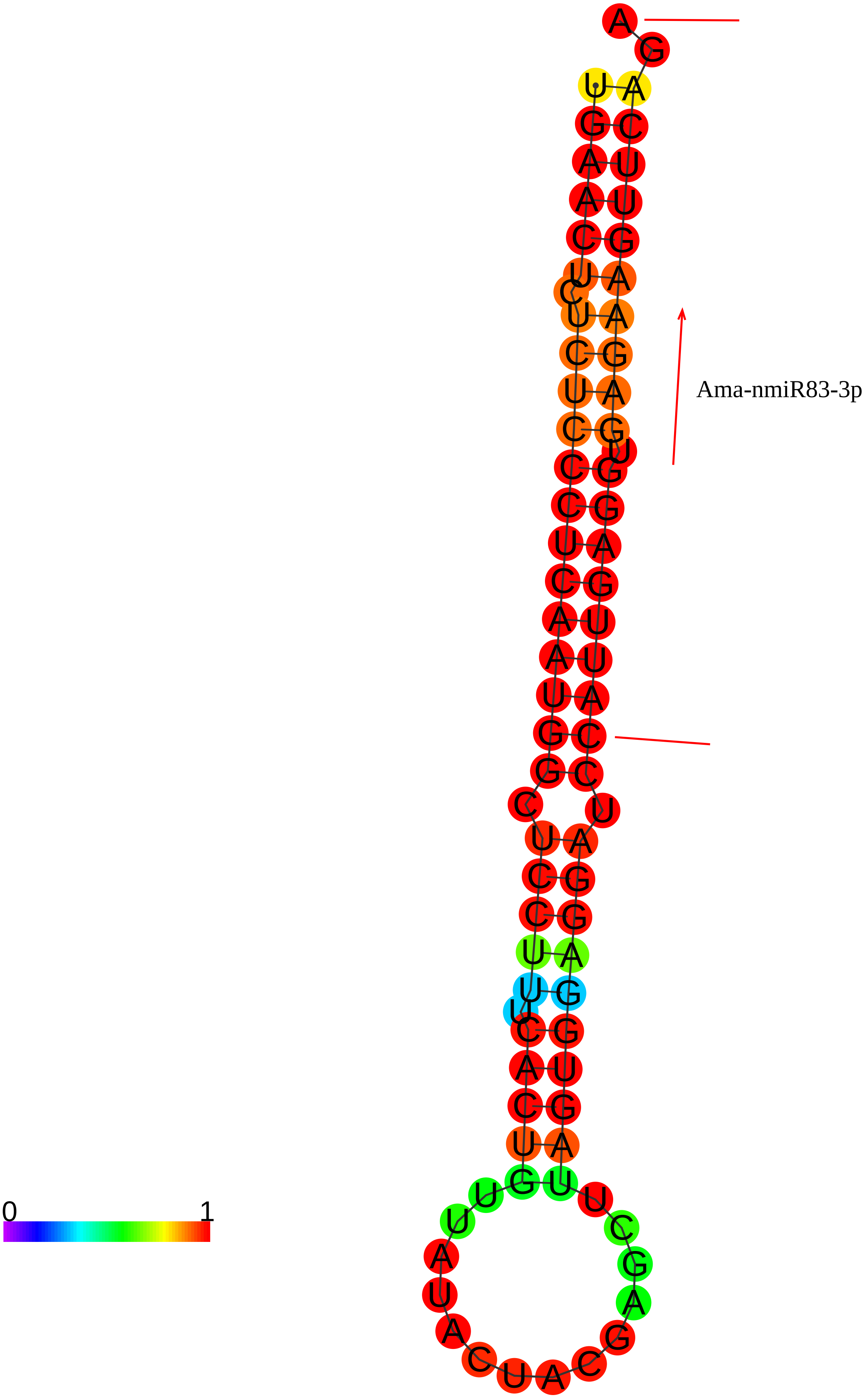
Ama-nmiR83-3p
0
1

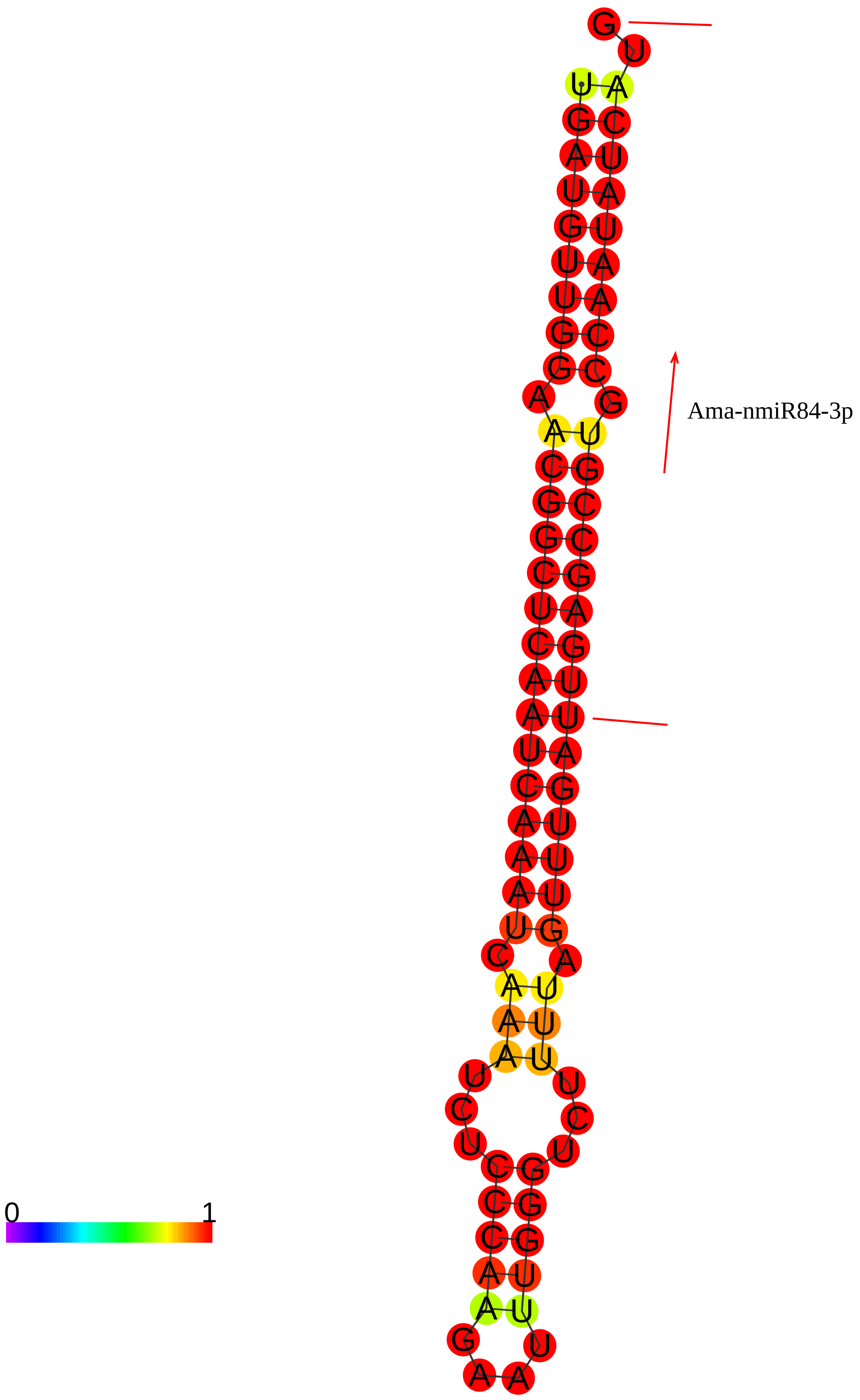

0
1
Ama-nmiR84-3p

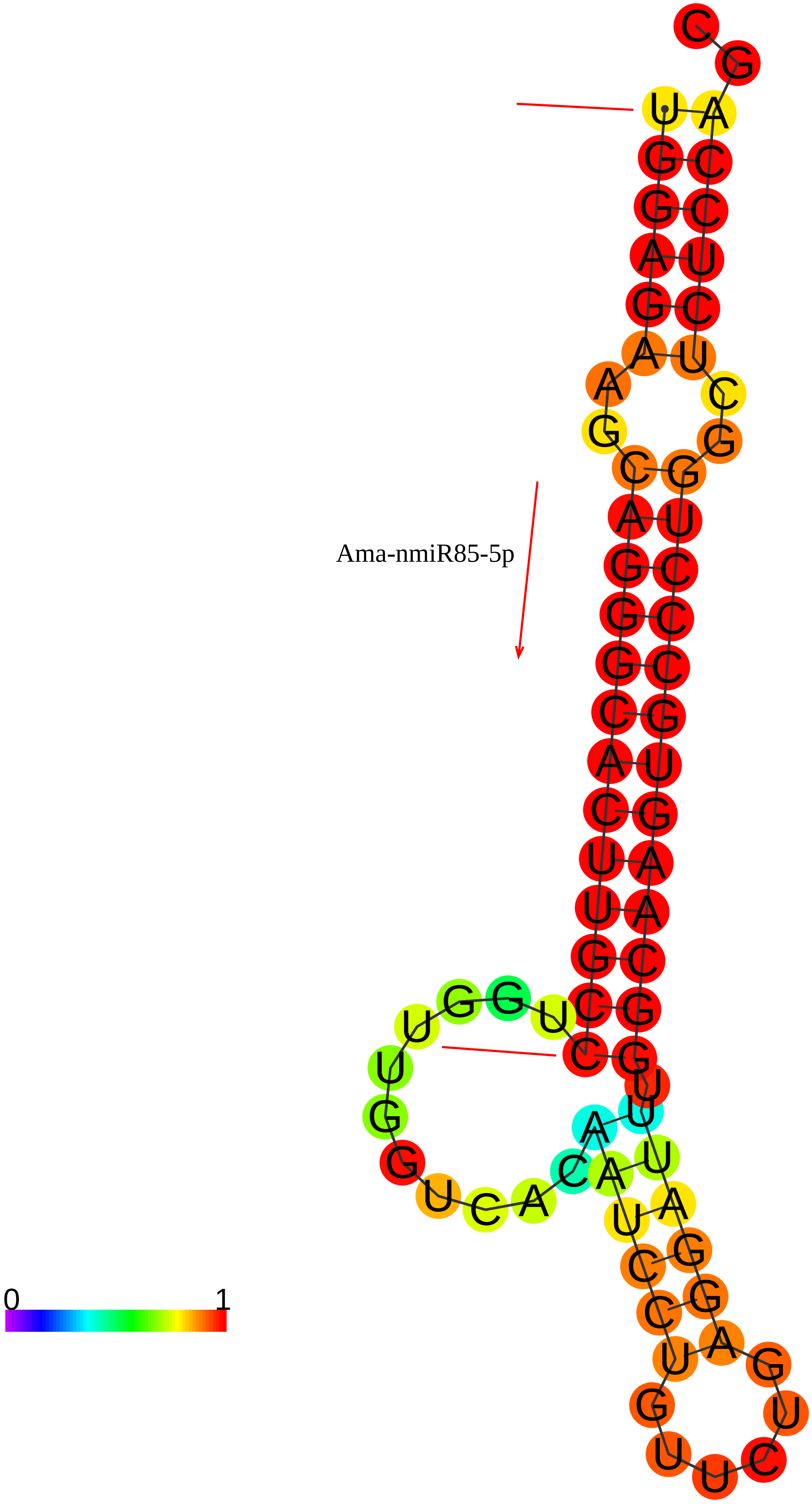
Ama-nmiR85-5p
0
1

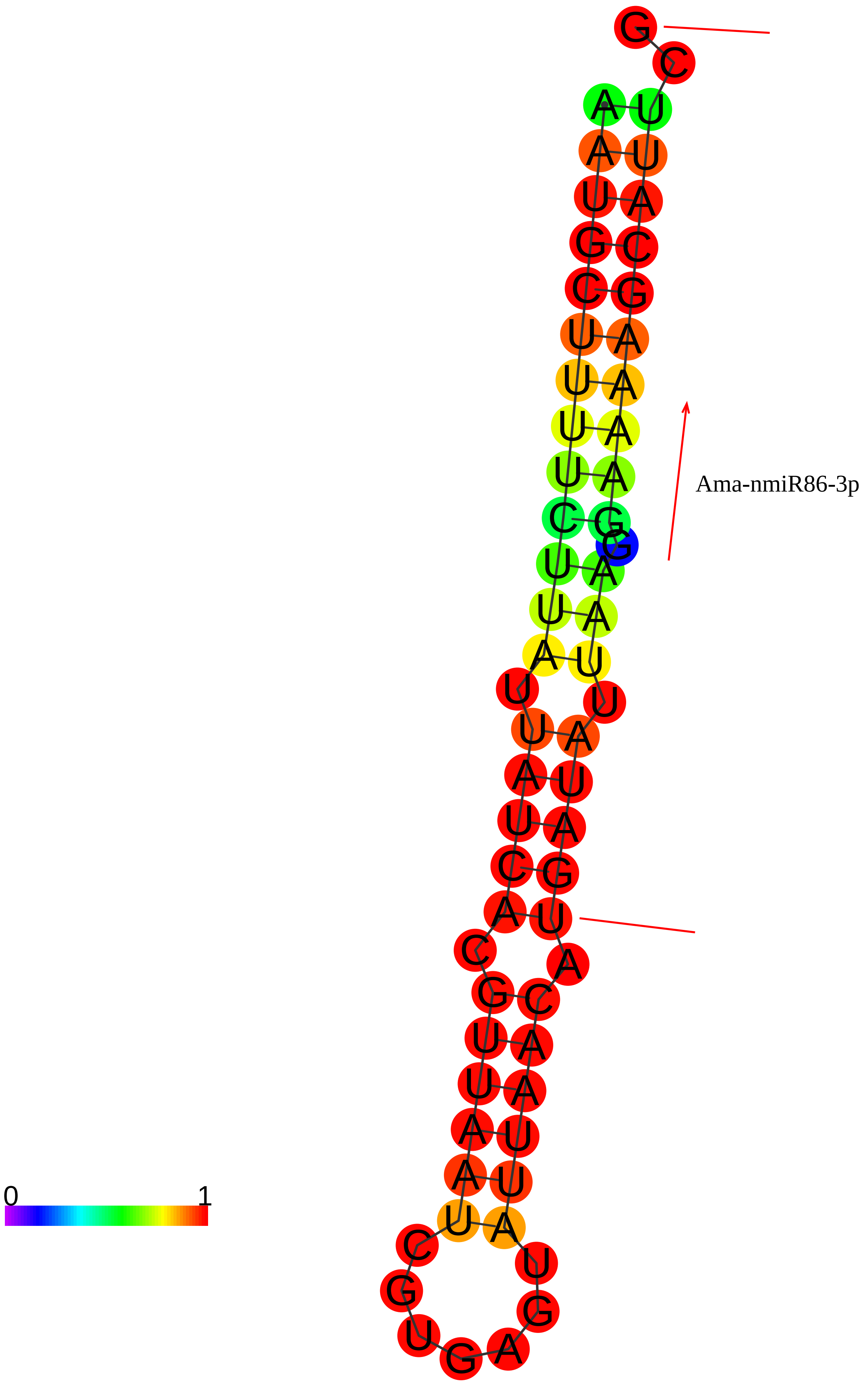
Ama-nmiR86-3p
0
1

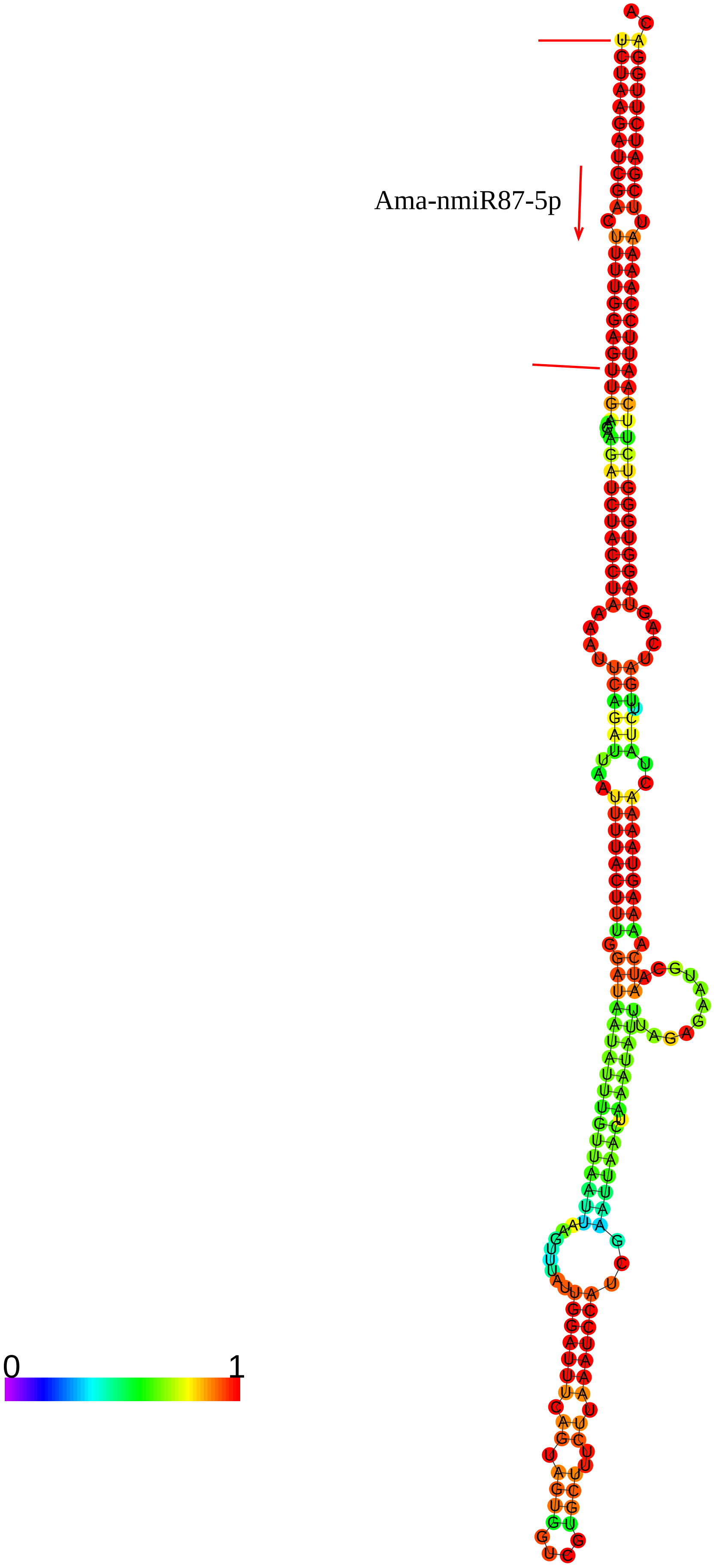
Ama-nmiR87-5p
0
1

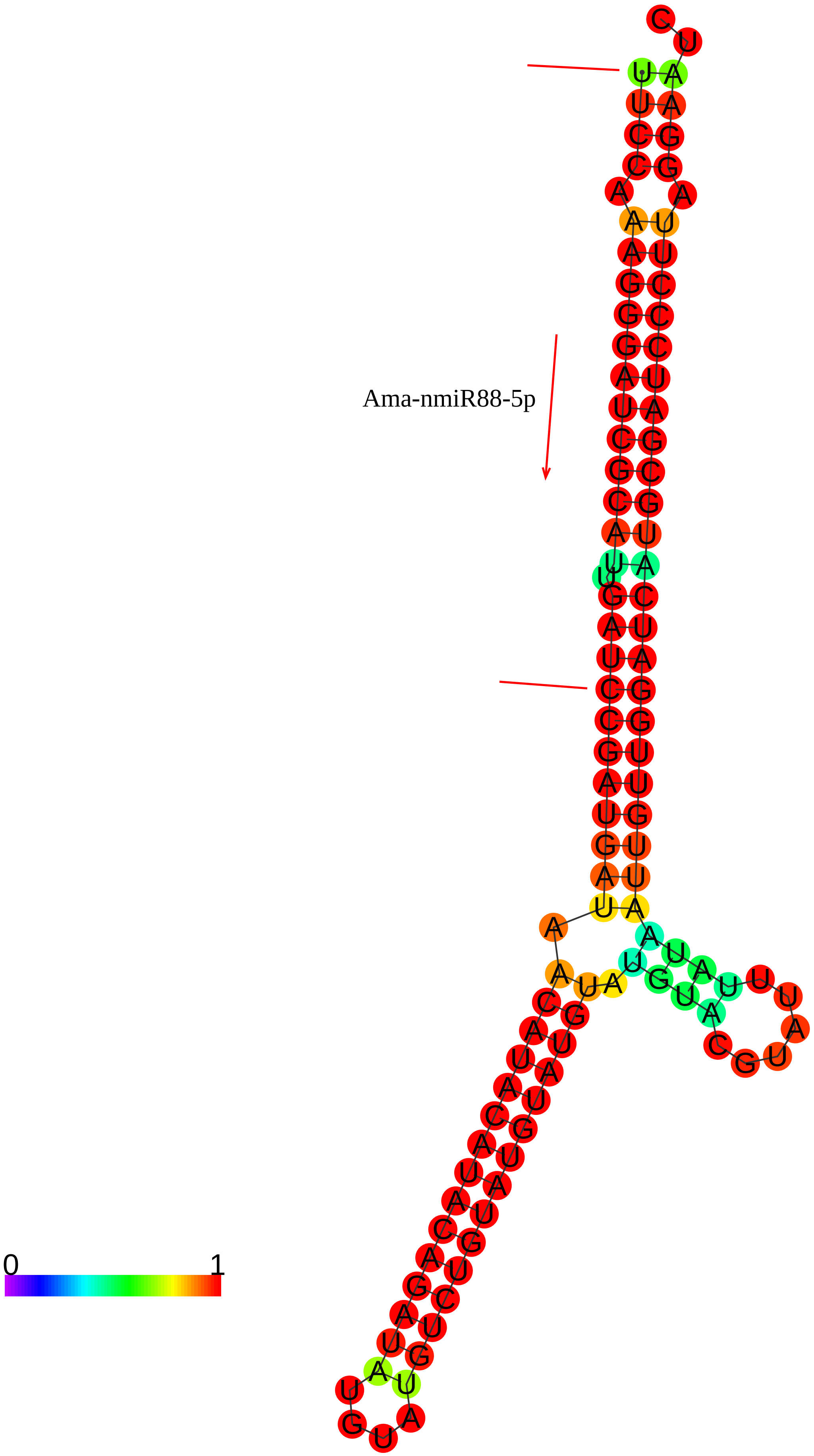
Ama-nmiR88-5p
0
1

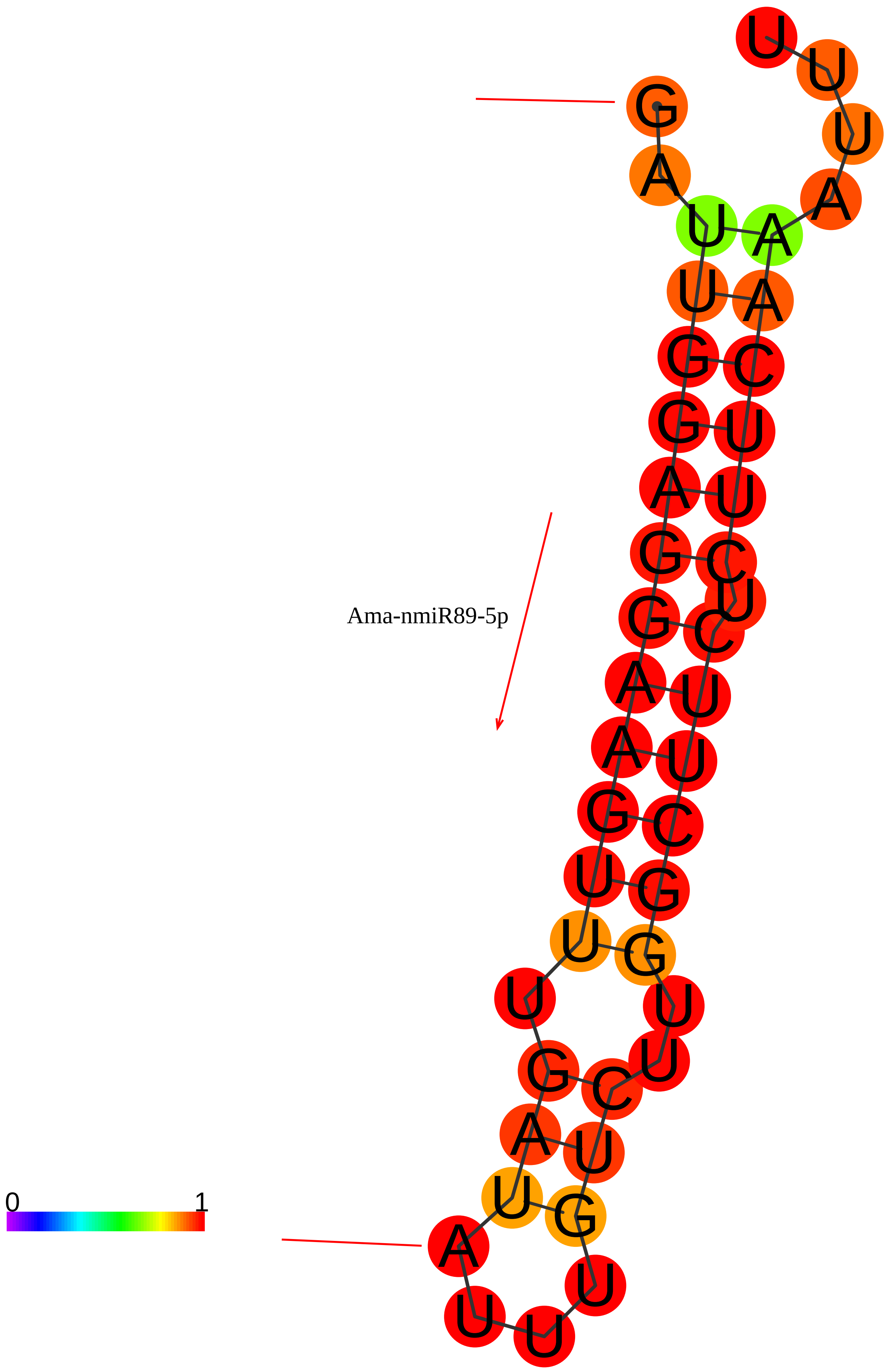

Ama-nmiR89-5p
0
1

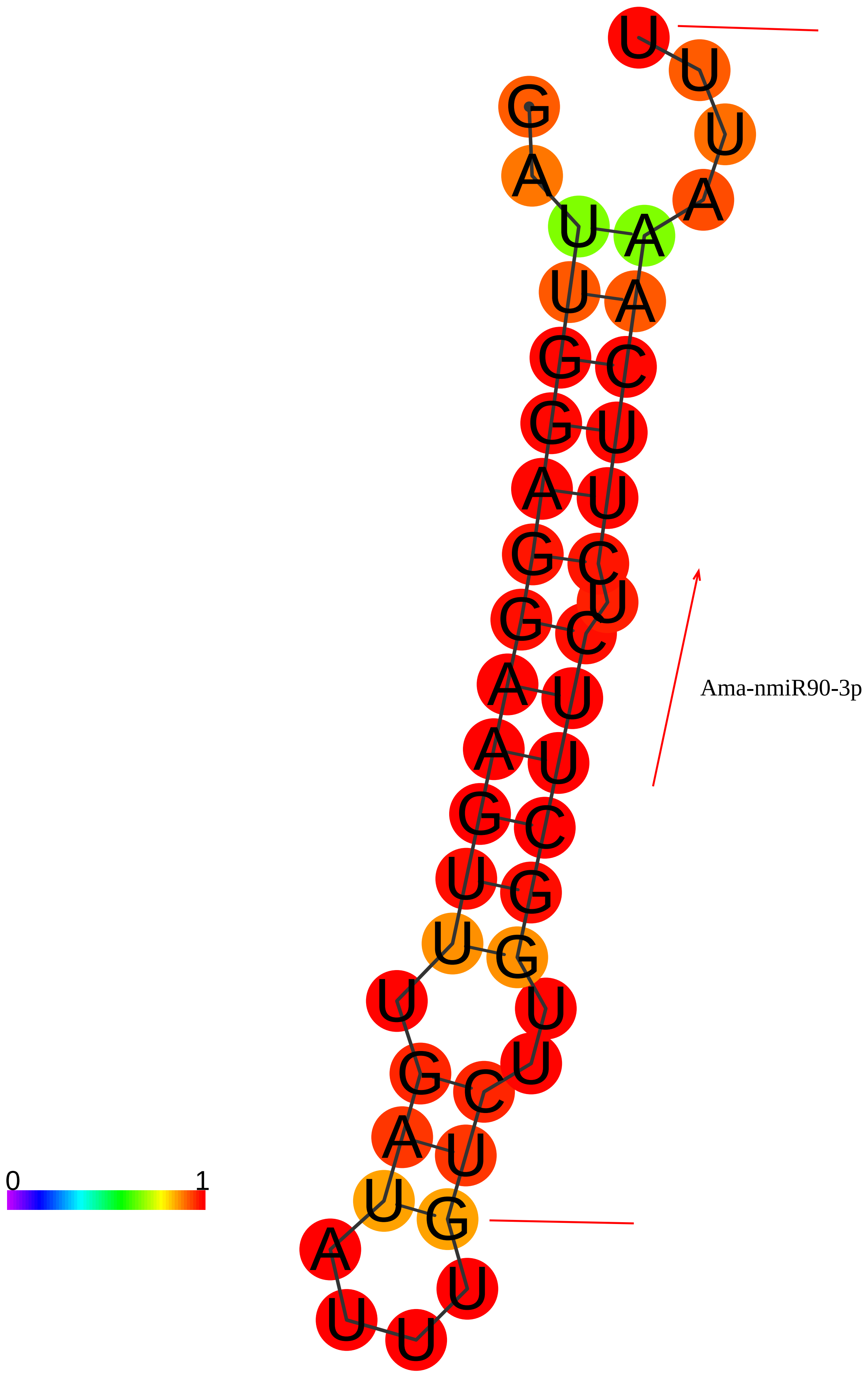
Ama-nmiR90-3p
0
1

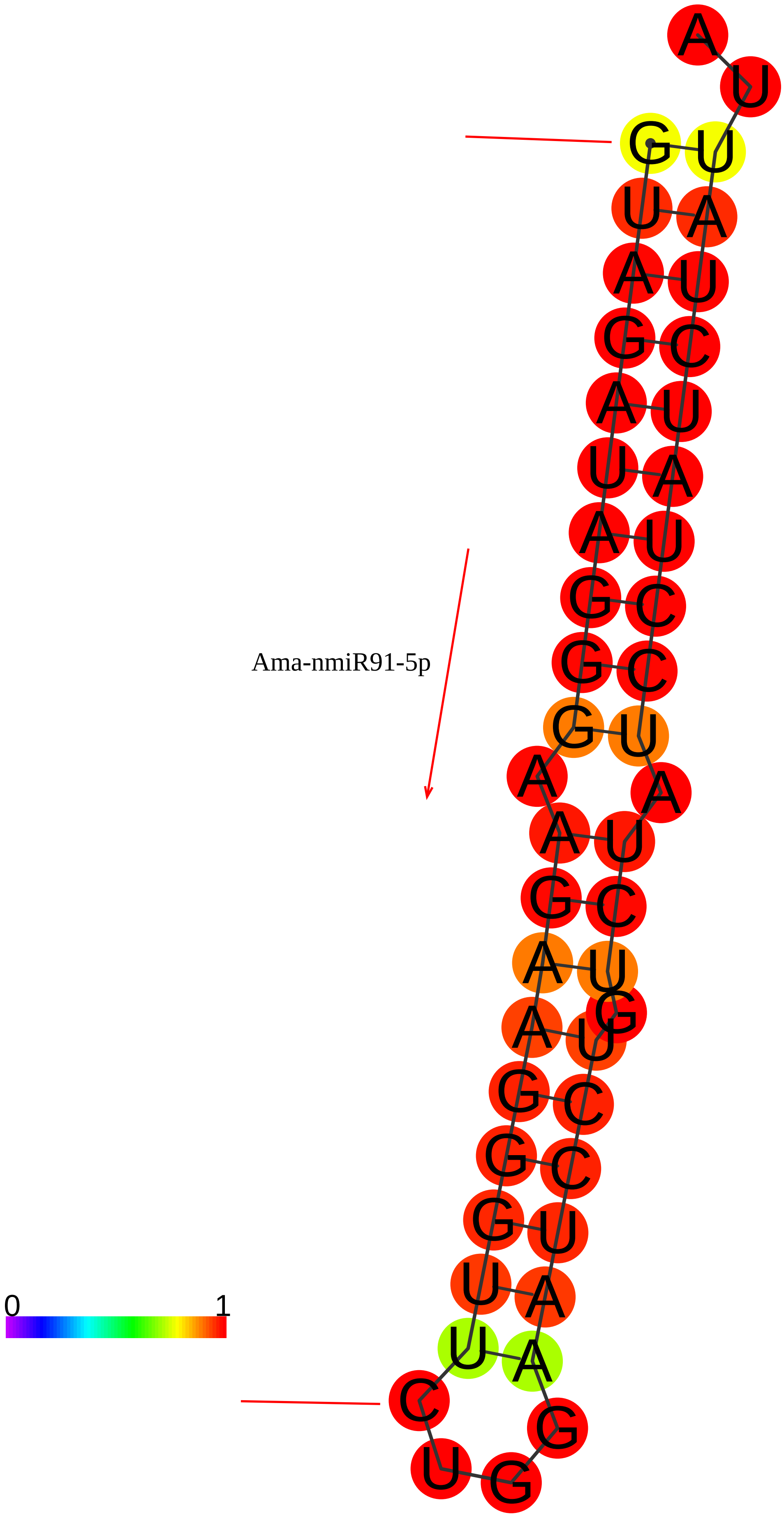

Ama-nmiR91-5p
0
1

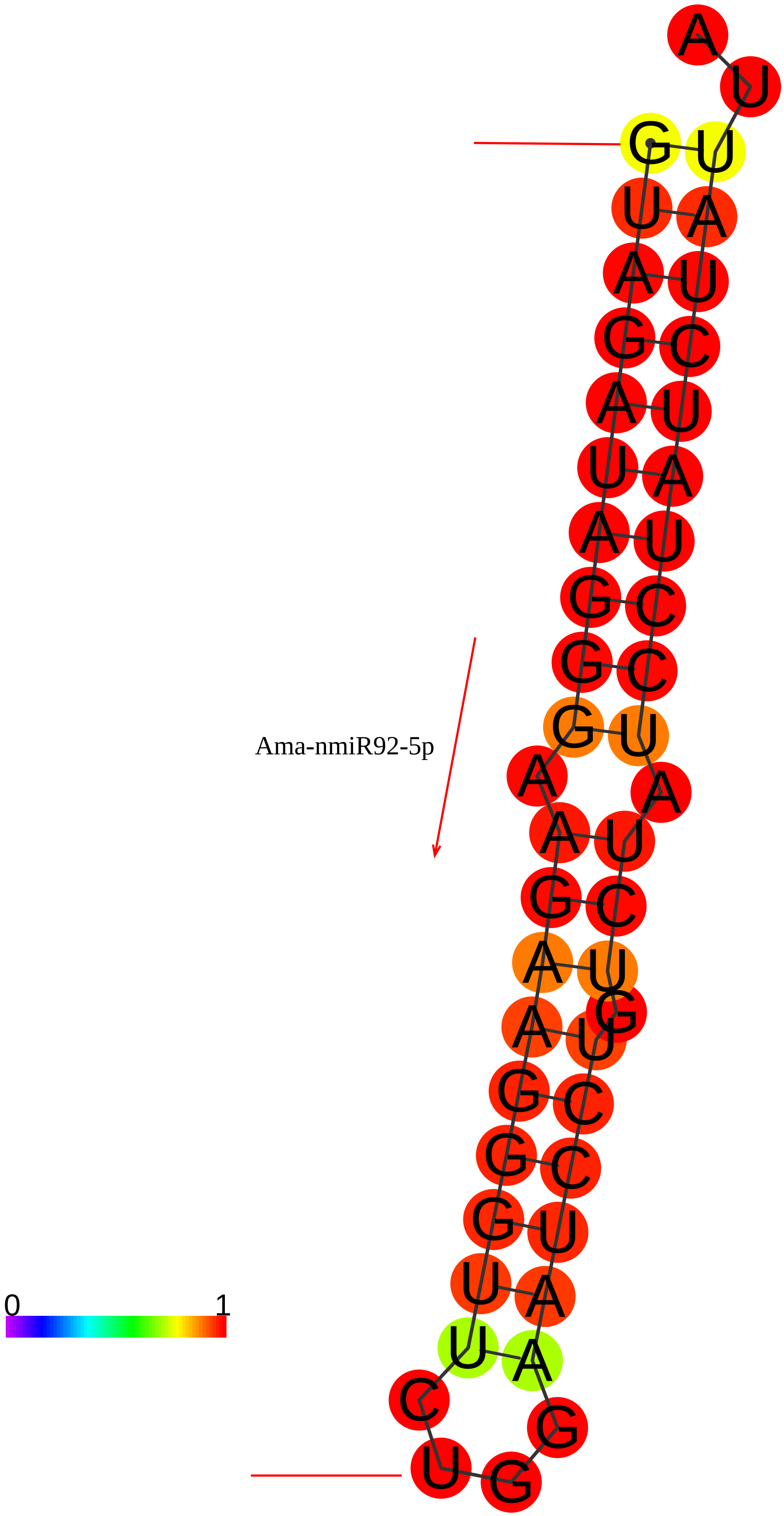
Ama-nmiR92-5p
0
1

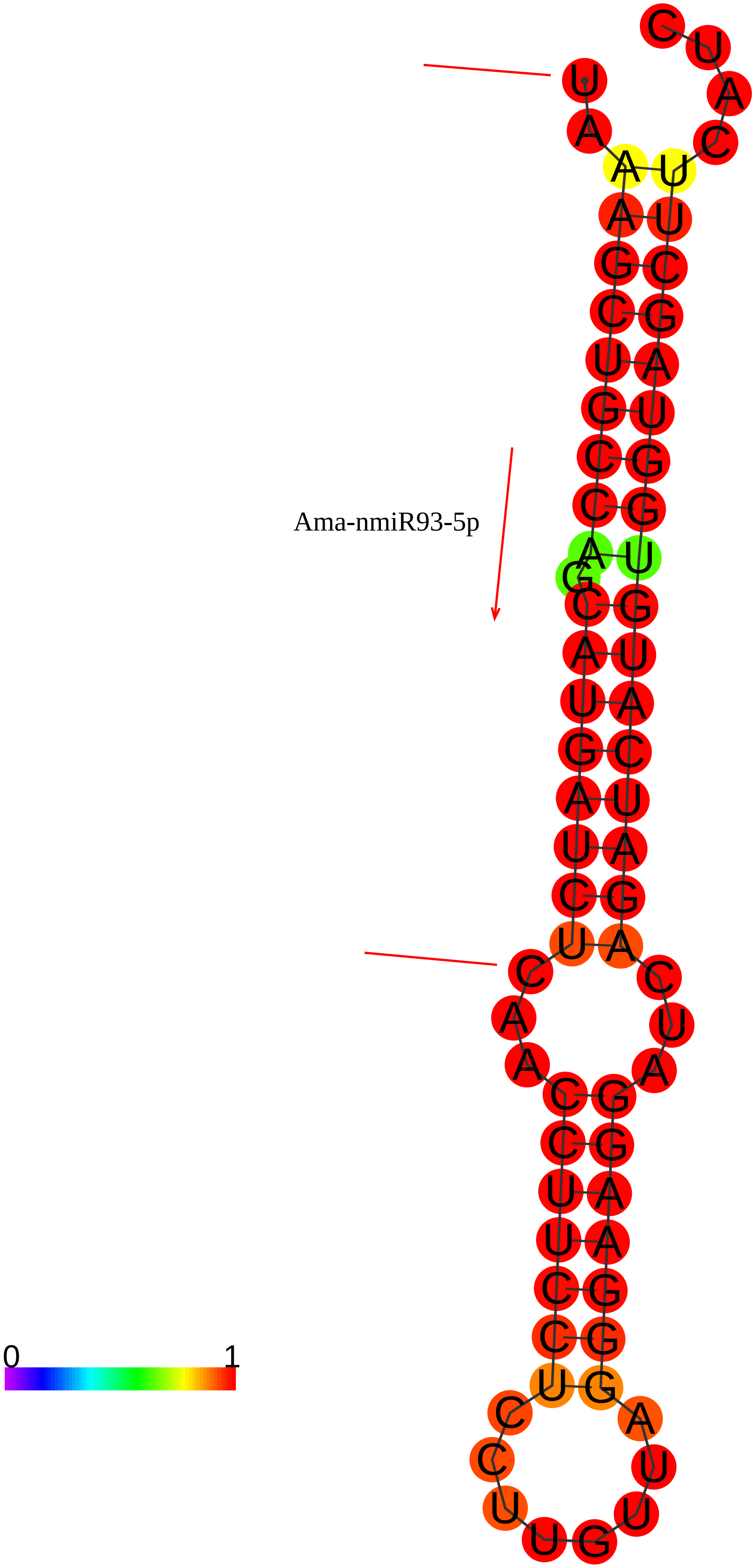
Ama-nmiR93-5p
0
1

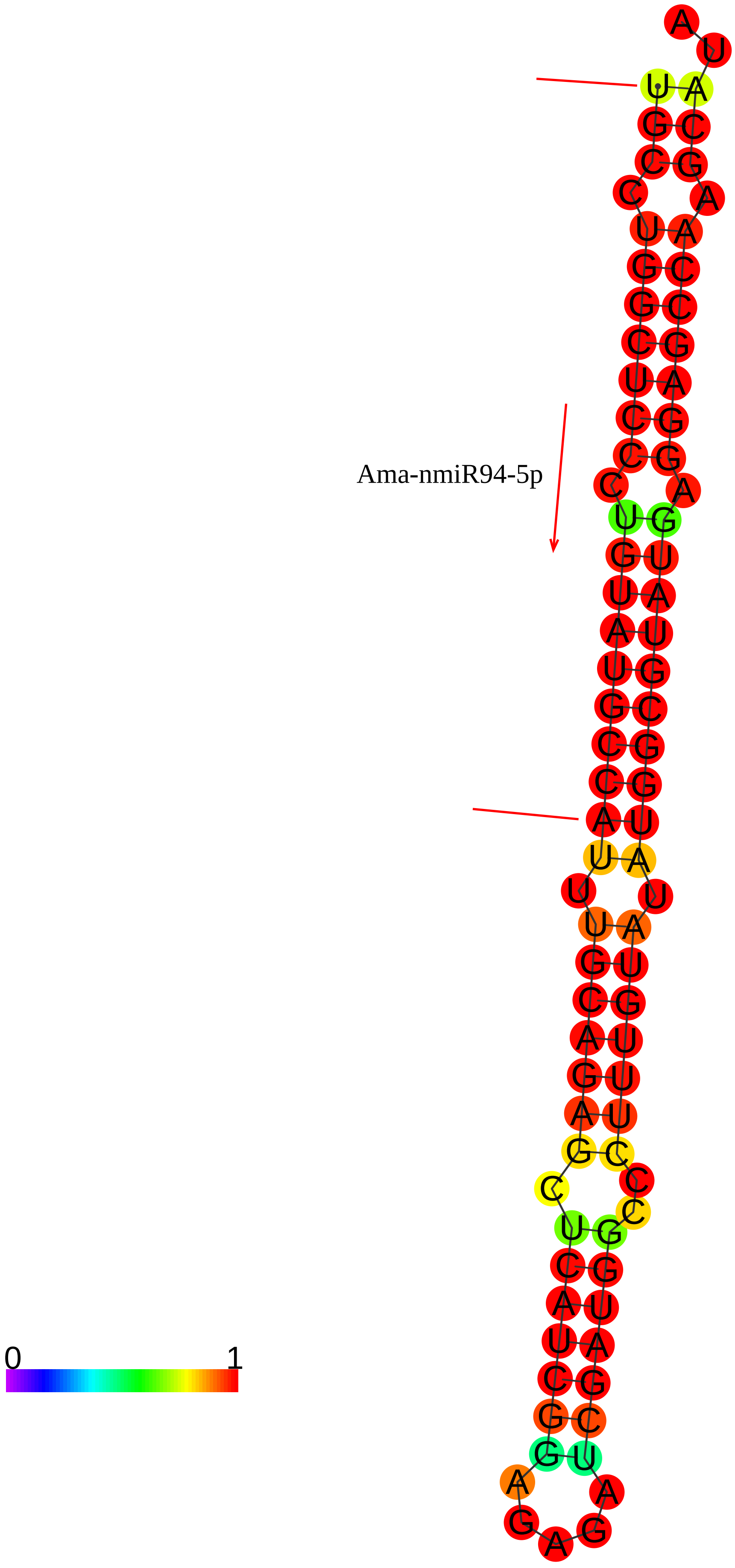
Ama-nmiR94-5p
0
1

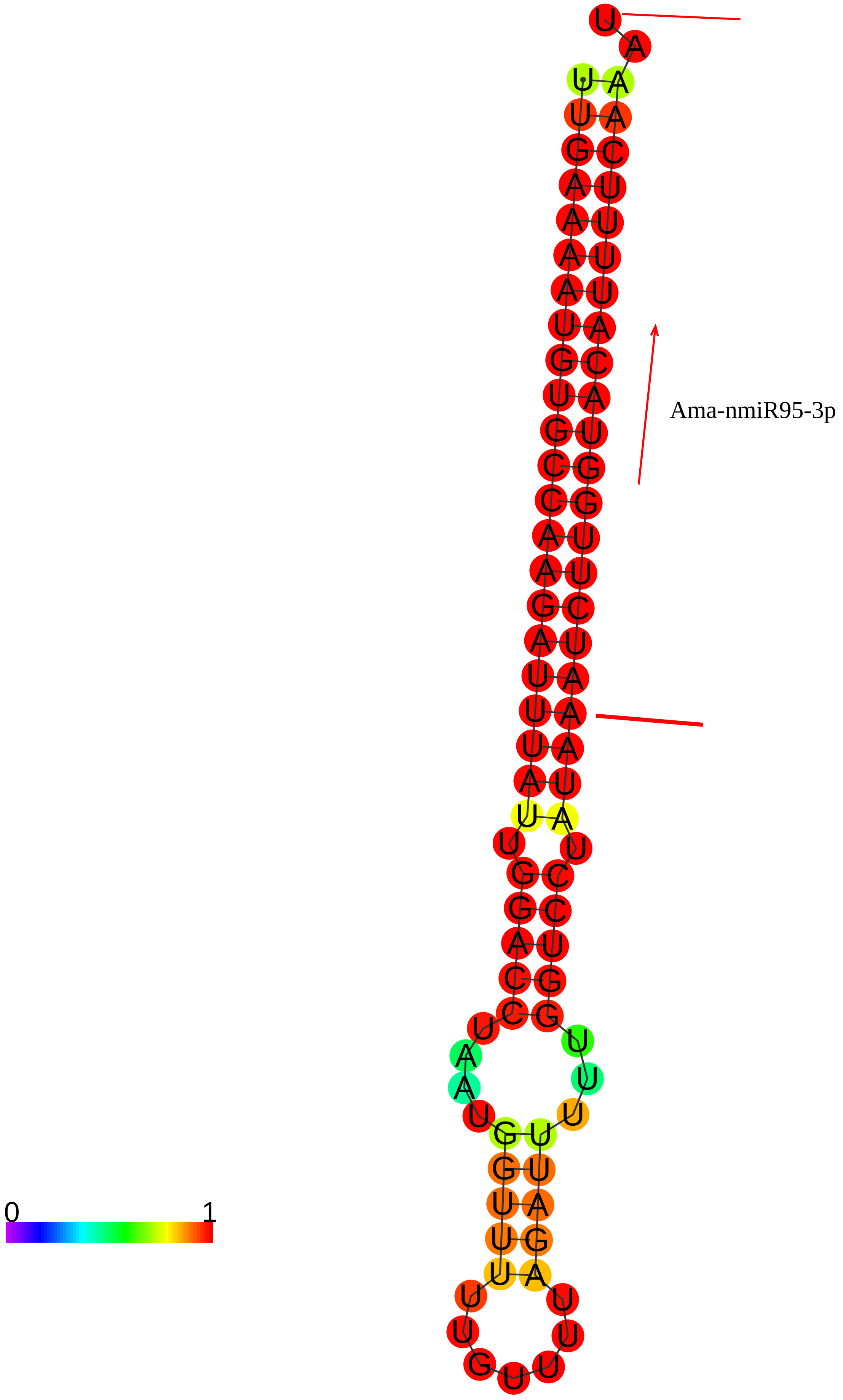
Ama-nmiR95-3p
0
1

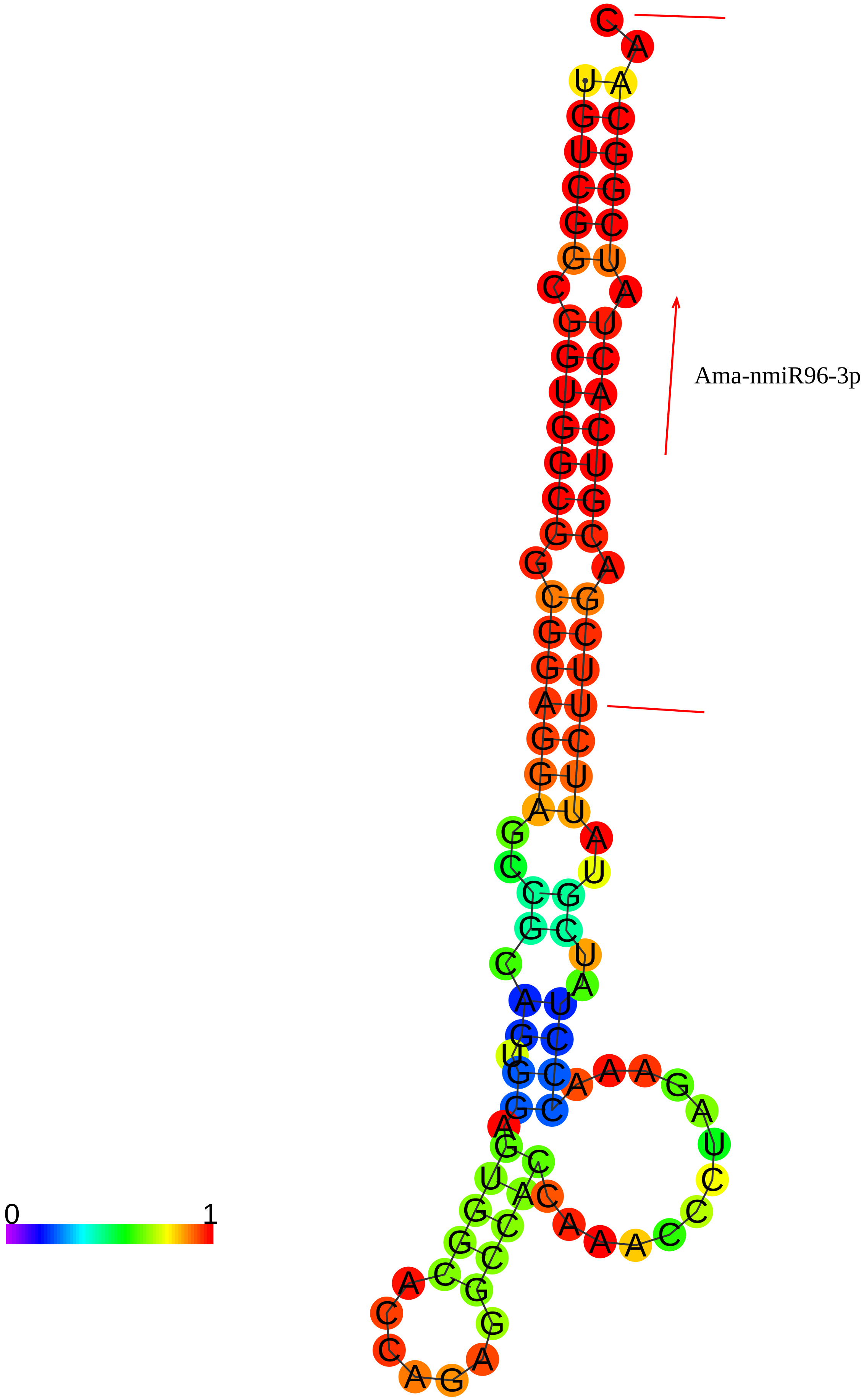
Ama-nmiR96-3p
0
1

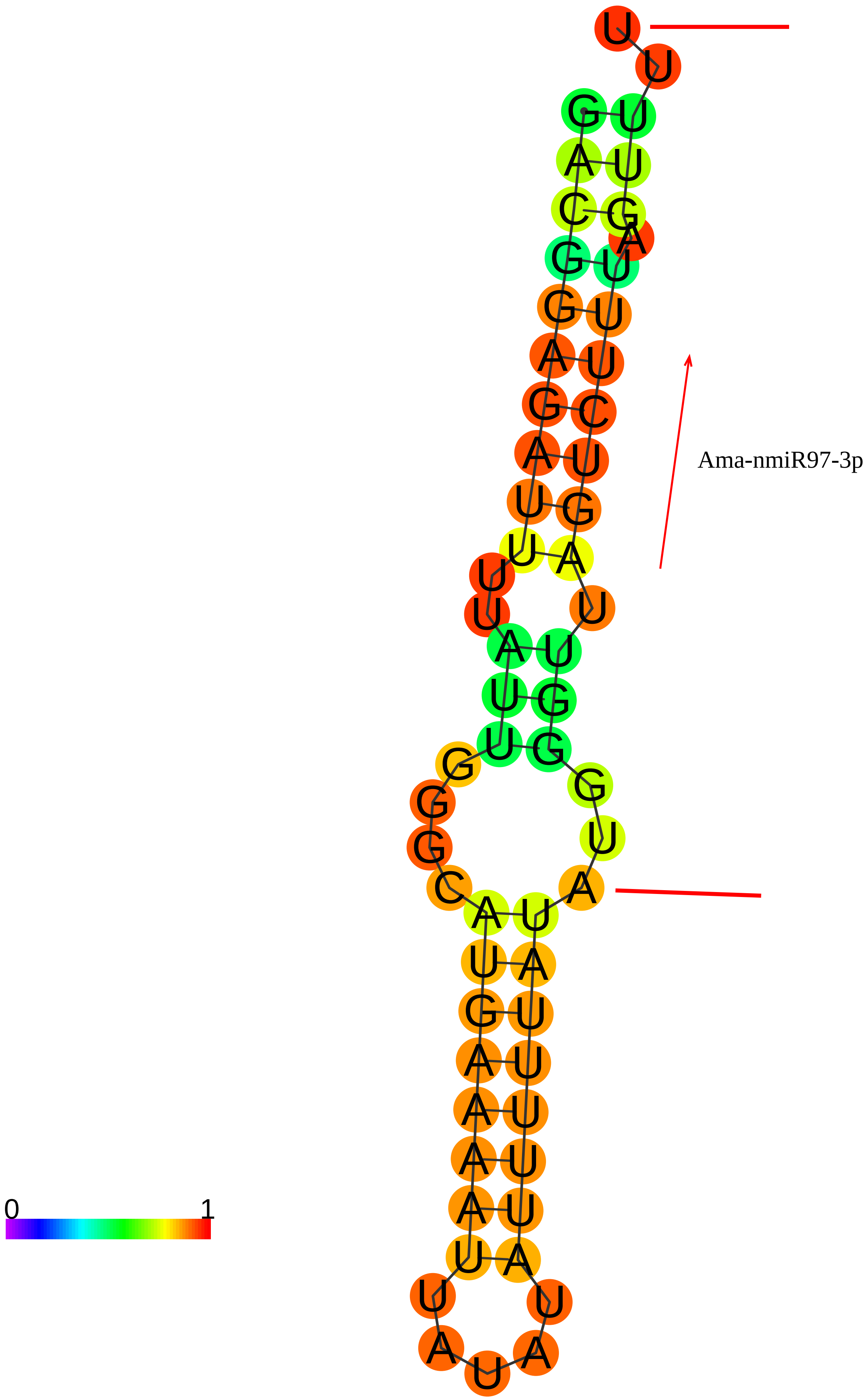
Ama-nmiR97-3p
0
1

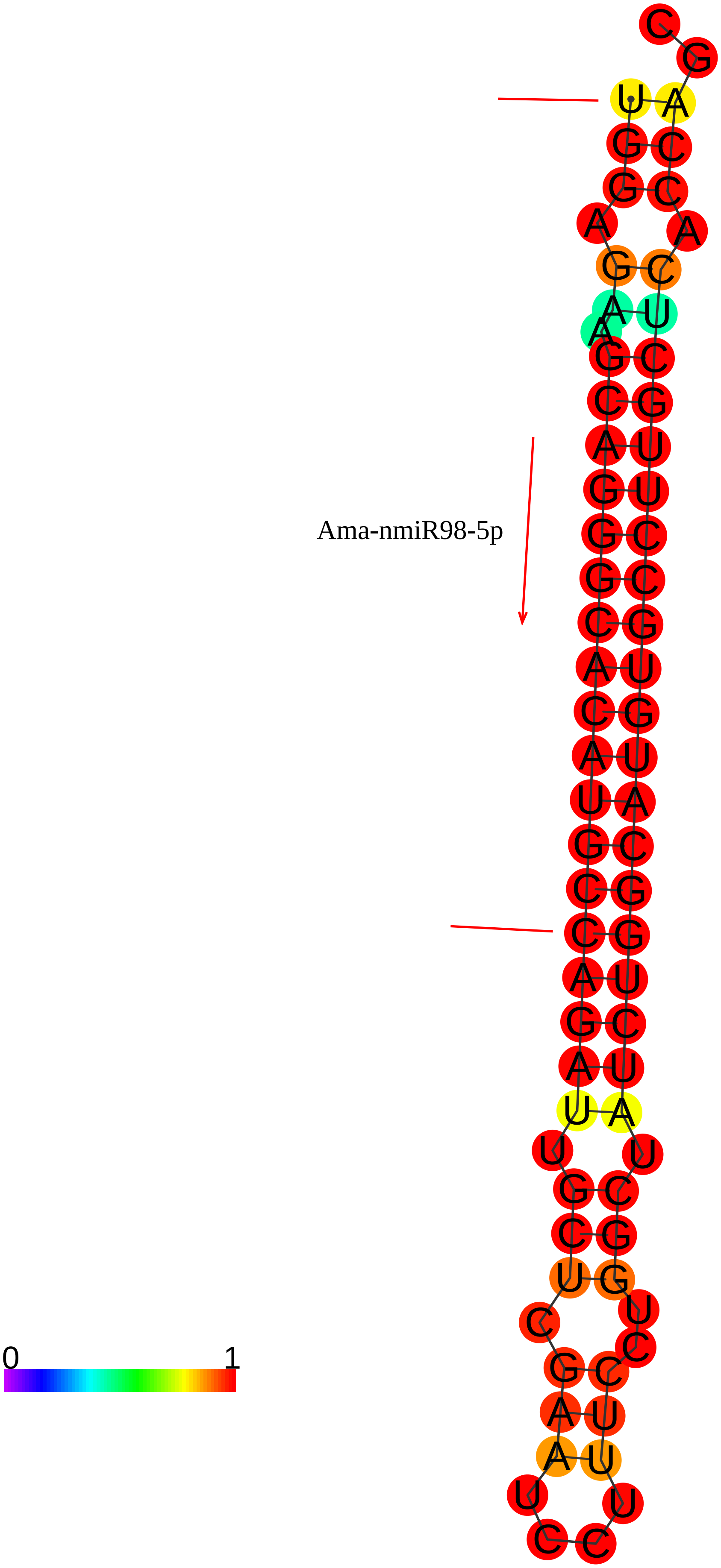
Ama-nmiR98-5p
0
1

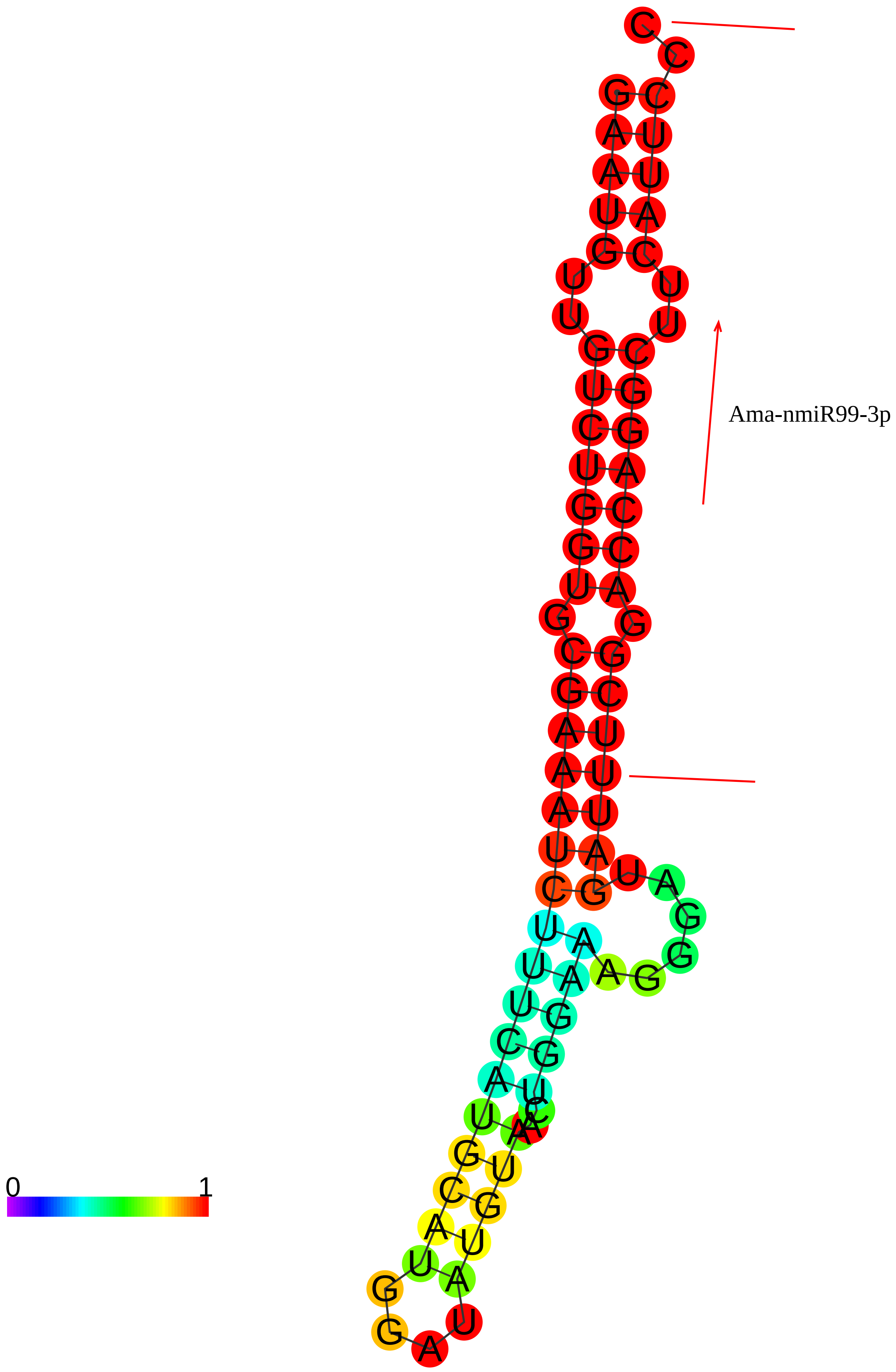
Ama-nmiR99-3p
0
1

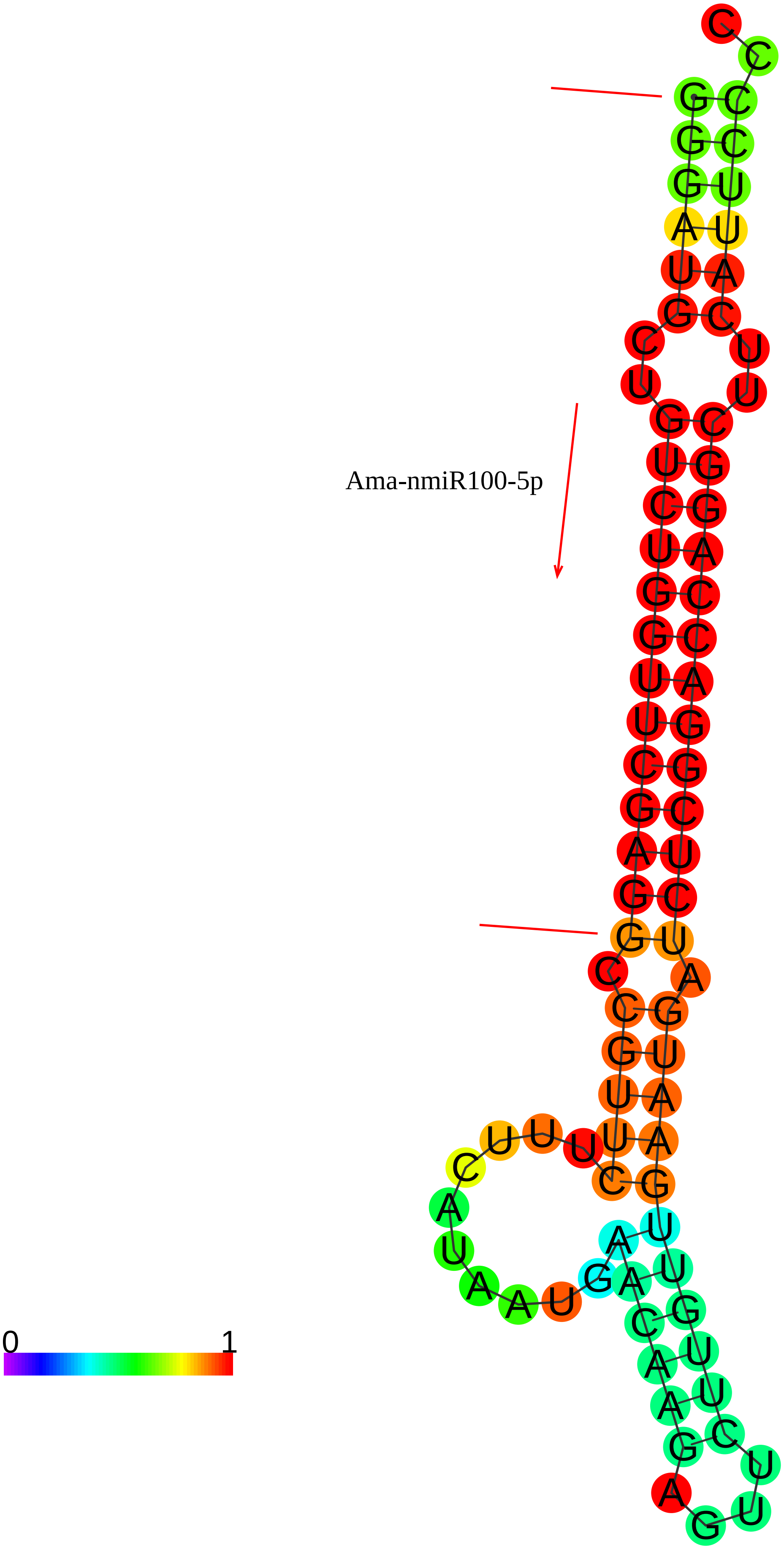
Ama-nmiR100-5p
0
1

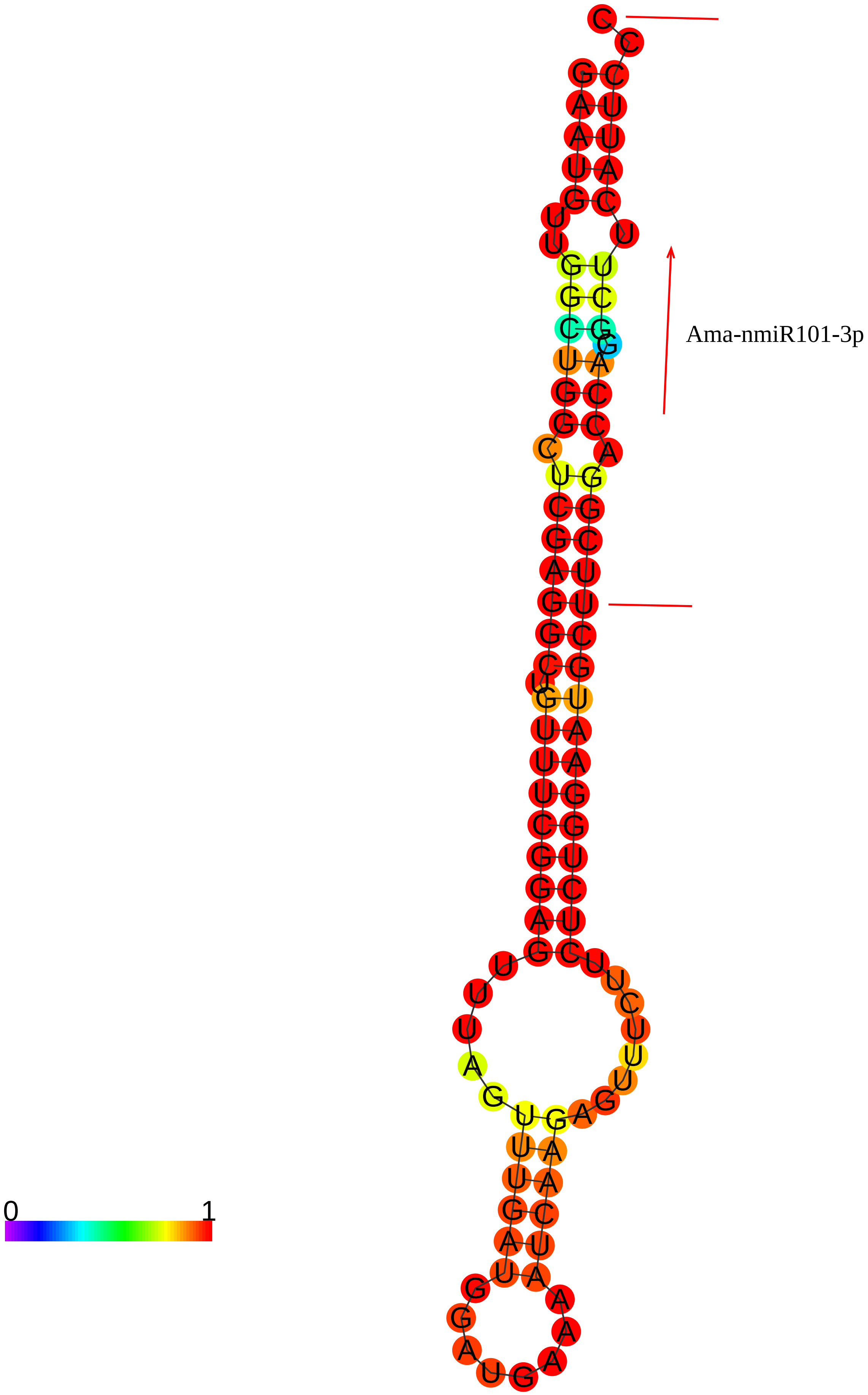
Ama-nmiR101-3p
0
1

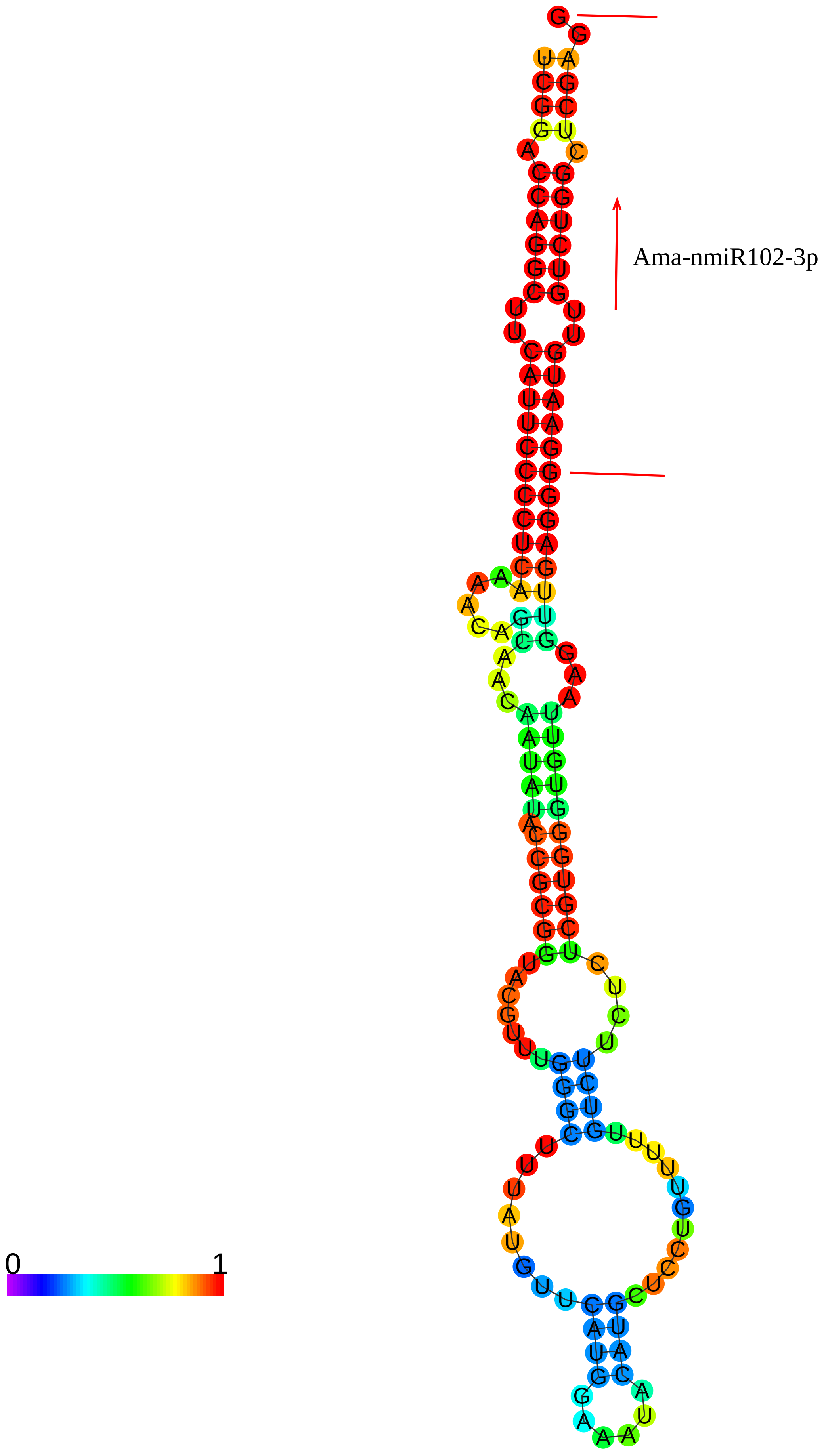
Ama-nmiR102-3p
0
1

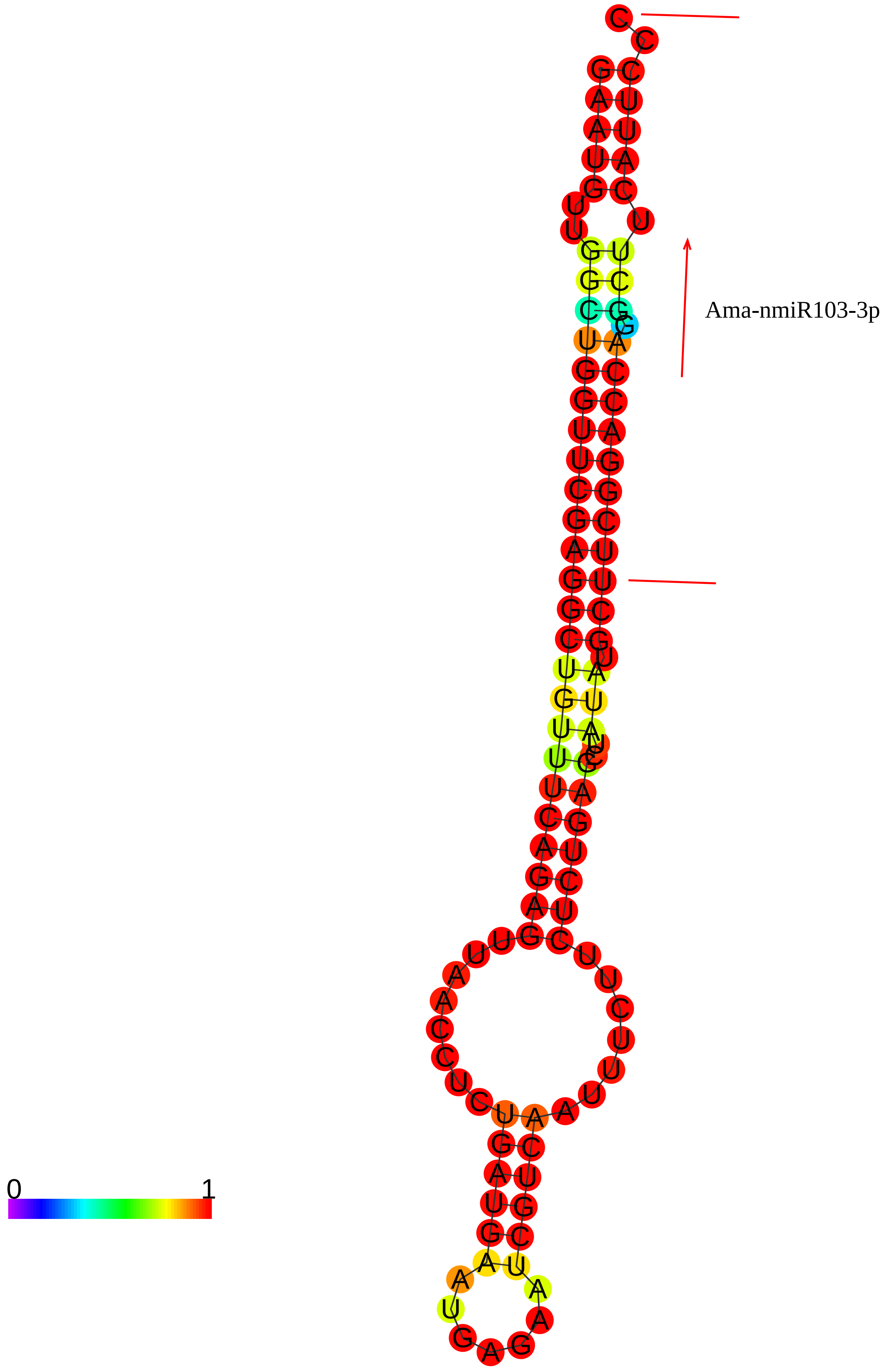
Ama-nmiR103-3p
0
1

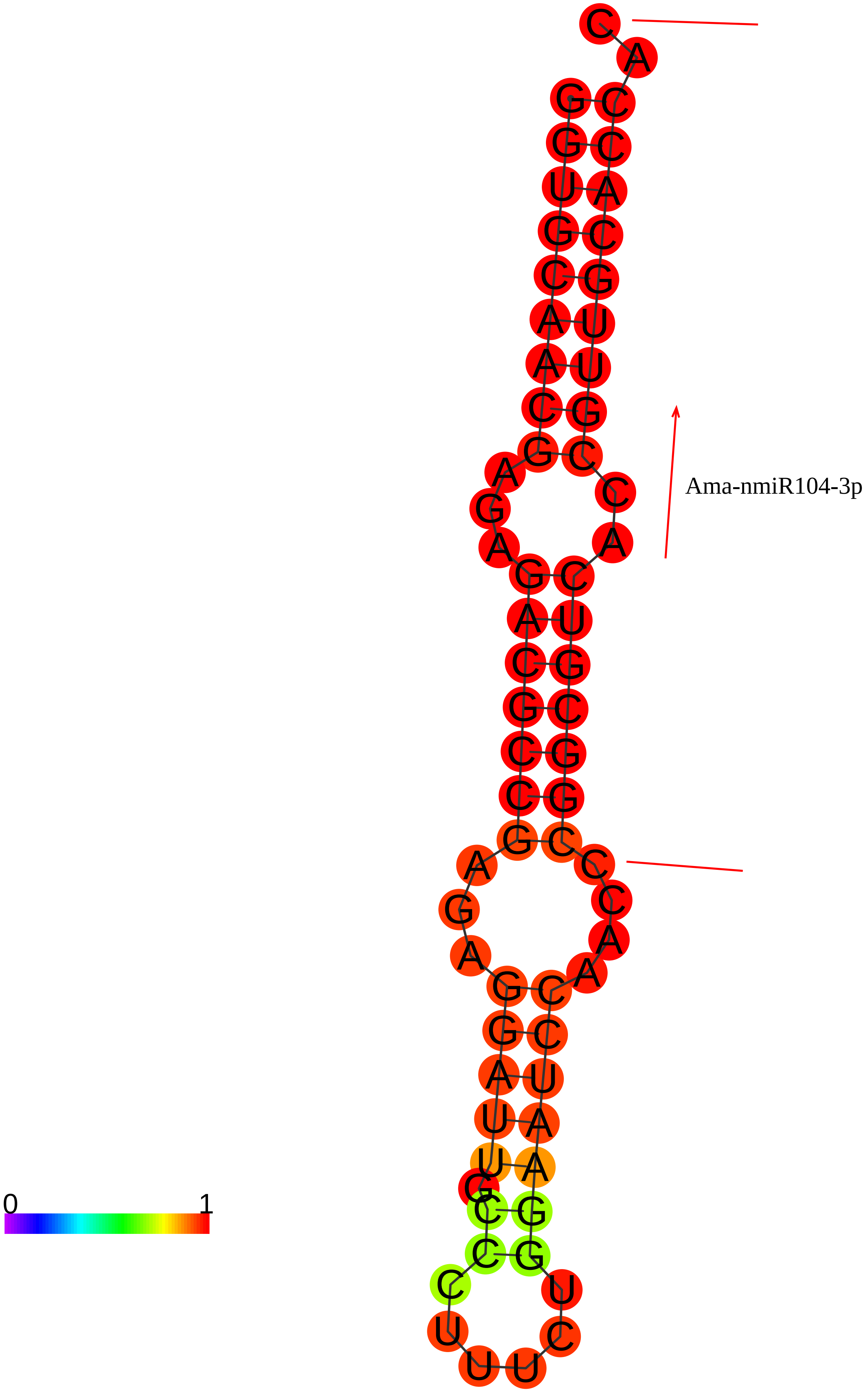
Ama-nmiR104-3p
0
1

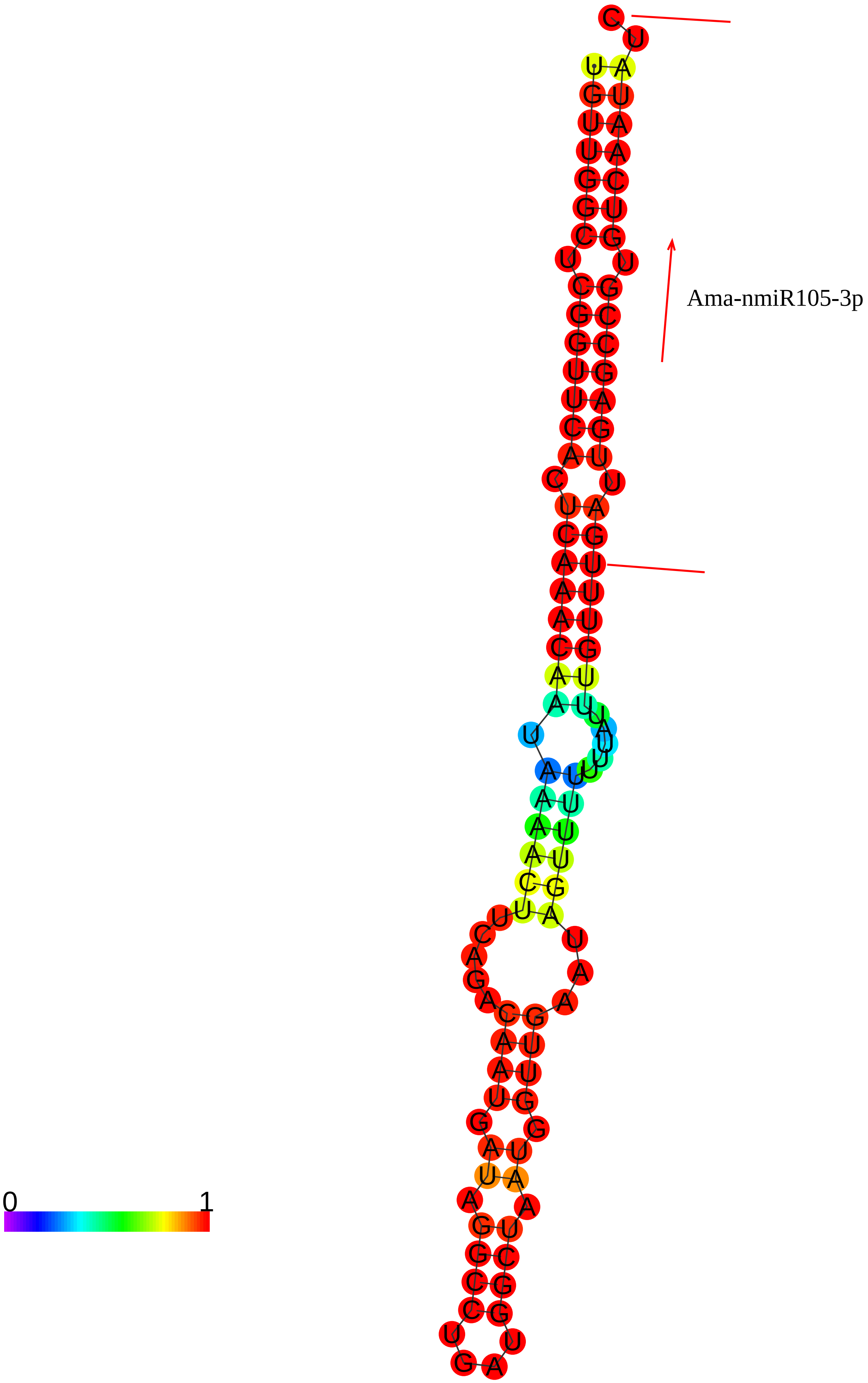
Ama-nmiR105-3p
0
1

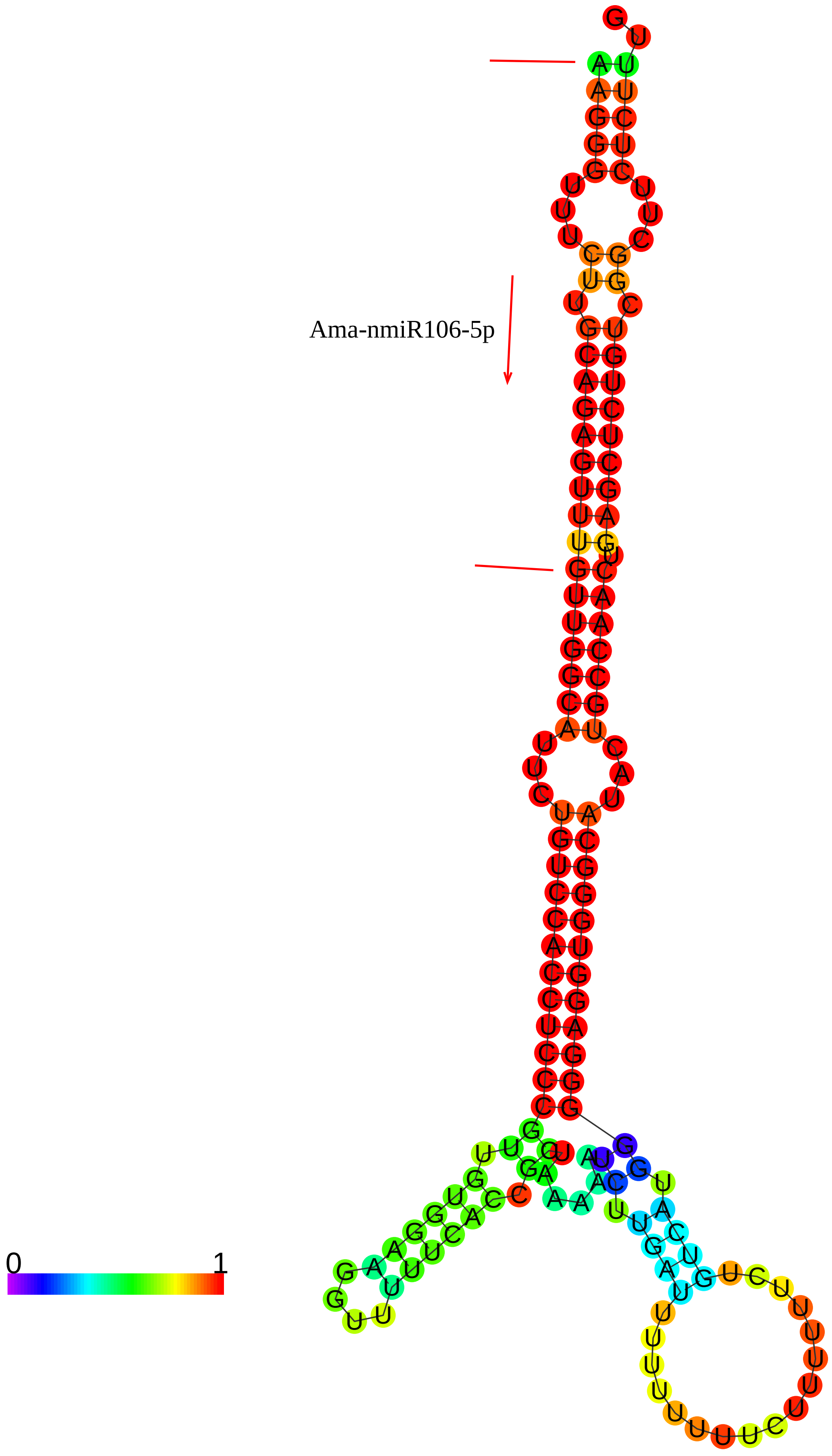
Ama-nmiR106-5p
0
1

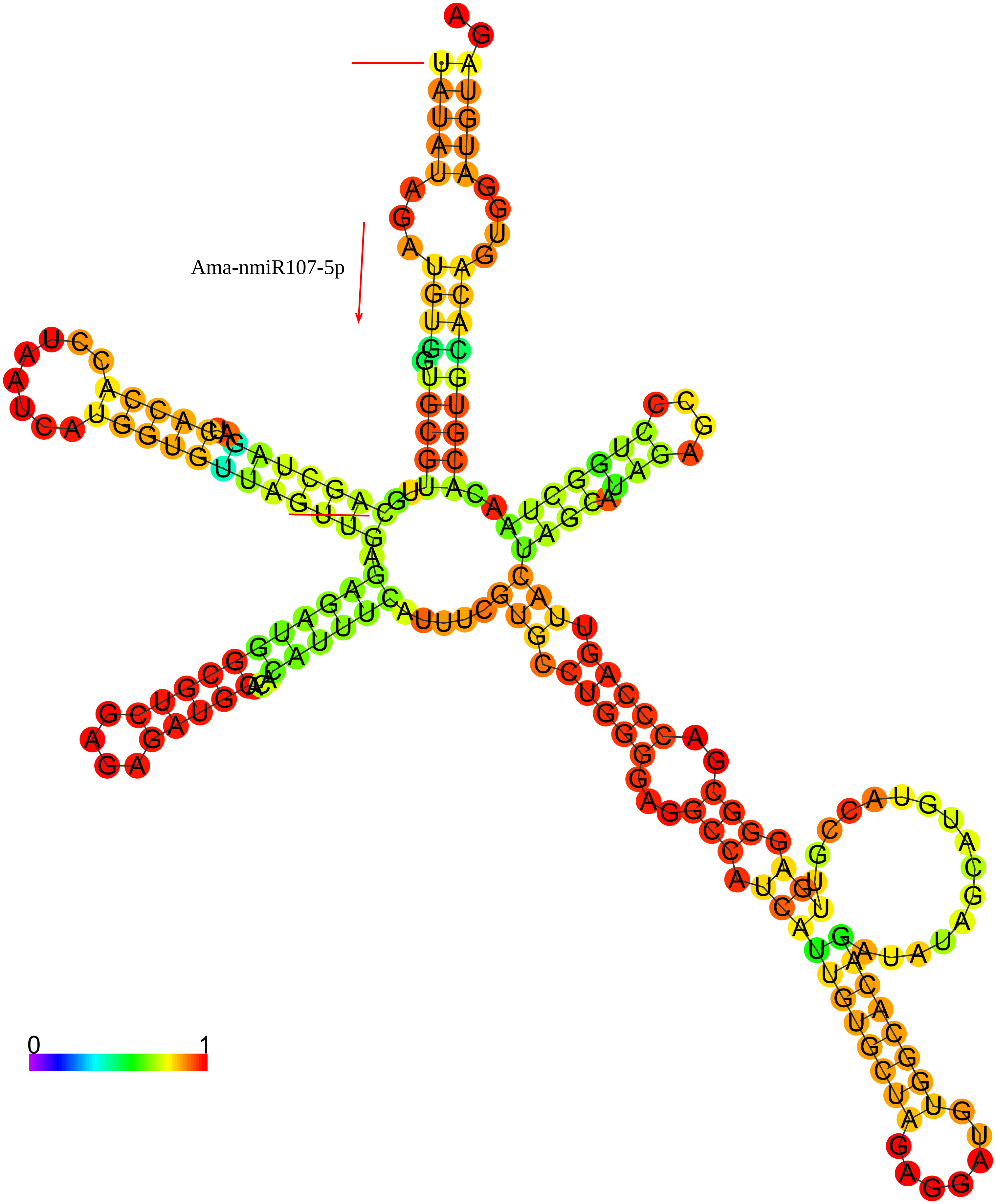
Ama-nmiR107-5p
0
1

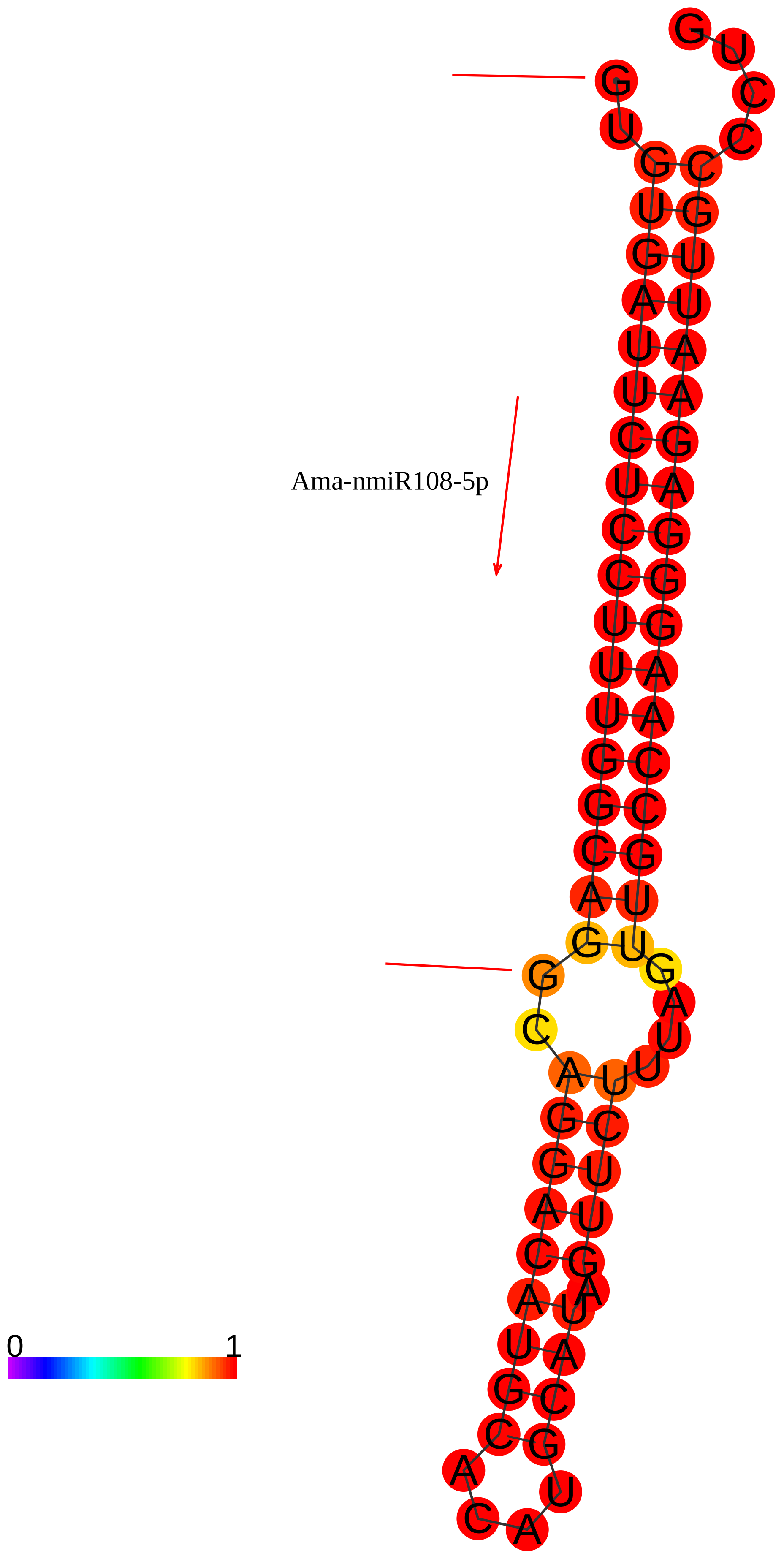
Ama-nmiR108-5p
0
1

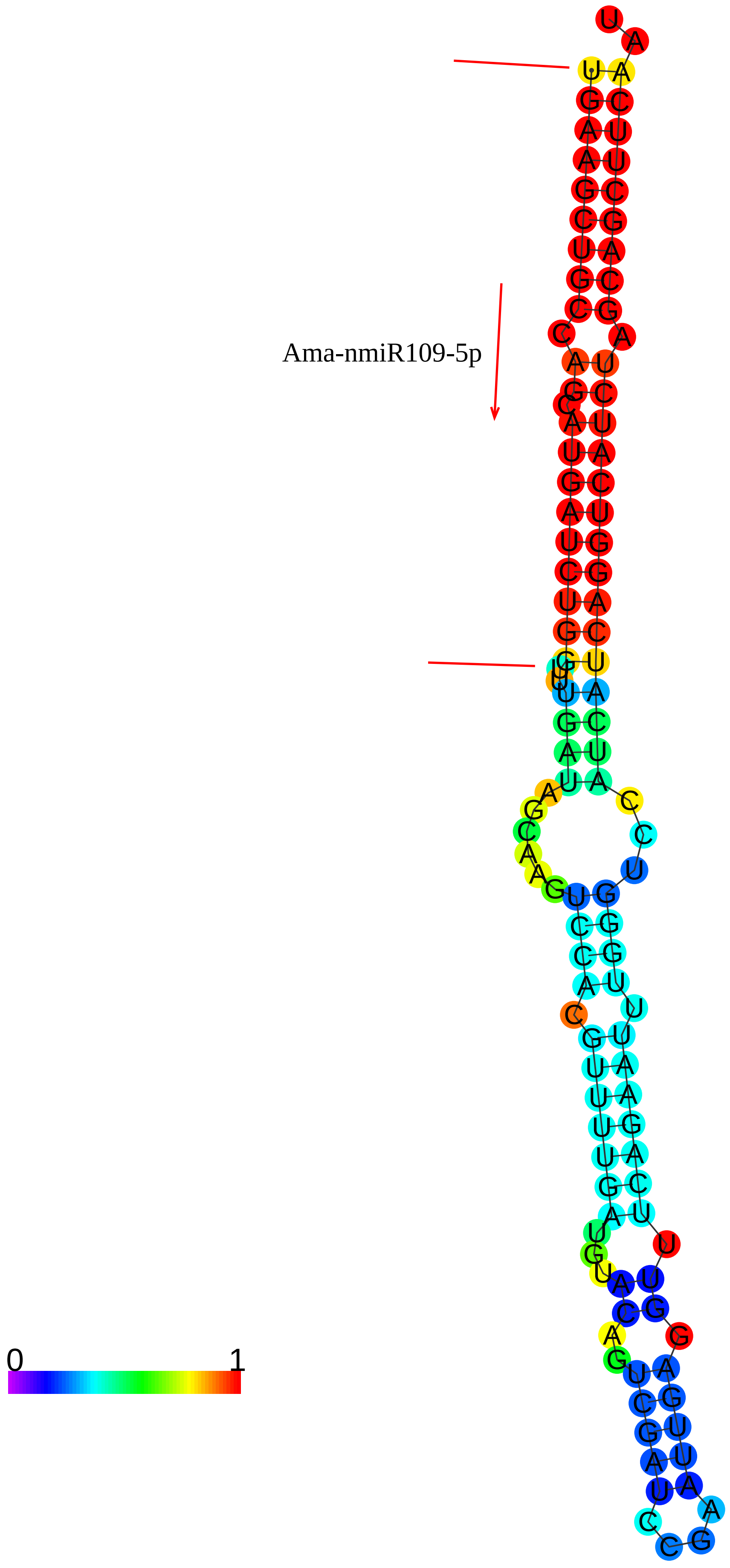
Ama-nmiR109-5p
0
1

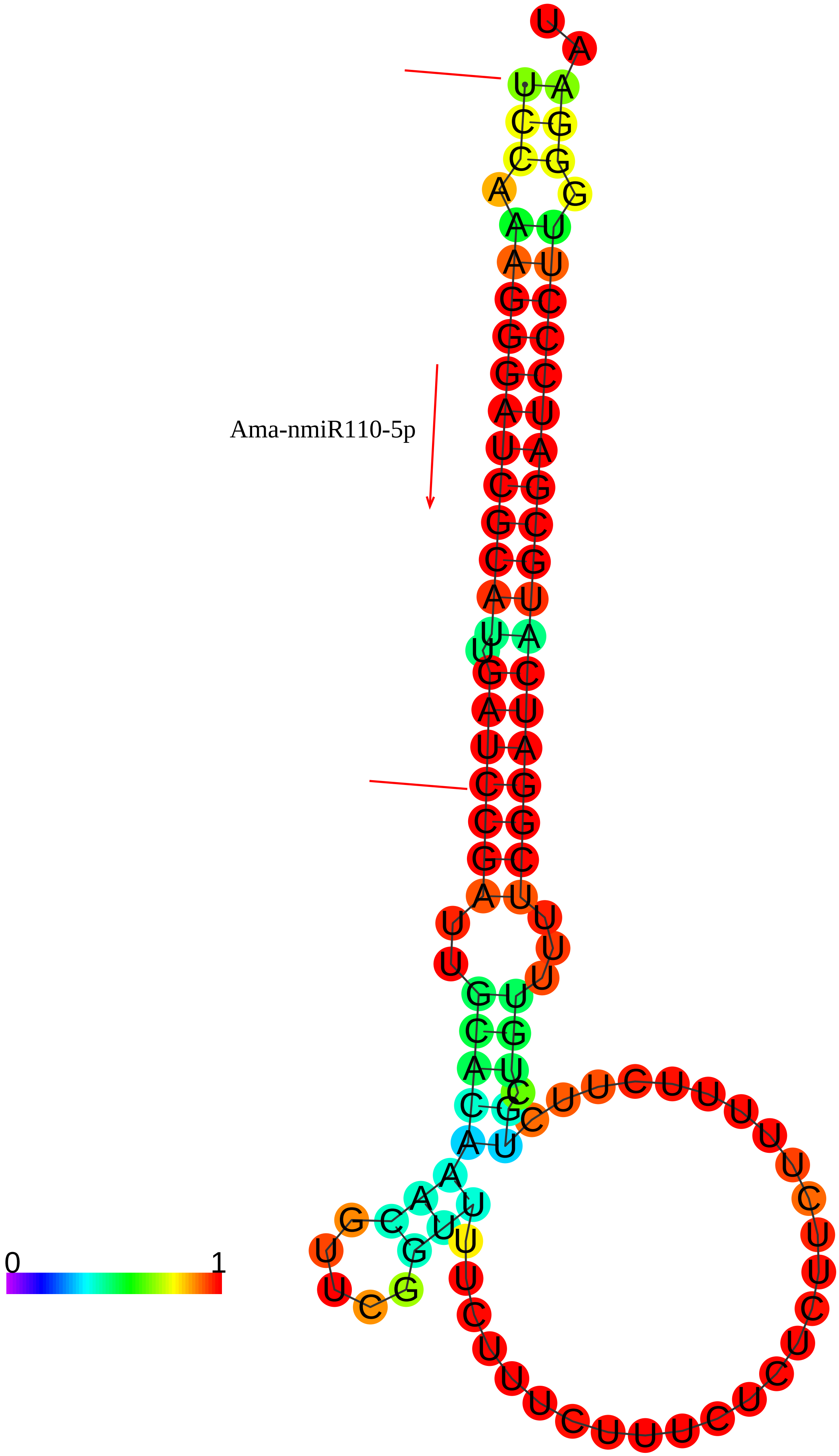
Ama-nmiR110-5p
0
1

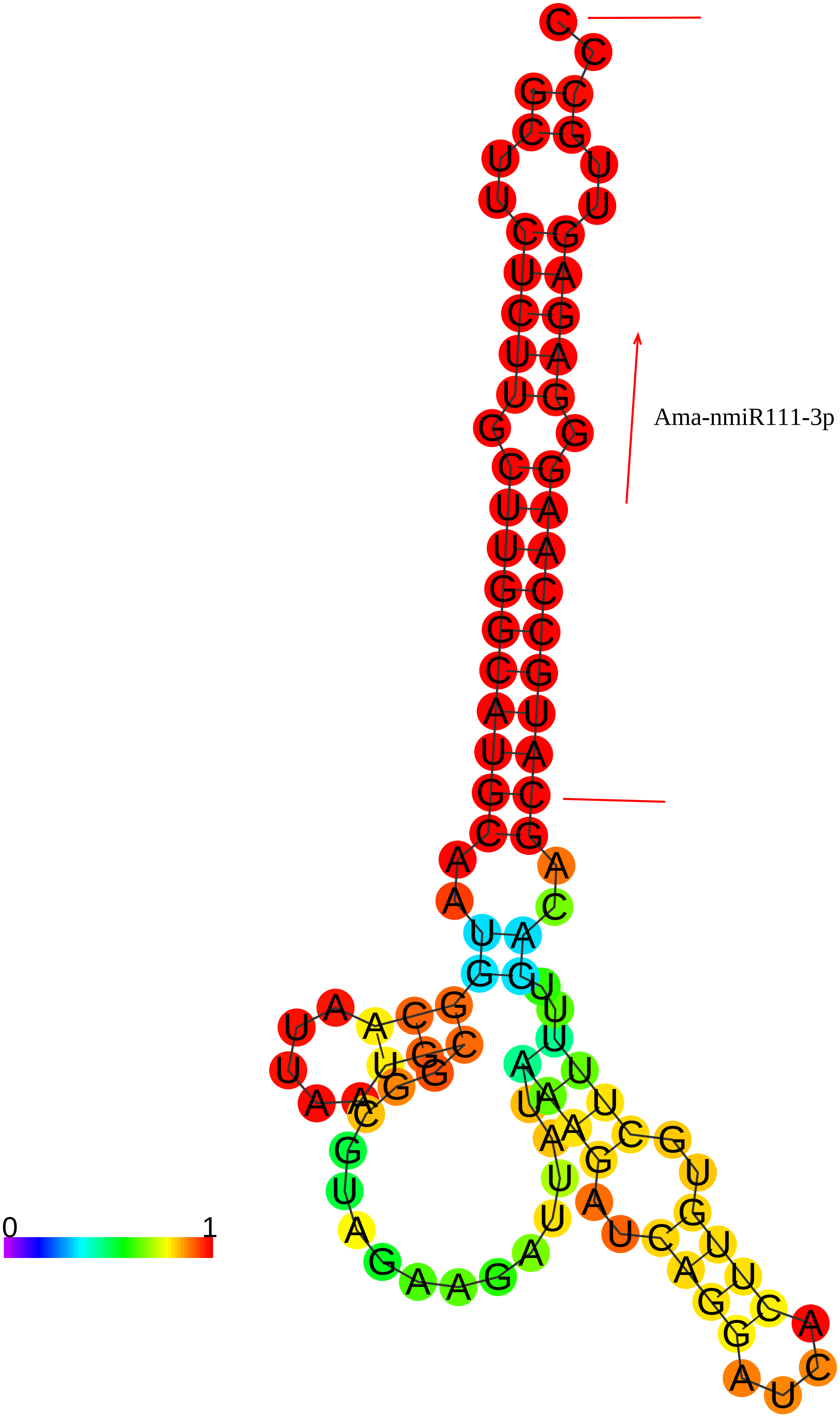
Ama-nmiR111-3p
0
1

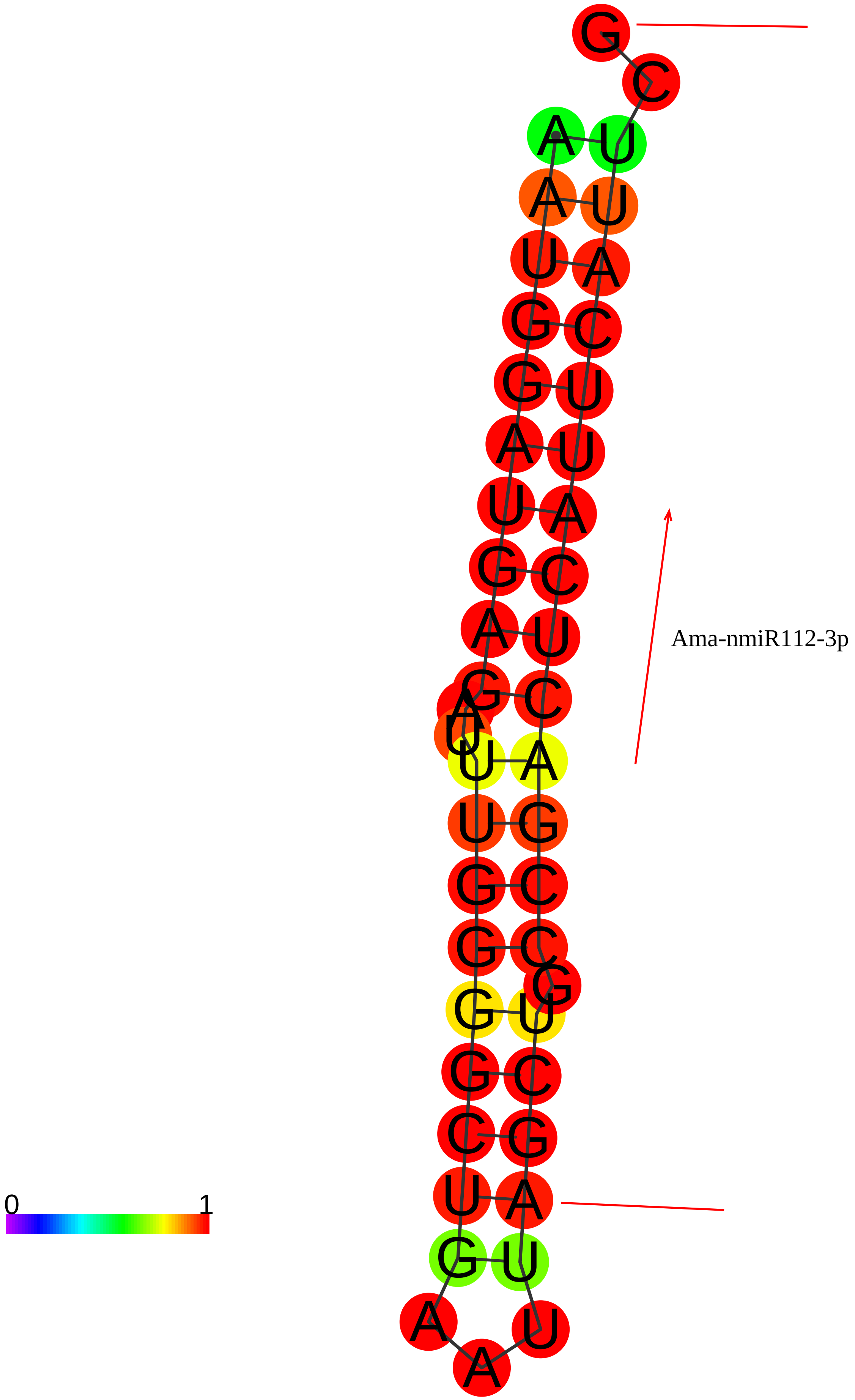
Ama-nmiR112-3p
0
1

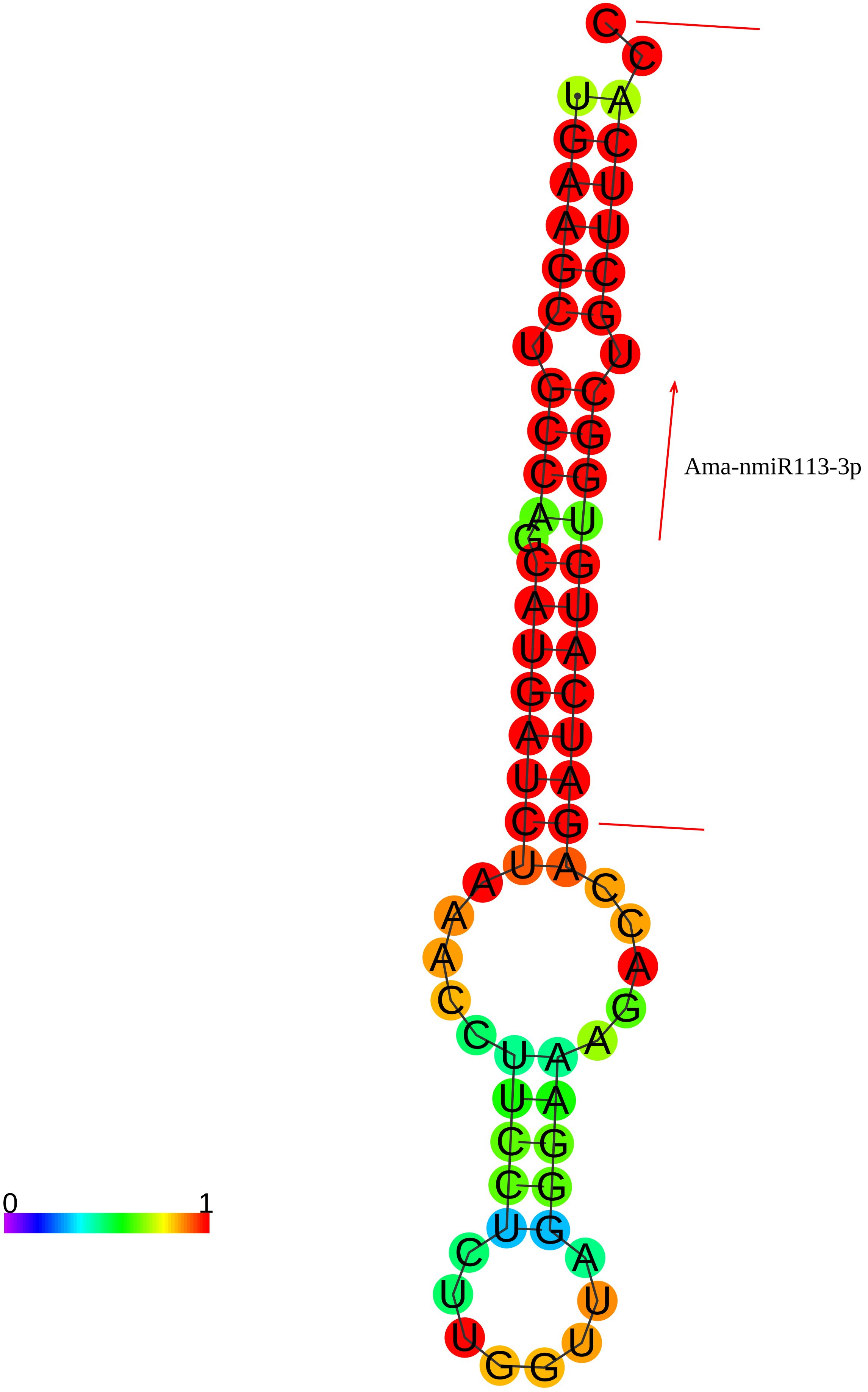
Ama-nmiR113-3p
0
1

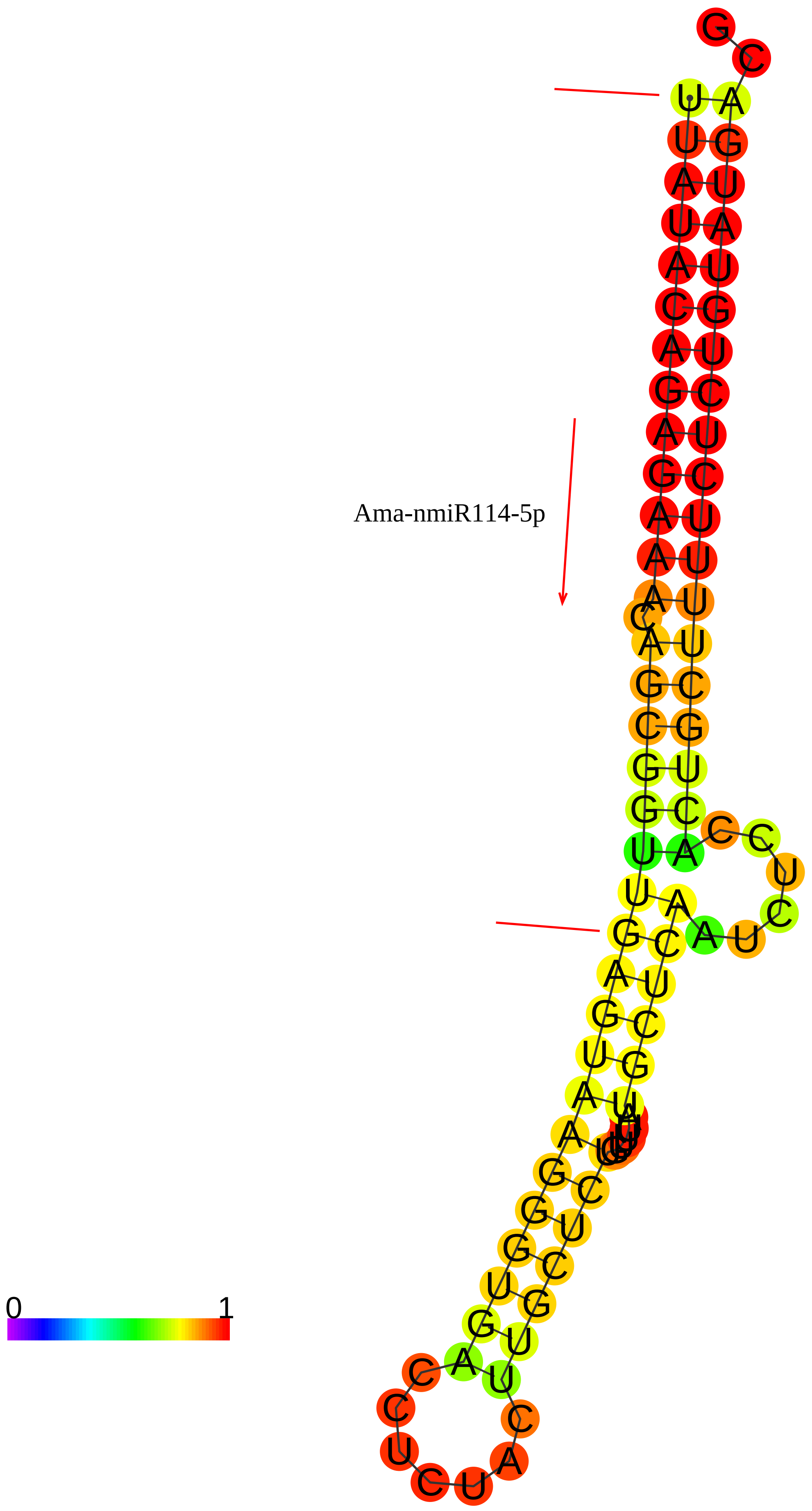
Ama-nmiR114-5p
0
1

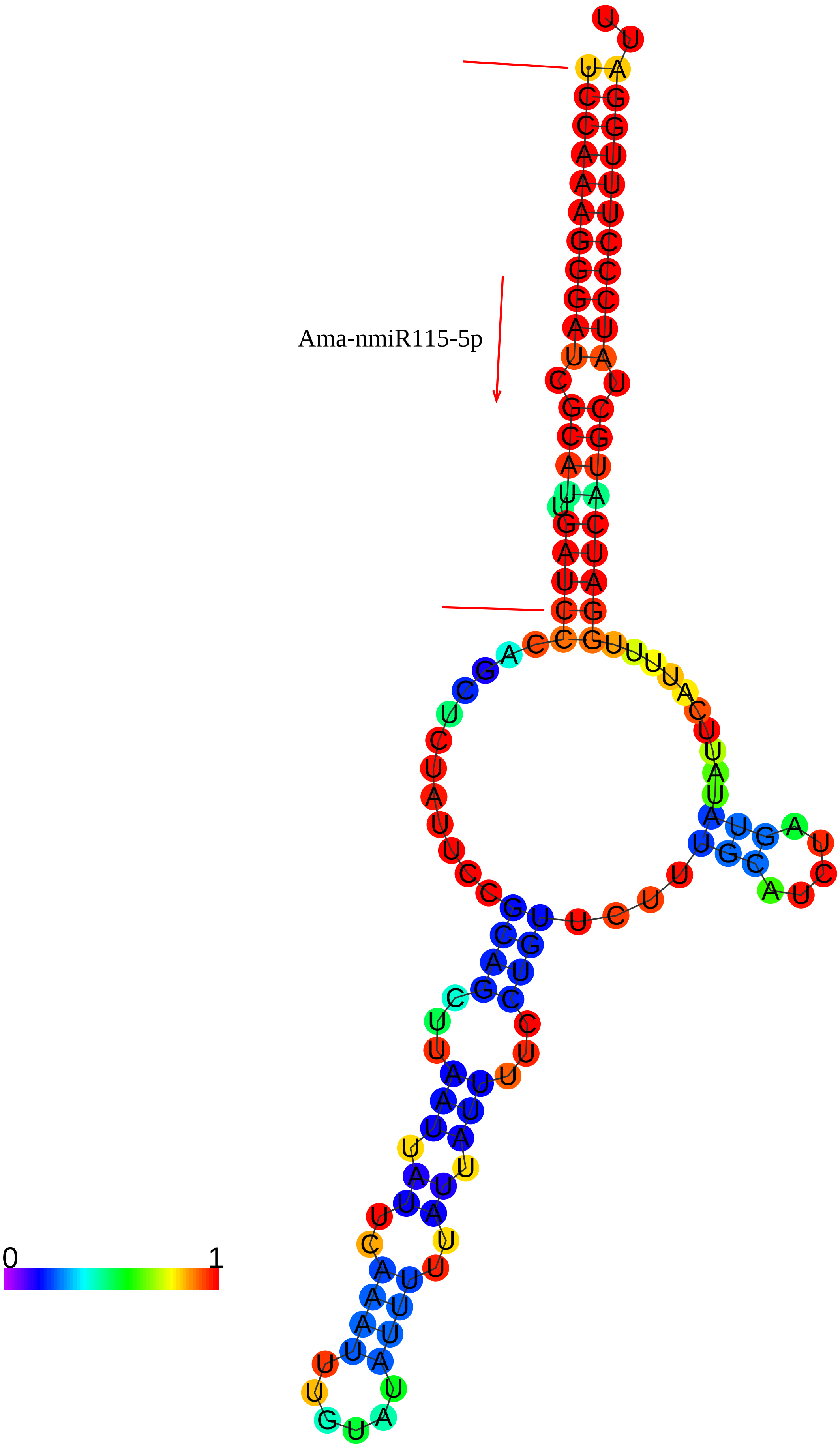
Ama-nmiR115-5p
0
1

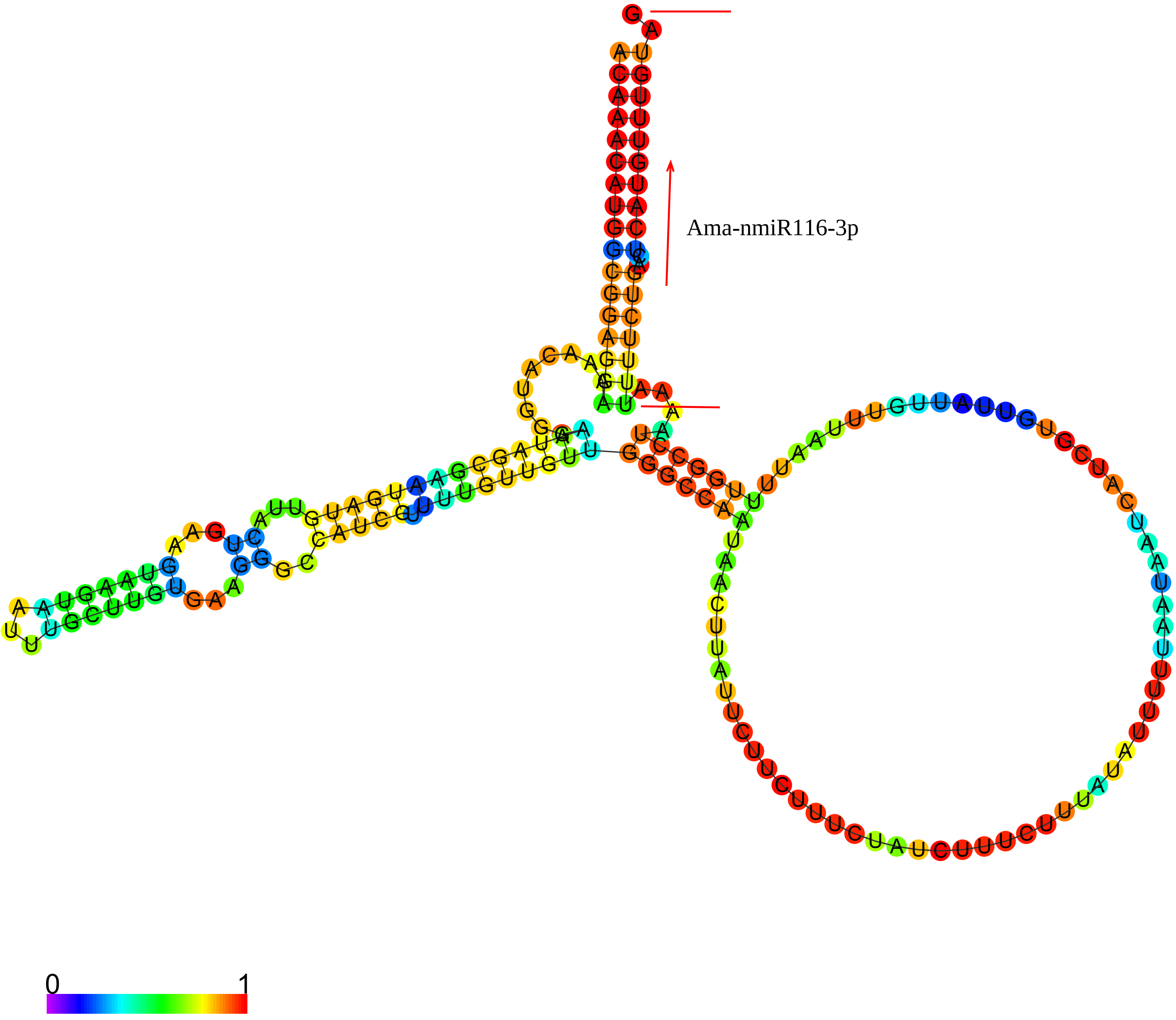
Ama-nmiR116-3p
0
1

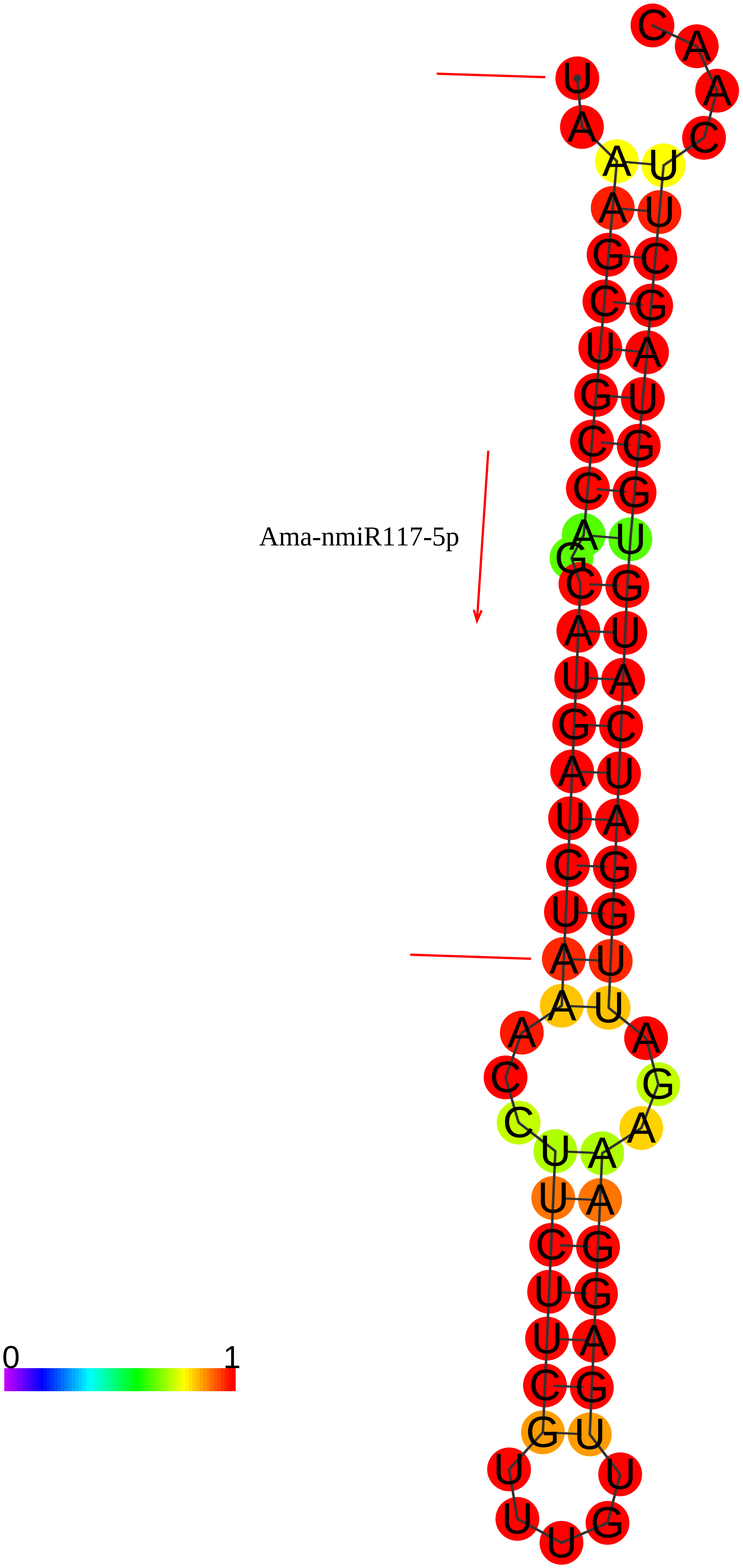
Ama-nmiR117-5p
0
1

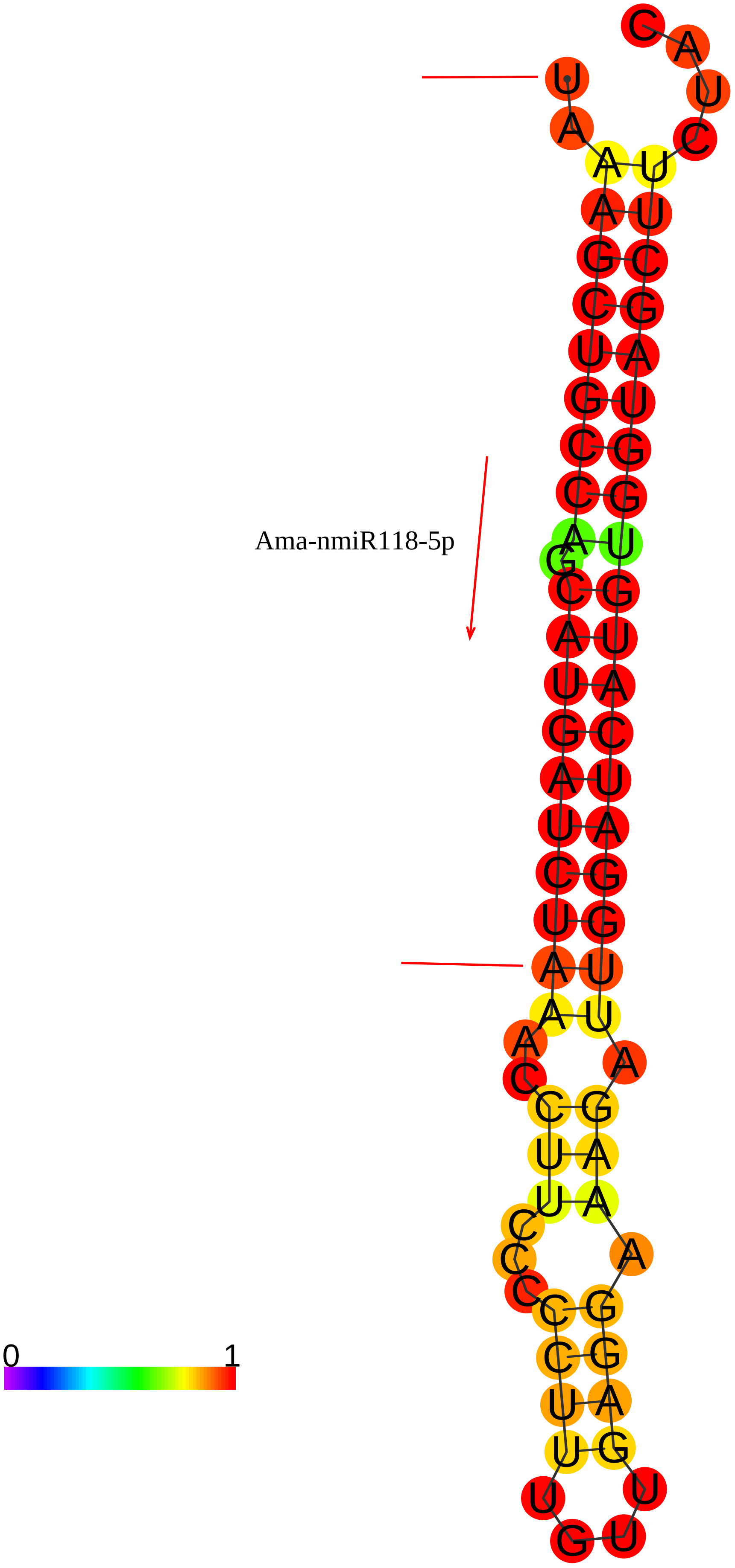

Ama-nmiR118-5p
0
1

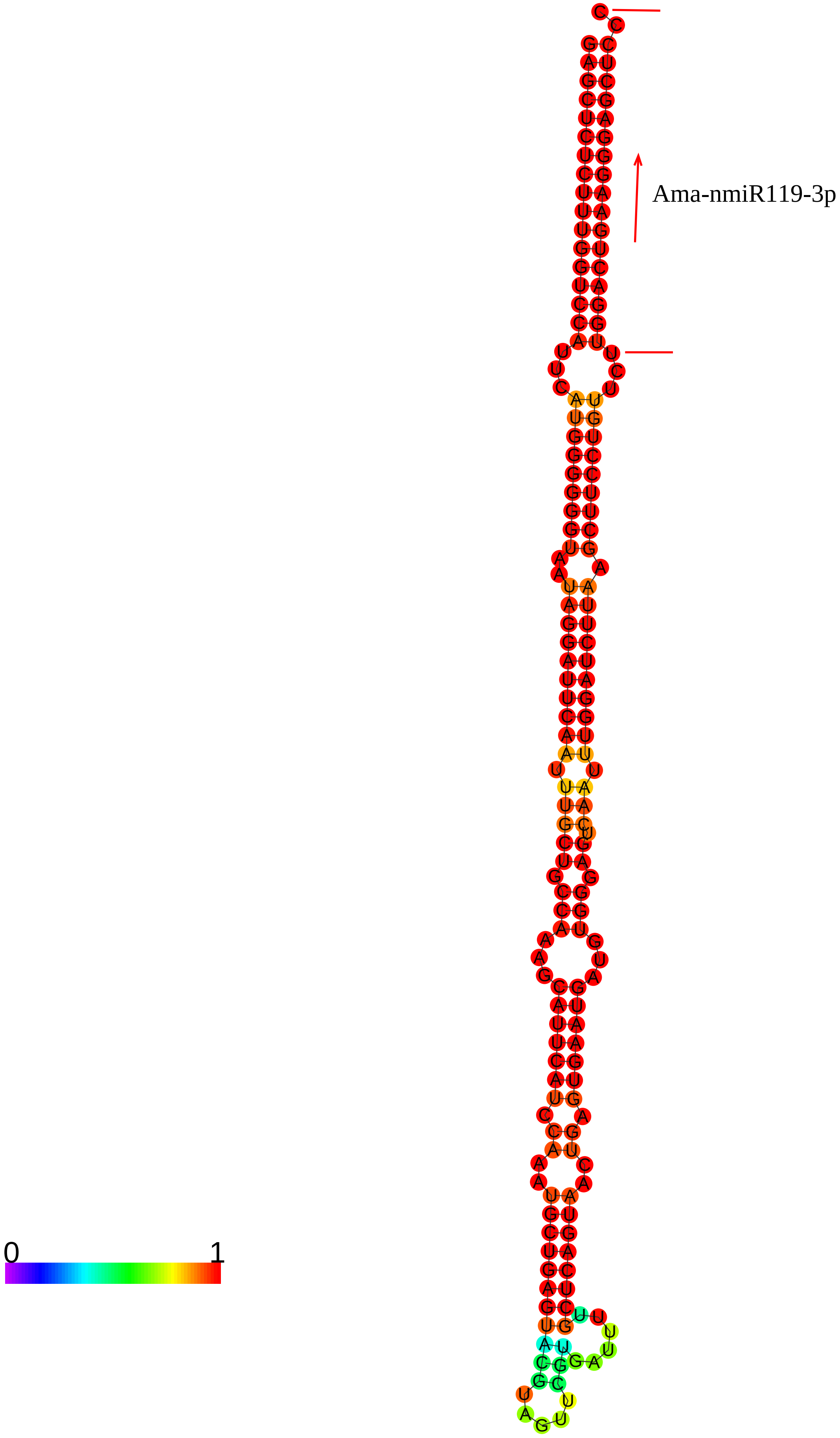
Ama-nmiR119-3p
0
1

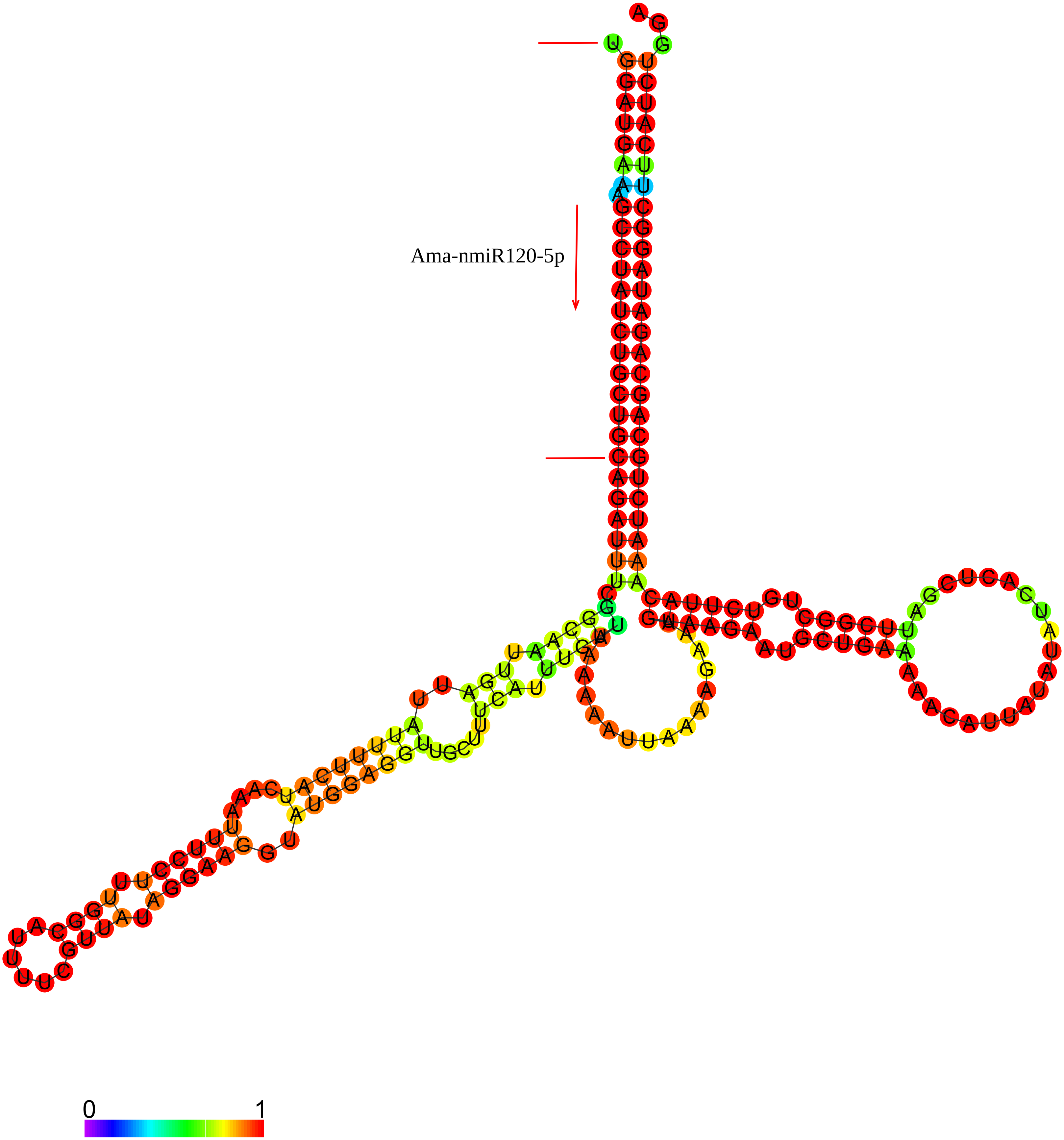
Ama-nmiR120-5p
0
1

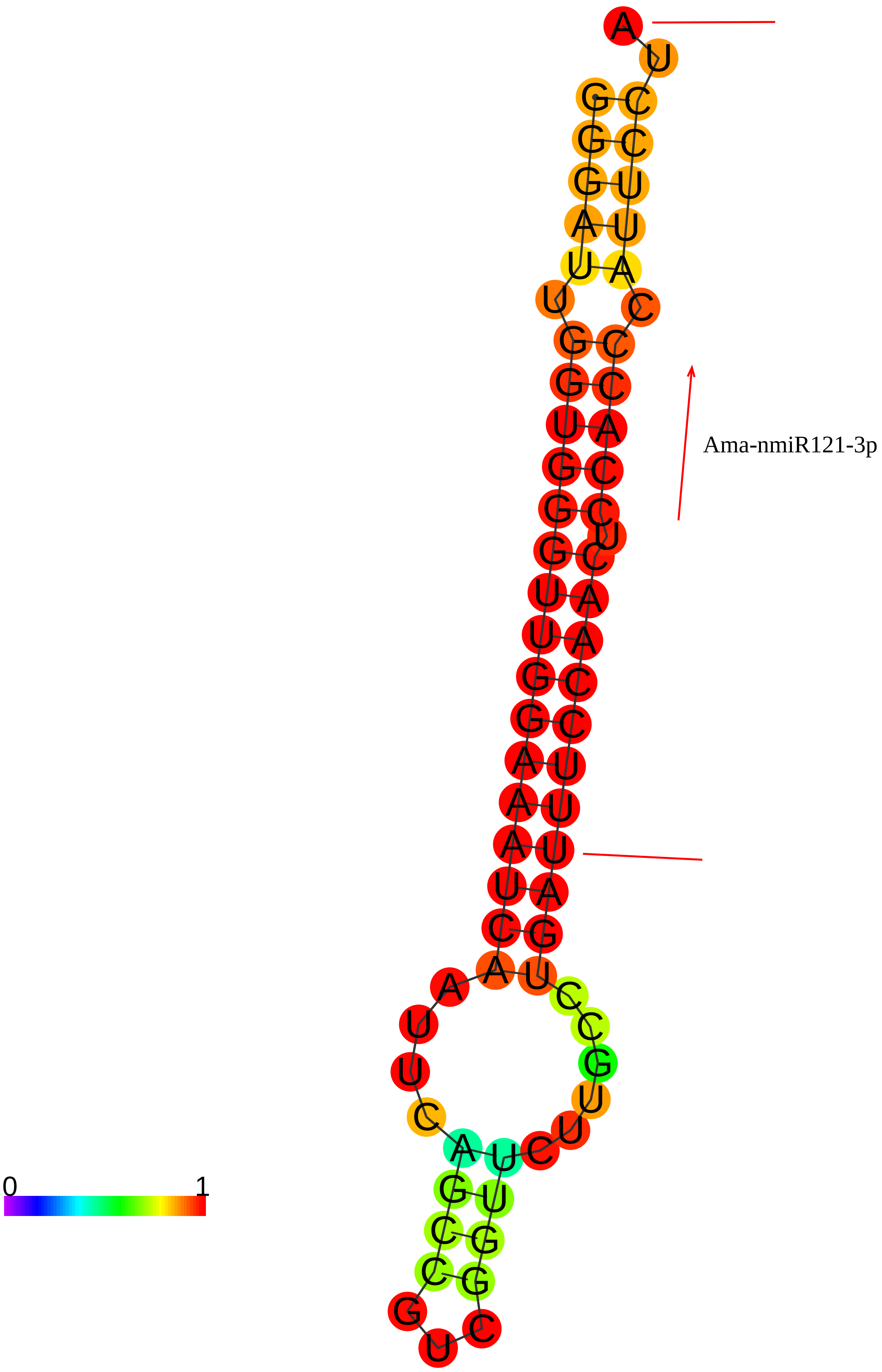
Ama-nmiR121-3p
0
1

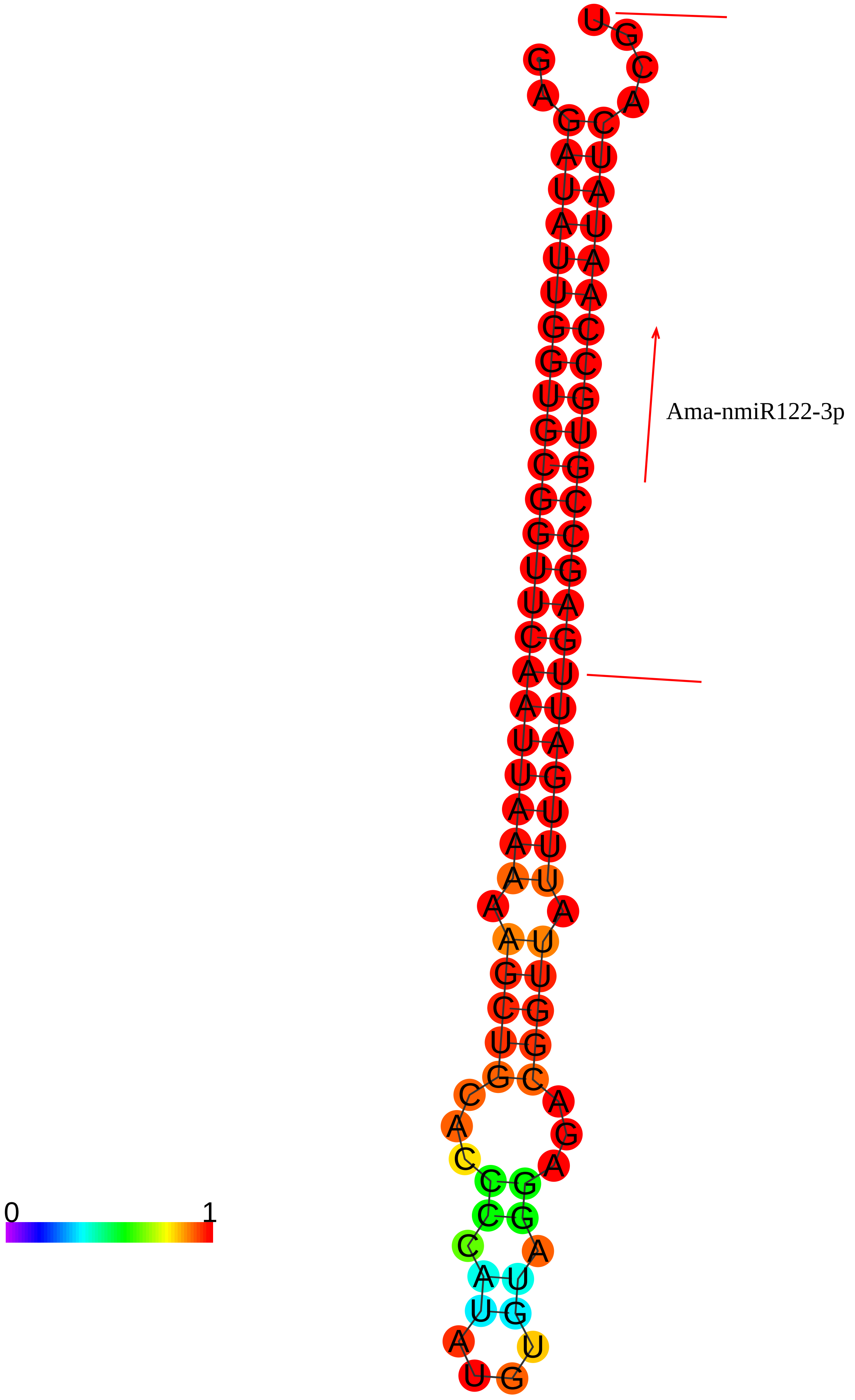

Ama-nmiR122-3p
0
1

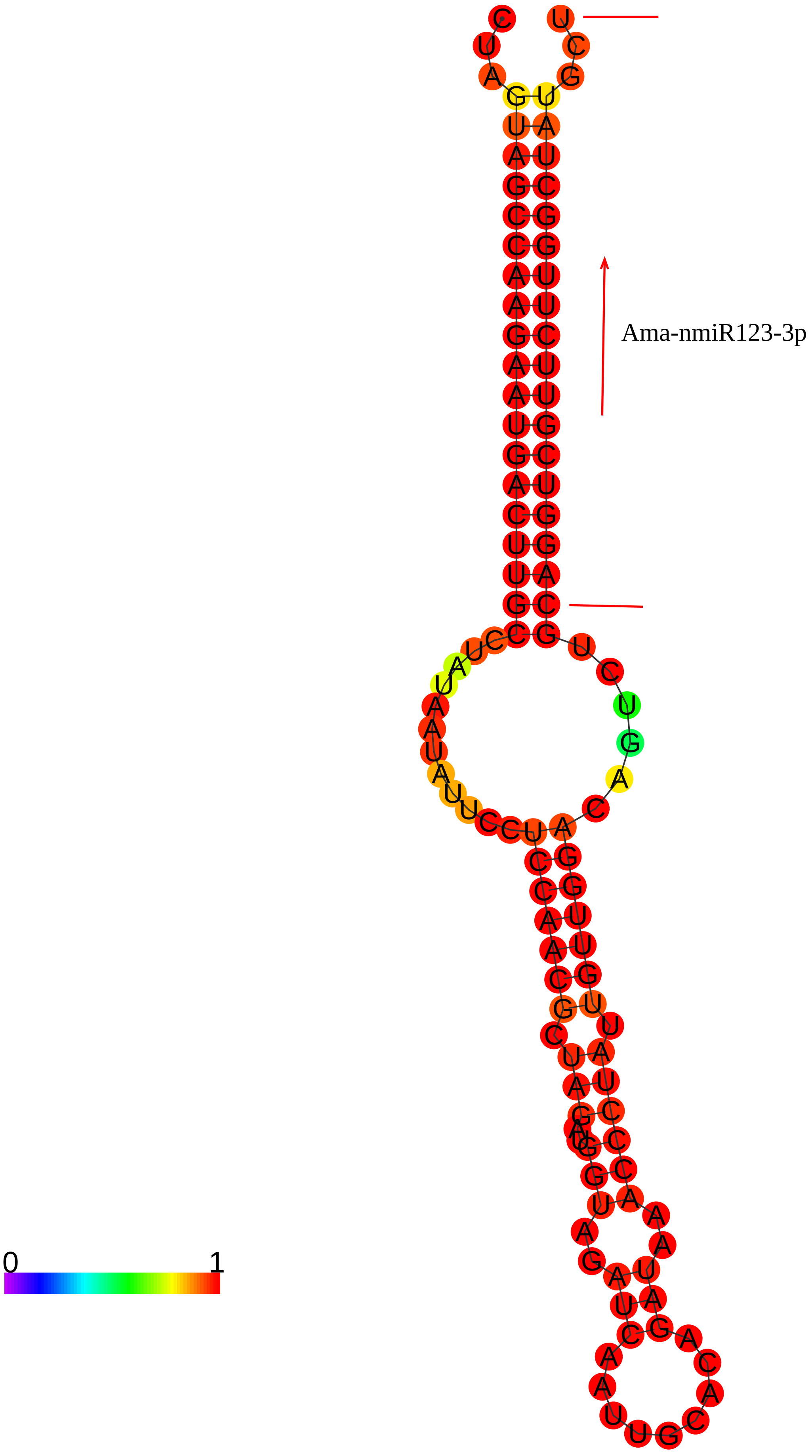
Ama-nmiR123-3p
0
1

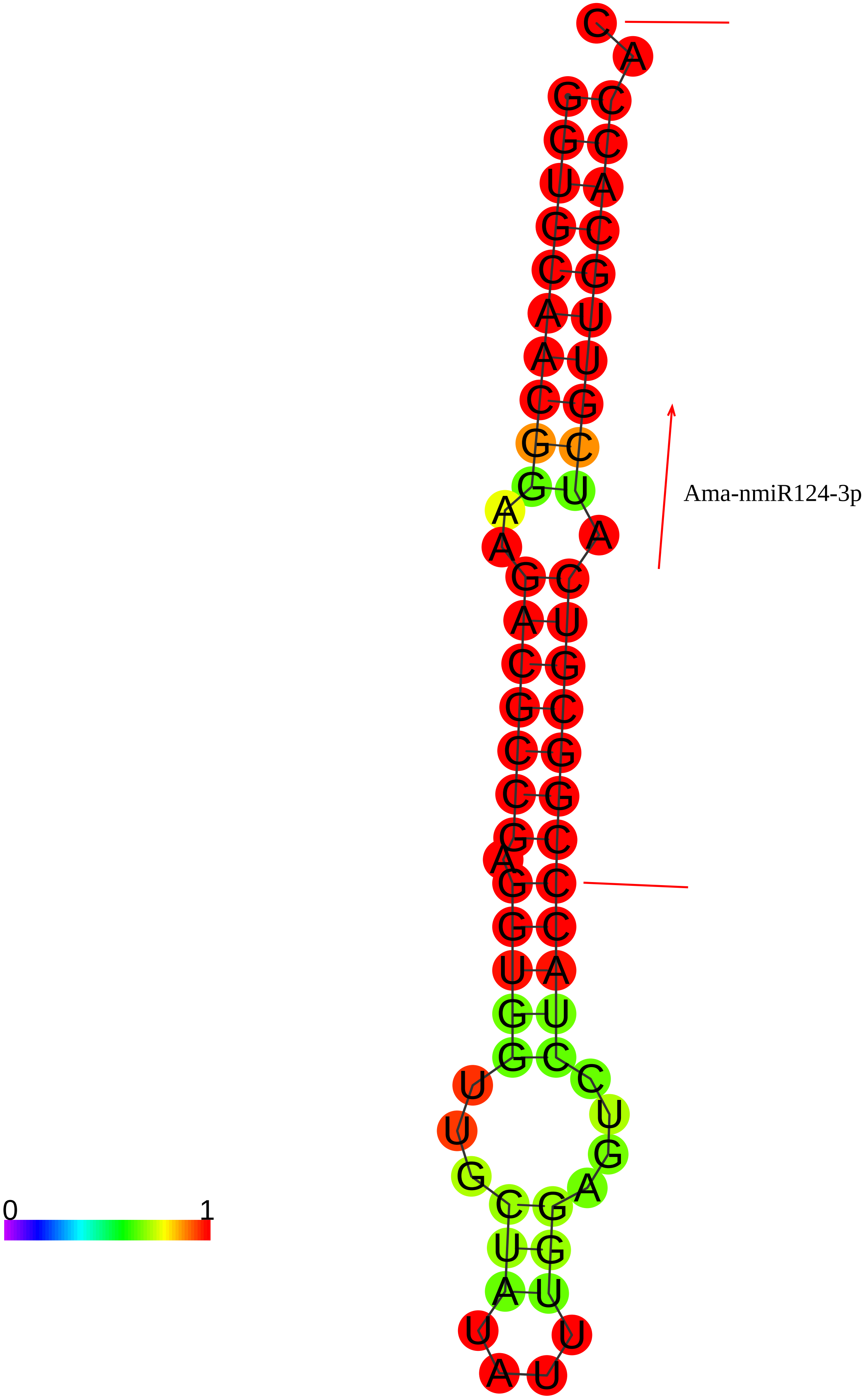

Ama-nmiR124-3p
0
1

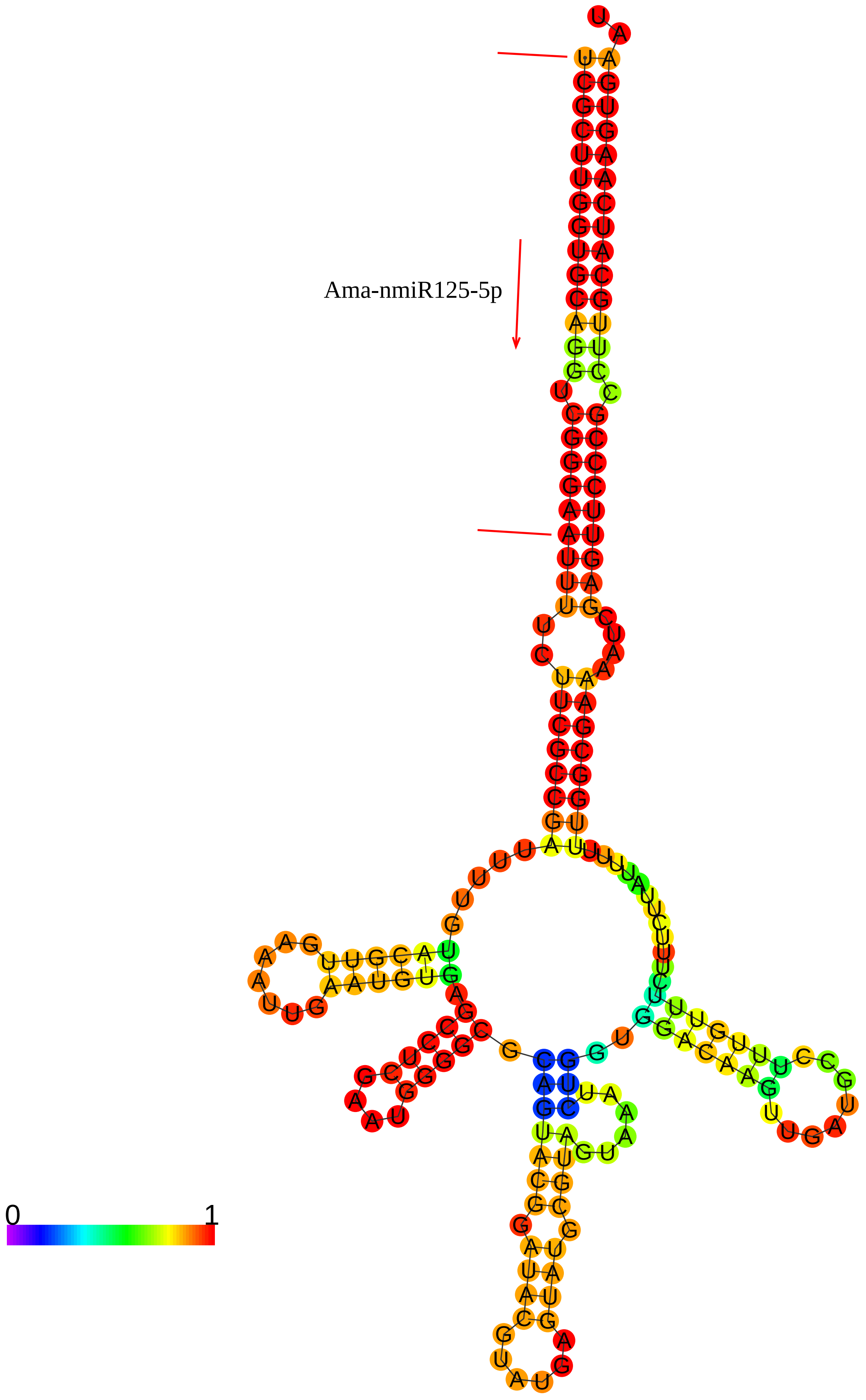
Ama-nmiR125-5p
0
1

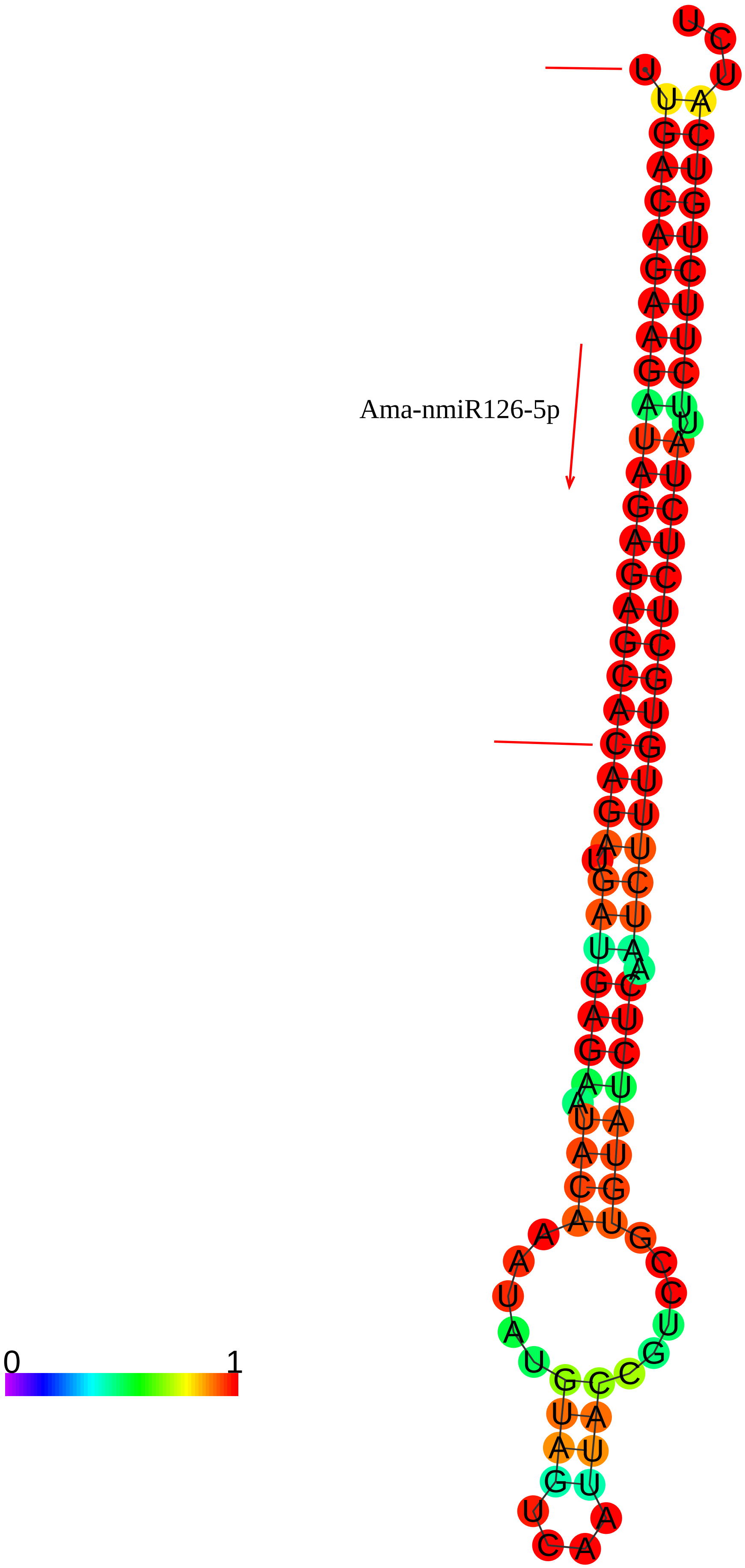
Ama-nmiR126-5p
0
1

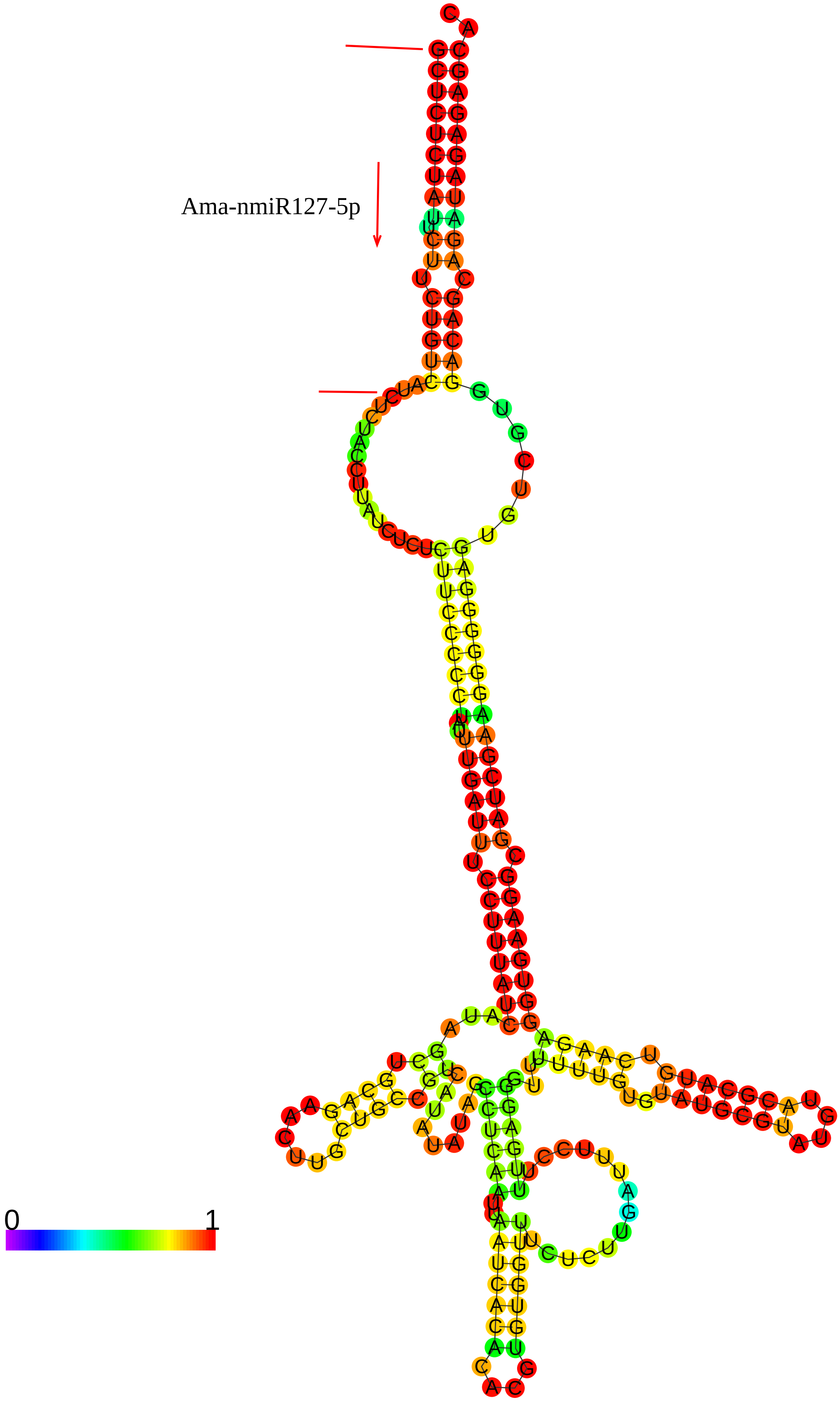
Ama-nmiR127-5p
0
1

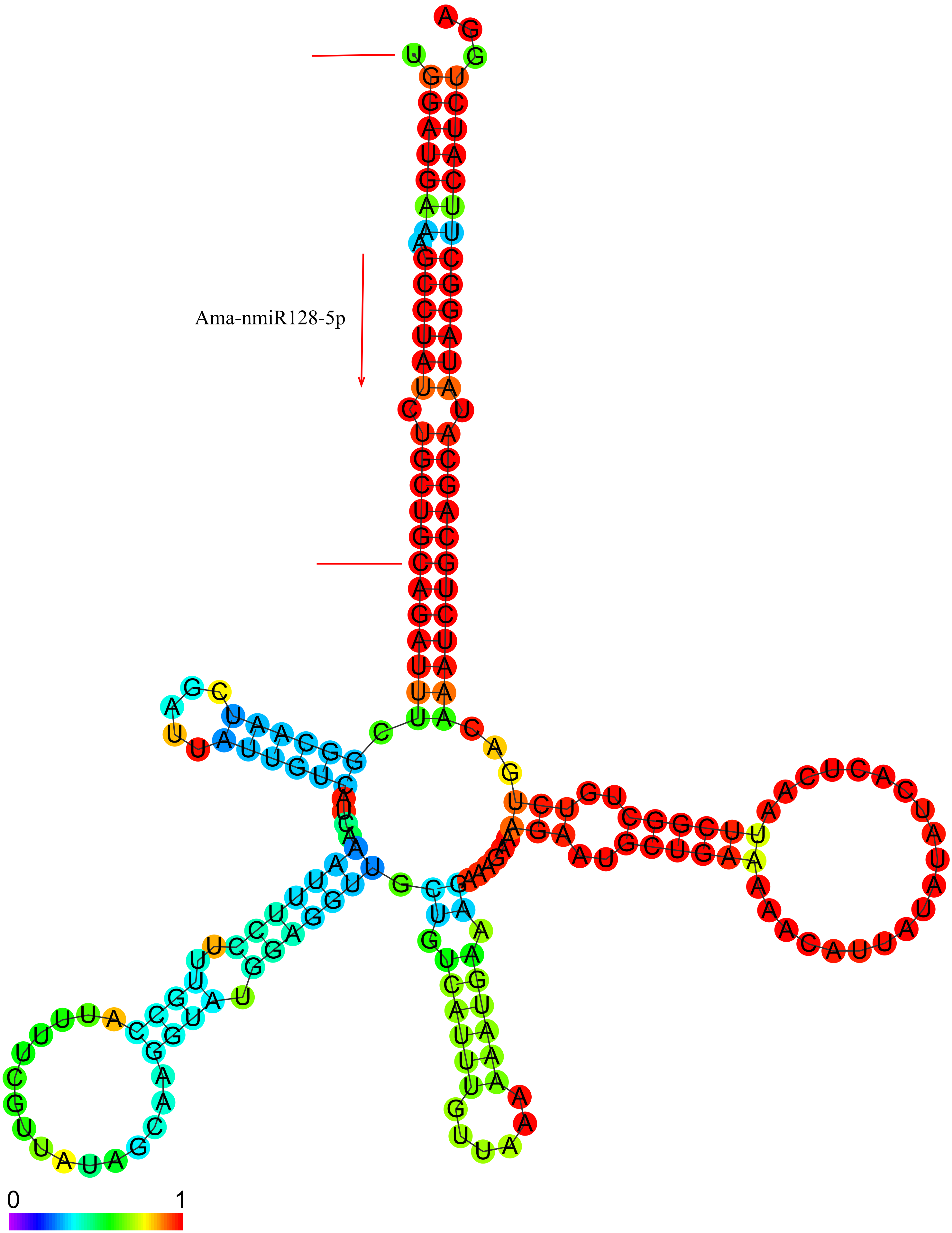
Ama-nmiR128-5p
0
1

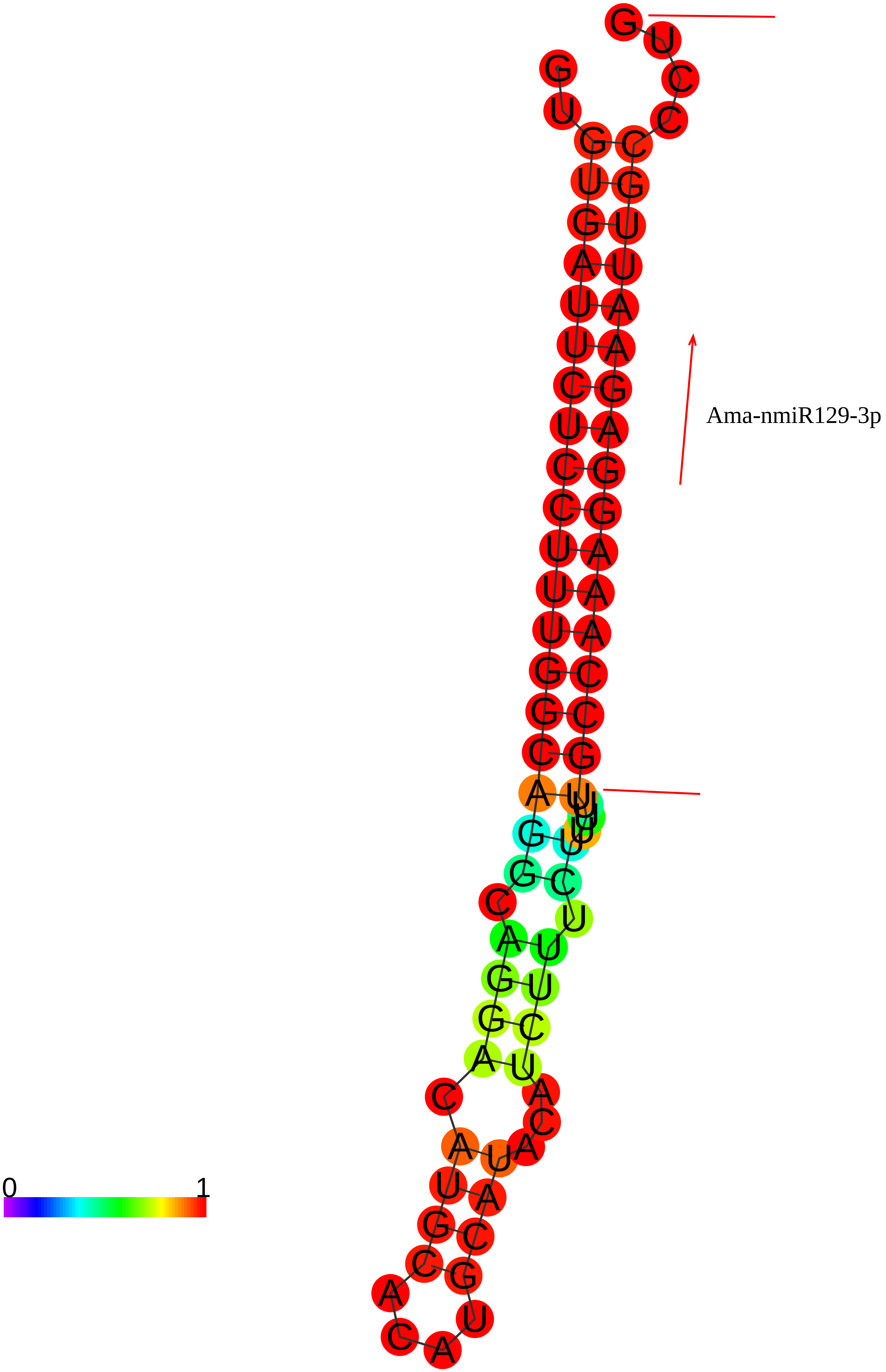
Ama-nmiR129-3p
0
1

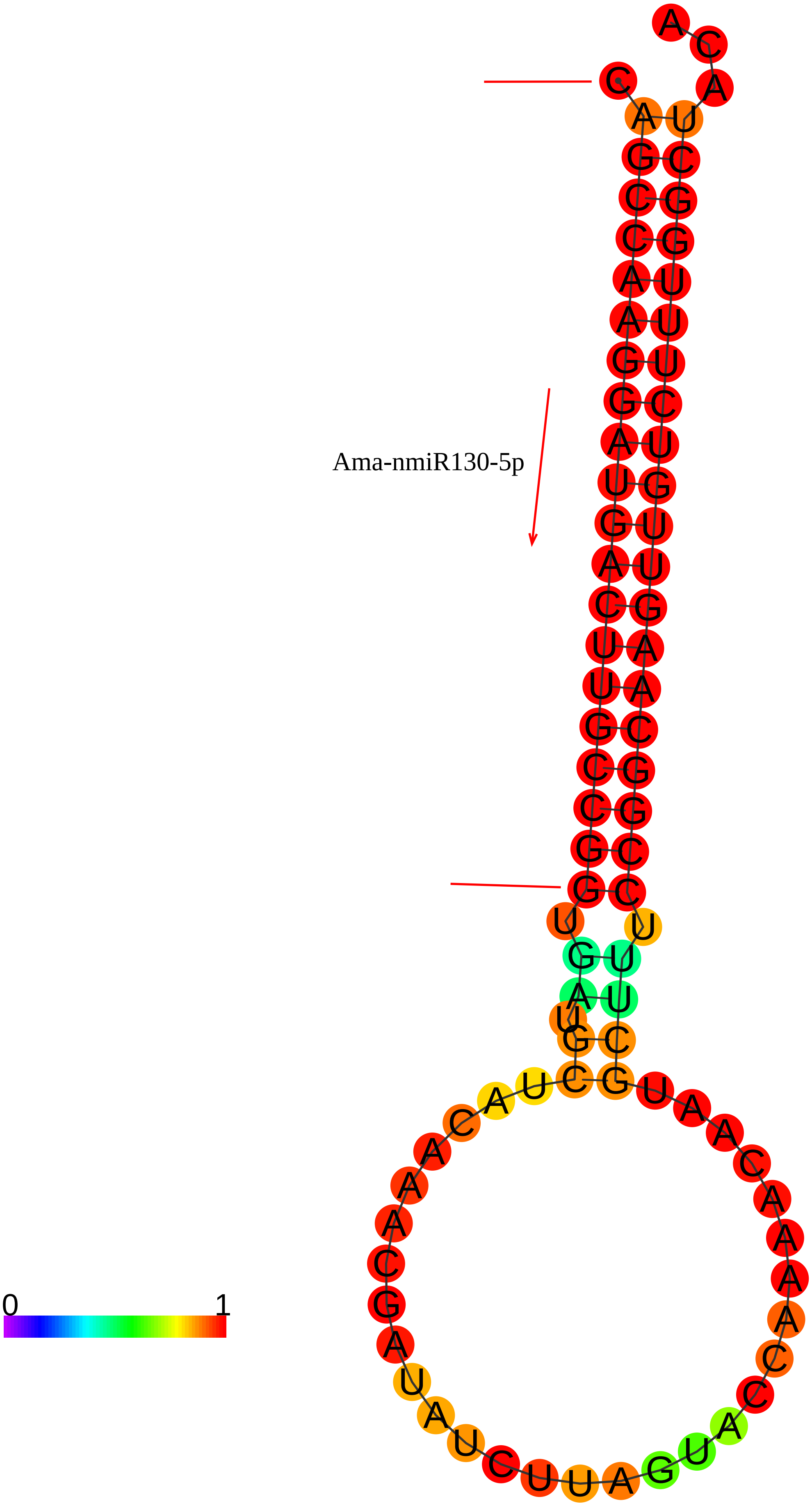
Ama-nmiR130-5p
0
1

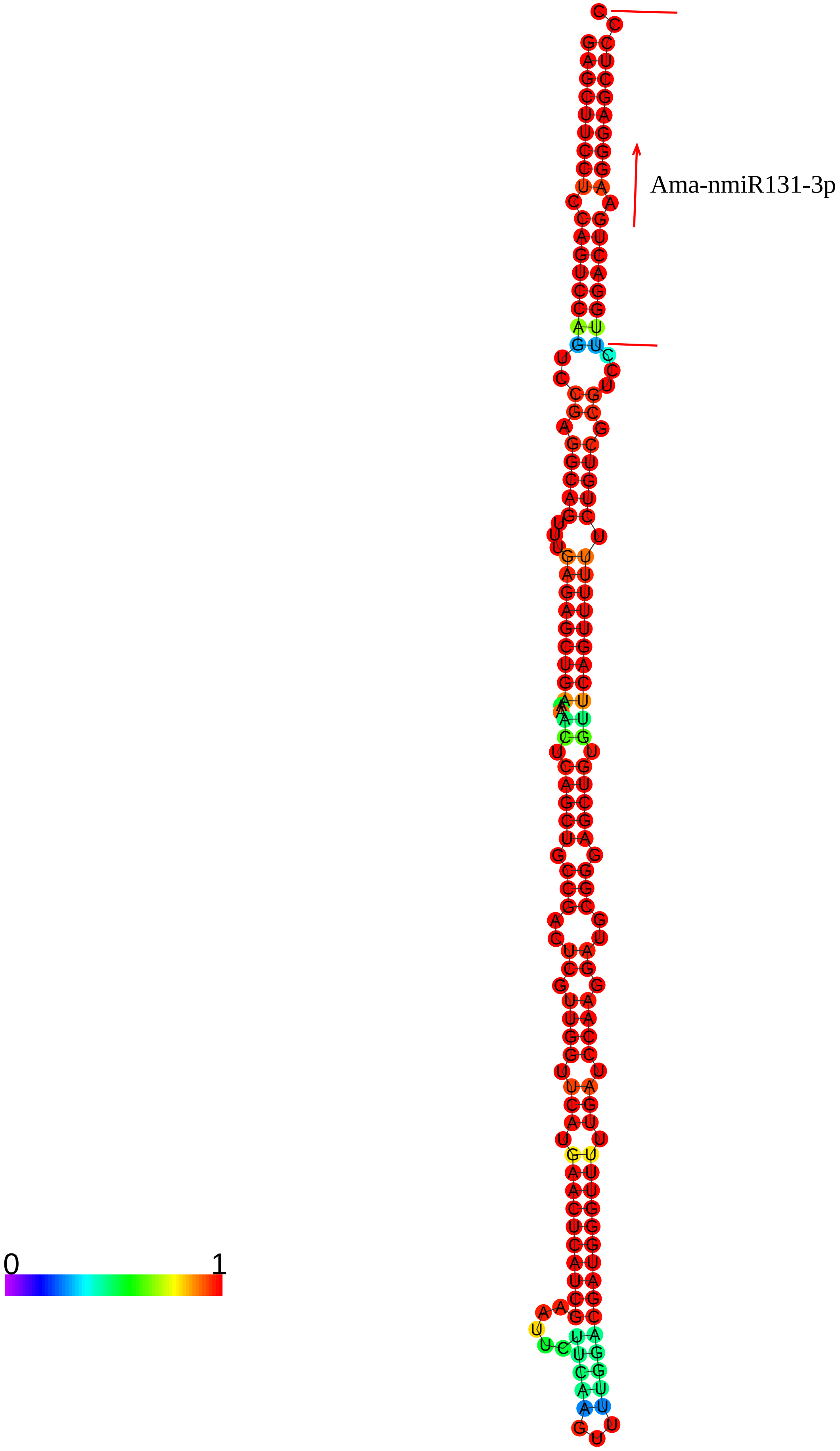
Ama-nmiR131-3p
0
1

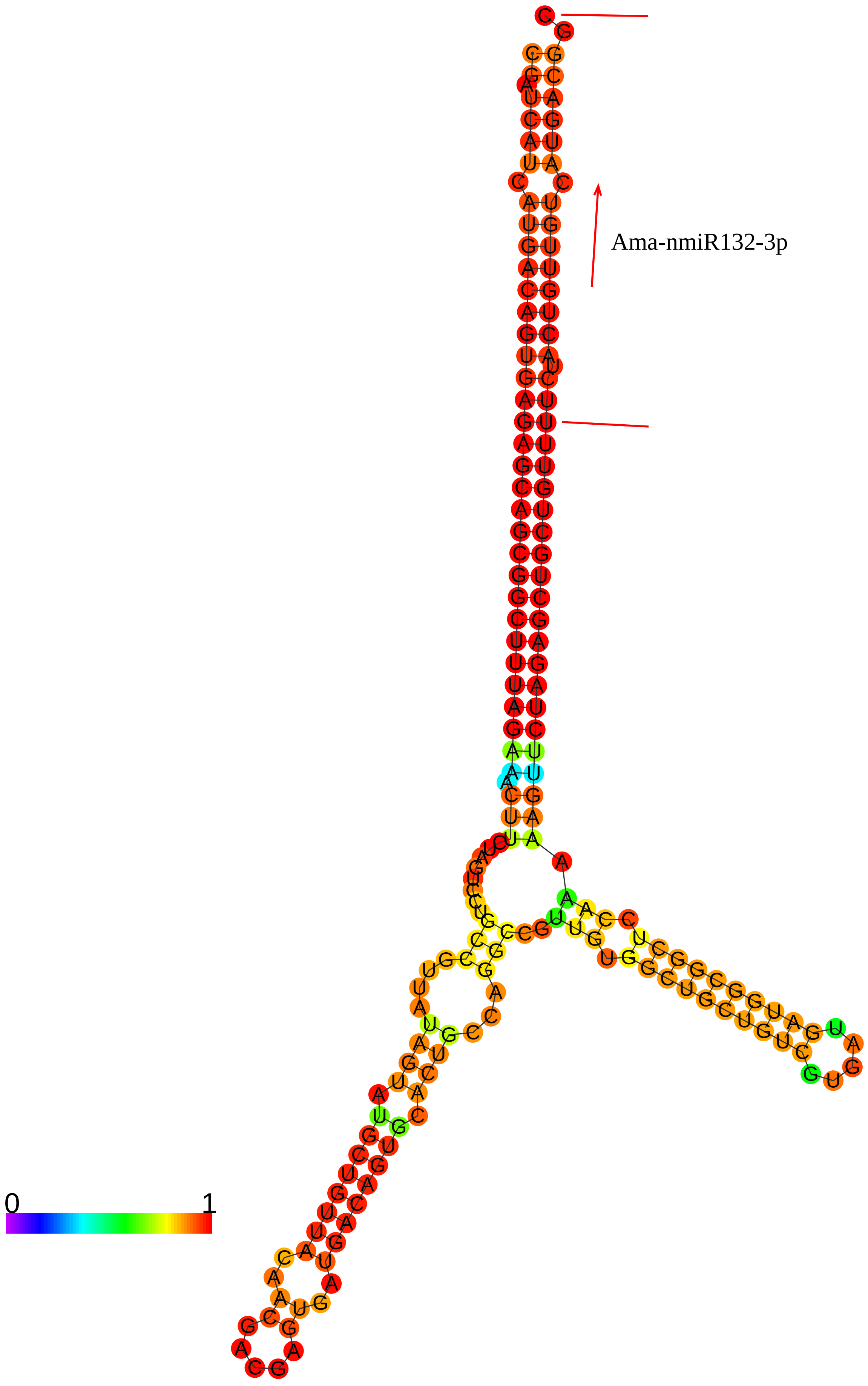
Ama-nmiR132-3p
0
1

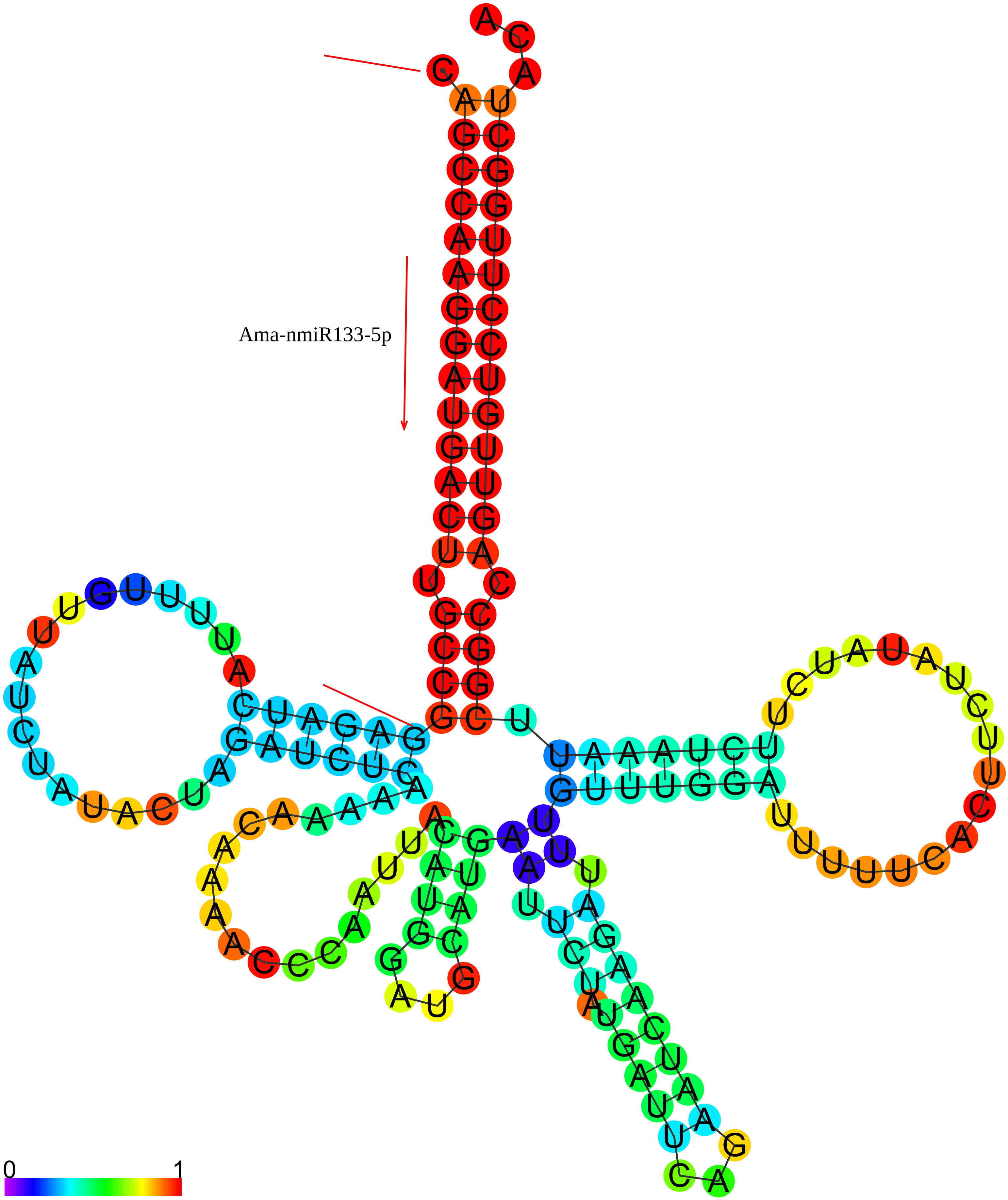
Ama-nmiR133-5p
0
1

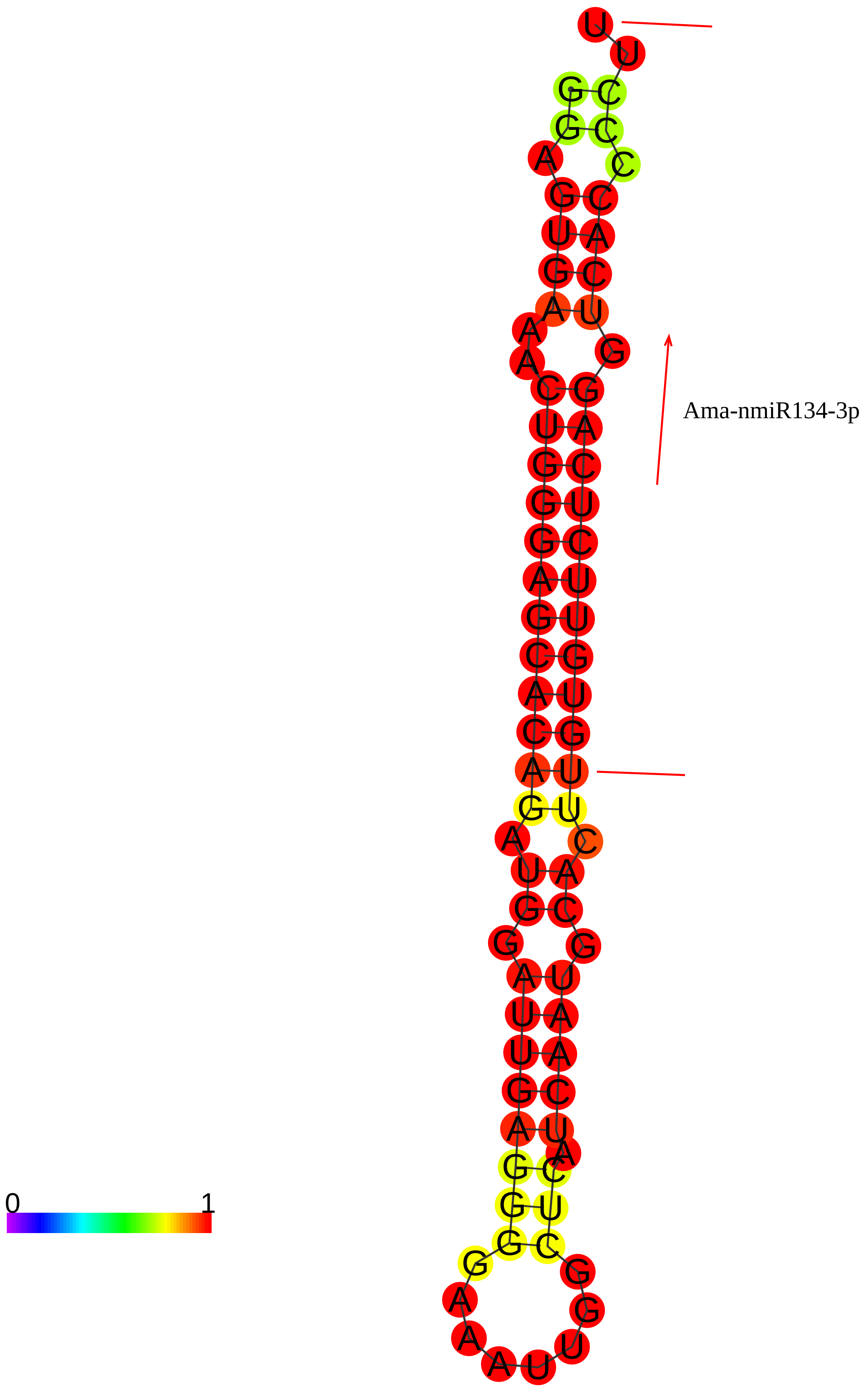
Ama-nmiR134-3p
0
1

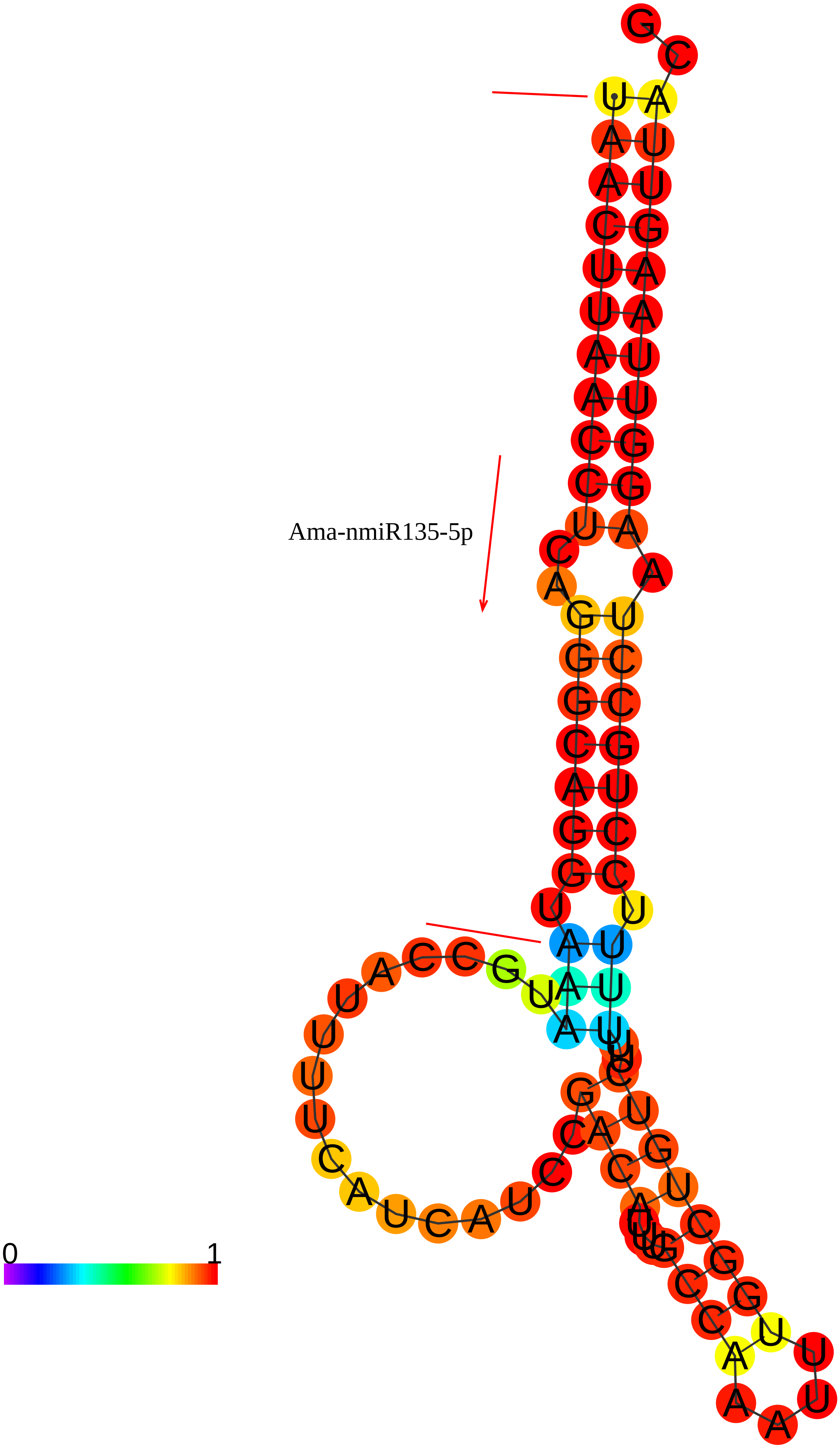
Ama-nmiR135-5p
0
1

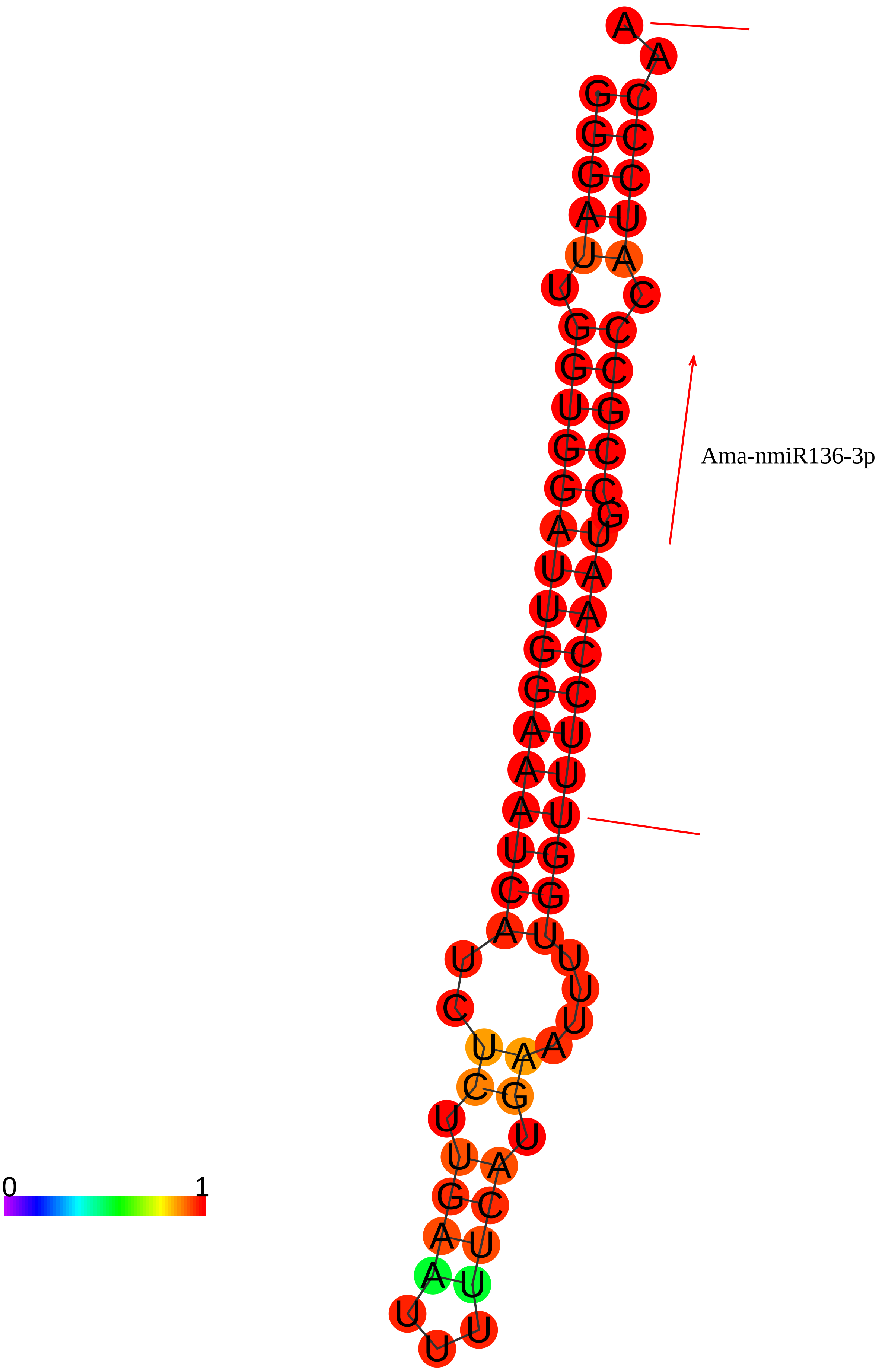
Ama-nmiR136-3p
0
1

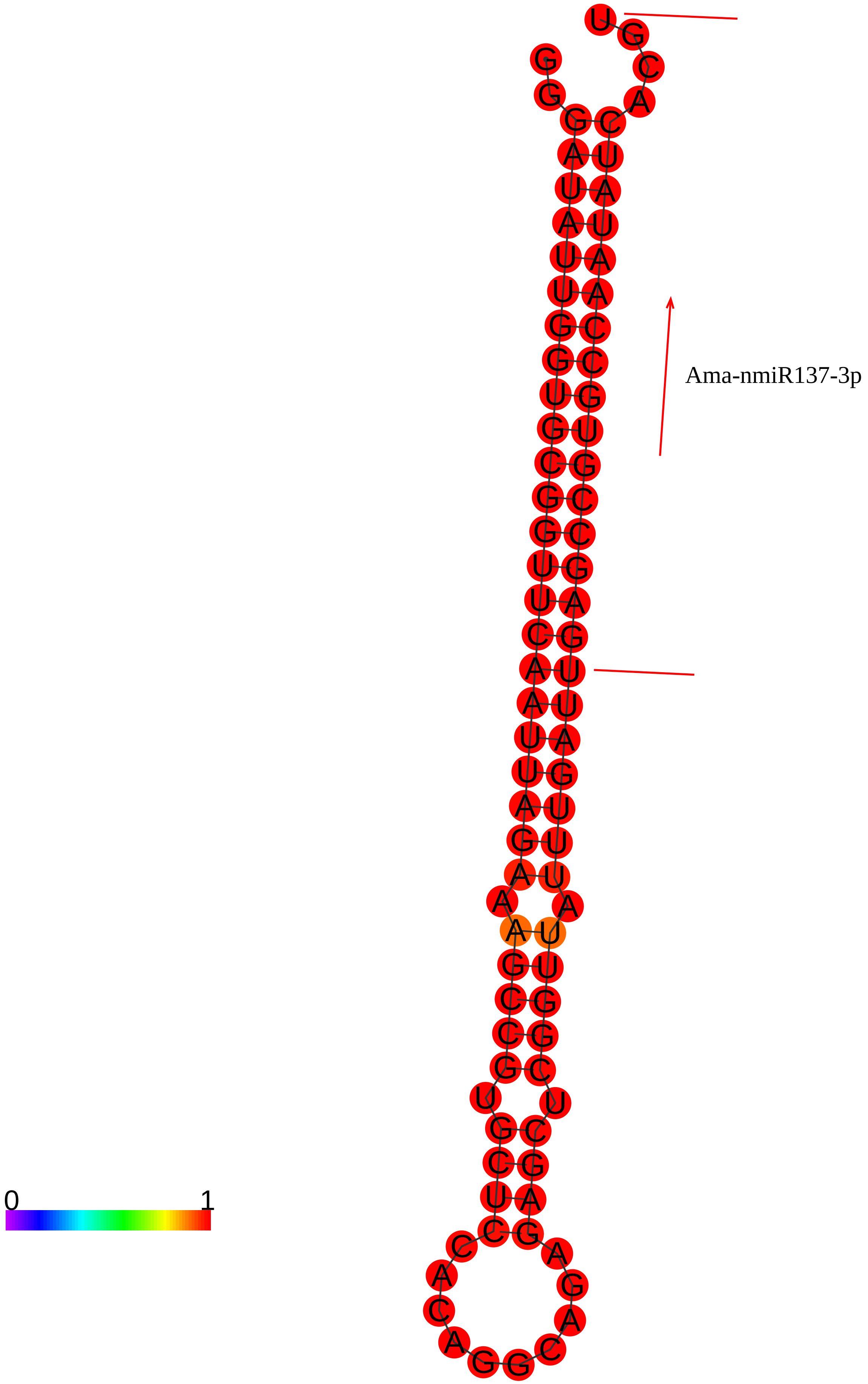
Ama-nmiR137-3p
0
1

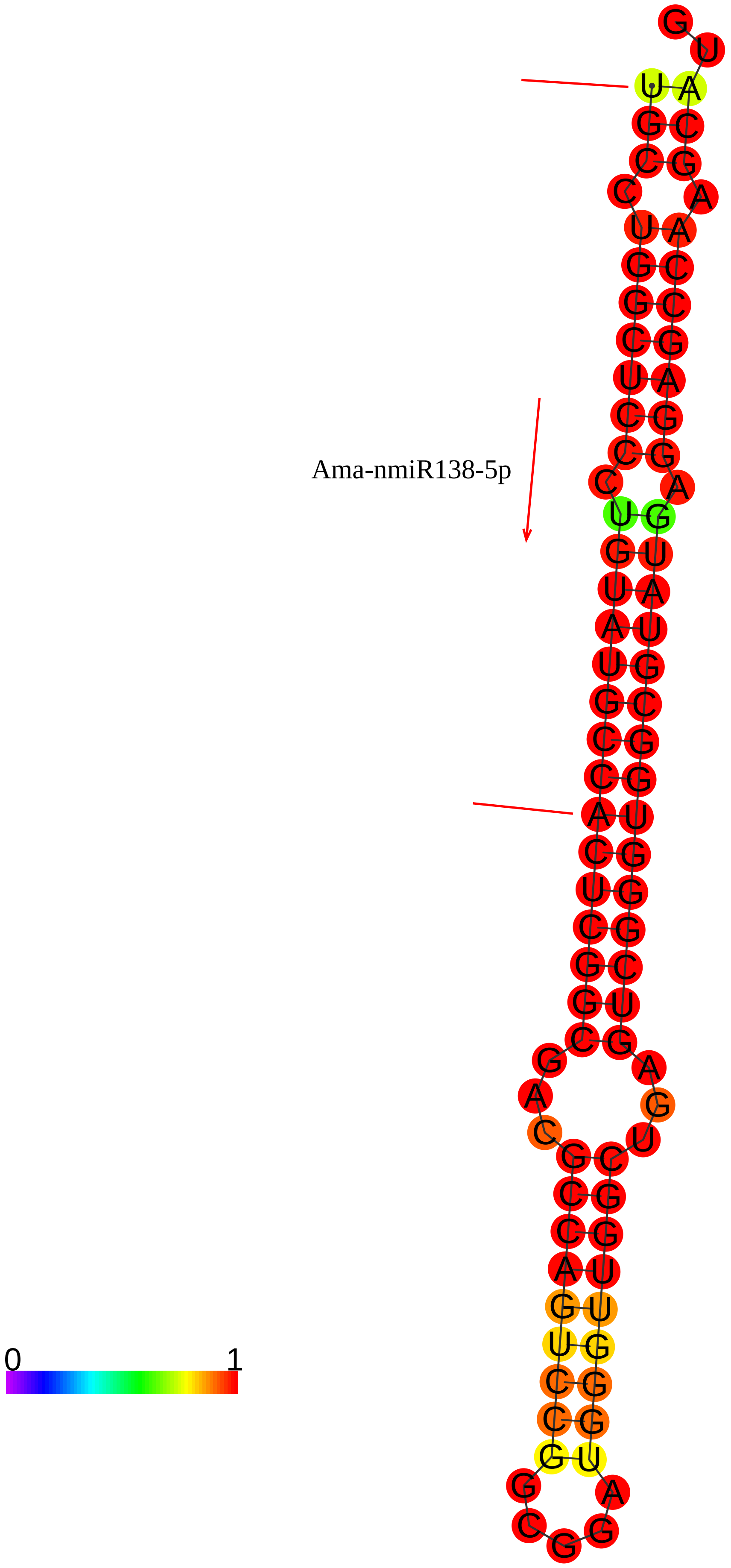
Ama-nmiR138-5p
0
1

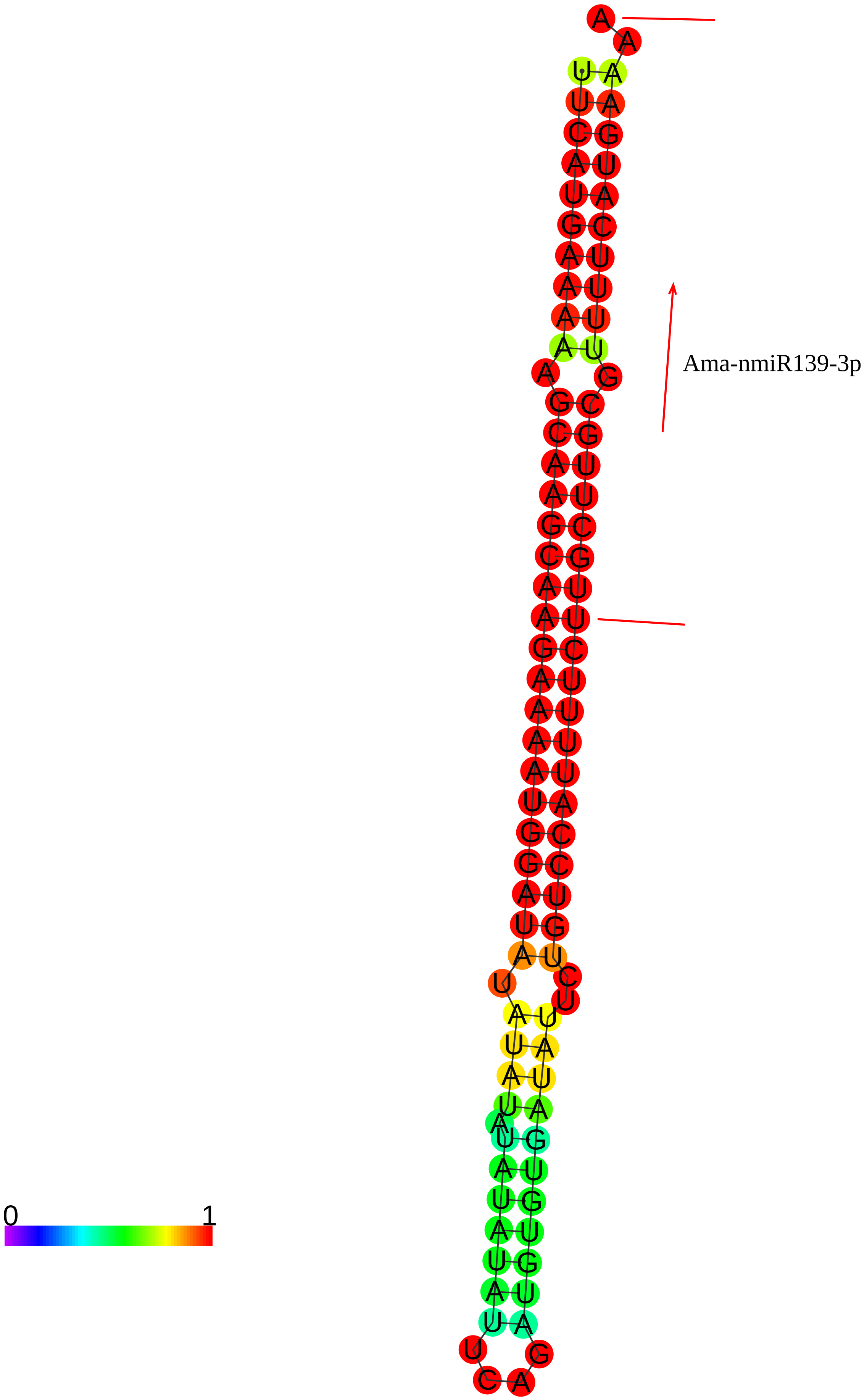
Ama-nmiR139-3p
0
1

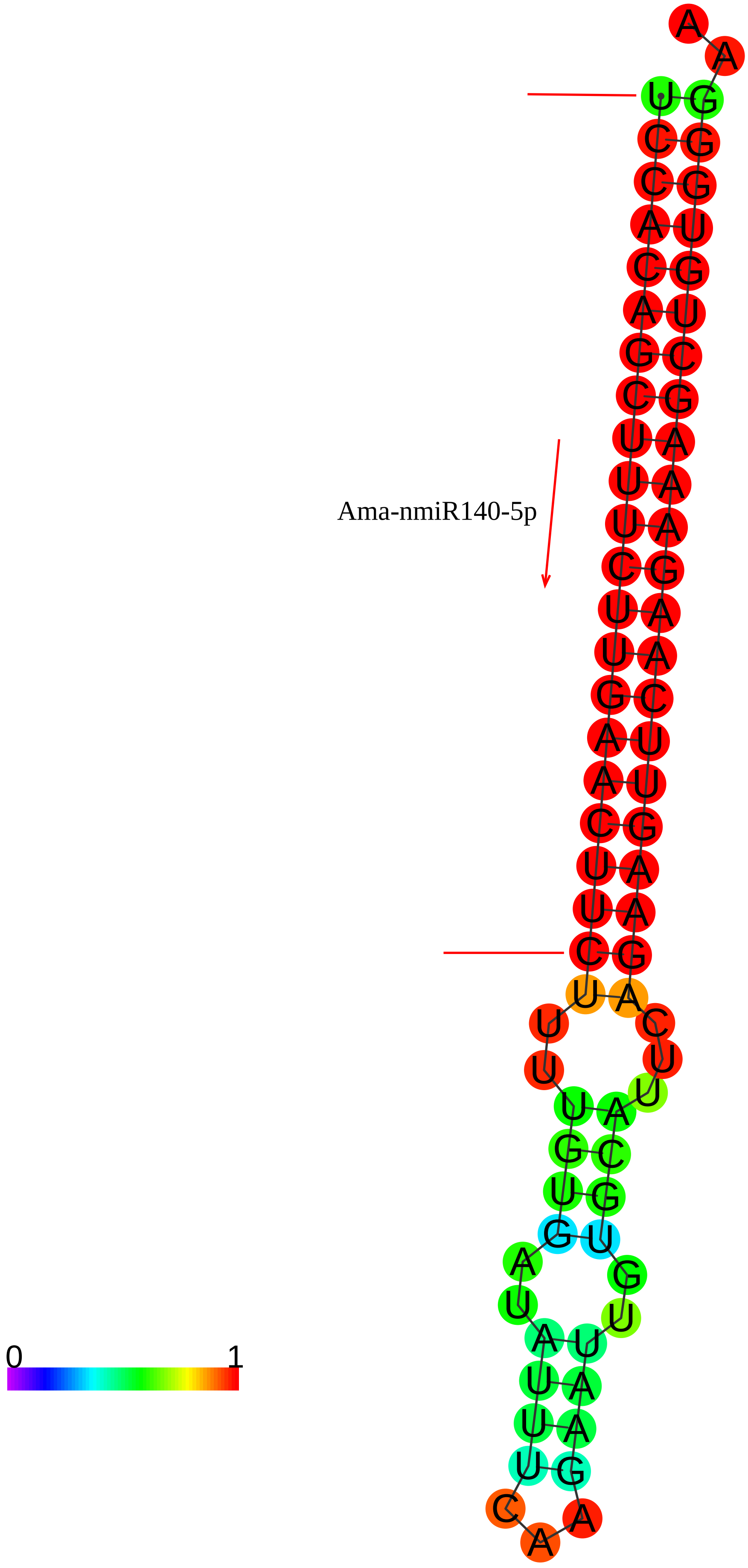
Ama-nmiR140-5p
0
1

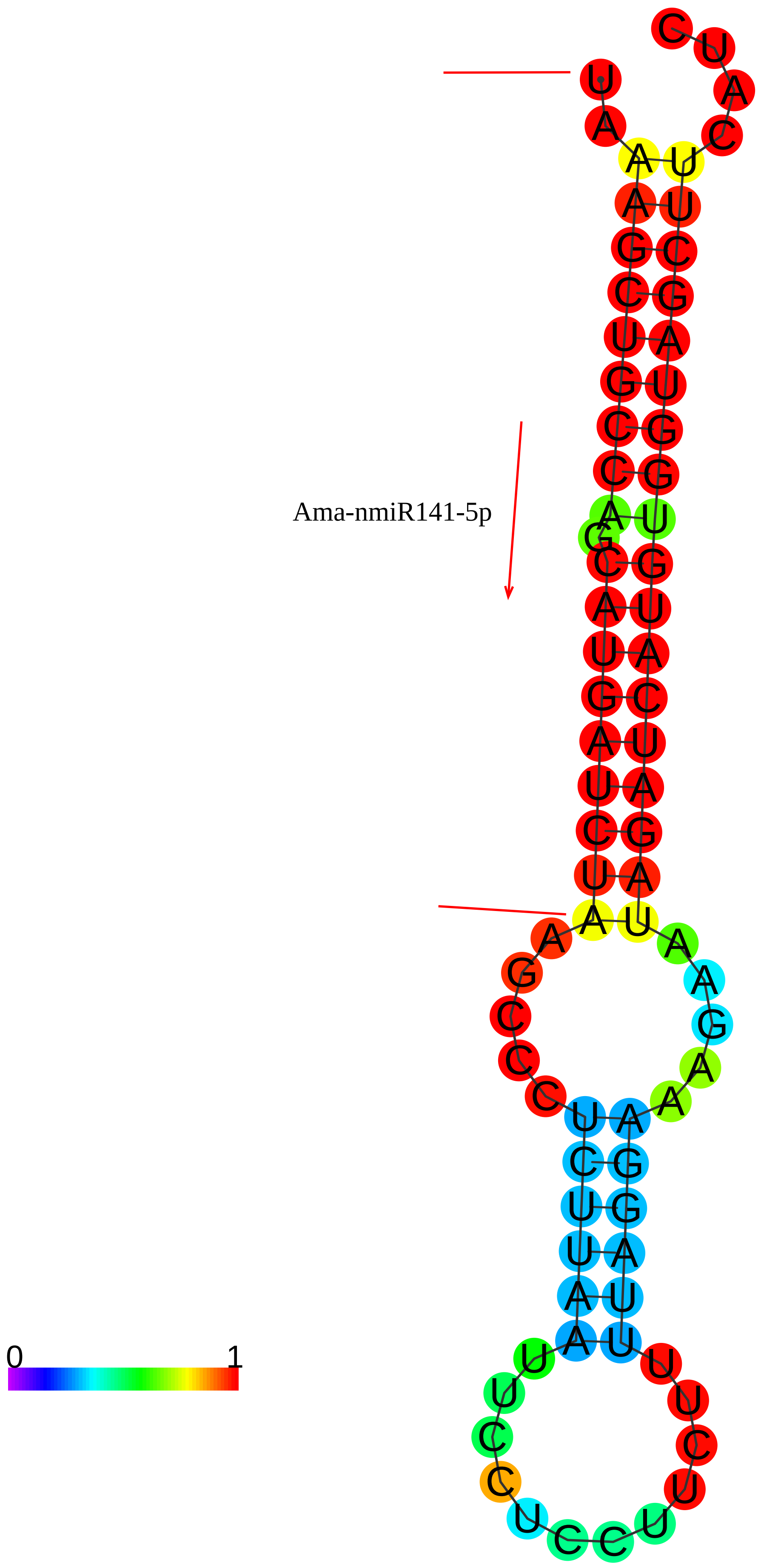
Ama-nmiR141-5p
0
1

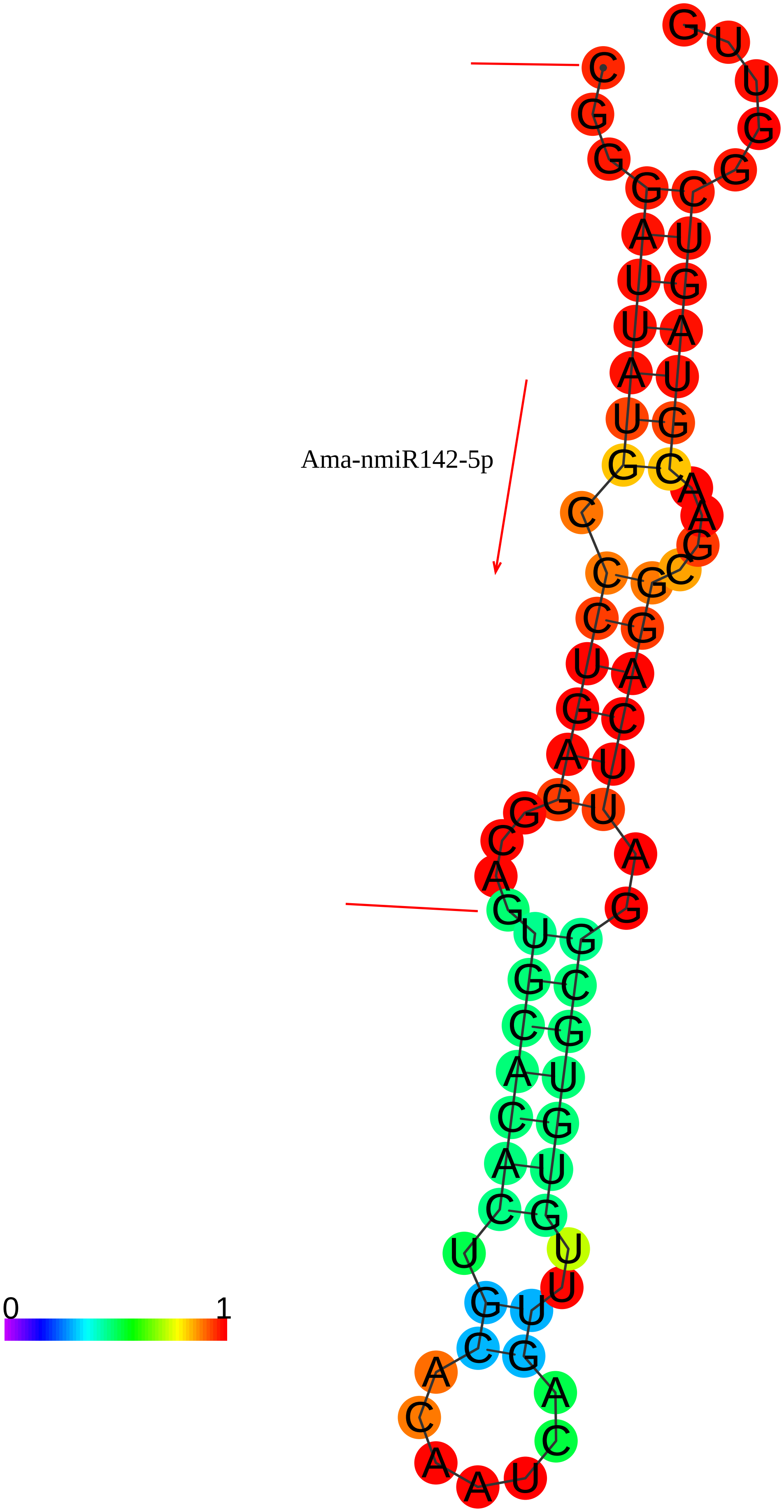
Ama-nmiR142-5p
0
1

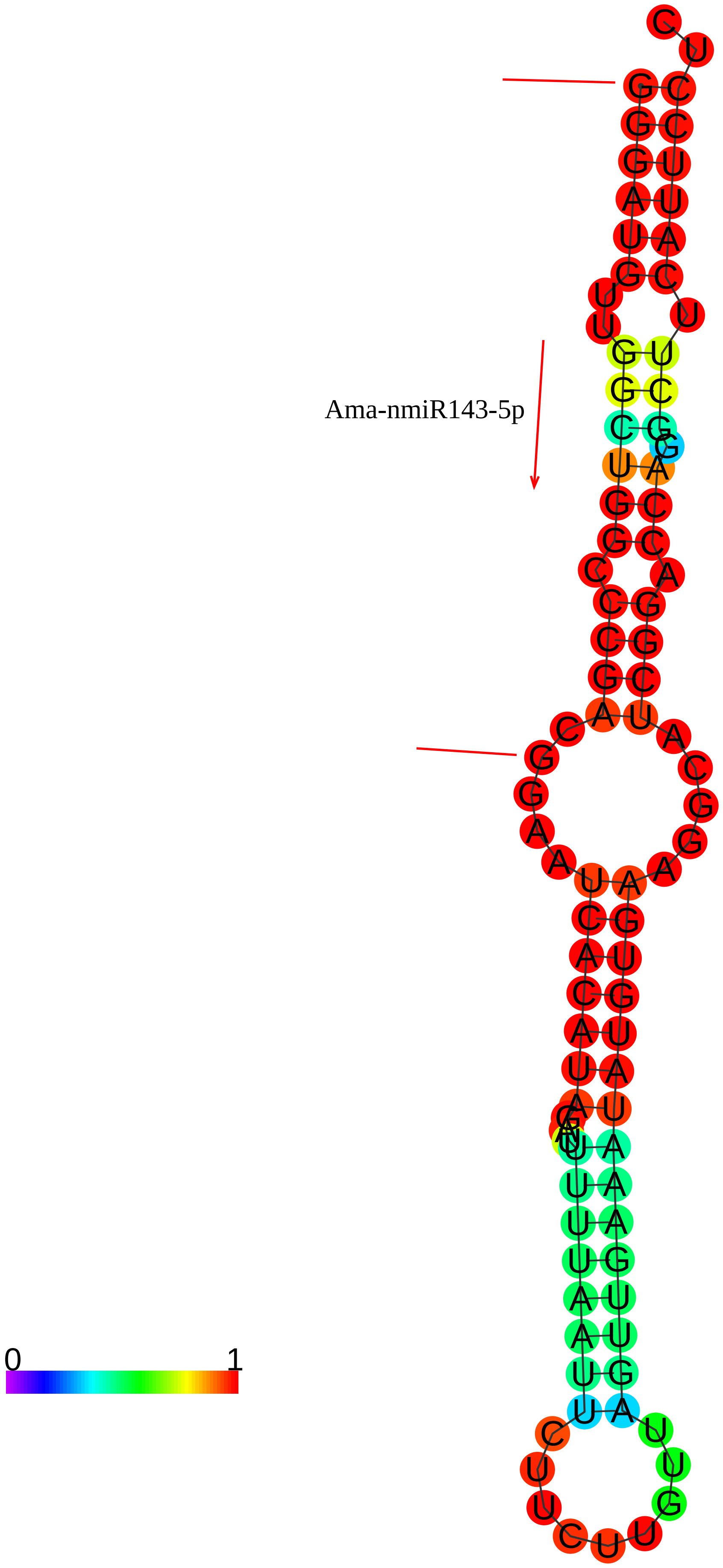
Ama-nmiR143-5p
0
1

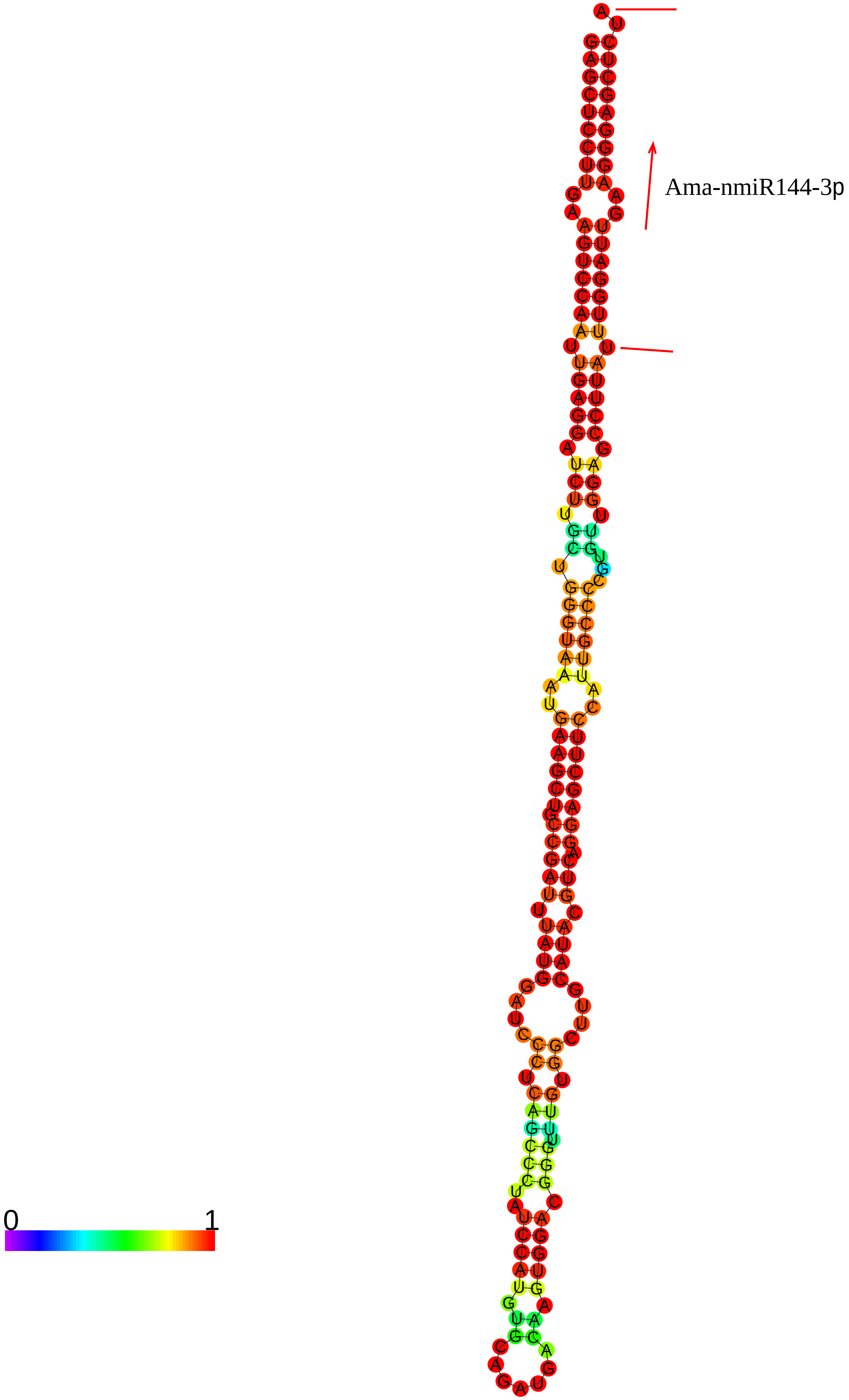
Ama-nmiR144-3p
0
1

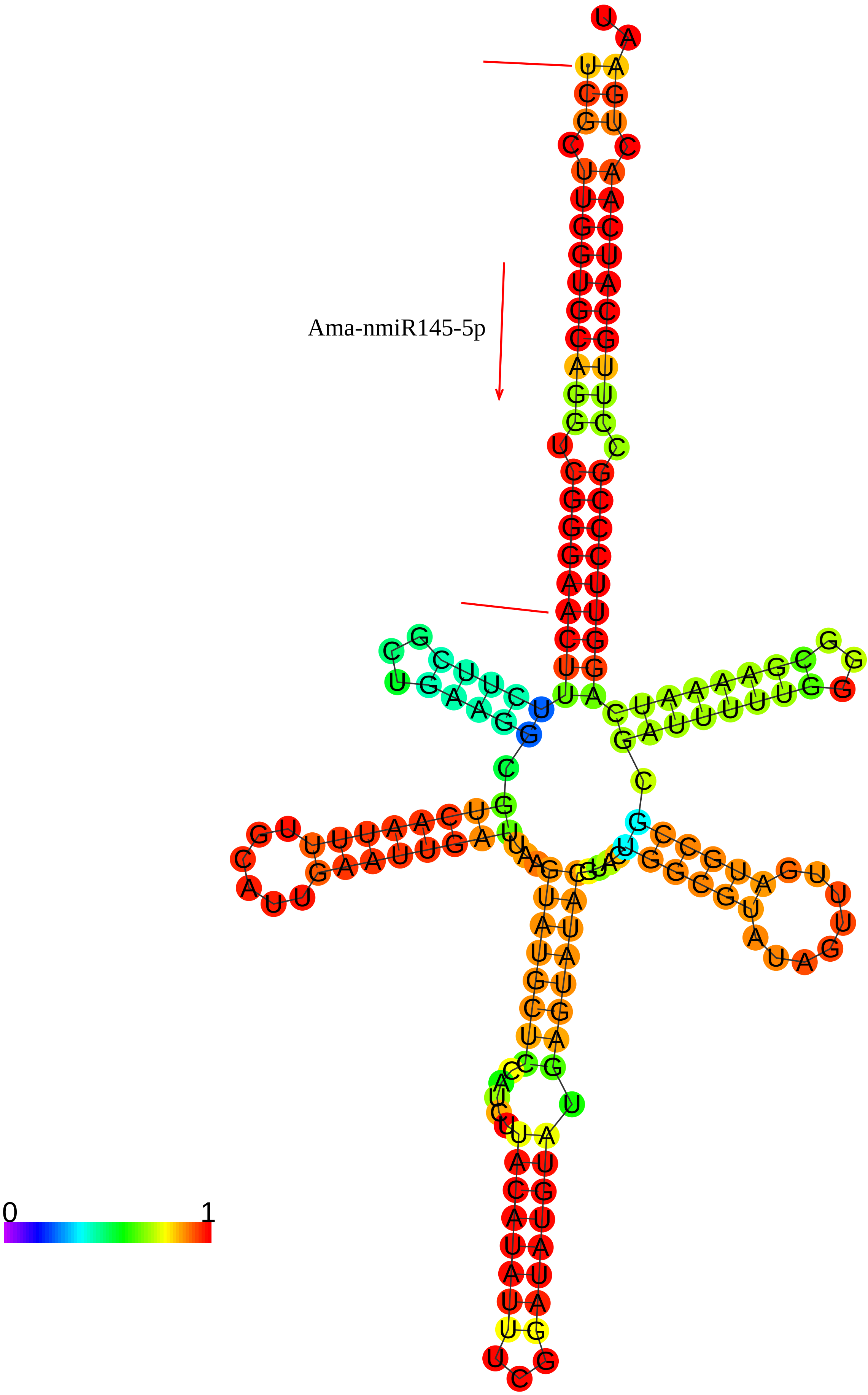

Ama-nmiR145-5p
0
1

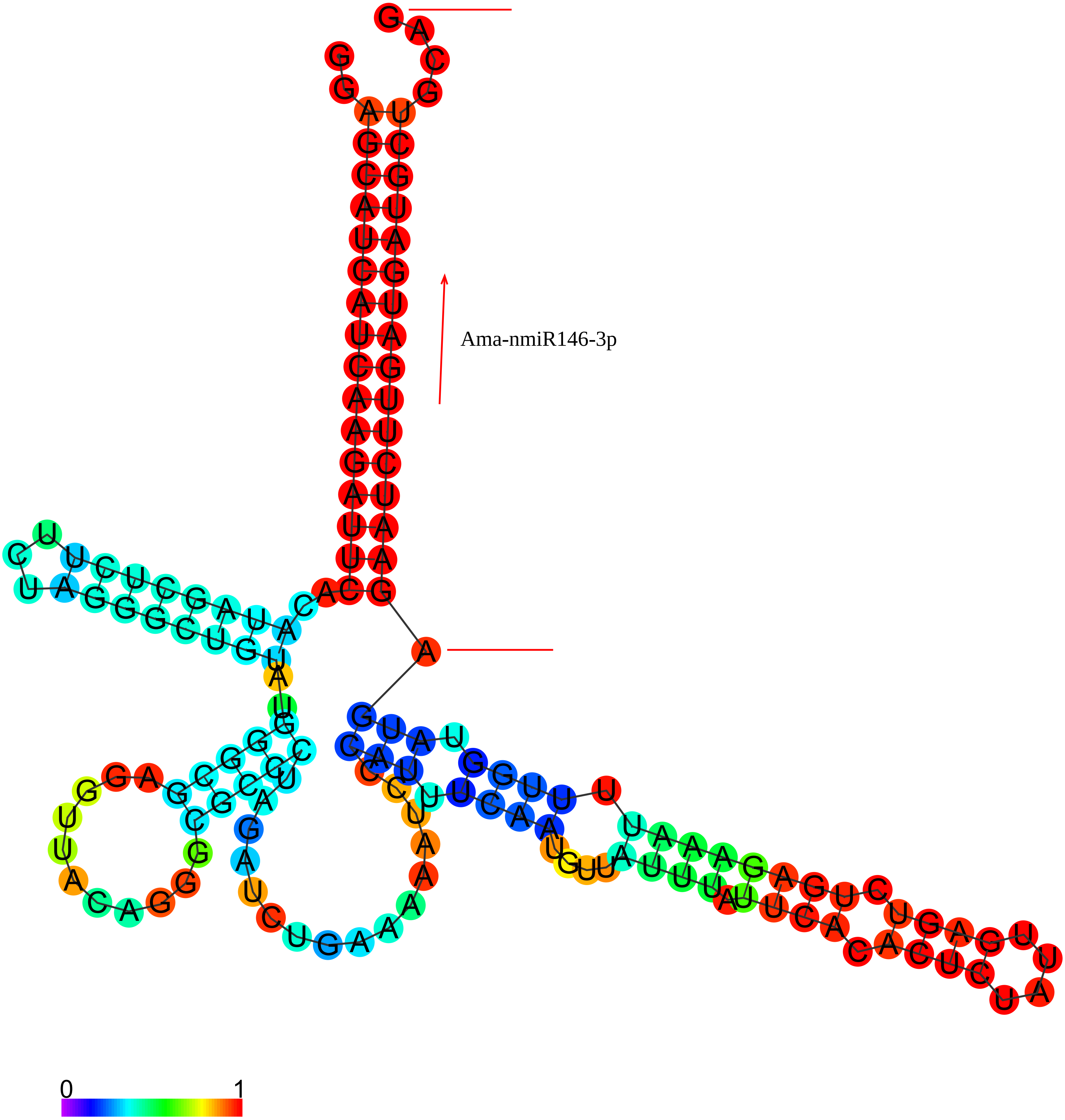
Ama-nmiR146-3p
0
1

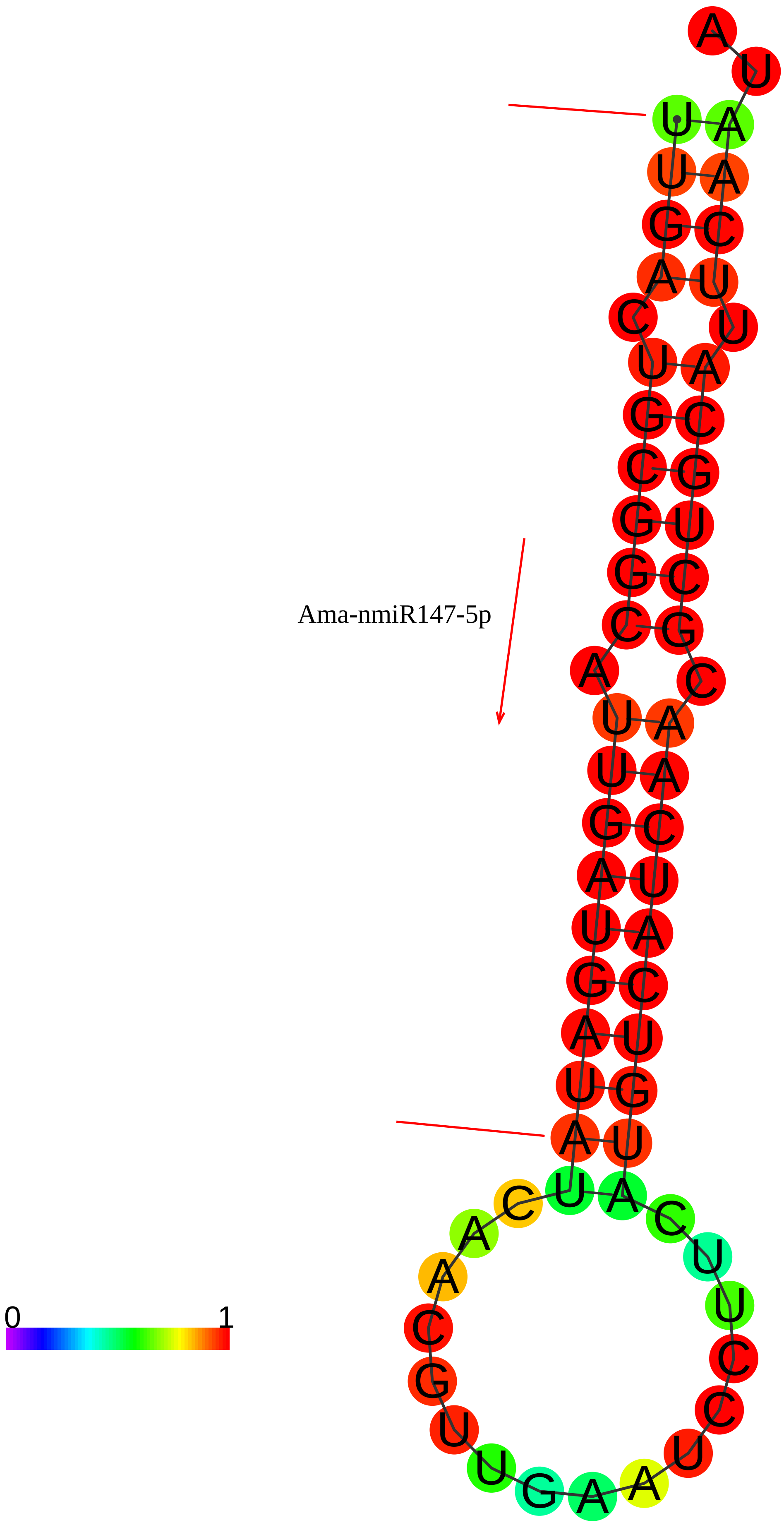

Ama-nmiR147-5p
0
1

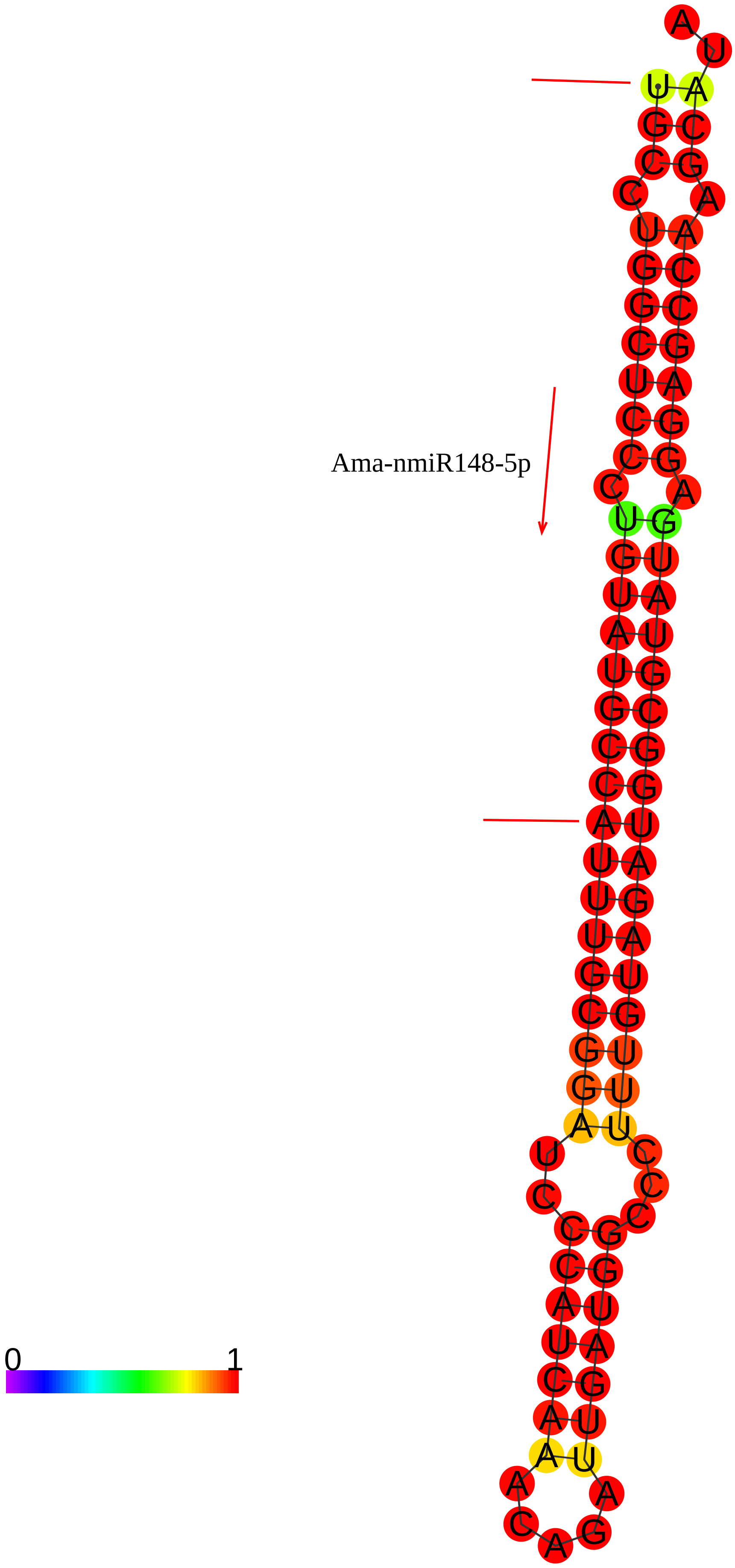
Ama-nmiR148-5p
0
1

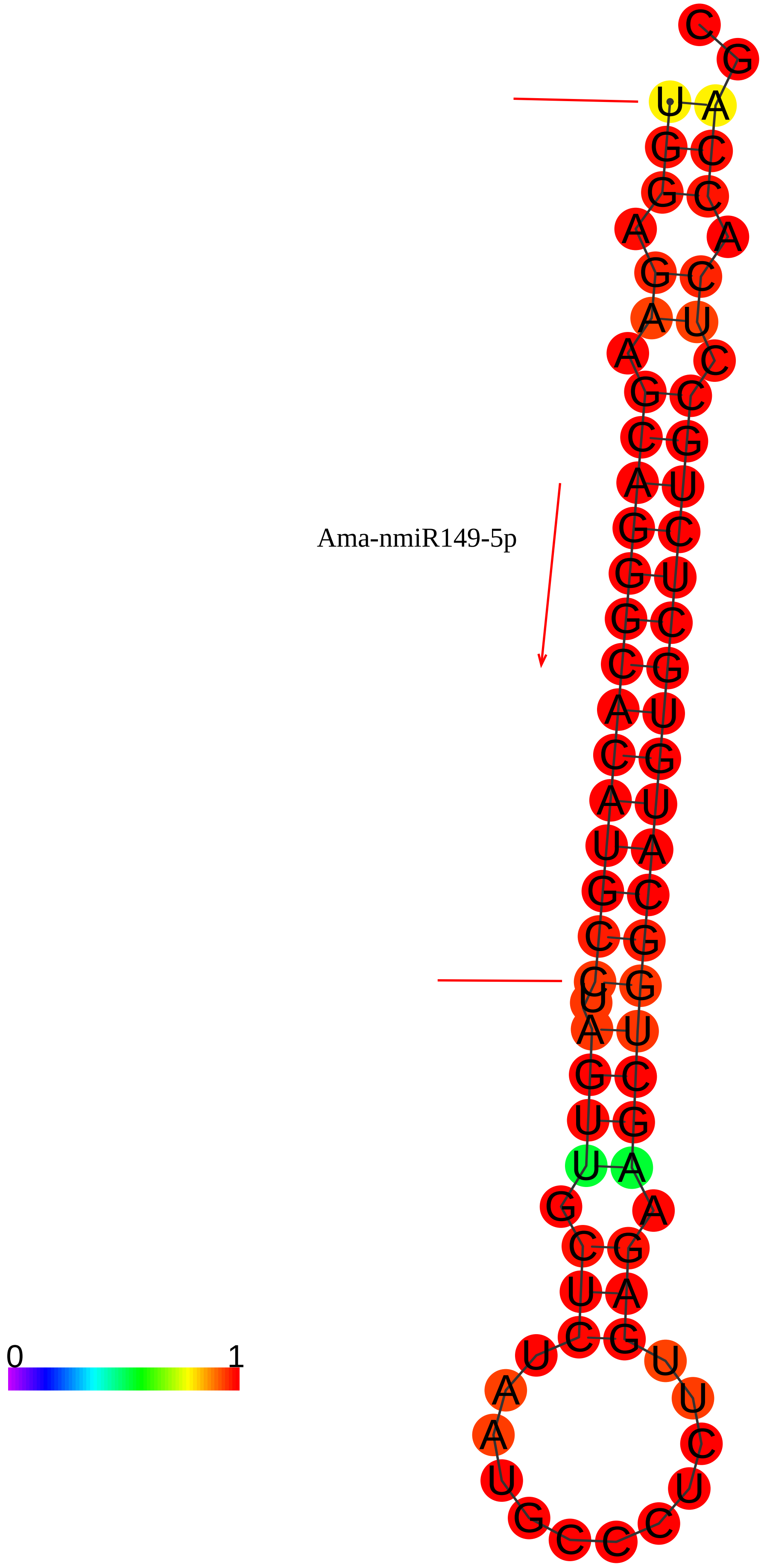

Figure S4. Hairpin structures of novel miRNA sidentifiedin *A.marina.*

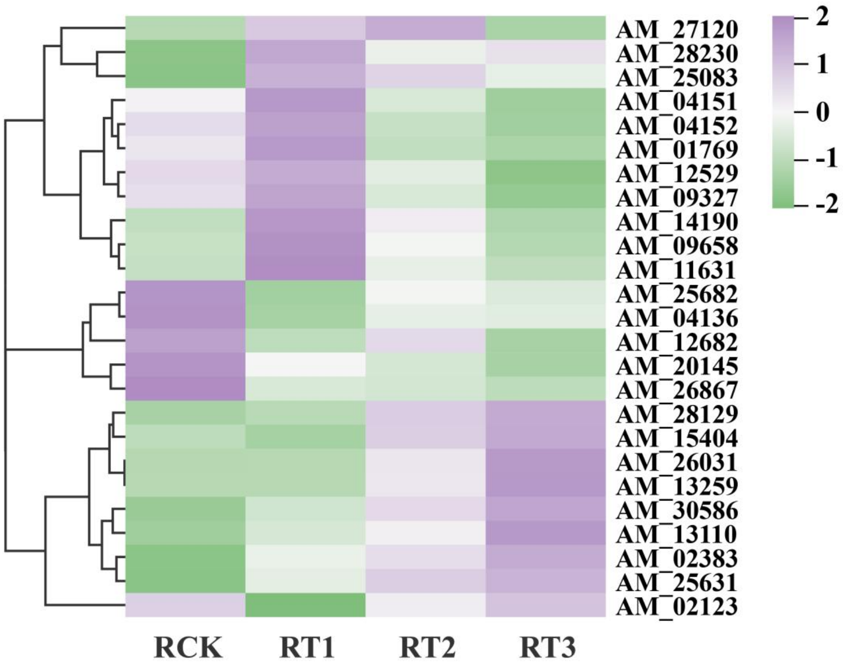

Figure S5. Heatmap of expression levels for differentially expressed target genes in *A. marina*.

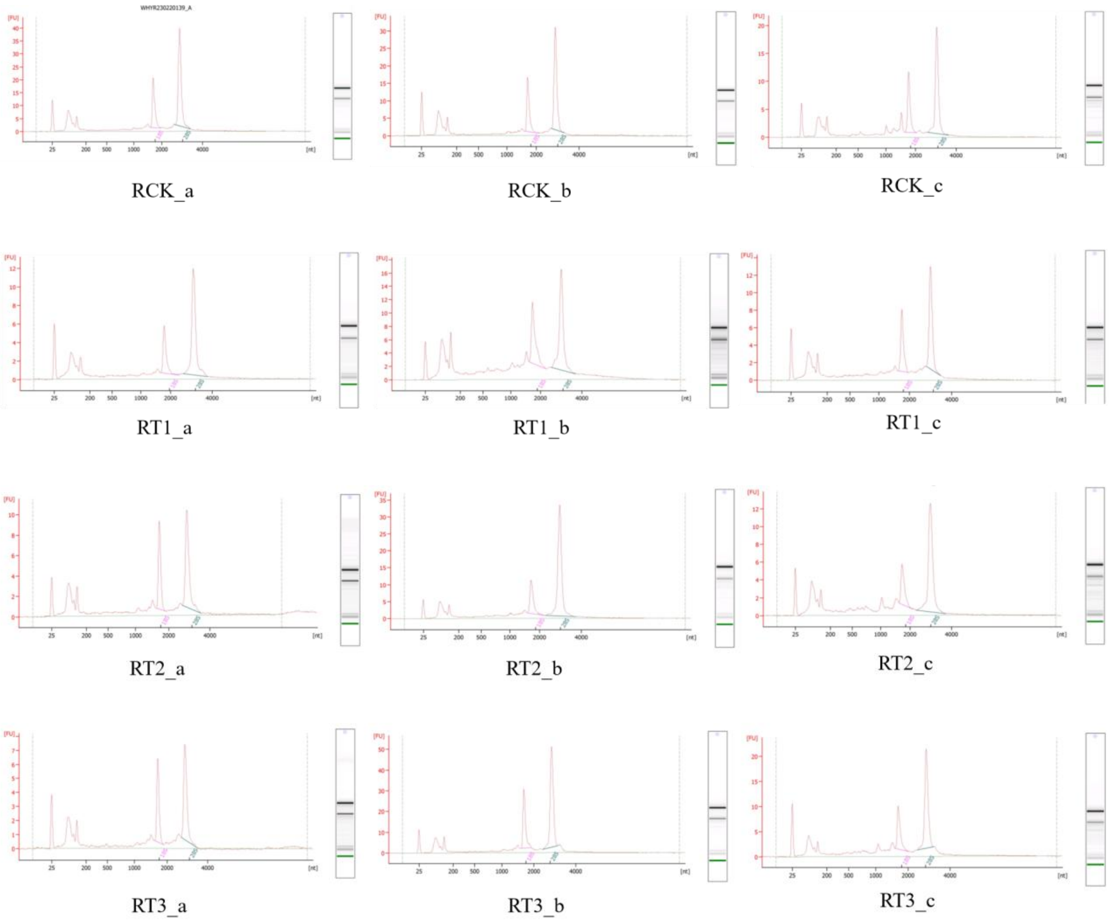

Figure S6. RNA quality assessment using Agilent 2100.
